# GWAS of self-reported mosquito bite size, itch intensity and attractiveness to mosquitoes implicates immune-related predisposition loci

**DOI:** 10.1093/hmg/ddx036

**Published:** 2017-02-11

**Authors:** Amy V. Jones, Mera Tilley, Alex Gutteridge, Craig Hyde, Michael Nagle, Daniel Ziemek, Donal Gorman, Eric B. Fauman, Xing Chen, Melissa R. Miller, Chao Tian, Youna Hu, David A. Hinds, Peter Cox, Serena Scollen

**Affiliations:** 1 Pfizer WRD, Human Genetics and Computational Biomedicine, The Portway Building, Granta Park, Cambridge CB21 6GS, UK; 2 Pfizer WRD, Pharmatherapeutics Clinical R&D, Precision Medicine, 300 Technology Square Fl #3, Cambridge, MA 02139, USA; 3 Pfizer WRD, Computational Sciences CoE, The Portway Building, Granta Park, Cambridge CB21 6GS, UK; 4 Pfizer WRD, Research Statistics, 558 Eastern Point Rd, Groton, CT 06340, USA; 5 Pfizer WRD, Human Genetics and Computational Biomedicine, 610 Main Street S, Cambridge, MA 02139, USA; 6 Pfizer WRD, Computational Sciences CoE, Linkstraße 10, 10785 Berlin, Germany; 7 Pfizer WRD, Research Statistics, The Portway Building, Granta Park, Cambridge CB21 6GS, UK; 8 Pfizer WRD, Computational Sciences CoE, 610 Main Street S, Cambridge, MA 02139, USA; 9 23andMe, Inc, 899 W Evelyn Avenue, Mountain View, California, CA 94043, USA; 10 Pfizer Ltd, Neuroscience and Pain Research Unit, The Portway Building, Granta Park, Cambridge CB21 6GS, UK

## Abstract

Understanding the interaction between humans and mosquitoes is a critical area of study due to the phenomenal burdens on public health from mosquito-transmitted diseases. In this study, we conducted the first genome-wide association studies (GWAS) of self-reported mosquito bite reaction size (*n* = 84,724), itchiness caused by bites (*n =* 69,057), and perceived attractiveness to mosquitoes (*n =* 16,576). In total, 15 independent significant (*P *<* *5×10^−^^8^) associations were identified. These loci were enriched for immunity-related genes that are involved in multiple cytokine signalling pathways. We also detected suggestive enrichment of these loci in enhancer regions that are active in stimulated T-cells, as well as within loci previously identified as controlling central memory T-cell levels. Egger regression analysis between the traits suggests that perception of itchiness and attractiveness to mosquitoes is driven, at least in part, by the genetic determinants of bite reaction size.

Our findings illustrate the complex genetic and immunological landscapes underpinning human interactions with mosquitoes.

## Introduction

Being bitten by mosquitoes is a common, but generally minor irritation. However, mosquitoes are vectors for many infectious agents that affect humans and have the potential to transmit deadly diseases like malaria, dengue, yellow fever and Zika virus-associated microcephaly (1,2). Understanding the interactions between mosquitoes and humans, including genetic factors, may lead to the development of methods to reduce the spread of mosquito-borne infections and predict risk of infection.

Following a mosquito bite, mosquito salivary antigens elicit a cutaneous hypersensitivity reaction (3), predominated by mast cell degranulation of pruritic mediators, lymphocyte recruitment and localised inflammation (4). Longstanding evidence has suggested a natural history of sensitisation and subsequent desensitisation to mosquito saliva antigen in humans (5). As well as this within-individual variation, levels of mosquito antigen hypersensitivity are known to vary between individuals with bite reaction sizes ranging from mild wheals to large papules. It has been suggested that bite reaction size correlates with the level of pruritus. Previous studies have also shown that mosquitoes prefer to bite some individuals over others who are in close proximity (6). Variation in host attractiveness to mosquitoes is thought to be mediated by body odour composition. Mosquitoes have evolved odorant receptors that are acutely sensitive to compounds unique to human odour (7). Twin studies have suggested that host attractiveness to mosquitoes is a highly heritable trait (8), suggesting that there may be a genetic basis to both hypersensitivity to bites and attractiveness, but no factors have been so far described (8,9).

To identify genetic factors related to mosquito bite traits, we analysed data from three self-reported phenotypes: mosquito bite reaction size (hereon described as ‘bite size’), itchiness caused by mosquito bites, and perceived attractiveness to mosquitoes. We identified a high degree of pleiotropy between these traits, and our GWAS identified a collective total of 15 independent genome-wide significant (GWS) loci, all of which map to immune genes. Further annotation with epigenetic functional data and mining of immune cell phenotyping datasets identified evidence for a prominent role for activated T cells, and overlap with predisposition factors identified for allergic diseases. Using Egger regression, we demonstrate evidence for a causal relationship between bite size and perception of both itch intensity and self-reported attractiveness to mosquitoes.

## Results

### Description of GWAS studies and cohort

We designed three questionnaires to capture variance in mosquito bite size, itch intensity of mosquito bites and perceived attractiveness to mosquitoes (Supplementary Material, Note), and deployed them to research participants and customers of 23andMe Inc., a personal genetics company (10) that had previously developed a range of health and lifestyle surveys. Bite size was captured using one web-based questionnaire on a six point scale (Supplementary Material, Table S1); itch intensity was captured using one web-based questionnaire on a four point scale (Supplementary Material, Table S2); and mosquito attractiveness was captured using one web-based questionnaire (Supplementary Material, Table S3) where participants scored their perceived attractiveness to mosquitoes as being either more or less attractive, compared to other people. Participant responses of ‘I’m not sure’ to any question were removed from phenotypic and genotypic analyses. Table 1 illustrates final participant numbers for each of the three mosquito-related traits, broken down by gender and age.

**Table 1:** Demographic characteristics of the GWAS cohort. Itch intensity numbers reflect exclusion of participants positive for a common immune condition

	Phenotype	Bite size (*n*, % of total)	Itch intensity (*n*, % of total)	Attractiveness (*n*, % of total)
	total	84 724 (100)	69 057 (100)	16 576 (100)
Sex	male	41 355 (48.8)	35 460 (51.3)	8122 (49.0)
	female	43 369 (51.2)	33 597 (48.7)	8454 (51.0)
Age (years)	≤30	9381 (11.1)	8418 (12.2)	2497 (15.1)
	>30 and ≤45	24 578 (29.0)	20 814 (30.1)	5238 (31.6)
	>45 and ≤60	23 721 (28.0)	18 778 (27.2)	4354 (26.3)
	>60	27 044 (31.9)	21 047 (30.5)	4487 (27.0)

Cross-correlation of phenotypic responses using linear regression (Materials and Methods) for bite size and itch intensity indicated a high positive correlation (adjust_*R *= 0.51) after adjusting for age and sex, implying each variable explains approximately 26% (adjust_*R*^2 ^=^ ^0.26) of the variability of the other (Supplementary Material, Table S4 and Fig. S1). Cross-comparison of mosquito bite size and mosquito attractiveness phenotypes (Supplementary Material, Table S5 and Fig. S2) indicated a positive correlation (adjust_*R* = 0.49) after adjusting for age and sex, implying that 24% (adjust_*R*^2 ^=^ ^0.24) of the variance is explained by each other. The cross-comparison between itch intensity and attractiveness phenotypes (Supplementary Material, Table S6 and Fig. S3), showed a high positive correlation (adjust_*R* = 0.63) after adjusting for age and sex, implying that over 39% (adjust_*R*^2 ^=^ ^0.39) of the variance is explained by each other.

**Figure 1:**
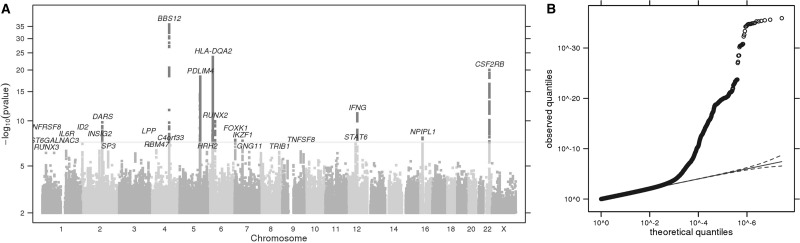
**(A)** Manhattan plot of GWS loci for mosquito bite size. The grey horizontal line corresponds to *P*= 5 × 10^−8^, and results above this threshold are shown in dark grey. Gene labels are annotated as the nearby genes to the significant SNPs. λ = 1.073. **(B)** Quantile-quantile plot. Observed *P* values versus theoretical *P* values under the null hypothesis of no association, plotted on a log scale. The solid black line is shown with a slope of 1, and dashed black lines represent a 95% confidence envelope under the assumption that the test results are independent.

Mosquito bite size data were analysed by linear regression (Materials and Methods) and after applying quality controls we identified 10 GWS (*P *<* *5×10^−^^8^) associations, as illustrated in the Manhattan plot (Fig. 1). Quantile-Quantile plots for this and all other GWAS are provided. Index single nucleotide polymorphisms (SNPs) and nearby genes are listed in Table 2 (regional plots are provided in Supplementary Material, Note, and association test results are provided in Supplementary Material, Data S1). Bite size was scored in a positive direction, meaning the effect size identified for the most significant association (rs377070, *P *=* *1.2×10^−^^36^, effect size -0.07) predicted a 0.07 point reduction in size on a 6-point ordinal scale.

**Table 2:** Index SNPs that are associated with mosquito bite size variation (*P *<* *1.0e-06)

Marker name	Region	Chr	Position	SNP quality	Alleles (A/B)	EAF (B allele)	Effect size for B allele (95% CI)	*P*	Gene context
rs377070	4q27	4	123629002	0.99	C/G	0.388	−0.070 (−0.081, −0.060)	1.2 × 10^−36^	IL21--[]--BBS12
rs3134995	6p21.32	6	32672089	0.99	C/T	0.242	−0.065 (−0.077, −0.052)	1.9 × 10^−24^	HLA-DQB1--[]--HLA-DQA2
rs5750339	22q12.3	22	37319589	0.96	C/G	0.457	−0.052 (−0.062, −0.041)	9.2 × 10^−21^	[CSF2RB]
rs55722650	5q31.1	5	131607300	>0.99	C/T	0.41	−0.049 (−0.060, −0.038)	4.8 × 10^−19^	[PDLIM4]
rs2906856	12q15	12	68390201	0.99	C/T	0.353	0.039 (0.028, 0.050)	7.9 × 10^−12^	DYRK2---[]---IFNG
rs11751172	6p21.1	6	45552731	0.99	C/T	0.244	−0.040 (−0.053, −0.028)	1.1 × 10^−10^	RUNX2--[]---CLIC5
rs6754311	2q21.3	2	136707982	0.97	C/T	0.609	0.039 (0.027, 0.051)	1.6 × 10^−10^	[DARS]
rs143626010	16p11.2	16	28539398	0.91	D/I	0.611	0.033 (0.021, 0.044)	1.5 × 10^−8^	[NPIPL1]
rs7793919	7p22.1	7	4769030	0.94	C/T	0.435	0.031 (0.020, 0.042)	2.8 × 10^−8^	[FOXK1]
rs62447171	7p12.2	7	50309890	>0.99	A/G	0.736	0.034 (0.022, 0.046)	3.5 × 10^−8^	C7orf72---[]--IKZF1
rs3024971	12q13.3	12	57493727	>0.99	G/T	0.896	0.047 (0.030, 0.065)	5.9 × 10^−8^	[STAT6]
rs3102960	2p25.1	2	8452497	0.98	C/T	0.793	−0.036 (−0.049, −0.023)	7.6 × 10^−8^	[]---ID2
rs2230624	1p36.22	1	12175658	0.80	A/G	0.988	0.162 (0.102, 0.222)	1.1 × 10^−7^	[TNFRSF8]
rs9815073	3q28	3	188115682	0.80	A/C	0.657	−0.033 (−0.046, −0.021)	1.1 × 10^−7^	[LPP]
rs112637405	4q28.2	4	130258336	0.63	A/T	0	1.946 (1.228, 2.664)	1.1 × 10^−7^	C4orf33---[]
rs6841258	4p14	4	40565426	0.85	C/T	0.179	0.039 (0.024, 0.054)	3.6 × 10^−7^	[RBM47]
rs78587638	2q14.2	2	119187509	0.98	A/G	0.842	−0.037 (−0.051, −0.023)	4.2 × 10^−7^	INSIG2---[]---EN1
rs12133641	1q21.3	1	154428283	>0.99	A/G	0.395	0.028 (0.017, 0.038)	4.3 × 10^−7^	[IL6R]
rs2075533	9q32	9	117693631	>0.99	A/G	0.572	0.027 (0.017, 0.038)	4.7 × 10^−7^	TNFSF8[]--TNC
rs921533	2q31.1	2	174551931	0.98	A/T	0.658	0.029 (0.018, 0.040)	5.2 × 10^−7^	CDCA7---[]---SP3
rs2963854	5q35.2	5	175083754	0.84	A/G	0.196	−0.034 (−0.047, −0.021)	5.3 × 10^−7^	SFXN1---[]-HRH2
rs112724722	7q21.3	7	93546386	>0.99	A/G	0.998	−0.384 (−0.534, −0.234)	5.3 × 10^−7^	GNGT1-[]-GNG11
rs969584	8q24.13	8	126620429	0.99	C/G	0.351	−0.028 (−0.040, −0.017)	5.4 × 10^−7^	TRIB1---[]---FAM84B
rs71872522	1p31.1	1	77050728	0.73	D/I	1	−1.797 (−2.505, −1.089)	6.6 × 10^−7^	[ST6GALNAC3]
rs200872239	1p36.11	1	25250916	0.77	D/I	0.313	0.032 (0.020, 0.045)	6.8 × 10^−7^	[RUNX3]

Region, cytogenetic band; chr, chromosome; position, build 37 map position of the SNP; SNP quality is average *r*^2^ from imputation; alleles A and B are assigned based on their alphabetical order; EAF (B allele), the frequency of the effect allele, which is denoted here as allele B, across all study participants; effect size, magnitude of effect for the B allele; CI, confidence interval; *P*, λ adjusted significance level; gene context, gene(s) spanning or flanking (<1Mb away from) the index SNP: brackets indicate the position of the SNP, and dashes indicate distance to a flanking gene (−, >1 kb; −, >10kb; − −, >100kb).

**Figure 2:**
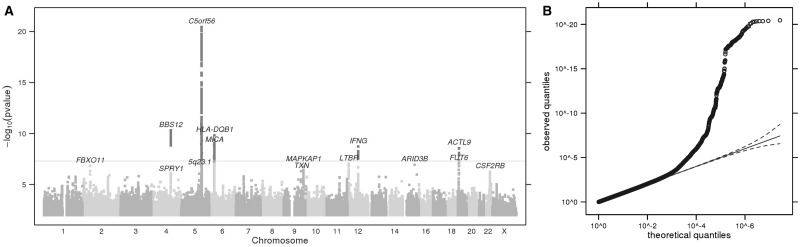
**(A)** Manhattan plot of GWS loci for itch intensity from mosquito bites, adjusted for bite size and excluding responders positive for common immune-related conditions. The grey horizontal line corresponds to *P *=* *5 × 10^−8^, and results above this threshold are shown in dark grey. Gene labels are annotated as the nearby genes to the significant SNPs. λ = 1.042. **(B)** Quantile-quantile plot (insert). Observed *P* values versus theoretical *P* values under the null hypothesis of no association, plotted on a log scale. The solid black line is shown with a slope of 1, and dashed black lines represent a 95% confidence envelope under the assumption that the test results are independent.

Since we observed a high correlation between the bite size and itch intensity traits, to enrich for genetic factors specifically driving the itch response, we conducted a GWAS using linear regression and adjusting for bite size (Materials and Methods). In addition and differing to the analysis of other traits reported, we sought to reduce any genetic contribution from immune-related loci that may play a transitive role in underlying predisposition to immune conditions, hypothesising that this may reveal drivers for the sensory aspect to perception of itch. To address this, we excluded data from 15,667 individuals who self-reported as positive for any one of the 19 autoimmune-related conditions (Supplementary Material, Table S7), reducing the total dataset to 69,057 individuals (Supplementary Material, Table S8). GWAS analysis revealed six GWS associations (*P *<* *5×10^−^^8^) (Fig. 2 and Table 3, Supplementary Material, Note and Data S1). We consider this maximally adjusted itch GWAS as the most representative dataset describing the genetic determinants for itch response to mosquito bites. Regarding the effect magnitude for the itch intensity GWAS, the most significant association at 5q31.1 (rs2248116, *P *=* *3.5×10^−^^21^, effect size; 0.034) corresponds to a 0.034 point reduction in itch on a four point ordinal scale, as this phenotype was scored in a negative direction. Results from the interim itch intensity GWAS without and with bite size adjustment are provided in Supplementary Material, Figures S4 and S5, Tables S9 and S10, respectively.

**Table 3:** Index SNPs that are associated with itch intensity from mosquito bites  (*P *<* *1.0e-06)

Marker name	Region	Chr	Position	SNP quality	Alleles (A/B)	EAF(B allele)	Effect size for B allele (95% CI)	*P*	Gene context
rs2248116	5q31.1	5	131804347	0.98	A/C	0.414	0.034 (0.027, 0.041)	3.5 × 10^−21^	*C5orf56-[]--IRF1*
rs309394	4q27	4	123613670	>0.99	G/T	0.433	0.024 (0.017, 0.031)	4.9 × 10^−11^	*IL21--[]--BBS12*
rs12055445	6p21.32	6	32626739	0.84	A/G	0.513	−0.025 (−0.032, −0.017)	1.5 × 10^−10^	*HLA-DQA1--[]HLA-DQB1*
rs2523614	6p21.33	6	31320654	0.97	C/T	0.689	0.024 (0.016, 0.031)	1.4 × 10^−9^	*HLA-C--[]--MICA*
rs3814244	12q15	12	68413471	>0.99	A/T	0.401	−0.022 (−0.029, −0.015)	1.9 × 10^−9^	*DYRK2---[]---IFNG*
rs4499342	19p13.2	19	8786913	0.98	C/T	0.147	0.029 (0.020, 0.039)	2.7 × 10^−9^	*ADAMTS10---[]--ACTL9*
rs778798	19p13.3	19	5839613	>0.99	A/C	0.74	−0.022 (−0.029, −0.014)	8.6 × 10^−8^	*[FUT6]*
rs5796229	12p13.31	12	6491256	0.75	D/I	0.691	0.023 (0.015, 0.032)	9.1 × 10^−8^	*SCNN1A-[]-LTBR*
rs183794680	15q24.1	15	74807954	0.56	C/T	0.996	0.468 (0.295, 0.640)	1.1 × 10^−7^	*UBL7--[]--ARID3B*
rs72816448	2p16.3	2	48314623	0.81	A/G	0.977	−0.071 (−0.097, −0.044)	1.5 × 10^−7^	*FBXO11---[]---FOXN2*
rs10117812	9q33.3	9	128251025	>0.99	C/G	0.697	0.02 (0.013, 0.028)	1.8 × 10^−7^	*[MAPKAP1]*
rs4978899	9q31.3	9	113032356	0.98	C/T	0.099	0.03 (0.018, 0.041)	4.9 × 10^−7^	*TXN--[]--TXNDC8*
rs5756391	22q12.3	2	37298344	>0.99	A/G	0.621	−0.018 (−0.025, −0.011)	5.5 × 10^−7^	*NCF4--[]--CSF2RB*
rs140260076	5q23.1	5	117114517	0.82	C/T	0.017	−0.073 (−0.102, −0.044)	8.8 × 10^−7^	*[]*
rs55665660	4q28.1	4	124786263	0.99	C/T	0.959	−0.043 (−0.061, −0.026)	9.7 × 10^−7^	*SPRY1---[]---ANKRD50*

The analysis was adjusted for bite size and excluding responders positive for common immune-related conditions). Region, cytogenetic band; chr, chromosome; position, build 37 map position of the SNP; SNP quality is average *r*^2^ from imputation; alleles A and B are assigned based on their alphabetical order; EAF  (B allele), the frequency of the effect allele, which is denoted here as allele B, across all study participants; effect size, magnitude of effect for the B allele; CI, confidence interval; *P*, λ adjusted significance level; gene context, gene(s) spanning or flanking  (<1Mb away from) the index SNP: brackets indicate the position of the SNP, and dashes indicate distance to a flanking gene  (−, >1 kb; −, >10kb; − −, >100kb).

We also carried out ordinal regression analyses on the bite size and itch intensity datasets, using proportional and partial proportional odds models on associations *P *<* *1×10^−^^5^ (Materials and Methods). Compared to the results determined using linear regression, ordinal analysis produced highly concordant *p* values for itch intensity and bite size (Supplementary Material, Fig. S6), demonstrating that both approaches were similarly powered for analysing ordinal traits.

Inspection of participant responses revealed that females (in comparison to males) were more likely to report a severe itch response rather than a mild or non-noticeable itch response, and chose the largest mosquito bite size answer option. Females were also more likely to report being more attractive to mosquitoes than others, compared to males (Pearson’s chi-squared test, *P *<* *2.2×10^−^^16^ for all). We explored this gender bias further in the itch intensity GWAS by conducting two additional linear regression analyses, using data from male (*n =* 41,343, Supplementary Material, Table S11) and female responders (*n =* 43,355, Supplementary Material, Table S12) only. We identified two GWS associations in the male-restricted dataset (Supplementary Material, Fig. S7, Table S13, Data S1), and three GWS associations in the female-restricted dataset (Supplementary Material, Fig. S8, Table S14, Data S1). An association at 6p21.32 was the only locus among the five GWS associations to survive an interaction test (*P = *0.0045, significant against five tests). A comparison of the effect sizes suggested that this locus in the HLA region confers an approximately three-fold greater effect on itch intensity in females compared to males (effect size -0.040 versus -0.017 respectively, Supplementary Material, Table S15).

**Figure 3:**
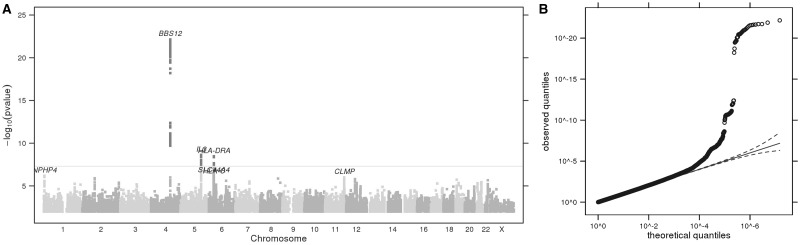
**(A)** Manhattan plot showing GWS loci for perceived attractiveness to mosquitoes. The horizontal grey line corresponds to *P*= 5 × 10^−8^, and results above this threshold are shown in dark grey. Gene labels are annotated as the nearby genes to the significant SNPs. λ = 1.027. **(B)** Quantile-quantile plot (insert). Observed *P* values versus theoretical *P* values under the null hypothesis of no association, plotted on a log scale. The solid black line is shown with a slope of 1, and dashed black lines represent a 95% confidence envelope under the assumption that the test results are independent.

We conducted a GWAS for mosquito attractiveness using logistic regression on 16,576 unrelated European individuals (Supplementary Material, Table S3), grouped into cases (less attractive, *n =* 7,296) or controls (more attractive, *n =* 9,280), and identified three GWS associations (Fig. 3, Table 4, Supplementary Material, Data S1 and Note). Attractiveness was scored in a negative direction; an odds ratio >1 indicates that the effect allele confers a protective effect against being bitten (reduced attractiveness), and an odds ratio <1 indicates an increased perceived risk of being bitten of being bitten (increased attractiveness).

**Table 4:** Index SNPs that are associated with perceived attractiveness to mosquitoes  (*P *<* *1.0e-06)

SNP	Region	Chr	Position	SNP quality	Alleles (A/B)	EAF(B allele)	OR for B allele (95% CI)	*P*	Gene context
rs309403	4q27	4	123616822	0.99	C/T	0.393	1.269 (1.210, 1.330)	6.8 × 10^−23^	*BC045668-[]--CETN4P*
rs1858074	5q31.1	5	131371999	>0.99	A/G	0.317	1.161 (1.105, 1.219)	2.4 × 10^−9^	*ACSL6--[]--IL3*
rs9268659	6p21.32	6	32410941	>0.99	C/T	0.42	1.15 (1.098, 1.205)	3.5 × 10^−9^	*[HLA-DRA]*
rs521977	6p21.33	6	31836827	>0.99	G/T	0.276	1.14 (1.083, 1.200)	5.9 × 10^−7^	*[SLC44A4]*
rs76338894	1p36.31	1	5914245	0.79	G/T	0.949	1.345 (1.195, 1.513)	6.5 × 10^−7^	*AK125078---[]-MIR4689*
rs3132479	6p21.33	6	31274752	0.99	A/G	0.466	0.891 (0.851, 0.933)	8.2 × 10^−7^	*HLA-C--[]--MICA*
rs139253612	11q24.1	11	123114582	0.98	A/G	0.985	1.57 (1.306, 1.887)	9.7 × 10^−7^	*CLMP--[]---MIR4493*

Region, cytogenetic band; chr, chromosome; position, build 37 map position of the SNP; SNP quality is average *r*^2^ from imputation; alleles A and B are assigned based on their alphabetical order; EAF  (B allele), the frequency of the effect allele, which is denoted here as allele B, across all study participants; CI, confidence interval; *P*, λ adjusted significance level; gene context, gene(s) spanning or flanking  (<1Mb away from) the index SNP: brackets indicate the position of the SNP, and dashes indicate distance to a flanking gene  (−, >1 kb; −, >10kb; − −, >100kb).

A comparison of effect size and *P* values between traits for the top index significant SNPs of each trait (mosquito bite size variation, itch intensity from mosquito bites and perceived attractiveness to mosquitoes) is shown in Supplementary Material, Table S29, where the summary statistics for the top hits of each trait are highlighted in gold, and the corresponding summary statistics for the other two traits are shown in adjacent columns as labelled. While it can be seen that many loci are significant across each trait, there are some which are specific to both bite size and itch intensity.

### Heritability and cross-trait correlations

We investigated the heritability of the measured traits by applying LD Score regression (11) on GWAS summary statistics to determine the fraction of heritability (cumulative variance) explained by the GWS loci (*P *<* *5×10^−^^8^) that were identified by each mosquito-trait GWAS (Supplementary Material, Table S16, Materials and Methods).

For mosquito bite size, we calculated the heritability to be 5.7% (*h^2^* =0.0572, 0.01 SE), with GWS bite size loci explaining approximately 13.1% of this variance (or 0.75% of the total variance). For itch intensity, the heritability was 4.3% (*h^2^* =0.0425, 0.01 SE), with GWS itch loci accounting for approximately 10% of this variance (0.43% of the total). For mosquito attractiveness the heritability was 9.1% (*h^2 ^*=^* *^0.091, 0.04 SE), with GWS attractiveness loci accounting for 11.1% of this variance (1.0% of the total) (Materials and Methods).

Cross-trait LD Score correlation analysis (12) identified a positive genotypic correlation between mosquito bite size and itch intensity (*R =* 0.56, 0.14 SE, *P *=* *7.14×10^−^^5^, Supplementary Material, Table S16, Materials and Methods). Genetic determinants for mosquito attractiveness were also positively correlated with mosquito bite size (*R =* 0.97, 0.19 SE, *P *=* *5.18×10^−^^7^) and itch intensity (*R =* 0.94, 0.18 SE, *P *=* *8.16×10^−^^8^).

### MR analysis

**Figure 4:**
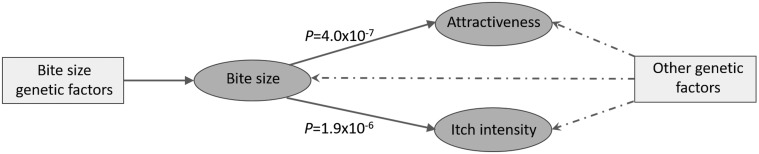
Causal relationships between the three mosquito-related traits. Evidence provided by Egger regression revealed a high degree of overall pleiotropy between all three traits, and also suggested bite size may have a causal relationship on attractiveness to mosquitoes and itch intensity. *P*; slope coefficient significance level.

To find evidence in support of causal relationships between the three traits, we applied a Mendelian Randomisation (MR) approach called MR Egger regression (13) (Supplementary Material, Table S17 and Supplementary Material, Fig. S9, Materials and Methods). Our most significant finding was for the test for bite size affecting attractiveness, where we observed a negative intercept estimate (-0.09, *P *=* *0.0047) and a significant positive slope coefficient (4.33, *P *=* *1.8×10^−^^5^). After constraining at the origin, the magnitude of the slope decreased to 2.28, but the significance level increased to *P *=* *4.0×10^−^^7^. In the test for bite size affecting itch intensity, we also observed a negative but insignificant intercept estimate and a significantly positive slope coefficient (0.43, *P *=* *0.016). After constraining at the origin, the slope estimate again decreased modestly while increasing dramatically in significance (0.34, *P *=* *1.88×10^−^^6^). These results provide evidence of a causal relationship by virtue of a significant slope both with and without constraint through the origin, and by intercepts that are respectively of modest and no significance. While the remaining pairwise tests all failed to demonstrate a significant slope or intercept when not constrained through the origin, each did exhibit at least a modestly significant positive slope when constrained through the origin, suggesting some degree of pleiotropy or causality between each trait (Fig. 4).

### Genetic association analyses

We investigated whether mosquito trait loci also predispose to other traits and/or diseases by annotating our findings with published results from the National Human Genome Research Institute (NHGRI) GWAS Catalogue (14) (Supplementary Material, Tables S18–S22), data from Immunobase (Supplementary Material, Table S23), plus coding variation (Supplementary Material, Table S24), and expression quantitative trait loci (eQTL) (Supplementary Material, Table S25). Additionally, to inform mechanistic hypotheses, we annotated index SNPs in high LD (*r*^2 ^>^ ^0.8) with variants in regulatory regions identified by public databases, namely co-localisation with chromatin states, conservations, and regulatory motif alterations, using the web-based computational tool HaploReg (15,16) (Supplementary Material, Table S26). This information is summarised in Table 5.

**Table 5:** Summary of supporting evidence for GWS mosquito-associated loci

					HaploReg v4.1 annotation					
Region	Mosquito-related trait + SNP	Gene context	Immune disease/ trait	NS SNP	eQTL	Promoter	Enhancer	DNase	Proteins bound	5' or 3'
2q21.3	S: rs6754311	[DARS]		*LCT* N1639S	*MCM6, UBXN4, DARS, CXCR4*		*	*		
4q27	S: rs377070, A: rs309403, I: rs309394	IL21--[]--BBS12			*CETNN4P, BBS12*	*	*	*	*	
5q31.1	I: rs2248116, S: rs55722650	C5orf56-[]--IRF1	***	*SLC22A4* L503F	*PDLIM4, P4HA2, P4HA2-AS1, SLC22A4, SLC22A5, IL5, IRF1, RAD50*	*	*	*	*	*
5q31.1	A: rs1858074	*ACSL6--[]--IL3*	***		*PDLIM4, P4HA2, P4HA2-AS1,SLC22A4, SLC22A5, ACSL6*		*	*	*	
6p21.1	S: rs11751172	RUNX2--[]---CLIC5					*	*		
6p21.32	S: rs3134995	HLA-DQB1--[]--HLA-DQA2	***	*HLA-DQA1* M230V	*HLA-DQA1, HLA-DQB1, HLA-DQB1-AS1, HLA-DRB1, HLA-DRB5, HLA-DRB6, HLA-DQA2, HLA-DQB2*	*	*	*	*	
6p21.32	I: rs12055445	HLA-DQA1--[]HLA-DQB1	***		*HLA-DQA1, HLA-DQB1, HLA-DQB1-AS1, HLA-DRB1, HLA-DRB5, HLA-DRB6, HLA-DQA2, HLA-DQB2, TNXA, HLA-DOB*		*	*	*	
6p21.32	A: rs9268659	*[HLA-DRA]*	***	*HLA-DRA* L242V	*HLA-DQA2, HLA-DQB1, HLA-DQB1-AS1, HLA-DRB6, HLA-DRA*	*	*	*	*	*
6p21.33	I: rs2523614	HLA-C--[]--MICA			*HCG27, HLA-B, PSORS1C1*	*	*	*		
7p12.2	S: rs62447171	C7orf72---[]--IKZF1	***			*	*	*		
7p22.1	S: rs7793919	[FOXK1]			*FOXK1*	*	*	*	*	
12q15	S: rs2906856, I: rs3814244	DYRK2---[]---IFNG			*IFNG-AS1*		*	*		
16p11.2	S: rs143626010	[NPIPL1]			*CDC37P1, EIF3CL, RP11-1348G14.4, RP11-1348G14.6, SH2B1, SULT1A1, SULT1A2, TUFM, NUPR1, CCDC101, IL-27, NPIPB6, EIP3C, EIF3CL, SPNS1*	*	*	*	*	
19p13.2	I: rs4499342	ADAMTS10---[]--ACTL9	***				*	*		
22q12.3	S: rs5750339	[CSF2RB]			*NCF4, CSF2RB*	*	*	*	*	

Cases are highlighted, by endpoint, where an index SNP is within 500 kb and *r*^2^ > 0.5 with a SNP that has been reported to be an eQTL index SNP (i.e. the strongest SNP associated with gene expression for a particular study, tissue and gene) from the literature. Datasets used for the eQTL lookup are contained in Supplementary Material, Table S34. Region, cytogenetic band; trait defined as I: mosquito itch intensity (adjusted for bite size and excluding responders positive for common immune-related conditions), S: mosquito bite size, A: mosquito attractiveness, followed by index association SNP (traits were grouped if index SNPs were in the same chromosomal region and highly correlated at *r*^2^ >0.5), gene context, gene(s) spanning or flanking (<1Mb away from) the index SNP: brackets indicate the position of the SNP, and dashes indicate distance to a flanking gene (−, >1 kb; −, >10kb; − −, >100kb); immune disease/trait, correlation with variant associated with other immune condition (na; not available), where strength of association is: **P <* *5 × 10^−7^, ***P <* *5 × 10^−8^ (Supplementary Material, Tables S18–S22); NS SNP, nonsynonymous SNP, *r^2 ^*>^* *^0.5 with nonsynonymous SNP (Supplementary Material, Table S24); HaploReg annotation summary (*denotes positive for annotation, Supplementary Material, Table S26); eQTL, index SNP association with the expression of the listed gene(s); Promoter, co-localisation with promoter histone marks; Enhancer, co-localisation with enhancer histone marks, DNase, co-localisation with DNase I hypersensitivity marks; Proteins bound, co-localisation with protein binding detected by ChIP-sequencing; 5’ or 3’, index variant(s) in LD (*r*^2^ >0.8) with variants located in a genes 3’ or 5’ UTR region, and dashes indicate distance to a flanking gene  (−, >1 kb; −, >10kb; − −, >100kb).

Many mosquito-related loci have previously been identified as predisposition factors for other immune-mediated traits (Table 5). For example, the itch intensity variant rs2967678 at 19p13.2 (*P*= 2.6 × 10^−^^14^) was previously identified as a risk factor for atopic dermatitis (AD) (17) and another itch intensity variant (rs13079741) located on 3q28 (*P*= 3.2 × 10^−^^8^) is a top locus for celiac disease (18).

We identified significant associations for all three mosquito-related traits at 6p21.32, which harbours the major histocompatibility complex (MHC) locus that encodes the highly polymorphic classical human leukocyte antigen (HLA) class I and class II genes, which are essential for self versus non-self-immune recognition. For mosquito bite size, the index SNP (rs3134995; *P *=* *1.9 × 10^−^^24^) is in LD (*r*^2 ^=^ ^0.51) with the nonsynonymous variant rs9260 (M230V) in *HLA-DQA1*, plus variants that are eQTLs for eight *HLA* genes (Table 5) and the variant rs3129720 previously identified as a risk factor for multiple sclerosis (19), and hypothyroidism (20). We identified two independent GWS associations at 6p21.32 for mosquito itch intensity. The variant rs12055445 (*P *=* *1.5 × 10^−^^10^) correlates with expression of ten *HLA* genes and the other variant (rs2523614, *P *=* *1.4 × 10^−^^9^) was also identified as an eQTL for *HCG27, HLA-B*, and *PSORS1C1* (Table 5)*.* We also identified one association near *HLA-DQB1* that was significantly associated with itch intensity only in female responders. For mosquito attractiveness, the index GWS variant (rs9268659; *P *=* *3.5×10^−^^9^) is in high LD (*r*^2 ^>^ ^0.8) with a *HLA-DRA* variant rs7192 (L242V) and other variants that are eQTLs for five *HLA* genes (Supplementary Material, Table S25). However, each mosquito trait was associated with an independent set (LD *r*^2 ^<^ ^0.1) of HLA variants, reflecting the highly complex patterns of recombination and structural variation at 6p21.32, as well as the gene density (21). Previous studies have identified the MHC region as a prominent susceptibility locus for many immune-related diseases (22).

Two variants spanning a 2.4Mb locus at 5q31.1 were significantly associated with mosquito bite size (rs55722650; *P *=* *4.8×10^−^^19^) and itch intensity (rs2248116; *P*= 3.5 × 10^−^^21^), and are in LD (*r*^2 ^=0.87). Our mosquito attractiveness GWAS also identified a 5q31.1 GWS association that is in partial LD with the bite and itch intensity loci (rs1858074, *P *=* *2.4×10^−^^9^, *r*^2^= 0.32 with rs55722650). The 5q31.1 locus also harbours variants that predispose to autoimmune diseases (23,24) and carnitine metabolite levels (25). The index variants for mosquito bite size and itch intensity are in high LD with rs1050152 (*r*^2 ^=^ ^0.85 for rs55722650 and *r*^2^= 0.84 for rs2248116), a missense variant that encodes the amino acid substitution L503F in the carnitine transporter OCTN1, the protein product of the *SLC22A4* gene. Furthermore, the GWS mosquito-related associations tag variants that are eQTLs for eight genes in the 5q31.1 region (Supplementary Material, Table S25).

All three mosquito-related trait GWASs identified associations at 4q27 in an intergenic region flanked by *IL21* and *BBS12* (bite size: rs377070, *P *=* *1.2 × 10^−^^36^; itch intensity: rs309403, *P *=* *6.8×10^−^^23^; mosquito attractiveness: rs309394, *P *=* *4.9 × 10^−^^11^). These variants are on the same haplotype (for rs377070 and rs309403, *r*^2 ^=^ ^0.93; for rs377070 and rs309394, *r*^2 ^=^ ^0.8), and are eQTLs for *CETNN4P* and *BBS12. BBS12* encodes a membrane trafficking chaperone protein widely expressed in the haematopoietic system, and inherited *BBS12* mutations cause Bardet-Biedl syndrome (26). Genetic variants near *IL21* confer risk of immune diseases (27–29), and allergic sensitisation (29), but are not in LD with our mosquito trait loci.

We observed several independent GWS associations in genomic regions associated with cytokines and their receptors. A GWS association for mosquito bite size was identified at 22q12.3 co-localising with the *CSF2RB* gene that encodes the common β chain receptor component for the IL3, IL5 and CSF2 cytokines (rs5750339, *P*= 9.2 × 10^−^^21^). Sub-significant associations were also detected in the itch intensity and attractiveness GWAS (itch: rs5756391, *P *=* *3.20 × 10^−^^7^; attractiveness: rs5750339, *P*= 8.03×10^−^^4^). The bite size variant is an eQTL for *CSF2RB* in lymphoblastoid cells (Table 5). Mosquito bite size and itch intensity were both significantly correlated with variants located downstream of the *IFNG* locus, encoding interferon-γ (IFN-γ), an important innate immunity cytokine (bite size: rs2906856, *P*= 7.9×10^−^^12^; itch intensity: rs3814244, *P*= 1.9×10^−^^9^, *r*^2 ^=^ ^1). Variants at this locus correlate with reduced expression of *IFNG-AS1*, a non-coding RNA suggested to positively control *IFNG* expression post-transcriptionally (30). Finally, we identified a bite size association located intronic to *DARS* (rs6754311, *P *=* *1.6 × 10^−^^10^), a gene that encodes an aspartyl-tRNA synthetase. Further inspection shows that this index variant co-localises with enhancer histone modifications and is an eQTL for *DARS*, *MCM6, UBXN4*, and *CXCR4* in a range of cell types. *CXCR4* encodes the C-X-C chemokine receptor type 4, a receptor for SDF-1, an important chemotactic factor for lymphocytes. CXCR4 is primarily expressed by naïve and T_H_2 cells, eosinophils and mast cells, where it plays a significant role in T_H_2-type allergic diseases (31).

Four mosquito trait loci were mapped to transcription factor genes all of which have links to immune system function. Mosquito bite size was associated with a region located 5’ of *IKZF1*, which encodes the Ikaros transcription factor (rs62447171, *P *=* *3.5 × 10^−^^8^). Ikaros is upregulated in lymphoid cells and regulates T_H_2 differentiation (32). The index variant co-localises with active histone marks in lymphocytes and is predicted to disrupt a putative binding site for the regulatory transcription factor complex, AP-1. This index SNP is also in high LD with a systemic lupus erythematosus risk factor (33) (rs4917014, *r*^2 ^=^ ^0.699). Bite size was associated with an intronic variant of *FOXK1* (rs7793919, *P *=* *2.8 × 10^−^^8^). This variant co-localises with enhancer histone marks in embryonic and mature cell types, and is an eQTL for *FOXK1* in peripheral blood leukocytes. FOXK1 is a member of the human Forkhead-box family, a group with diverse roles including control of lymphocyte development and regulation (34). A further bite size association was located in a dense haematopoietic enhancer region at 6p21.1, situated 3’ to *RUNX2* (rs62447171, *P *=* *1.1 × 10^−^^10^). RUNX2 is a key transcriptional regulator for T cell development (35). A sub-GWS bite size association was detected at the *STAT6* locus (rs3024971, P= 5.9 × 10^−^^8^). This SNP is an eQTL for the genes encoding STAT6 and STAT2, transcription factors important in the mammalian cytokine response. Descriptions of sub-GWS genetic associations are listed in the Supplementary Material, Note.

### Mosquito-related trait loci are enriched for immune-related enhancers and T lymphocyte loci

To further explore the regulatory nature of the mosquito-related trait associations, we mapped GWS associations (*P *<* *5×10^−^^8^) and their proxies onto sequences annotated with ChromHMM epigenetic markers specifically for active enhancers in published reference tissues and cell types (15,16) (Materials and Methods). For the ‘7_Enh’ annotation across all tissues, the mosquito bite size loci are more enriched for ‘Primary T helper cells PMA-I stimulated’ than any other tissue (10 of 19 lead SNPs) with an uncorrected *P* value of 0.018, but it does not reach significance after correcting for multiple tissues (Fisher’s exact, Supplementary Material, Table S27).

The overlap with loci known to explain variation in levels of T lymphocyte cell subtypes was investigated (36). Although no single immune cell subset obtained significance at a Bonferroni corrected *P* value of 3.5×10^−^^4^, central memory T lymphocytes emerged as a theme among immune cell subsets (*P *<* *0.05) (Supplementary Material, Tables S28 and S30). Five of eight itch subsets, five of 17 subsets for bite size, and two of 12 subsets for attractiveness reflected associations with central memory T cell subsets.

### Pathway analyses

**Figure 5:**
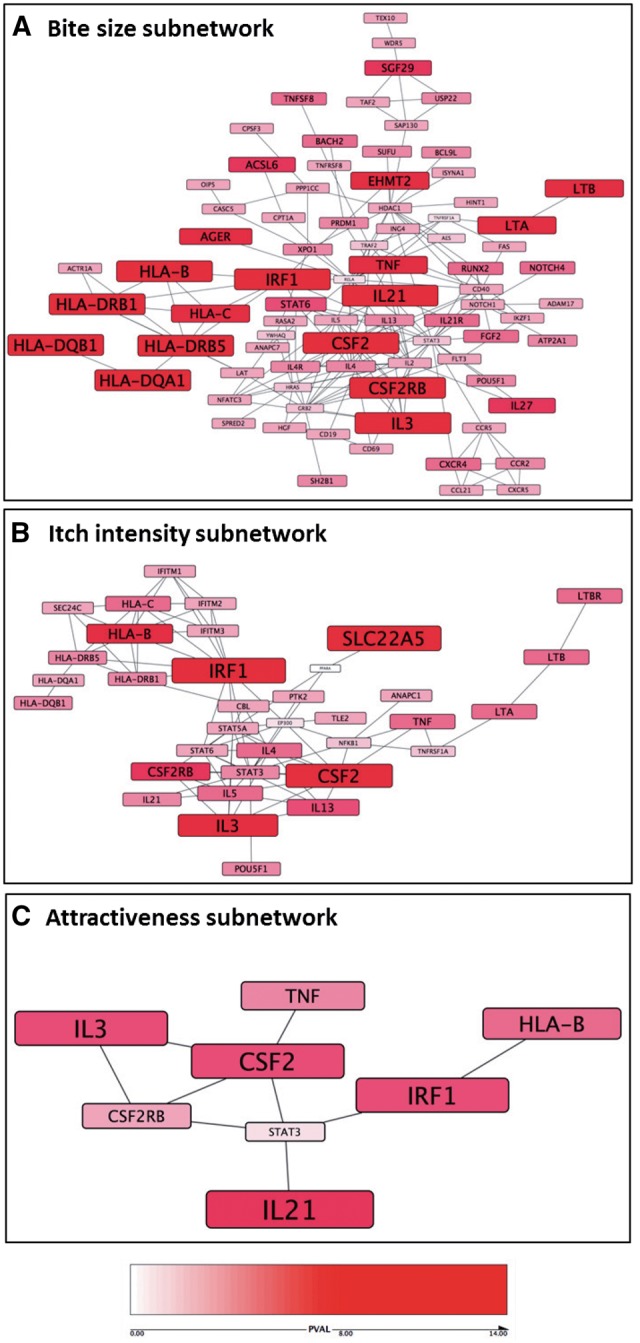
Mosquito-trait pathway analysis. The highest scoring PPI subnetworks based on the PASCAL gene-trait association scores are illustrated for the bite size (**A**), itch intensity (**B**) and attractiveness (**C**) GWAS; nodes are sized and coloured according to *P* value (red = Gene-trait association *P* > 1 × 10^−8^, white = Gene-trait association *P* = 0).

We evaluated whether any biological pathways were enriched in our GWAS results using PASCAL, a tool which integrates signals from multiple SNPs in order to map trait associated genomic regions to genes and annotated pathways (37). First, we applied protein-protein interaction (PPI) network analysis (Materials and Methods) to the PASCAL gene lists in order to find subnetworks of physically and functionally interacting genes with consistently strong associations with our traits. The highest scoring subnetwork for each trait is shown in Figure 5. For the bite size and itch intensity traits, the top scoring subnetworks are significantly larger than observed given a random permutation of the association signals (*P <* 0.05). As we discuss below, a number of cytokine/receptor pairs are identified within the subnetworks, especially the bite size network, which includes CSF2 and CSF2RB, IL21 and IL21R, and IL4 and IL4R.

The top enriched annotated pathways identified by PASCAL include the cytokine receptor interaction pathway from KEGG (χ^2^ FDR = 7.36×10^−^^6^), the BIOCARTA ‘NKT’ pathway which describes the selective expression of chemokine receptors during T-cell polarization (χ^2^ FDR, *P *=* *0.0003) and the REACTOME interleukin receptor SHC signalling pathway (R-HSA-912526, χ^2^ FDR, *P *=* *0.001) (Supplementary Material, Table S31). The BIOCARTA IL3 signalling pathway was significantly enriched in both the itch intensity and attractiveness datasets (both χ^2^ FDR, *P *=* *0.01, Supplementary Material, Tables S32 and S33).

## Discussion

Our analysis shows that mosquito bite size, itch intensity and perceived attractiveness to mosquitoes are all highly correlated traits, at the phenotypic and genotypic level. The primary aim of this GWAS was discovery, and we identified many GWS loci associated with these traits, almost all of which mapped to immune-related genes and pathways, many with well-characterised roles in the acquired immune response to antigenic stimulation. Since this is the first study of this type and scale and there are no comparable studies with sufficient power for replication, we sought to validate our findings by identifying evidence of pleiotropy with other immune phenotypes, annotating loci with epigenetic regulatory information generated in relevant cell types, and identifying biological links with mechanistic pathways, some of which are already linked to mammalian responses to mosquito saliva antigens by *in vitro* and *in vivo* experiments.

The heritability of attractiveness to mosquitoes was previously calculated to be 62% (8). In this previous study, an olfactomer was used to assess female *Aedes aegypti* mosquito preferences for body odour emitted by identical compared to non-identical twin pairs. We used LD Score regression and calculated the heritability for each mosquito-related trait to be below 10%. We estimated that the proportion of variance explained by GWAS factors explains only a fraction of the heritability, suggesting that a large proportion of genetic liability remains undiscovered. LD Score regression relies on the strength of genetic association from all common SNPs (partly determined by sample size), and our estimates are a likely underestimate compared to the *h^2^* parameter predicted by twin studies that consider all genetic variation (38). Despite being derived from a smaller sample size, our analysis suggested that attractiveness was marginally more heritable than bite size or itch intensity. Attractiveness also shared a greater genetic component with bite size and itch intensity, than they did with one another. However, the itch intensity results were adjusted by bite size during analysis, and this will have reduced the shared genotypic component available for capture using cross-trait analysis. A high degree of genetic correlation (12) was detected between the mosquito traits; however, interpretation of this analysis is confounded by the complete overlap in subjects between the bite size analysis and the itch intensity analysis, and also substantial overlap with the attractiveness analysis, together with the fact that these traits likely share variation whether genotypically or environmentally driven. In the latter case, even stochastic false-positive signals may correlate with LD score in each trait, and likewise such a signal would also likely correlate across traits.

Our study also indicated that compared to males, females report greater bite sizes, itchier bites, and greater perceived attractiveness to mosquitoes compared to other individuals. We identified one genetic locus in the HLA region relevant to this difference (rs201452941). This contrasts with previous studies that failed to determine any gender difference in self-reported frequency of bite and symptoms (9) and in experimental models examining mosquito preference for body odours collected from different people (6,39). The itch intensity GWAS was designed with the aim of preferentially identifying non-immune related itch-specific loci, and we identified one such GWS loci (rs4499342, near *ACTL9*) that was not significantly associated (<5×10^−^^8^) with bite size nor attractiveness. In this analysis, we excluded 18.7% participants who had previously reported a common immune condition. This percentage was greater than current estimates of between 7.6-9.4% Europeans recorded as affected by autoimmune disease in hospitalisation registries (40), although this is not directly comparable as the diseases classed as autoimmune differ to our study. Differences may also reflect the phenotyping ascertainment by self-reporting rather than medical diagnosis. In addition, we acknowledge that we have not captured individuals that may develop an immune disease in the future.

Desensitisation to mosquito bites correlates with increasing age, but for the general population this takes many years to occur or may not even happen, as individuals prone to allergic responses naturally avoid getting bitten. The majority of our study participants were older than 30 years (88.9% of the bite size cohort) and could therefore be placed in a sensitizing stage. There are two causal hypotheses we consider when thinking about the interaction of the bite size and itch intensity traits and the perceived attractiveness trait. In the first hypothesis, we suggest that individuals who are predisposed to be more attractive to mosquitoes would be bitten more often, therefore increasing their exposure to mosquito antigens and sensitising the immune system to manifest bigger bite sizes and a more severe itch over time. Alternatively, one might hypothesise that it is simply an individual’s predisposition to increased bite size and itchiness that drives the perception of being bitten, which results in a greater perceived bite frequency and perceived attractiveness to mosquitoes compared to others. To distinguish between these two hypotheses, we applied an MR approach to identify evidence for causality in either direction between the three traits. There was a high degree of pleiotropy between all three traits, but Egger regression suggests that there is strong evidence for causality running in the direction predicted by the second hypothesis of bite size to attractiveness (*P* = 4×10^−^^7^), along with bite size to itch intensity (*P* = 1.88×10^−^^6^) (Fig. 4). Interestingly, in addition to the evidence for causality of bite size on attractiveness, the significantly negative intercept value from Egger regression might be interpreted as negative pleiotropy running counter to the causal relationship (Supplementary Material, Fig. S9A), meaning that genetic factors predisposing to greater bite size would independently reduce attractiveness to mosquitoes. However, a more plausible explanation for this apparent causal relationship is that bite size is driving the *perception* of bite frequency but not *actual* bite frequency. As bite size was measured as a five-point ordinal trait, it is plausible that there would be a minimum threshold of increased bite size necessary to affect the perception of bite frequency. We advise caution in over interpreting this finding and note that the lack of evidence for causality in the reverse direction (of attractiveness on bite size or itch) could be due to the reduced power of the attractiveness dataset due to the smaller sample size.

The control of mammalian body odour production, which may mediate attractiveness to mosquitoes, has been linked to MHC genes (41), which encode cell-surface glycoproteins that present peptides to antigen receptors of T cells, an interaction pivotal for the maintenance of self-tolerance, and protection against pathogens and tumours (42). It is these MHC-derived peptides that undergo metabolism by skin microflora to produce a particular composition of odour (43,44). One study supporting this link demonstrated that carriers of the HLA allele Cw*07 were more attractive to *Anopheles gambiae* mosquitoes compared to carriers of other HLA allelic profiles (45). Unfortunately, we were unable to test for this association due to the density of genotyping and imputing in this region. Further investigation is required to determine whether the mosquito attractiveness genetic factors identified in this study will translate to a measurable difference in mosquito host preference in a controlled environment. Our mosquito trait GWAS results demonstrated significant associations at the MHC region, which has been previously identified as a major risk locus for other immune and allergic sensitivity phenotypes (46,47), suggesting shared genetic aetiology.

There is mounting evidence demonstrating that mosquitoes have evolved to skew host haemostasis and immune responses to create a more favourable environment for pathogen transmission, at least in mouse models (48). Mosquito saliva antigens appear to induce CD4+ helper T cells to differentiate into CD8+ helper T cells, which in turn is thought to facilitate more efficient transmission of a number of arboviruses that would otherwise be neutralised by T_H_1 cytokines (49–51). The suppression of host immune defences brought about by this process is thought to be broadly beneficial to the mosquito population, as there would be a reduction in the host immunity development of salivary antigens such as anti-coagulants, which are essential for blood feeding. In mouse models, exposure to mosquito saliva antigens induces a drop in IFN-γ expression (marker of a T_H_1 phenotype), and stimulates a rise in IL-4 and IL-10 expression (markers of a T_H_2 phenotype) (50–54). Whether a similar immune reaction occurs in humans is not clear; however, the genetic predisposition factors identified by our GWAS implicate many of these players in the human inflammatory response to mosquito antigens.

We detected enrichment of GWS mosquito trait loci mapping to active enhancer regions present in stimulated T cells, and evidence for overlap with loci controlling levels of central memory T cells, a more differentiated T_H_2 cell subset. A top locus identified by the mosquito-trait GWAS was the central memory T cell cytokine IL21 (55) and the PPI network-based analysis (Fig. 5) also implicated the IL21 receptor (IL21R). The association at IL21 is substantiated biologically by previous work demonstrating upregulation of IL21 and its receptor IL21R in skin lesions from psoriasis and atopic dermatitis (AD) patients (56). An IL21 blocking antibody has been shown to reduce epidermal thickness and inflammation in a human psoriasis xenograft SCID model (57).

Pathway analysis identified a number of other network hubs and cytokine/receptor pairs including *IRF1, CSF2, CSF2RB, IL-3*, and *HLA-B*. One such cytokine/receptor pair is IL4 and IL4R. Levels of the T_H_2 cytokine IL-4 are known to be modulated in response to mosquito saliva antigen *in vitro* (49) and pathway analysis on our GWAS results placed *IL-4* and its receptor *IL-4R* central to the bite size network (rs2070874, *P *=* *2.74×10^−^^5^ and rs3024662, *P *=* *4.4×10^−^^4^, respectively). Another pairing identified by mosquito trait GWAS was *CSF2RB*, which encodes the common β chain receptor component for the IL3, IL5 and CSF2 cytokines, the genes of which were all implicated by an independent GWS association at 5q31.1. The bite size subnetwork also highlighted variation mapping to *LTA* and *LTB* (rs9267485, *P *=* *2.4×10^−^^17^), which encode lymphotoxins TNF-β and TNF-C, respectively (58). *TNFA* is another hub central for bite size (rs9267485, *P *=* *2.4×10^−^^17^) and TNF-α has been previously identified as a target for immune modulation by *Aedes aegypti* antigen in rat mast cells (59). Downstream of many of these cytokines and cytokine receptors lie transcriptional regulators such as STAT3. STAT3 was placed central to the itch intensity network (rs9891119, *P *=* *2.55×10^−^^5^) and was recently identified as a critical mediator for chronic itch in mouse models of AD (60).

The hypersensitivity reaction observed in response to mosquito saliva antigens is central to the pathogenesis of common allergic diseases. GWAS for atopy, defined by elevated antigen-specific IgE in blood serum, has identified several susceptibility loci (29,61), many of which overlap with those identified by GWAS for self-reported allergies (47). Topical pruritus is a common complaint for AD and the genetic landscape for this phenotype has been well characterised (17,62). Many of these loci map to proteins implicated in innate immunity and allergic inflammation (63), with some overlap with genetic factors for mosquito-related traits. For both the bite size and itch intensity traits one GWS loci mapped to IFN-γ, and whilst not genetically predisposing to AD, patients with AD treated with topical IFN-γ demonstrated an improvement in pruritus and oedema, supporting IFN-γ as a key mediator of itch response to antigenic stimulation (64).

Web-based surveys capturing phenotypic information on common traits in large recontactable cohorts have proved useful for identifying novel genetic associations by GWAS (10) and are also suitable for replicating genetic associations previously identified in datasets compiled using stricter clinical diagnostic records (65). In this study, we relied on the participants’ assessment of their bite response and perceived mosquito attractiveness, which potentially introduced confounders because the questionnaires used were not standardized or validated. We anticipate some bias of effect estimates to the null due to reporting error and this should be taken into consideration when interpreting effect sizes and odds ratios identified in this study. We encourage others replicate these findings in independent datasets, including those of non-European descent. We also encourage experimental validation, for example through studies examining mosquito preference for a particular genotype in a controlled setting and by exploring whether candidate proteins modulate the immune system in the presence of mosquito saliva antigens.

Our findings provide a further understanding of the complex interaction between humans and mosquitoes, and may provide useful information for identifying individuals at risk for mosquito-borne diseases, monitoring disease-vector populations, and developing novel methods for attracting and repelling mosquito populations.

## Materials and Methods

### Data availability

The full GWAS summary statistics for the 23andMe discovery data set may be requested from 23andMe, Inc. and received subject to the execution of 23andMe's standard data transfer agreement, which includes clauses intended to protect the privacy of 23andMe participants, among other matters. Please contact David Hinds (dhinds@23andme.com) for more information on how to apply for access to the data.

### 23andMe cohort

Participants in the 23andMe dataset were drawn from the customer base of 23andMe, Inc., a personal genetics company. All individuals included in the analyses provided informed consent and answered surveys online according to 23andMe’s human subjects protocol, which was reviewed and approved by Ethical & Independent Review Services, a AAHRPP-accredited institutional review board. The protocol includes methods described in this paper, including online recruitment and data collection, and filtering data for genome-wide analyses. The 23andMe research program, which aims to use genetic and self-reported information to make and support scientific discoveries, is described in the consent document available online (https://www.23andme.com/about/consent/).

Individuals were included in the analysis based on selection for having >97% European ancestry, as determined by analysis of local ancestry via comparison to the CEU, YRI and JPT + CHB HapMap 2 populations (66). A maximal set of unrelated individuals was chosen for each analysis using a segmental identity-by-descent (IBD) estimation algorithm (67). Individuals were defined as related if they shared more than 700cM IBD, including regions where the two individuals share either one or both genomic segments identical-by-descent. This level of relatedness (roughly 20% of the genome) corresponds approximately to the minimal expected sharing between first cousins in an outbred population. DNA extraction and genotyping were performed on saliva samples by the National Genetics Institute (NGI), a CLIA licensed clinical laboratory and a subsidiary of Laboratory Corporation of America. Samples have been genotyped on one of four genotyping platforms. The V1 and V2 platforms were variants of the Illumina HumanHap550+ BeadChip, including about 25,000 custom SNPs selected by 23andMe, with a total of about 560,000 SNPs. The V3 platform was based on the Illumina OmniExpress+ BeadChip, with custom content to improve the overlap with our V2 array, with a total of about 950,000 SNPs. The V4 platform in current use is a fully custom array, including a lower redundancy subset of V2 and V3 SNPs with additional coverage of lower-frequency coding variation, and about 570,000 SNPs. Samples that failed to reach 98.5% call rate were reanalyzed. Individuals whose analyses failed repeatedly were recontacted by 23andMe customer service to provide additional samples.

Participant genotype data were imputed against the March 2012 ‘v3’ release of 1000 Genomes reference haplotypes (68). We phased and imputed data for each genotyping platform separately. First, we used Beagle (69) (version 3.3.1) to phase batches of 8000-9000 individuals across chromosomal segments of no more than 1,000 genotyped SNPs, with overlaps of 200 SNPs. We excluded SNPs with Hardy-Weinberg equilibrium *P* < 10^−^^20^, call rate < 95%, SNPs with minor allele frequency <0.001, or SNPs with large allele frequency discrepancies compared to European 1000 Genomes reference data. The Hardy Weinberg equilibrium test was performed on the full 23andMe cohort (approximately 500,000 Europeans). This threshold was considered conservative and selected as a *P* value of 1.0e-20 with a sample size of 500,000 represents a magnitude of deviation from equilibrium that would give a *P* value of 0.003 with a sample size of 50,000. Frequency discrepancies were identified by computing a 2×2 table of allele counts for European 1000 Genomes samples and 2000 randomly sampled 23andMe customers with European ancestry, and identifying SNPs with a chi squared *P *<* *10^−^^15^. We imputed each phased segment against all-ethnicity 1000 Genomes haplotypes (excluding monomorphic and singleton sites) using Minimac2 (70), using five rounds and 200 states for parameter estimation. For the non-pseudoautosomal region of the X chromosome, males and females were phased together in segments, treating the males as already phased; the pseudoautosomal regions were phased separately. We then imputed males and females together using minimac, as with the autosomes, treating males as homozygous pseudo-diploids for the non-pseudoautosomal region. 23andMe participants were able to fill out web-based questionnaires whenever they logged into their 23andMe accounts.

We performed genome-wide tests for each association relating to the mosquito bite size and itch intensity phenotypes, using linear regression assuming an additive model for allelic effects, adjusting for age, gender, and five principal components of the genotype data matrix. The mosquito bite size phenotype was captured as an ordered variable with assigned numerical values scored as “less_than_pencil” <“ pencil” <“ m_m” <“ dime” <“ quarter” <“more_than_quarter”; 0 < 1<2 < 3<4 < 5, respectively.

The itch intensity phenotype was also captured as ordered variable with assigned numerical values scored as “very_badly” > “badly” > “mildly” > “none”; 0 > 1>2 > 3, respectively.

The mosquito attractiveness phenotype was analysed by logistic regression adjusting for age, gender, and five principal components of the genotype data matrix. The cases were defined as answering “less_than_people_around_me” and controls defined as answering “more_than_people_around_me.” The answer option ‘same as people around me’ was not available.

We applied a genomic control inflation correction of 1.07 to bite size GWAS result, and 1.042 to itch intensity GWAS result (λ = 1.067 and 1.049, to the itch intensity GWASs without removing cases having autoimmune conditions, unadjusted and adjusted by bite size, respectively). We applied a genomic control inflation factor of 1.027 to mosquito attractiveness GWAS.

Following quality controls, a total of 13,520,550 SNPs were available for GWAS for bite size, 13,519,193 SNPs for the representative itch intensity GWAS (with 13,520,550 SNPs both available for the bite size unadjusted and bite size adjusted itch intensity GWASs without removing cases having autoimmune conditions). The itch intensity GWAS performed in males only tested 13,515,082 validated SNPs and in females only tested 13,515,836 validated SNPs. Following quality controls, a total of 7,433,384 SNPs were available for GWAS for attractiveness.

### Cross-correlation analysis on phenotypic responses

For bite size against itch intensity, bite size survey responses were scored in numerical fashion, and cross-compared with that from itch intensity, using linear regression analysis, adjusting for age and gender, with data from 84724 unrelated Europeans who answered both web-based surveys. The partial correlation between bite size and itch intensity, controlling for age and gender was calculated as:

Adjust_*R*^2^ = (*R*^2^ – *R*_(-i)_^2^)/(1-*R*_(-i)_^2^), where *R*^2^ is the coefficient of determination from the linear regression of bite size against itch intensity, age and gender and $R_{\left(-i\right)}^{2}$ is the coefficient of determination from the linear regression of bite size against only age and gender. The partial correlation can be interpreted as the proportion of the remaining unexplained variance that is accounted for by adding "itch intensity" to the existing model.

For bite size against attractiveness, bite size survey responses were scored in numerical fashion, and cross-compared against responses from attractiveness using linear regression analysis, adjusting for age and gender, with data from 7353 unrelated Europeans who answered both web-based surveys. The similar partial correlation between bite size and attractiveness, controlling for age and gender was calculated

For itch intensity against attractiveness, itch intensity survey responses were scored in numerical fashion and cross-compared against responses from attractiveness, using linear regression analysis, adjusting for age and gender, with data from 7353 unrelated Europeans who answered both web-based surveys. The similar partial correlation coefficient between bite size and attractiveness, controlling for age and gender was calculated.

### Ordinal regression GWAS

We applied ordinal models, a proportional odds model and a partial proportional odds model, to the mosquito bite size and itch intensity phenotypes, restricting the analysis to SNPs with *P* < 1×10^−^^5^ in the linear regression GWASs. To apply a proportional odds model a cut-off value was defined and used to create odds ratios that describe the odds of being in equal to or lower than categories than the chosen cut-off versus the odds of being in greater categories. For example, for the itch intensity phenotype, we chose a cut-off value "very_badly", "badly" or "mildly." We applied the proportional odds model to both phenotypes and compared the *P* values calculated in this model versus those calculated in the linear regression model. *P* values from the two models were plotted against one another (Supplementary Material, Fig. S6B), and showed high concordance (*r*^2^ =0.946 for bite size and *r*^2^ 0.994 for itch intensity). For the partial proportional odds models, we relaxed the proportional odds assumption for age, sex and genotypes, and compared the *P* values to those from the linear regression model. Again, the *P* values were highly concordant (*r*^2^ =0.965 for bite size and *r*^2 =^0.973 for itch intensity) (Supplementary Material, Fig. S6C).

### LD score regression

We used the LD score regression method (11,12) to estimate heritability of the three mosquito-related traits as well as to determine the genetic correlation between all pair-wise combinations of traits. The intercepts of the cross-trait analyses were not constrained, as sample overlap was present among all GWAS analyses performed. All other options were left with the default settings, and we utilized the set of SNPs used to calculate the LD Score files (utilized for the regression analyses) to assess strand ambiguity, remove variants that are not SNPs, and remove SNPs with duplicated rs numbers.

The fraction of variance explained by genome-wide significant loci was calculated by adding the *r*^2^ value across independent loci having adjusted *P* < 5.0×10^−^^8^, where *r*^2^ was estimated from each SNP’s z-statistic (beta/SE) via *r*^2^= zstat^2^/(zstat^2^ + N - 2), N being the number of subjects. This was then divided by heritability to get the fraction of heritable variance explained.

### Egger regression

MR Egger regression (13) is a weighted linear regression analysis that estimates a causal relationship between two traits analysed in a pairwise fashion, by taking the most significant summarised genetic association estimates for one trait (the putative causal trait), and regressing these effect estimates against effect estimates for those same variants in some other trait (the putative affected trait). MR Egger regression weights by the inverse variance of the genetic effects on the putative affected trait (by analogy to a meta-analysis), and further it allows for pleiotropy by permitting the regression line to have a non-zero intercept. Under certain assumptions (13), a significantly non-zero intercept can be interpreted as evidence of pleiotropy, while a significant slope can be interpreted as evidence of a causal effect. Note that this represents a parsing of the more traditional measure, in which the regression line is constrained to pass through the origin. Where the slope of the line through the origin is significant (here, using the same inverse-variance weights), this is evidence that there is either pleiotropy or a causal relationship (or any mix thereof), but the distinction between the two is only resolved by removing the constraint on the intercept.

Effect sizes for top GWS associations (<5×10^−^^8^) for mosquito bite size, itch intensity (adjusted for size and excluding individuals with prior common immune condition), and attractiveness were used as instrumental variables in Egger regression, where we used only peaks at each loci (pruning away SNPs within 300Kb of a more significant peak, or with *r*^2 ^>^ ^0.1 from a more significant peak).

In order to match the positive directionality of the bite size GWAS (where a positive effect size denotes greater bite size), we reversed the effect signs for both itch intensity and attractiveness results, so that a positive effect corresponds to increased itch intensity and greater attractiveness.

MR Egger regression further requires that we choose the effect allele, which increases the putative causal trait, by analogy to the reason that a meta-analysis uses the same definition of reference group across studies.

We then executed MR Egger regression for each pair of traits, in each case looking up the effect sizes for the effect alleles (chosen as described above) of the GWS pruned loci from the putative causal trait in the putative affect trait. We then conducted two linear regressions weighted by inverse variance of the effect size on the putative affected trait, as described by Bowden *et al.* (13); one through the origin (which hence does not distinguish between pleiotropy and causality), and one not constrained through the origin (where the intercept is interpreted to represent pleiotropy and the slope to represent causality). Note that aside from the obvious considerations of power, an insignificant slope does not necessarily eliminate the possibility of pleiotropy with inconsistent mutual directions across loci.

### Assessing enrichment for immune-related enhancers

Lead mosquito-related trait SNPs (*P *<* *5×10^−^^8^) were pruned according to a peak height-dependent distance cut off; SNPs were pruned based on LD (*r*^2 ^<^ ^0.1) within a 500Kb window using 1000G human reference genome, where SNPs outside 500Kb from a more significant SNP were automatically considered independent, regardless of LD. Each lead variant was annotated using HaploReg v4.1 (15,16) specifically for ‘7_Enh’ enhancer marks (71) ascribed to any SNP in LD with that lead SNP, using an *r*^2^ of 0.8 according to the European population in 1000 Genomes. Background counts were calculated by applying the same procedure to significant (*P *<* *5×10^−^^8^) GWAS associations from the NHGRI GWAS Catalogue (14). Significance was calculated using Fisher’s exact test, comparing the frequency of each enhancer mark in each trait compared to the frequency for all other GWAS association.

### Enrichment of GWAS loci explaining variation in T lymphocyte cell subtypes

To test for significant overlap of mosquito-related trait loci with loci known to explain variation in levels of T lymphocyte cell subtypes, we used publically available immunophenotyping GWAS data generated from the SardiNIA study from 144 T lymphocyte subsets (36). A Wilcoxon test was applied to test for an enrichment in –log(P) rankings of mosquito trait SNPs with *P* values < 1×10^−^^5^ compared to SNPs genotyped in SardiNIA that did not meet this level of significance for a mosquito trait. A Bonferroni corrected *P* value of *P *<* *3.5×10^−^^4^ was applied to determine significant enrichment.

### Pathway analyses

We applied PASCAL (Pathway scoring algorithm) (37) to the summary statistics from each mosquito trait GWAS with default parameter. PASCAL assigns SNPs to genes if they are located within a 50kb window either side of the gene body; it corrects for LD effects, using 1000 Genomes data. We estimated gene scores using the sum of chi squares method. The gene scores were used for the protein-protein interaction (PPI) analysis (see below). The PASCAL pathway scoring method recalculates scores by aggregating *P* values across pathway genes that are in LD and thus cannot be treated independently. It fuses the genes and calculates a pathway score that takes the full LD structure of the corresponding genes into account. Using pathways from the Reactome (72), Biocarta, and KEGG (73) databases we used PASCAL to compute chi-squared and empirically sampled pathway scores. To find high scoring PPI subnetworks we mapped PASCAL gene scores onto their encoded proteins in the StringDB v10 (74) Human PPI network. We restricted the network to PPIs with >90% confidence and removed nodes with a degree >99th percentile of the degree distribution. Any proteins without a PASCAL score were assigned a score taken from a random uniform distribution between 0 and 1. We then used the BioNet R/Bioconductor package (75) to fit a beta-uniform mixture model to the gene score distribution and BioNet’s fast heuristic approach to find the highest scoring subnetwork. This process was repeated 100 times for each trait and only those nodes appearing in >60% of the highest scoring subnetworks and that are within the largest connected component were retained.

### URLs

1000 Genomes Project, http://www.1000genomes.org/; UCSC Genome Browser, http://genome.ucsc.edu/; HaploReg, http://www.broadinstitute.org/mammals/haploreg/haploreg.php; Immunobase, http://www.immunobase.org/page/Welcome/display; BIOCARTA, http://cgap.nci.nih.gov/Pathways/BioCarta_Pathways

## Supplementary Material

Supplementary Material is available at *HMG* online.

## Supplementary Material

Supplementary Data
