# Supplementary material for: GWAS of self-reported mosquito bite size, itch intensity and attractiveness to mosquitoes implicates immune-related predisposition loci
Source: Hum Mol Genet. 2017 Feb 11;26(7):1391–406. doi: 10.1093/hmg/ddx036 (PMC5390679; doi:10.1093/hmg/ddx036)
Supplement: Supplementary Data [file ddx036_Supp.zip › Supplementary Information_HMG-2016-EZ-00922_Jones.docx]

**GWAS of self-reported mosquito reaction bite size, itch intensity and attractiveness to mosquitoes identifies immune-related predisposition loci**

Amy V. Jones^1^, Mera Tilley^2a^, Alex Gutteridge^3a^, Craig Hyde^4a^, Michael Nagle^5^, Daniel Ziemek^6^, Donal Gorman^7^, Eric Fauman^8^, Xing Chen^4^, Melissa R Miller^5^, Chao Tian^9^, Youna Hu^9^, David A. Hinds^9^, Peter Cox^1b^, Serena Scollen^1b^

**Supplementary Information**

Contains Supplementary Figures 1-9, Supplementary Tables 1-26, 28, and Supplementary Note

**Supplementary Figures**


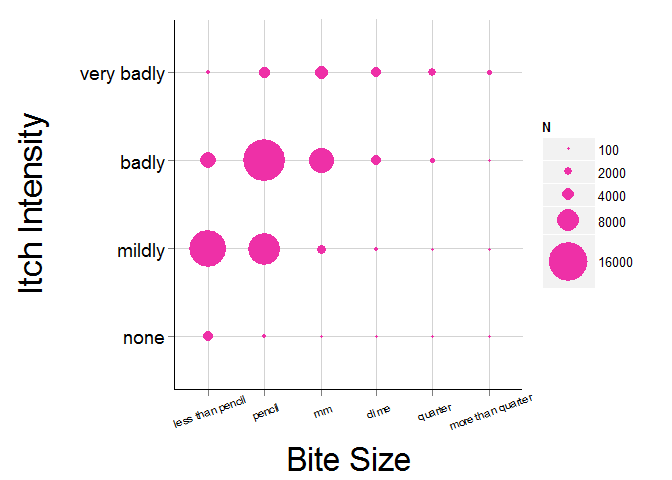


**Figure S1. Dot plot showing correlation between mosquito bite size and itch intensity.** Dot size reflects the relative number of participant responses, as indicated by the key.


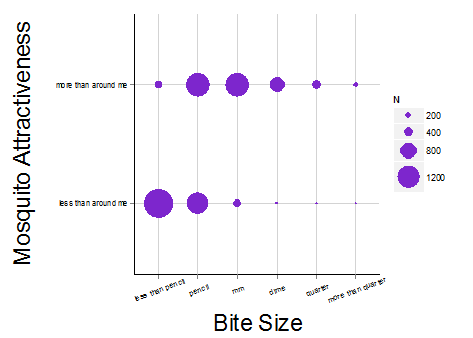


**Figure S2. Dot plot showing correlation between mosquito attractiveness and bite size.** Dot size reflects the relative proportion of participant responses, as indicated by the key.


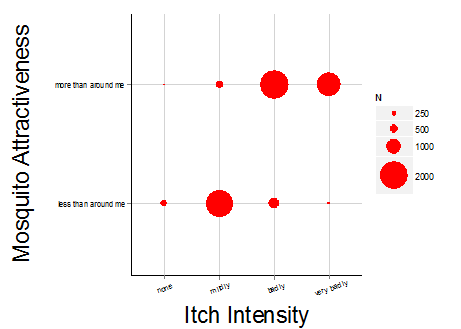


**Figure S3. Dot plot showing correlation between mosquito attractiveness and itch intensity.** Dot size reflects the relative proportion of participant responses, as indicated by the key.


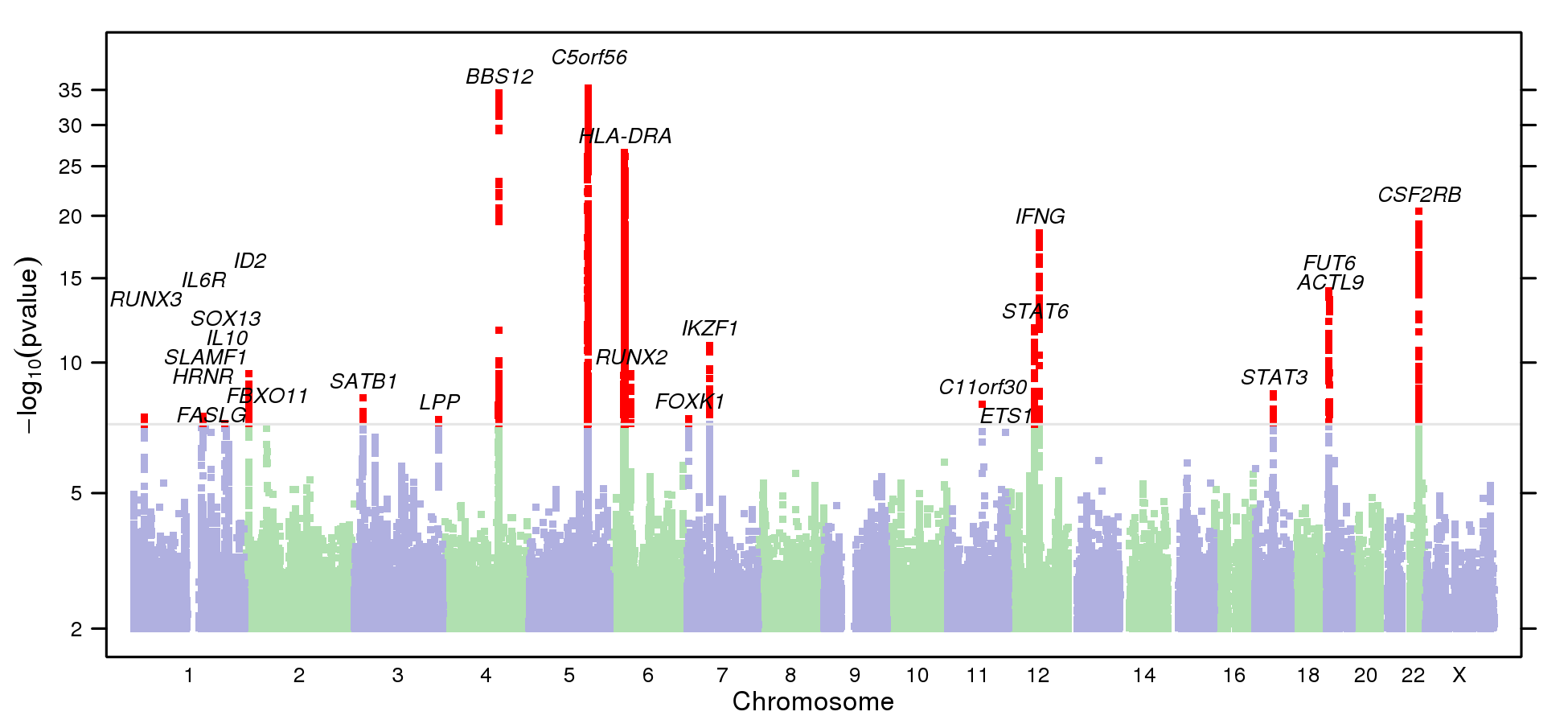


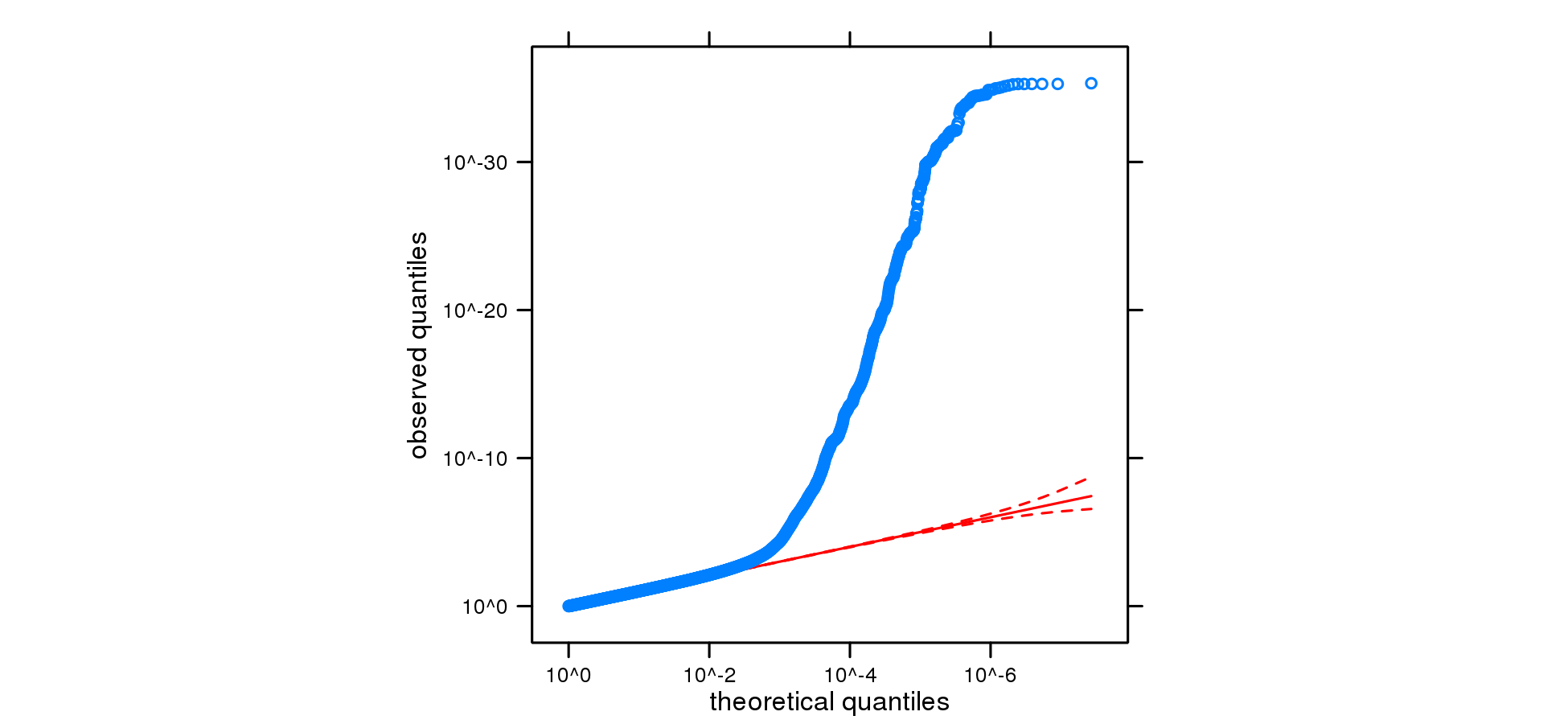


**Figure S4. Manhattan plot of GWS loci for itch intensity from mosquito bites.** The grey line corresponds to *P*=5x10^-8^, and results above this threshold are shown in red. Gene labels are annotated as the nearby genes to the significant SNPs. λ=1.067. **Quantile-quantile plot (insert).** Observed *P* values versus theoretical *P* values under the null hypothesis of no association, plotted on a log scale. The solid red line is shown with a slope of 1, and dashed red lines represent a 95% confidence envelope under the assumption that the test results are independent.


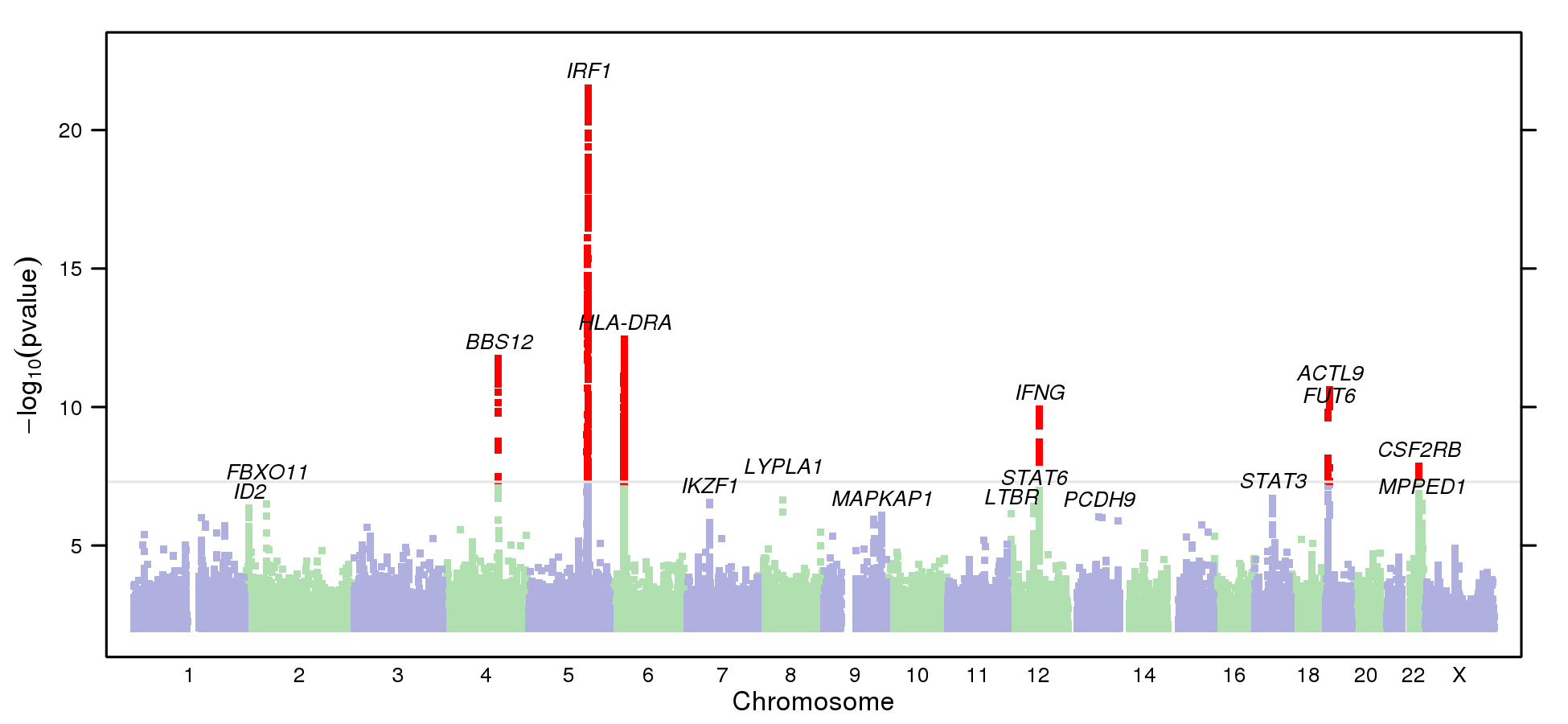


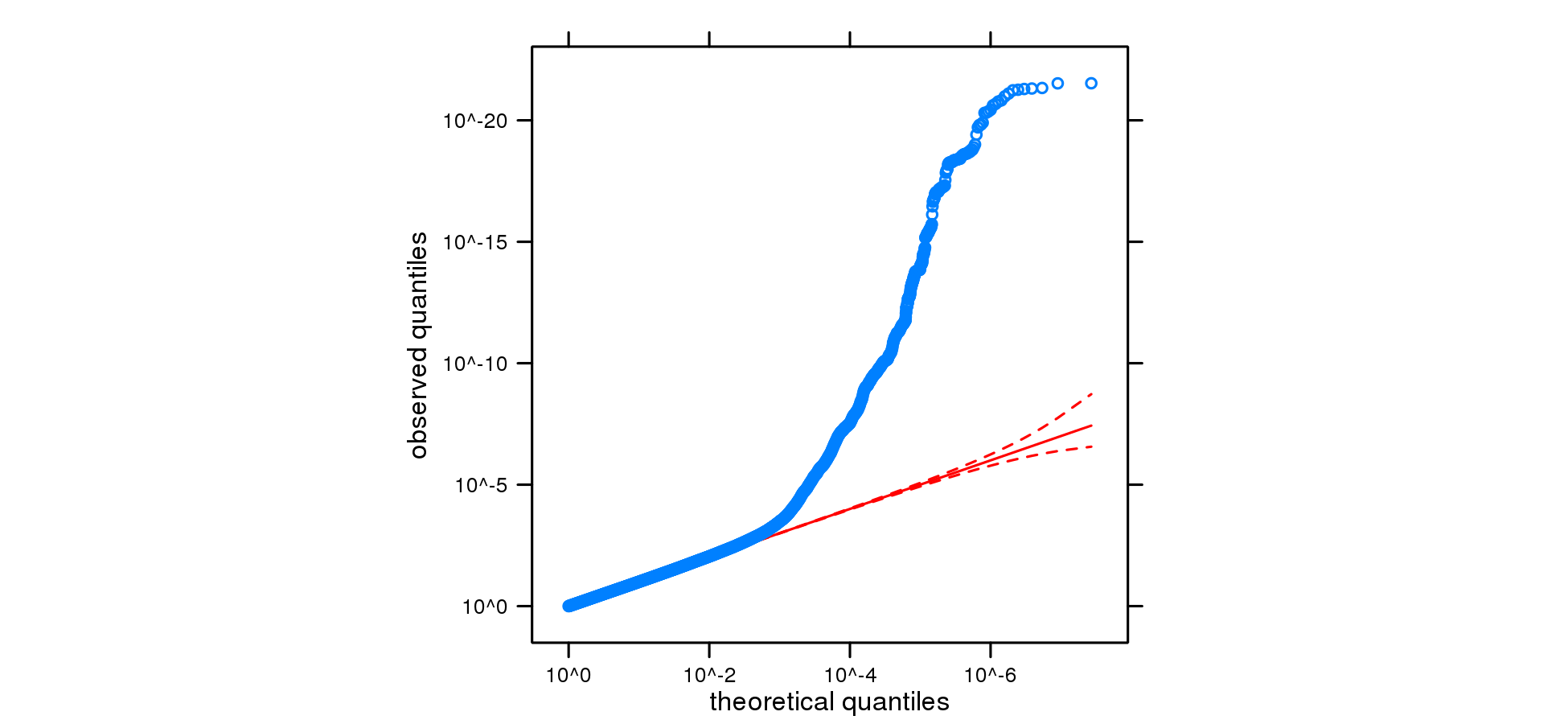


**Figure S5. Manhattan plot of GWS loci for variation in itch intensity from mosquito bites, adjusted by variation in bite size.** The grey line corresponds to *P*=5x10^-8^, and results above this threshold are shown in red. Gene labels are annotated as the nearby genes to the significant SNPs. λ=1.049. **Quantile-quantile plot (insert).** Observed *P* values versus theoretical *P* values under the null hypothesis of no association, plotted on a log scale. The solid red line is shown with a slope of 1, and dashed red lines represent a 95% confidence envelope under the assumption that the test results are independent.


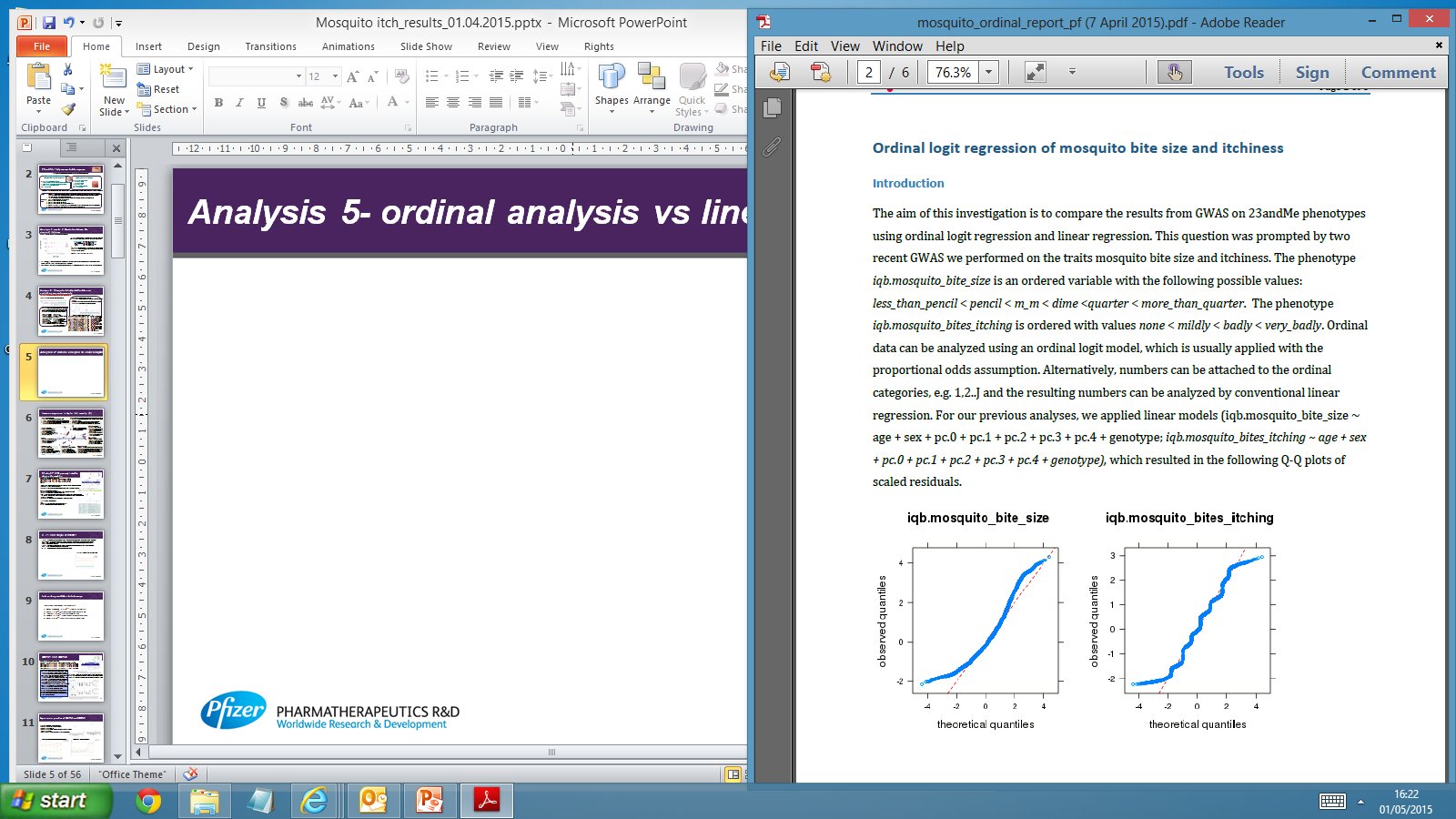

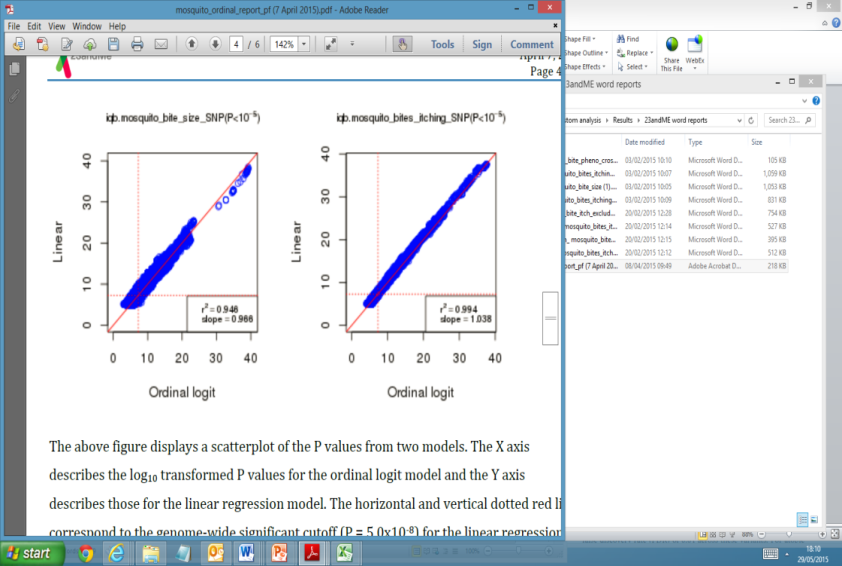

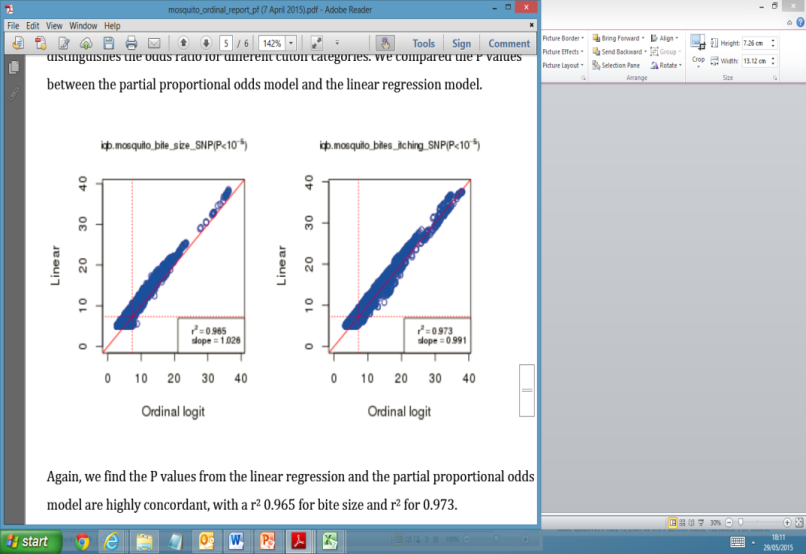


**A**

**B**

**C**

**Figure S6. Comparison of linear and ordinal regression analysis for mosquito bite size and itch intensity.**

(A) Q-Q plots of scaled residuals from linear regression analysis for mosquito bite size and itch intensity phenotypes. Residuals did not scatter evenly along the diagonal line, suggesting non-normal distribution. Scatterplot of *p* values (<1x10^-5^) produced from ordinal proportional model vs linear model (B), and ordinal partial proportional model vs linear model (C) for both mosquito bite size and itch intensity phenotypes. The horizontal and vertical dotted red line correspond to the GWS cut-off (P=5x10^-8^) for the linear regression and ordinal logit models respectively. The red solid line is the diagonal line of slope 1, describing when the two *p* values are equal.


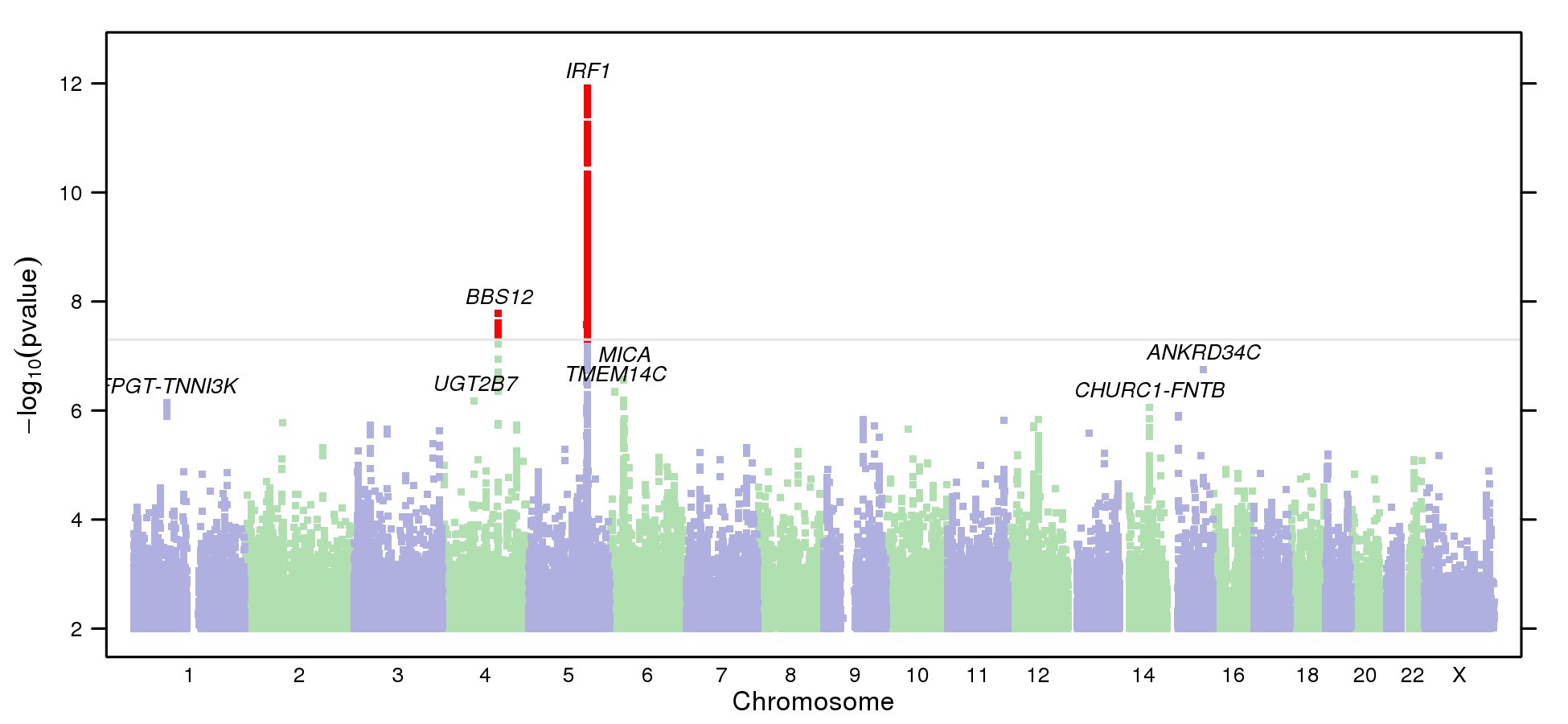


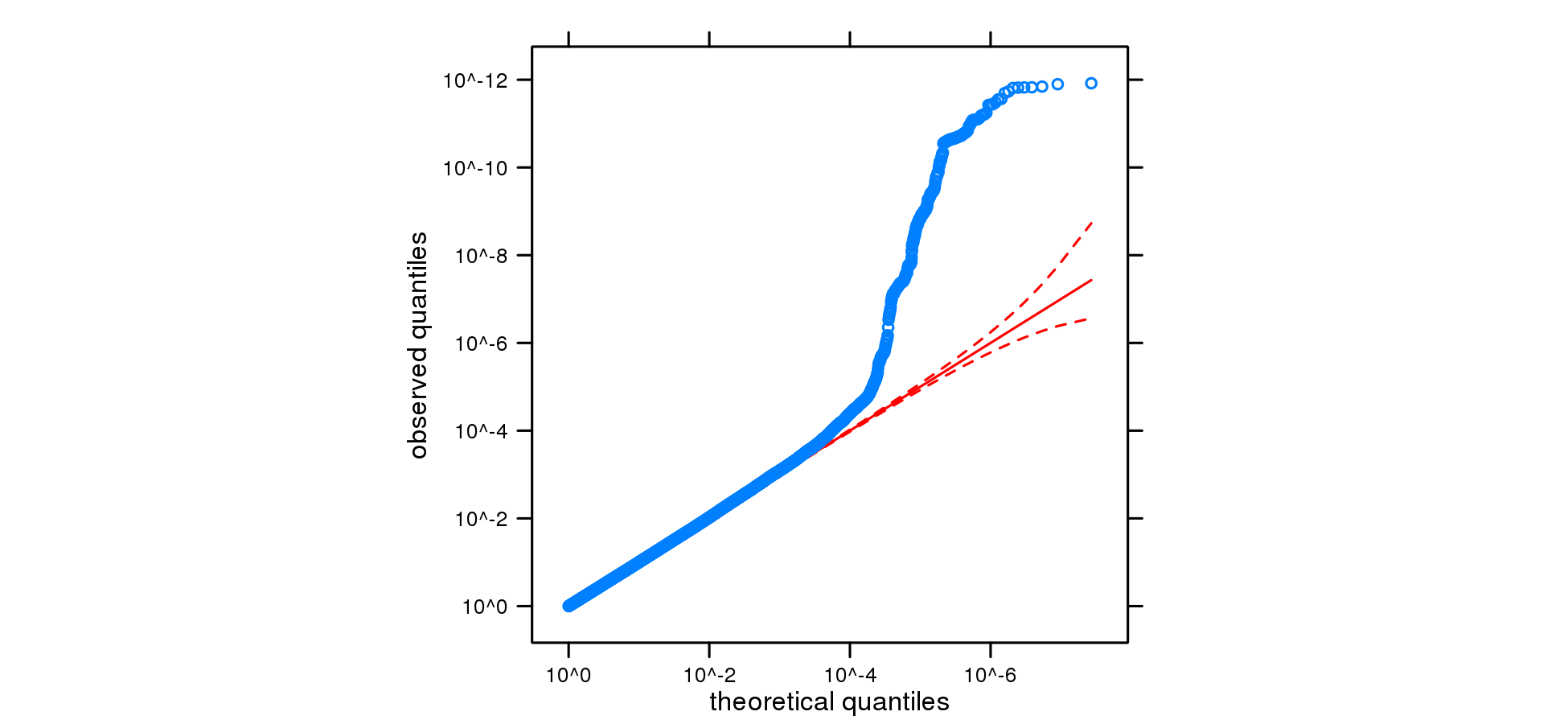


**Figure S7. Manhattan plot showing GWS loci for variation in itch intensity from mosquito bites (adjusted for bite size), analysis restricted to unrelated male Europeans from 23andMe.** Gene labels are annotated as the nearby genes to the significant SNPs. λ=1.025. **Quantile-quantile plot (insert).** Observed *P* values versus theoretical *P* values under the null hypothesis of no association, plotted on a log scale. The solid red line is shown with a slope of 1, and dashed red lines represent a 95% confidence envelope under the assumption that the test results are independent.


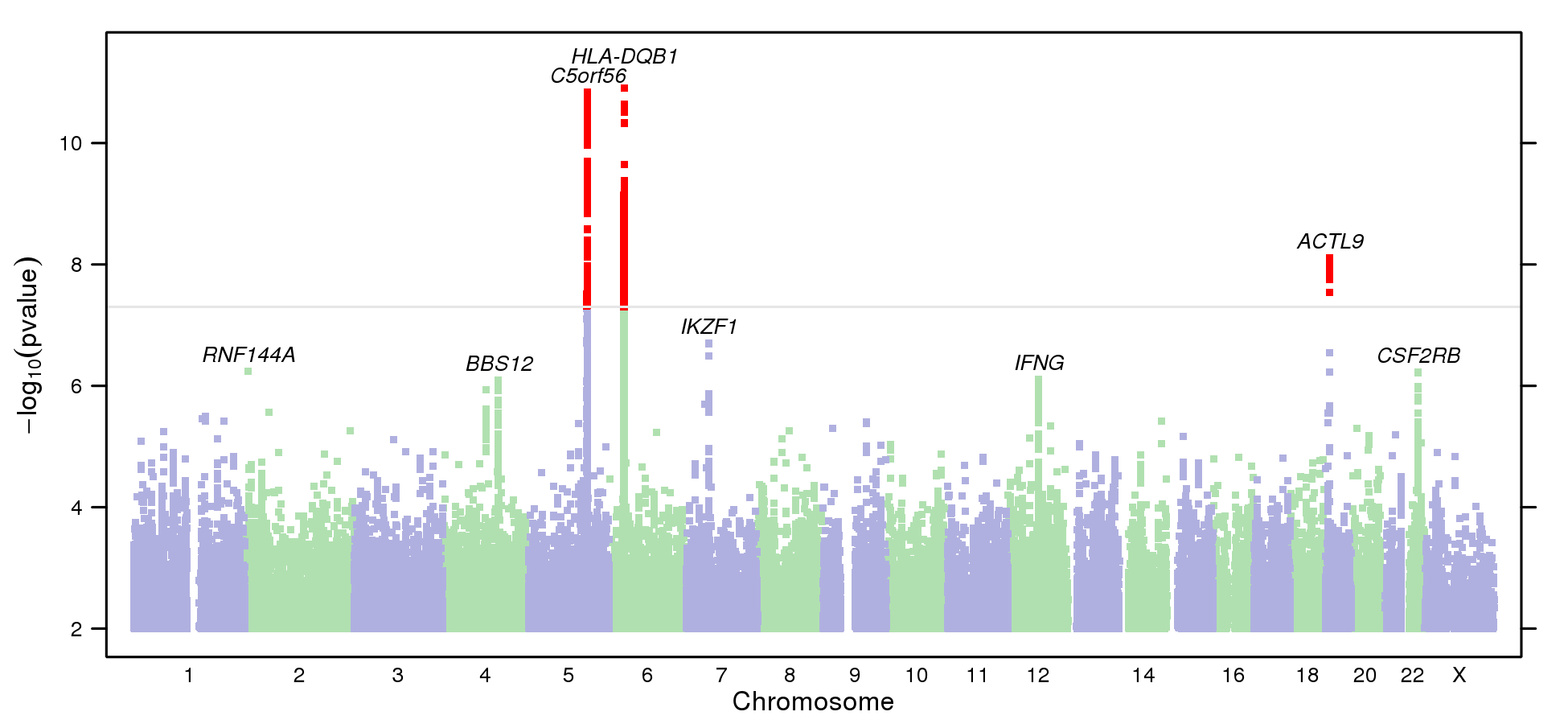


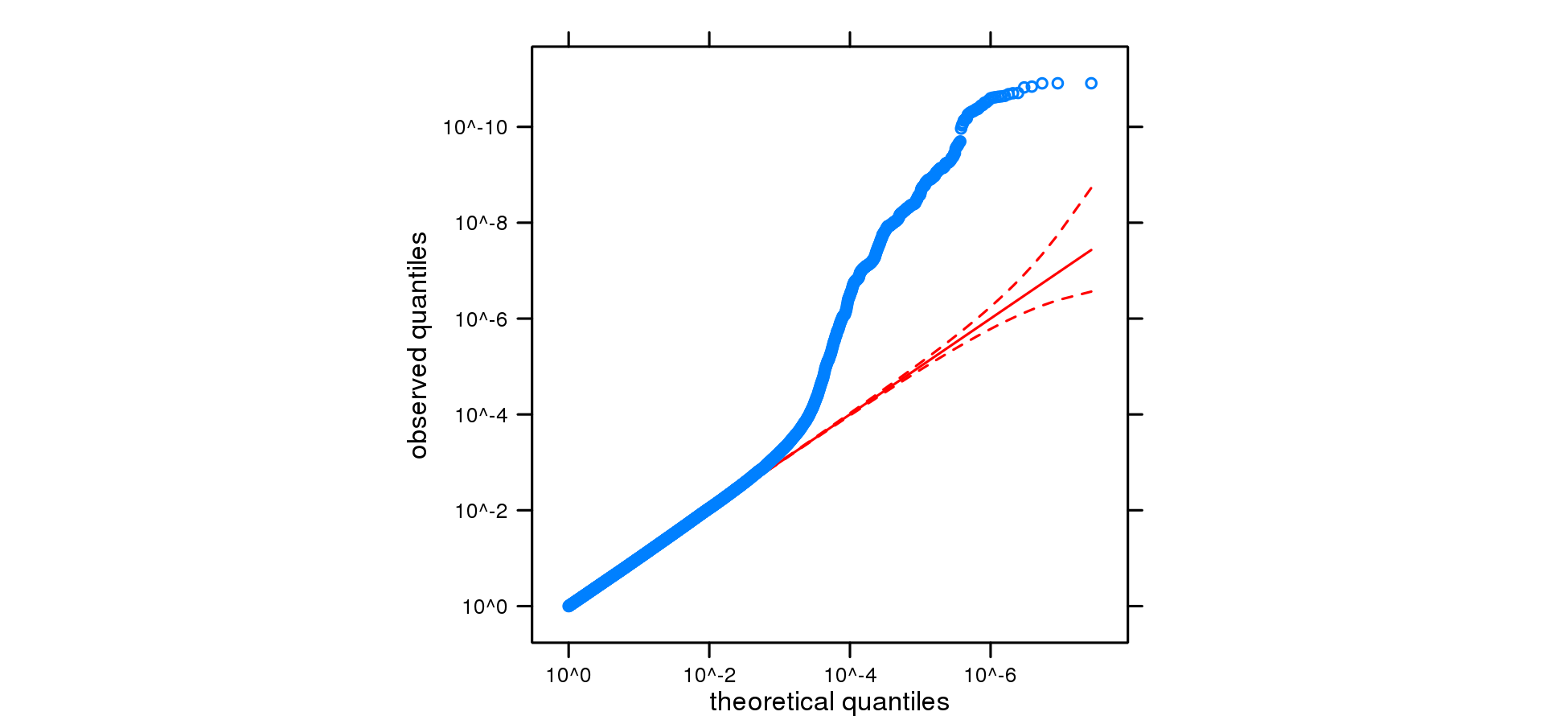


**Figure S8. Manhattan plot showing GWS loci for variation in itch intensity from mosquito bites (adjusted for bite size), analysis restricted to unrelated female Europeans from 23andMe.** Gene labels are annotated as the nearby genes to the significant SNPs. λ=1.022. **Quantile-quantile plot (insert).** Observed *P* values versus theoretical *P* values under the null hypothesis of no association, plotted on a log scale. The solid red line is shown with a slope of 1, and dashed red lines represent a 95% confidence envelope under the assumption that the test results are independent.

**Figure S9. Egger regression plots for pairwise comparison between mosquito-related traits.** The MR Egger regression estimate (red line), and the origin-constrained MR Egger regression estimate (purple line) are illustrated, accompanied by statistics describing the slope coefficients and *p* values.

1. **MR Egger plot of mosquito bite size on attractiveness**


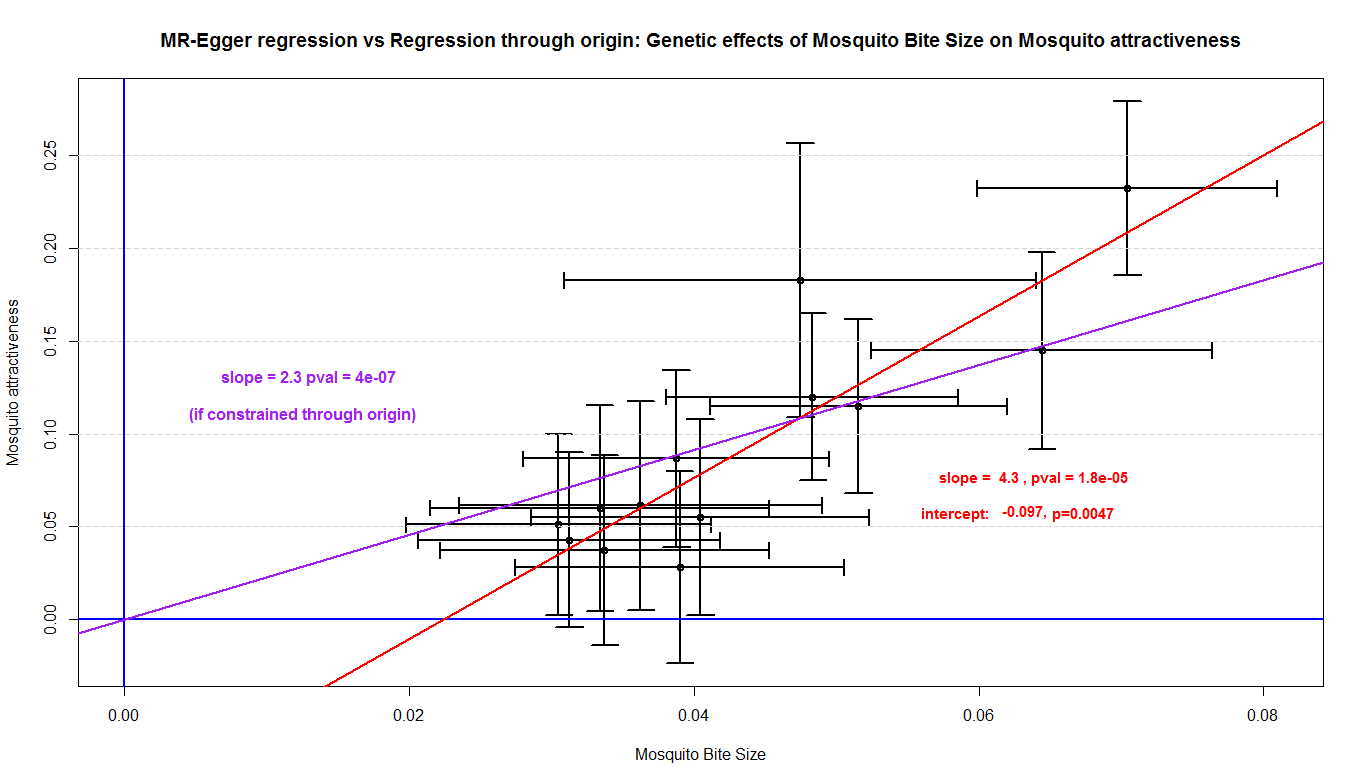


1. **MR Egger plot of mosquito bite size on itch intensity**


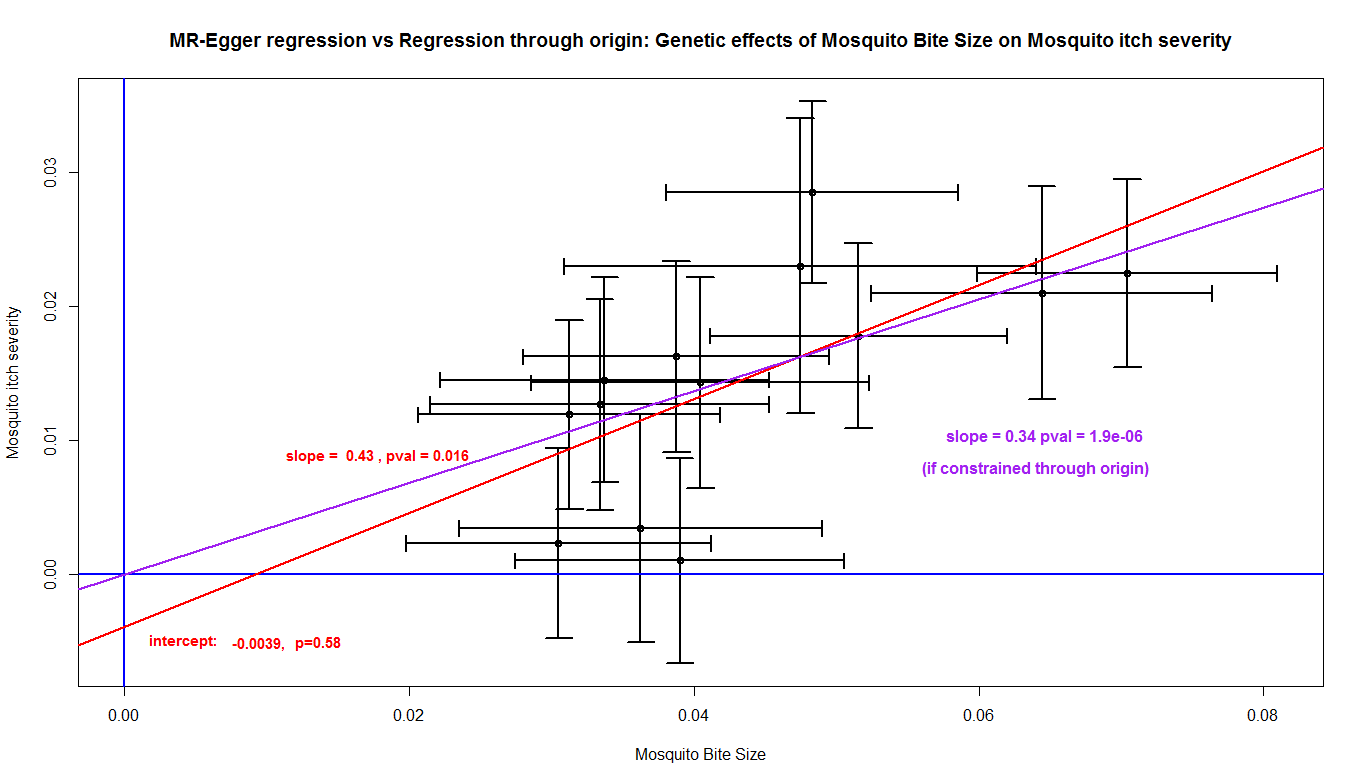


1. **MR Egger plot of mosquito itch intensity on bite size**


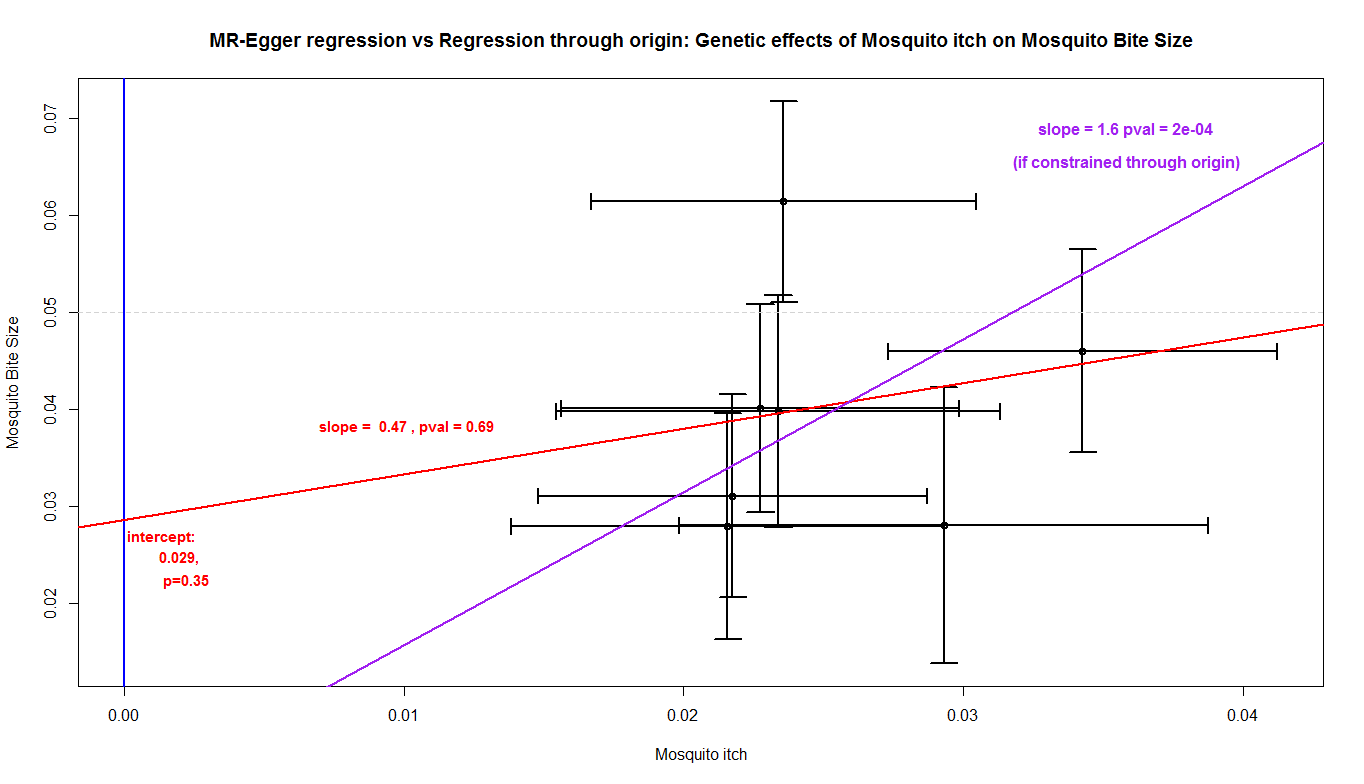


1. **MR Egger plot of mosquito itch intensity on attractiveness**


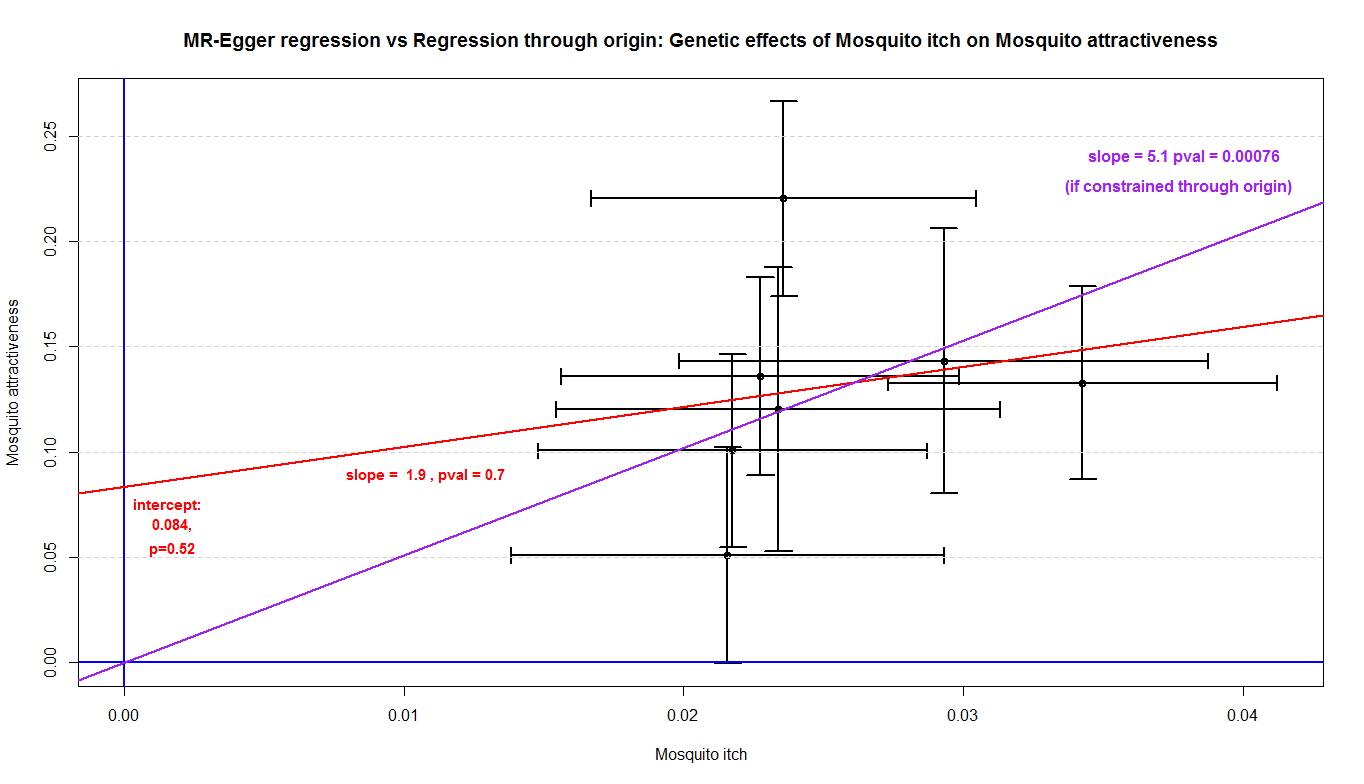


1. **MR Egger plot of attractiveness on bite size**


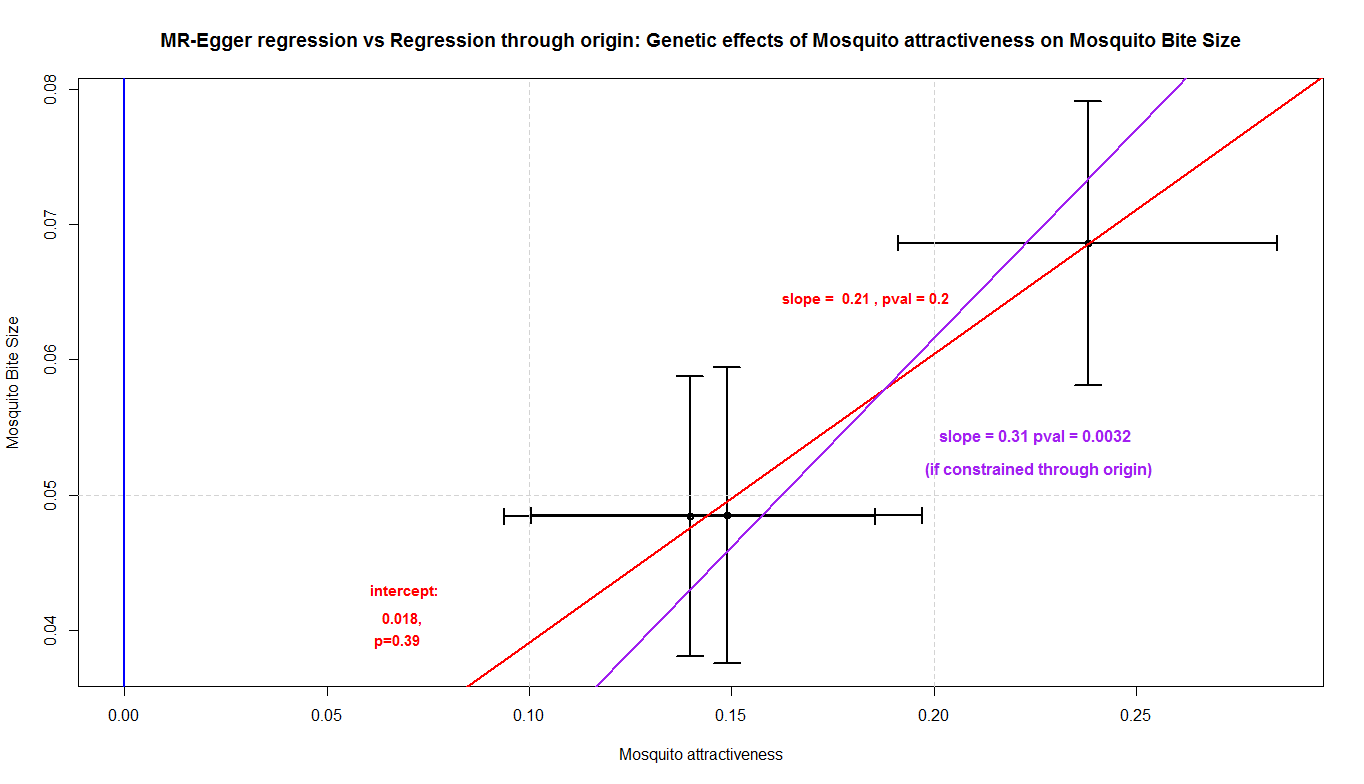


1. **MR Egger plot of attractiveness on itch intensity**


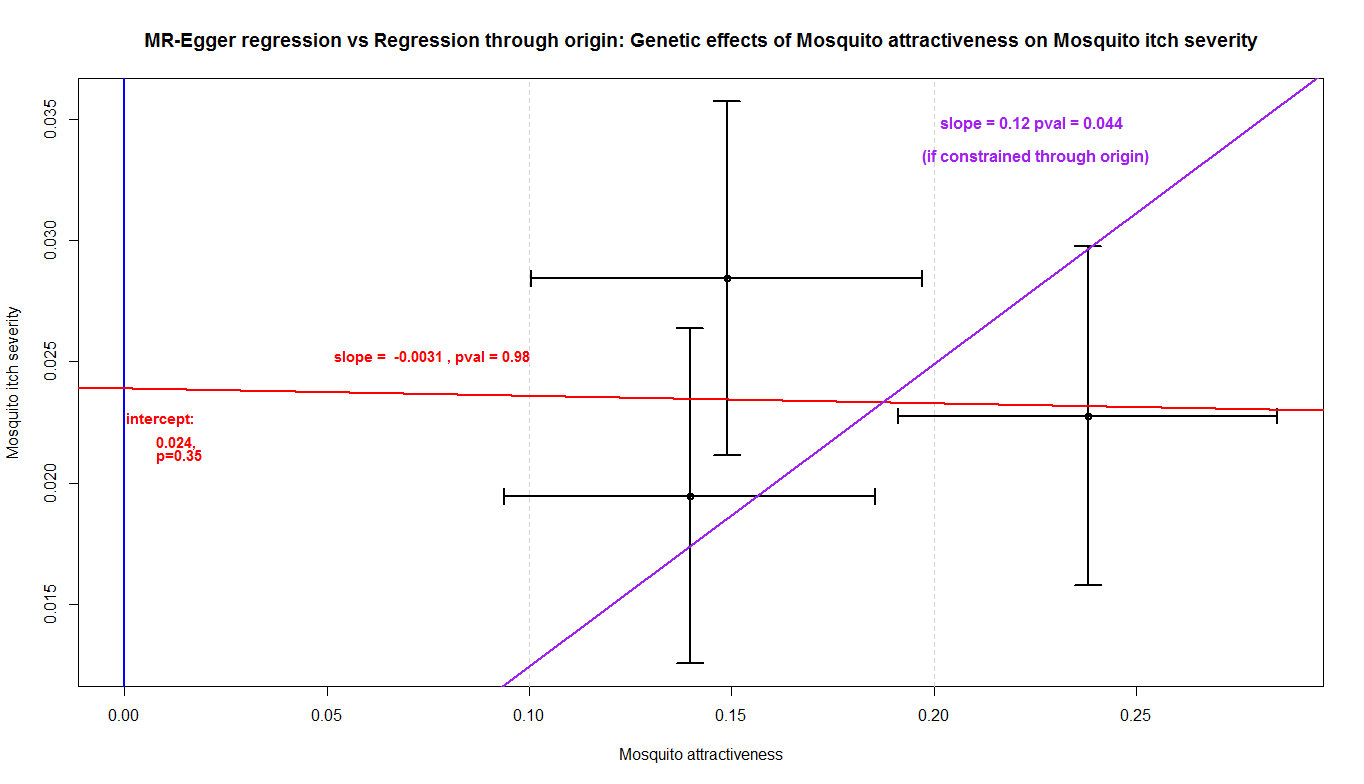


**Supplementary Tables**

|  |  |  |  | Sex | | Age (years) | | | |
| --- | --- | --- | --- | --- | --- | --- | --- | --- | --- |
| Phenotype | Question | Response | Total, n (%) | Male, n (%) | Female, n (%) | ≤30, n (%) | >30 and ≤45, n (%) | >45 and ≤60, n (%) | >60, n (%) |
| Mosquito bite size | When you get bitten by a mosquito, how big do the bites usually get? | Much less than 1/4 inch (much less than the width of a pencil) | 24562 (29.0) | 14843 (35.9) | 9719 (22.4) | 1595 (17.0) | 5355 (21.8) | 7305 (30.8) | 10307 (38.1) |
|  |  | Around 1/4 inch (about the width of a pencil) | 33356 (39.4) | 17734 (42.9) | 15622 (36.0) | 3644 (38.8) | 9962 (40.5) | 9405 (39.6) | 10345 (38.3) |
|  |  | Around 1/2 inch (about the width of a plain M&M) | 16379 (19.3) | 6224 (15.1) | 10155 (23.4) | 2306 (24.6) | 5455 (22.2) | 4433 (18.7) | 4185 (15.5) |
|  |  | Around 3/4 inch (about the width of an American dime) | 6113 (7.2) | 1753 (4.2) | 4360 (10.1) | 1040 (11.1) | 2196 (8.9) | 1513 (6.4) | 1364 (5.0) |
|  |  | Around 1 inch (about the width of an American quarter) | 3273 (3.9) | 671 (1.6) | 2602 (6.0) | 608 (6.5) | 1255 (5.1) | 773 (3.3) | 637 (2.4) |
|  |  | Much more than an inch (much more than the width of an American quarter) | 1041 (1.2) | 130 (0.3) | 911 (2.1) | 188 (2.0) | 355 (1.4) | 292 (1.2) | 206 (0.8) |
|  |  | total | 84724 | 41355 | 43369 | 9381 | 24578 | 23721 | 27044 |

**Table S1. Mosquito bite size phenotype research participant demographics.** Phenotype, mosquito-related trait; question, survey question; response, answer options; total, n (%), sum of total unrelated

d European responders for that answer option and percentage; sex, total responder number for a given answer option broken down into males or females; age, responders broken down into age ranges.

|  |  |  |  | Sex | | Age (years) | | | |
| --- | --- | --- | --- | --- | --- | --- | --- | --- | --- |
| Phenotypes | Question | Response | Total, n (%) | Male, n (%) | Female, n (%) | ≤30, n (%) | >30 and ≤45, n (%) | >45 and ≤60, n (%) | >60, n (%) |
| Mosquito itch, and mosquito itch adjusted by bite size | "When you are bitten by mosquitos, how much do the bites typically itch?" | Very badly (impossible to ignore) | 14159 (16.7) | 3277 (7.9) | 10882 (25.1) | 1580 (16.9) | 4214 (17.1) | 4169 (17.6) | 4196 (15.5) |
|  |  | Somewhat badly (definitely noticeable, at times hard to ignore) | 36012 (42.5) | 16435 (39.7) | 19577 (45.1) | 4604 (49.1) | 11183 (45.5) | 9637 (40.6) | 10588 (39.2) |
|  |  | Only mildly (noticeably itching, but easy to ignore) | 31207 (36.9) | 19505 (47.2) | 11702 (27.0) | 2847 (30.3) | 8246 (33.6) | 8912 (37.6) | 11202 (41.4) |
|  |  | Not at all (no noticeable itching) | 3346 (3.9) | 2138 (5.2) | 1208 (2.8) | 350 (3.7) | 935 (3.8) | 1003 (4.2) | 1058 (3.9) |
|  |  | total | 84724 | 41355 | 43369 | 9381 | 24578 | 23721 | 27044 |

**Table S2. Mosquito bite itch intensity research participant demographics.** Phenotype, mosquito-related trait; question, survey question; response, answer options; total, n (%), sum of total unrelated European responders for that answer option and percentage; sex, total responder number for a given answer option broken down into males or females; age, responders broken down into age ranges.

|  |  |  |  | Sex | | Age (years) | | | |
| --- | --- | --- | --- | --- | --- | --- | --- | --- | --- |
| Phenotype | Question | Response | Total, n (%) | Male, n (%) | Female, n (%) | ≤30, n (%) | >30 and ≤45, n (%) | >45 and ≤60, n (%) | >60, n (%) |
| Mosquito attractiveness | When you are exposed to mosquitoes, how much do you get bitten? | Less than the people around me | 7296 (40.0) | 4434 (54.5) | 2862 (33.8) | 979 (39.2) | 2052 (39.2) | 1965 (45.1) | 2300 (51.3) |
|  |  | More than the people around me | 9280 (60.0) | 3688 (54.5) | 5592 (66.1) | 1518 (60.8) | 3186 (60.8) | 2389 (44.1) | 2187 (48.7) |
|  |  | total | 16576 | 8122 | 8454 | 2497 | 5238 | 4354 | 4487 |

**Table S3. Mosquito attractiveness research participant demographics.** Phenotype, mosquito-related trait; question, survey question; response, answer options; total, n (%), sum of total unrelated European responders for that answer option and percentage; sex, total responder number for a given answer option broken down into males or females; age, responders broken down into age ranges.

|  |  |  | Mosquito bite size, n | | | | | |
| --- | --- | --- | --- | --- | --- | --- | --- | --- |
|  |  |  | Much less than 1/4 inch (much less than the width of a pencil) | Around 1/4 inch (about the width of a pencil | Around 1/2 inch (about the width of a plain M&M) | Around 3/4 inch (about the width of an American dime) | Around 1 inch (about the width of an American quarter) | Much more than an inch (much more than the width of an American quarter) |
|  |  | Scoring direction | 1 | 2 | 3 | 4 | 5 | 6 |
| Mosquito itch intensity, n | Very badly (impossible to ignore) | 1 | 683 | 3312 | 4339 | 2854 | 2139 | 832 |
|  | Somewhat badly (definitely noticeable, at times hard to ignore) | 2 | 5811 | 16906 | 9269 | 2829 | 1019 | 178 |
|  | Only mildly (noticeably itching, but easy to ignore) | 3 | 15108 | 12822 | 2721 | 422 | 110 | 24 |
|  | Not at all (no noticeable itching) | 4 | 2960 | 316 | 50 | 8 | 5 | 7 |

**Table S4. Cross-tabulation of research participant responses for mosquito bite size against itch intensity.** Numbers (n) of responses from unrelated European research participants who answered questions for mosquito itch intensity and mosquito bite size; scoring direction, for the phenotypic correlation analysis mosquito bite size was scored in a positive direction with increasing bite sizes, whereas itch intensity was scored in a negative direction, with decreasing itch intensity.

|  |  |  | Mosquito bite size (n) | | | | | |
| --- | --- | --- | --- | --- | --- | --- | --- | --- |
|  |  |  | Much less than 1/4 inch (much less than the width of a pencil) | Around 1/4 inch (about the width of a pencil) | Around 1/2 inch (about the width of a plain M&M) | Around 3/4 inch (about the width of an American dime) | Around 1 inch (about the width of an American quarter) | Much more than an inch (much more than the width of an American quarter) |
|  |  | Scoring direction | 1 | 2 | 3 | 4 | 5 | 6 |
| Mosquito attractiveness (n) | More than the people around me (controls) | 1 | 326 | 1287 | 1275 | 717 | 424 | 163 |
|  | Less than the people around me (cases) | 2 | 1569 | 1160 | 340 | 62 | 23 | 7 |

**Table S5. Cross-tabulation of research participant responses for bite size against mosquito attractiveness.** Numbers (n) of responses from unrelated European research participants who answered questions for mosquito attractiveness and mosquito bite size; scoring direction, for the phenotypic correlation analysis with mosquito attractiveness scored in a negative direction, whereas mosquito bite size was scored in a positive direction.

|  |  |  | Mosquito itch intensity, n | | | |
| --- | --- | --- | --- | --- | --- | --- |
|  |  |  | Very badly (impossible to ignore) | Somewhat badly (definitely noticeable, at times hard to ignore) | Only mildly (noticeably itching, but easy to ignore) | Not at all (no noticeable itching) |
|  |  | Scoring direction | 1 | 2 | 3 | 4 |
| Mosquito attractiveness (n) | More than the people around me (controls) | 1 | 1717 | 2033 | 422 | 20 |
|  | Less than the people around me (cases) | 2 | 92 | 710 | 1967 | 392 |

**Table S6. Cross-tabulation of research participant responses for itch intensity against mosquito attractiveness.** Numbers (n) of responses from unrelated European research participants who answered questions for mosquito itch intensity and mosquito bite size; scoring direction, for the phenotypic correlation analysis with mosquito attractiveness and itch intensity both scored in a negative direction.

| **Immune-related condition captured by 23andMe** |
| --- |
| Ulcerative colitis |
| Crohn’s disease |
| Type 1 diabetes |
| Rheumatoid arthritis |
| Multiple sclerosis |
| Psoriasis |
| Autoimmune hepatitis |
| Scleroderma |
| Coeliac disease |
| Systemic lupus erythematosus |
| Eczema (atopic dermatitis) |
| Rosacea |
| Severe acne |
| Keloids |
| Urticaria |
| Irritable bowel syndrome |
| Indeterminate colitis |
| Acid reflex/gastroesophageal reflux disease |
| Ankylosing spondylitis |

**Table S7. Immune-related conditions.** Participants self-reporting positive for one or more of these phenotypes were removed from itch intensity GWAS.

|  |  |  |  | Sex | | Age (years) | | | |
| --- | --- | --- | --- | --- | --- | --- | --- | --- | --- |
| Phenotype | Question | Response | Total, n (%) | Male, n (%) | Female, n (%) | ≤30, n (%) | >30 and ≤45, n (%) | >45 and ≤60 ,n (%) | >60, n (%) |
| Mosquito itch (excluding common immune-related conditions) | When you are bitten by mosquitos, how much do the bites typically itch? | Very badly (impossible to ignore) | 10704 (15.5) | 2709 (7.6) | 7995 (23.8) | 1345 (16.0) | 3363 (16.2) | 3044 (16.2) | 2952 (14.0) |
|  |  | Somewhat badly (definitely noticeable, at times hard to ignore) | 29510 (42.7) | 14083 (39.7) | 15427 (45.9) | 4168 (49.5) | 9505 (45.7) | 7592 (40.4) | 8245 (39.2) |
|  |  | Only mildly (noticeably itching, but easy to ignore) | 26135 (37.8) | 16851 (47.5) | 9284 (27.6) | 2604 (30.9) | 7158 (34.4) | 7338 (39.1) | 9035 (42.9) |
|  |  | Not at all (no noticeable itching) | 2708 (3.9) | 1817 (5.1) | 891 (2.7) | 301 (3.6) | 788 (3.8) | 804 (4.3) | 815 (3.9) |
|  |  | total | 69057 | 35460 | 33597 | 8418 | 20814 | 18778 | 21047 |

**Table S8. Mosquito itch intensity responder demographics excluding those positive for common immune-related conditions.** Phenotype, mosquito-related trait; question, survey question; response, answer options; total, n (%), sum of total unrelated European responders for that answer option and percentage; sex, total responder number for a given answer option broken down into males or females; age, responders broken down into age ranges.

| **SNP** | **Region** | **Chr** | **Position** | **SNP quality** | **Alleles (A/B)** | **BAF** | **Effect size for B allele (95% CI)** | ***P*** | **Gene context** |
| --- | --- | --- | --- | --- | --- | --- | --- | --- | --- |
| rs2248116 | 5q31.1 | 5 | 131804347 | 0.98 | A/C | 0.414 | 0.049 [0.041, 0.056] | 4.8×10^−36^ | *C5orf56-[]--IRF1* |
| rs309392 | 4q27 | 4 | 123611542 | 0.99 | G/T | 0.609 | −0.048 [-0.056, -0.041] | 2.8×10^−35^ | *IL21--[]--BBS12* |
| rs1548306 | 6p21.32 | 6 | 32427179 | 0.98 | A/T | 0.634 | −0.045 [-0.053, -0.037] | 2.6×10^−27^ | *HLA-DRA--[]--HLA-DRB5* |
| rs5750339 | 22q12.3 | 22 | 37319589 | 0.96 | C/G | 0.457 | 0.036 [0.029, 0.044] | 4.0×10^−21^ | *[CSF2RB]* |
| rs12832728 | 12q15 | 1 | 68420403 | 0.99 | A/G | 0.453 | −0.034 [-0.042, -0.027] | 3.3×10^−19^ | *DYRK2---[]---IFNG* |
| rs778798 | 19p13.3 | 19 | 5839613 | 0.99 | A/C | 0.74 | −0.034 [-0.042, -0.025] | 7.1×10^−15^ | *[FUT6]* |
| rs2967678 | 19p13.2 | 19 | 8790198 | 0.98 | C/T | 0.147 | 0.04 [0.030, 0.050] | 2.6×10^−14^ | *ADAMTS10---[]--ACTL9* |
| rs324014 | 12q13.3 | 12 | 57510309 | 0.99 | C/T | 0.566 | 0.027 [0.020, 0.035] | 1.4×10^−12^ | *STAT6-[]--LRP1* |
| rs2085423 | 7p12.2 | 7 | 50325815 | 0.97 | A/G | 0.742 | 0.03 [0.021, 0.039] | 1.3×10^−11^ | *C7orf72---[]--IKZF1* |
| rs3111414 | 2p25.1 | 2 | 8443859 | 0.98 | C/G | 0.778 | 0.029 [0.020, 0.038] | 3.4×10^−10^ | *[]---ID2* |
| rs11751172 | 6p21.1 | 6 | 45552731 | 0.99 | C/T | 0.244 | 0.028 [0.019, 0.036] | 3.4×10^−10^ | *RUNX2--[]---CLIC5* |
| rs12949918 | 17q21.2 | 17 | 40526273 | >0.99 | C/T | 0.588 | −0.023 [-0.030, -0.015] | 2.8×10^−9^ | *[STAT3]* |
| rs6809854 | 3p24.3 | 3 | 18784423 | >0.99 | A/G | 0.197 | −0.028 [-0.037, -0.019] | 4.2×10^−9^ | *SATB1---[]---KCNH8* |
| rs149082847 | 11q13.5 | 11 | 76311871 | 0.72 | A/G | 0.085 | −0.048 [-0.064, -0.031] | 7.6×10^−9^ | *C11orf30--[]--LRRC32* |
| rs2282718 | 1p36.11 | 1 | 25241056 | >0.99 | A/G | 0.605 | −0.022 [-0.029, -0.014] | 2.5×10^−8^ | *[RUNX3]* |
| rs12133641 | 1q21.3 | 1 | 154428283 | >0.99 | A/G | 0.395 | −0.021 [-0.029, -0.014] | 2.5×10^−8^ | *[IL6R]* |
| rs10261971 | 7p22.1 | 7 | 4776547 | 0.93 | A/G | 0.539 | 0.022 [0.014, 0.029] | 3.0×10^−8^ | *[FOXK1]* |
| rs13079741 | 3q28 | 3 | 188101861 | 0.99 | C/G | 0.542 | −0.021 [-0.029, -0.014] | 3.2×10^−8^ | *[LPP]* |
| rs12125543 | 1q32.1 | 1 | 204058737 | 0.99 | C/G | 0.348 | 0.022 [0.014, 0.030] | 4.6×10^−8^ | *[SOX13]* |
| rs6673928 | 1q32.1 | 1 | 206937245 | >0.99 | G/T | 0.255 | 0.023 [0.015, 0.032] | 6.4×10^−8^ | *MAPKAPK2--[]-IL10* |
| rs11265461 | 1q23.3 | 1 | 160630143 | >0.99 | C/T | 0.664 | 0.022 [0.014, 0.029] | 6.8×10^−8^ | *SLAMF1--[]--CD48* |
| rs12123821 | 1q21.3 | 1 | 152179152 | 0.72 | C/T | 0.053 | 0.053 [0.034, 0.073] | 6.9×10^−8^ | *RPTN--[]-HRNR* |
| rs72816448 | 2p16.3 | 2 | 48314623 | 0.81 | A/G | 0.977 | −0.078 [-0.106, -0.049] | 7.1×10^−8^ | *FBXO11---[]---FOXN2* |
| rs78037977 | 1q24.3 | 1 | 172715702 | 0.91 | A/G | 0.123 | 0.032 [0.020, 0.043] | 9.9×10^−8^ | *FASLG--[]---TNFSF18* |
| rs35488337 | 11q24.3 | 11 | 128418353 | 0.95 | A/G | 0.762 | −0.024 [-0.033, -0.015] | 1.0×10^−7^ | *[ETS1]* |

**Table S9. Index significant SNPs that are associated with itch intensity from mosquito bites** **(unadjusted by bite size).** Region, cytogenetic band; chr, chromosome; position, build 37 map position of the SNP; SNP quality is average r^2^ from imputation; alleles A and B are assigned based on their alphabetical order; BAF, B allele frequency across all study participants; effect size, magnitude of effect for the B allele; CI, confidence interval; ; *P,* λ adjusted significance level; gene context, gene(s) spanning or flanking (<1Mb away from) the index SNP: brackets indicate the position of the SNP, and dashes indicate distance to a flanking gene (-, >1 kb; ̶ , >10kb; ̶ ̶ , >100kb).

| **SNP** | **Region** | **Chr** | **Position** | **SNP quality** | **Alleles (A/B)** | **BAF** | **Effect size for B allele (95% CI)** | ***P*** | **Gene context** |
| --- | --- | --- | --- | --- | --- | --- | --- | --- | --- |
| rs11741255 | 5q31.1 | 5 | 131811182 | 0.97 | A/G | 0.579 | −0.032 [-0.039, -0.026] | 3.0×10^−22^ | *C5orf56--[]-IRF1* |
| rs1548306 | 6p21.32 | 6 | 32427179 | 0.98 | A/T | 0.634 | −0.026 [-0.032, -0.019] | 3.5×10^−13^ | *HLA-DRA--[]--HLA-DRB5* |
| rs309394 | 4q27 | 4 | 123613670 | 0.99 | G/T | 0.433 | 0.023 [0.017, 0.030] | 1.8×10^−12^ | *IL21--[]--BBS12* |
| rs4499342 | 19p13.2 | 19 | 8786913 | 0.98 | C/T | 0.147 | 0.03 [0.021, 0.039] | 2.4×10^−11^ | *ADAMTS10---[]--ACTL9* |
| rs10878725 | 12q15 | 12 | 68414710 | 1.00 | C/T | 0.57 | 0.021 [0.015, 0.027] | 1.2×10^−10^ | *DYRK2---[]---IFNG* |
| rs778798 | 19p13.3 | 19 | 5839613 | 0.99 | A/C | 0.74 | −0.024 [-0.031, -0.016] | 1.6×10^−10^ | *[FUT6]* |
| rs5756391 | 22q12.3 | 22 | 37298344 | 1.00 | A/G | 0.621 | −0.019 [-0.025, -0.012] | 1.4×10^−8^ | *NCF4--[]--CSF2RB* |
| rs9897389 | 17q21.2 | 17 | 40523725 | 1.00 | C/T | 0.587 | −0.017 [-0.023, -0.011] | 2.0×10^−7^ | *[STAT3]* |
| rs181336351 | 8q11.23 | 8 | 54965704 | 0.77 | A/G | 0.994 | −0.105 [-0.144, -0.065] | 2.3×10^−7^ | *[LYPLA1]* |
| rs2085423 | 7p12.2 | 7 | 50325815 | 0.97 | A/G | 0.742 | 0.019 [0.012, 0.027] | 2.8×10^−7^ | *C7orf72---[]--IKZF1* |
| rs11555872 | 22q13.2 | 22 | 43903508 | 0.95 | A/T | 0.561 | −0.017 [-0.024, -0.011] | 3.0×10^−7^ | *MPPED1[]--EFCAB6* |
| rs72816448 | 2p16.3 | 2 | 48314623 | 0.81 | A/G | 0.977 | −0.063 [-0.087, -0.039] | 3.2×10^−7^ | *FBXO11---[]---FOXN2* |
| rs324014 | 12q13.3 | 12 | 57510309 | 0.99 | C/T | 0.566 | 0.017 [0.010, 0.023] | 3.9×10^−7^ | *STAT6-[]--LRP1* |
| rs13412757 | 2p25.1 | 2 | 8458080 | 0.98 | A/G | 0.672 | −0.017 [-0.024, -0.011] | 4.4×10^−7^ | *[]---ID2* |
| rs7977602 | 12p13.31 | 12 | 6498734 | 0.95 | A/G | 0.659 | 0.017 [0.010, 0.024] | 7.0×10^−7^ | *[LTBR]* |
| rs10117812 | 9q33.3 | 9 | 128251025 | 0.99 | C/G | 0.697 | 0.017 [0.010, 0.024] | 8.0×10^−7^ | *[MAPKAP1]* |
| rs9541009 | 13q21.32 | 13 | 67696672 | 0.87 | C/T | 0.042 | −0.043 [-0.061, -0.026] | 9.1×10^−7^ | *[PCDH9]* |

**Table S10. Index significant SNPs that are associated with itch intensity from mosquito bites** **(adjusted by bite size).** Region, cytogenetic band; chr, chromosome; position, build 37 map position of the SNP; SNP quality is average r^2^ from imputation; alleles A and B are assigned based on their alphabetical order; BAF, B allele frequency across all study participants; effect size, magnitude of effect for the B allele; CI, confidence interval; ; *P,* λ adjusted significance level; gene context, gene(s) spanning or flanking (<1Mb away from) the index SNP: brackets indicate the position of the SNP, and dashes indicate distance to a flanking gene (-, >1 kb; ̶ , >10kb; ̶ ̶ , >100kb).

|  |  |  |  | Age (years) | | | |
| --- | --- | --- | --- | --- | --- | --- | --- |
| Phenotype | Question | Group | Male total, n (%) | ≤30, n (%) | >30 and ≤45, n (%) | >45 and ≤60, n (%) | >60, n (%) |
| Mosquito itch intensity, male responders only | When you are bitten by mosquitos, how much do the bites typically itch? | Very badly (impossible to ignore) | 3276 (7.9) | 353 (7.8) | 1190 (8.9) | 936 (8.5) | 797 (6.4) |
|  |  | Somewhat badly (definitely noticeable, at times hard to ignore) | 16431 (39.7) | 2143 (47.3) | 5846 (43.8) | 4112 (37.5) | 4330 (34.6) |
|  |  | Only mildly (noticeably itching, but easy to ignore) | 19498 (47.5) | 1819 (40.1) | 5646 (42.3) | 5307 (48.4) | 6726 (53.6) |
|  |  | Not at all (no noticeable itching) | 2138 (5.2) | 220 (4.9) | 662 (5.0) | 611 (5.6) | 645 (5.2) |
|  |  | total | 41343 | 4535 | 13344 | 10966 | 12498 |

**Table S11. Mosquito itch intensity, male research participant demographics.** Phenotype, mosquito-related trait; question, survey question; response, answer options; total, n (%), sum of total unrelated European responders for that answer option and percentage; sex, total responder number for a given answer option broken down into males or females; age, responders broken down into age ranges.

|  |  |  |  | Age (years) | | | |
| --- | --- | --- | --- | --- | --- | --- | --- |
| Phenotype | Question | Response | Female total, n (%) | ≤30, n (%) | >30 and ≤45, n (%) | >45 and ≤60, n (%) | >60, n (%) |
| Mosquito itch intensity, female responders only | When you are bitten by mosquitos, how much do the bites typically itch? | Very badly (impossible to ignore) | 10878 (25.1) | 1225 (25.3) | 3023 (26.9) | 3231 (25.3) | 3399 (23.4) |
|  |  | Somewhat badly (definitely noticeable, at times hard to ignore) | 19569 (45.1) | 2460 (50.8) | 5334 (47.5) | 5519 (43.3) | 6256 (43.0) |
|  |  | Only mildly (noticeably itching, but easy to ignore) | 11700 (27.0) | 1028 (21.2) | 2596 (23.1) | 3604 (28.3) | 4472 (30.8) |
|  |  | Not at all (no noticeable itching) | 1208 (2.8) | 130 (2.7) | 273 (2.4) | 392 (3.1) | 413 (2.8) |
|  |  | total | 43355 | 4843 | 11226 | 12746 | 14540 |

**Table S12. Mosquito itch intensity, female research participant demographics.** Phenotype, mosquito-related trait; question, survey question; response, answer options; total, n (%), sum of total unrelated European responders for that answer option and percentage; sex, total responder number for a given answer option broken down into males or females; age, responders broken down into age ranges.

| **SNP** | **Region** | **Chr** | **Position** | **SNP quality** | **Alleles (A/B)** | **BAF** | **Effect size for B allele (95% CI)** | ***P*** | **Gene context** |
| --- | --- | --- | --- | --- | --- | --- | --- | --- | --- |
| rs11741255 | 5q31.1 | 5 | 131811182 | 0.97 | A/G | 0.579 | −0.033 [-0.041, -0.024] | 1.2×10^−12^ | *C5orf56--[]-IRF1* |
| rs309392 | 4q27 | 4 | 123611542 | 0.99 | G/T | 0.609 | −0.026 [-0.035, -0.017] | 1.7×10^−8^ | *IL21--[]--BBS12* |
| rs28539697 | 15q25.1 | 15 | 79482907 | 0.96 | A/G | 0.301 | 0.026 [0.016, 0.035] | 1.8×10^−7^ | *RASGRF1--[]--ANKRD34C* |
| rs2523614 | 6p21.33 | 6 | 31320654 | 0.97 | C/T | 0.689 | 0.025 [0.016, 0.035] | 2.7×10^−7^ | *HLA-C--[]--MICA* |
| rs139257849 | 6p24.2 | 6 | 10725276 | 0.63 | A/G | 1 | −16.348 [-22.689, -10.008] | 4.4×10^−7^ | *[TMEM14C]* |
| rs201548632 | 4q13.2 | 4 | 69967920 | 0.60 | D/I | NA | 0.234 [0.142, 0.326] | 6.6×10^−7^ | *[UGT2B7]* |
| rs140656307 | 1p31.1 | 1 | 75013424 | 0.89 | C/T | 0 | 3.196 [1.932, 4.460] | 7.2×10^−7^ | *FPGT-TNNI3K-[]--C1orf173* |
| rs150546699 | 14q23.3 | 14 | 65440124 | 0.74 | A/G | 0.987 | 0.121 [0.073, 0.169] | 8.7×10^−7^ | *[CHURC1-FNTB]* |

**Table S13. Index significant SNPs that are associated with itch intensity from mosquito bites** **(adjusted by bite size), male research participants only.** Region, cytogenetic band; chr, chromosome; position, build 37 map position of the SNP; SNP quality is average r^2^ from imputation; alleles A and B are assigned based on their alphabetical order; BAF, B allele frequency across all study participants; effect size, magnitude of effect for the B allele; CI, confidence interval; *P,* λ adjusted significance level; gene context, gene(s) spanning or flanking (<1Mb away from) the index SNP: brackets indicate the position of the SNP, and dashes indicate distance to a flanking gene (-, >1 kb; ̶ , >10kb; ̶ ̶ , >100kb).

| **SNP** | **Region** | **Chr** | **Position** | **SNP quality** | **Alleles (A/B)** | **BAF** | **Effect size for B allele (95% CI)** | ***P*** | **Gene context** |
| --- | --- | --- | --- | --- | --- | --- | --- | --- | --- |
| rs201452941 | 6p21.32 | 6 | 32656065 | 0.7956 | D/I | 0.611 | −0.040 [-0.051, -0.028] | 1.2×10^−11^ | *HLA-DQB1--[]--HLA-DQA2* |
| rs2248116 | 5q31.1 | 5 | 131804347 | 0.983 | A/C | 0.414 | 0.032 [0.022, 0.041] | 1.4×10^−11^ | *C5orf56-[]--IRF1* |
| rs4499342 | 19p13.2 | 19 | 8786913 | 0.9764 | C/T | 0.147 | 0.037 [0.024, 0.049] | 7.8×10^−9^ | *ADAMTS10---[]--ACTL9* |
| rs186278057 | 7p12.2 | 7 | 50273159 | 0.732 | A/T | 0.015 | 0.103 [0.064, 0.142] | 2.0×10^−7^ | *C7orf72--[]--IKZF1* |
| rs192431720 | 2p25.1 | 2 | 7273716 | 0.5213 | A/G | not in DB | 0.053 [0.032, 0.073] | 5.8×10^−7^ | *RNF144A--[]* |
| rs5750339 | 22q12.3 | 22 | 37319589 | 0.9605 | C/G | 0.457 | 0.023 [0.014, 0.032] | 6.0×10^−7^ | *[CSF2RB]* |
| rs10492201 | 12q15 | 12 | 68385866 | 0.9997 | A/G | 0.866 | −0.033 [-0.045, -0.020] | 7.9×10^−7^ | *DYRK2---[]---IFNG* |
| rs309393 | 4q27 | 4 | 123613316 | 0.9927 | A/G | 0.433 | 0.023 [0.014, 0.032] | 8.1×10^−7^ | *IL21--[]--BBS12* |

**Table S14. Index significant SNPs that are associated with itch intensity from mosquito bites** **(adjusted by bite size), female research participants only.** Region, cytogenetic band; chr, chromosome; position, build 37 map position of the SNP; SNP quality is average r^2^ from imputation; alleles A and B are assigned based on their alphabetical order; BAF, B allele frequency across all study participants; effect size, magnitude of effect for the B allele; CI, confidence interval; ; *P,* λ adjusted significance level; gene context, gene(s) spanning or flanking (<1Mb away from) the index SNP: brackets indicate the position of the SNP, and dashes indicate distance to a flanking gene (-, >1 kb; ̶ , >10kb; ̶ ̶ , >100kb).

|  |  |  |  |  |  | **Female** | | | **Male** | | |  |  |
| --- | --- | --- | --- | --- | --- | --- | --- | --- | --- | --- | --- | --- | --- |
| **SNP** | **Region** | **Chr** | **Position** | **Alleles (A/B)** | **Gene context** | ***P*** | **Effect size for B allele** | **SD** | ***P*** | **Effect size for B allele** | **SD** | **Association by gender** | ***P* (interaction)** |
| rs201452941 | 6p21.32 | 6 | 32656065 | D/I | *HLA-DQB1--[]--HLA-DQA2* | 1.21x10^-11^ | -0.040 | 0.006 | 0.0032 | -0.017 | 0.006 | Female | 0.0045 |
| rs4499342 | 19p13.2 | 19 | 8786913 | C/T | *ADAMTS10---[]--ACTL9* | 7.80E x10^-9^ | 0.037 | 0.006 | 0.0002 | 0.023 | 0.006 | Female | 0.1535 |
| rs309392 | 4q27 | 4 | 123611542 | G/T | *IL21--[]--BBS12* | 6.24E x10^-6^ | -0.021 | 0.005 | 1.66 x10^-8^ | -0.026 | 0.005 | Male | 0.5480 |
| rs2248116 | 5q31.1 | 5 | 131804347 | A/C | *C5orf56-[]--IRF1* | 1.43 x10^-11^ | 0.032 | 0.005 | 1.27 x10^-12^ | 0.032 | 0.004 | Female | 0.9390 |
| rs11741255 | 5q31.1 | 5 | 131811182 | A/G | *C5orf56-[]--IRF1* | 1.48 x10^-11^ | -0.032 | 0.005 | 1.21 x10^-12^ | -0.033 | 0.005 | Male | 0.9459 |

**Table S15. Comparison of index significant SNPs identified by the males only and females only GWAS for itch intensity from mosquito bites (adjusted for bite size), with interaction term.** Associations achieving GWS (<5x10^-8^) in one gender have been illustrated, alongside association level from the complimentary analysis on the other gender analysis. Region, cytogenetic band; chr, chromosome; position, build 37 map position of the SNP; alleles A and B are assigned based on their alphabetical order; gene context, gene(s) spanning or flanking (<1Mb away from) the index SNP: brackets indicate the position of the SNP, and dashes indicate distance to a flanking gene (-, >1 kb; ̶ , >10kb; ̶ ̶ , >100kb); *P,* λ adjusted significance *p* value; effect size, magnitude of effect for the B allele; SD, standard deviation; *P* (interaction) λ adjusted significance level from the interaction test.

| Trait 1 (p1) | Trait 2 (p2) | Total SNPs | rg | se | z | *P* | h1_obs | h1_obs_se | h2_obs | h2_obs_se | h2_int | h2_int_se | gcov_int | gcov_int_se |
| --- | --- | --- | --- | --- | --- | --- | --- | --- | --- | --- | --- | --- | --- | --- |
| Itch intensity | Attractiveness | 4319563 | 1.05 | 0.189 | 5.56 | 2.70E-08 | 0.0717 | 0.0191 | 0.091 | 0.039 | 1.002 | 0.008 | 0.144 | 0.009 |
| Itch intensity | Itch intensity, adjusted by bite size, excluding prior immune conditions | 4774219 | 0.885 | 0.043 | 20.396 | 1.82E-92 | 0.0717 | 0.0191 | 0.043 | 0.013 | 1.011 | 0.011 | 0.789 | 0.013 |
| Itch intensity | Itch intensity, adjusted by bite size | 4774219 | 0.932 | 0.021 | 44.899 | 0.00E+00 | 0.0717 | 0.0191 | 0.042 | 0.013 | 1.015 | 0.013 | 0.877 | 0.014 |
| Itch intensity | Bite size | 4774219 | -0.862 | 0.036 | -23.944 | 1.08E-126 | 0.0717 | 0.0191 | 0.06 | 0.013 | 1.032 | 0.011 | -0.536 | 0.012 |
| Itch intensity, adjusted by bite size, excluding prior immune conditions | Attractiveness | 4319563 | 0.939 | 0.175 | 5.364 | 8.16E-08 | 0.0425 | 0.0129 | 0.091 | 0.039 | 1.002 | 0.008 | 0.081 | 0.007 |
| Itch intensity, adjusted by bite size, excluding prior immune conditions | Bite size | 4774219 | -0.556 | 0.14 | -3.972 | 7.14E-05 | 0.0425 | 0.0129 | 0.06 | 0.013 | 1.032 | 0.011 | -0.011 | 0.009 |
| Itch intensity, adjusted by bite size | Attractiveness | 4319563 | 0.954 | 0.164 | 5.816 | 6.04E-09 | 0.0421 | 0.0132 | 0.091 | 0.039 | 1.002 | 0.008 | 0.096 | 0.008 |
| Itch intensity, adjusted by bite size | Itch intensity, adjusted by bite size, excluding prior immune conditions | 4774219 | 0.986 | 0.013 | 75.985 | 0.00E+00 | 0.0421 | 0.0132 | 0.043 | 0.013 | 1.011 | 0.011 | 0.91 | 0.011 |
| Itch intensity, adjusted by bite size | Bite size | 4774219 | -0.642 | 0.106 | -6.051 | 1.44E-09 | 0.0421 | 0.0132 | 0.06 | 0.013 | 1.032 | 0.011 | -0.007 | 0.01 |
| Bite size | Attractiveness | 4319563 | -0.965 | 0.192 | -5.02 | 5.18E-07 | 0.0572 | 0.013 | 0.091 | 0.039 | 1.002 | 0.008 | -0.117 | 0.007 |

**Table S16. Heritability estimates and cross-trait comparisons for mosquito-related traits.** GWAS results from mosquito bite size, itch intensity (without any adjustments), itch intensity, adjusted by bite size, and itch intensity, adjusted by bite size, excluding prior immune conditions, and attractiveness to mosquitoes were analysed by LD score regression and compared pairwise (Trait 1 vs Trait 2); total SNPs, SNPs shared between both traits used for cross-trait comparison; rg, estimated correlation coefficient; se, standard error; z, z-score; *P*, cross-trait significance *p* value; h1_obs, h^2^ for Trait 1; h1_obs_se, standard error for h^2^ for Trait 1; h2_obs, h^2^ for Trait 2; h2_obs_se, standard error for h^2^ for Trait 2; h2_int, regression intercept for Trait 2; h2_int_se, standard error for regression intercept for Trait 2; gcov_int, cross-trait intercept; gcov_int_se, standard error for cross-trait intercept

|  | Bite size on attractiveness | Bite size on itch intensity | Itch intensity on attractiveness | Itch intensity on bite size | Attractiveness on bite size | Attractiveness on itch intensity |
| --- | --- | --- | --- | --- | --- | --- |
| intercept | -0.0968 | -0.0039 | 0.0835 | 0.0287 | 0.0179 | 0.0239 |
| slope | 4.3312 | 0.4254 | 1.8951 | 0.4691 | 0.2129 | -0.0031 |
| SEintercept | 0.0274 | 0.0068 | 0.1202 | 0.0280 | 0.0127 | 0.0149 |
| SEslope | 0.6035 | 0.1502 | 4.6864 | 1.0968 | 0.0698 | 0.0820 |
| *P* int | 0.0047 | 0.5764 | 0.5182 | 0.3527 | 0.3931 | 0.3544 |
| *P* slope | 1.80E-05 | 0.0163 | 0.7026 | 0.6867 | 0.2017 | 0.9760 |
| slope0 | 2.2824 | 0.3422 | 5.0966 | 1.5745 | 0.3082 | 0.1246 |
| SE0 | 0.2306 | 0.0400 | 0.8117 | 0.1955 | 0.0174 | 0.0270 |
| p.slope0 | 4.00E-07 | 1.88E-06 | 0.0008 | 0.0002 | 0.0032 | 0.0439 |

**Table S17. Egger regression results for pairwise comparisons of three mosquito-related traits**. Egger regression statistics for each comparison are provided; SE, 95% standard error; *P*, significance level; labels ending in O, denote regression through the origin.

| **Bite size associations** | | | | **Relationship** | | **Annotation** | | | | |
| --- | --- | --- | --- | --- | --- | --- | --- | --- | --- | --- |
| region | **position** | **SNP** | ***P*** | **Distance** | **r^2^** | **SNP** | ***P*** | **Study ID** | **Trait** | **Genes** |
| 6p21.32 | 32672089 | rs3134995 | 1.9×10^−24^ | −8458 | 0.94 | rs3129720 | 5.0×10^−15^ | 23472185 | Multiple sclerosis (OCB status) | *HLA-DQB1* |
| 6p21.32 | 32672089 | rs3134995 | 1.9×10^−24^ | −8458 | 0.94 | rs3129720 | 5.0×10^−7^ | 22493691 | Hypothyroidism | *HLA-DRB9, HLA-DRB5, …* |
| 6p21.32 | 32672089 | rs3134995 | 1.9×10^−24^ | 9542 | 0.588 | rs9275596 | 2.0×10^−26^ | 21399633 | Nephropathy | *HLA-DRB1, HLA-DQA1, HLA-DQB1* |
| 22q12.3 | 37319589 | rs5750339 | 9.2×10^−21^ | −9543 | 0.613 | rs2075726 | 9.0×10^−6^ | 22138694 | Ankylosing spondylitis |  |
| 5q31.1 | 131607300 | rs55722650 | 4.8×10^−19^ | −204562 | 0.64 | rs3091338 | 4.0×10^−8^ | 22412388 | Crohn’s disease | *IL3, ACSL6, …* |
| 5q31.1 | 131607300 | rs55722650 | 4.8×10^−19^ | 78846 | 0.695 | rs10058074 | 4.0×10^−12^ | 23563607 | Height | *FLJ44796* |
| 5q31.1 | 131607300 | rs55722650 | 4.8×10^−19^ | 163505 | 0.745 | rs2188962 | 1.0×10^−52^ | 23128233 | Inflammatory bowel disease | *IRF1,IL13,CSF2,SLC22A4,IL4,IL3,IL5,PDLIM4,SLC22A5,ACSL6* |
| 5q31.1 | 131607300 | rs55722650 | 4.8×10^−19^ | 163505 | 0.745 | rs2188962 | 1.0×10^−7^ | 20570966 | Crohn’s disease | *IBD5* |
| 5q31.1 | 131607300 | rs55722650 | 4.8×10^−19^ | 163505 | 0.745 | rs2188962 | 2.0×10^−18^ | 18587394 | Crohn’s disease | Intergenic |
| 5q31.1 | 131607300 | rs55722650 | 4.8×10^−19^ | 177093 | 0.74 | rs12521868 | 1.0×10^−20^ | 21102463 | Crohn’s disease | *SLC22A4,SLC22A5,IRF1,IL3* |
| 7p12.2 | 50309890 | rs62447171 | 3.5×10^−8^ | −4027 | 0.699 | rs4917014 | 3.0×10^−23^ | 19838193 | Systemic lupus erythematosus | *IKZF1* |
| 12q13.3 | 57493727 | rs3024971 | 5.9×10^−8^ | −4018 | 1 | rs1059513 | 7.0×10^−6^ | 23834954 | Sensory disturbances after bilateral sagittal split ramus osteotomy | *STAT6* |
| 12q13.3 | 57493727 | rs3024971 | 5.9×10^−8^ | −4018 | 1 | rs1059513 | 1.0×10^−14^ | 23817571 | Allergic sensitization | *STAT6* |
| 12q13.3 | 57493727 | rs3024971 | 5.9×10^−8^ | −4018 | 1 | rs1059513 | 2.0×10^−12^ | 22075330 | IgE levels | *STAT6, NAB2* |
| 2p25.1 | 8452497 | rs3102960 | 7.6×10^−8^ | −18312 | 0.512 | rs3102947 | 2.0×10^−7^ | 22075330 | IgE levels | *ID2* |
| 3q28 | 188115682 | rs9815073 | 1.1×10^−7^ | −43169 | 0.549 | rs9865818 | 3.0×10^−10^ | 23817571 | Allergic sensitization | *LPP, BCL6* |
| 3q28 | 188115682 | rs9815073 | 1.1×10^−7^ | −28054 | 0.667 | rs9851967 | 9.0×10^−8^ | 22951725 | Vitiligo | *LPP* |
| 3q28 | 188115682 | rs9815073 | 1.1×10^−7^ | −3128 | 0.522 | rs1464510 | 1.0×10^−11^ | 20410501 | Vitiligo | *LPP* |
| 3q28 | 188115682 | rs9815073 | 1.1×10^−7^ | −3128 | 0.522 | rs1464510 | 3.0×10^−40^ | 20190752 | Celiac disease | *LPP* |
| 3q28 | 188115682 | rs9815073 | 1.1×10^−7^ | −3128 | 0.522 | rs1464510 | 5.0×10^−9^ | 18311140 | Celiac disease | *LPP* |
| 3q28 | 188115682 | rs9815073 | 1.1×10^−7^ | 13297 | 0.692 | rs9860547 | 1.0×10^−9^ | 23817569 | Self-reported allergy | *LPP, BCL6* |
| 1q21.3 | 154428283 | rs12133641 | 4.3×10^−7^ | −9404 | 0.927 | rs4537545 | 2.0×10^−14^ | 19567438 | C-reactive protein | *IL6R* |
| 1q21.3 | 154428283 | rs12133641 | 4.3×10^−7^ | −2019 | 0.977 | rs4129267 | 6.0×10^−27^ | 23969696 | Fibrinogen | *IL6R* |
| 1q21.3 | 154428283 | rs12133641 | 4.3×10^−7^ | −2019 | 0.977 | rs4129267 | 2.0×10^−8^ | 21907864 | Asthma | *IL6R* |
| 1q21.3 | 154428283 | rs12133641 | 4.3×10^−7^ | −2019 | 0.977 | rs4129267 | 2.0×10^−48^ | 21300955 | C-reactive protein | *IL6R* |
| 1q21.3 | 154428283 | rs12133641 | 4.3×10^−7^ | −2019 | 0.977 | rs4129267 | 2.0×10^−57^ | 18464913 | Protein quantitative trait loci | *IL6R* |
| 1q21.3 | 154428283 | rs12133641 | 4.3×10^−7^ | −2019 | 0.977 | rs4129267 | 7.0×10^−6^ | 17903307 | Pulmonary function | *IL6R* |

**Table S18. NHGRI GWAS Catalogue annotations near mosquito bite size index SNPs.** Region, cytogenetic band; position, build 37 map position of the index SNP from Table 2; relationship, the distance and r^2^ between the index SNP and the annotation from NHGRI GWAS Catalogue; SNP, reported best proxy, defined as the SNP with the highest r^2^, within 500kb and with r^2^>0.5; *P,* corresponding significance level; study ID, PubMed identifier; trait, corresponding phenotype; gene, nearby genes to the significant SNPs.

| **Itch intensity associations** | | | | **Relationship** | | **Annotation** | | | | |
| --- | --- | --- | --- | --- | --- | --- | --- | --- | --- | --- |
| **region** | **position** | **SNP** | ***P*** | **Distance** | **r^2^** | **SNP** | ***P*** | **Study ID** | **Trait** | **Gene** |
| 5q31.1 | 131804347 | rs2248116 | 4.8×10^−36^ | −118201 | 0.653 | rs10058074 | 4.0×10^−12^ | 23563607 | Height | *FLJ44796* |
| 5q31.1 | 131804347 | rs2248116 | 4.8×10^−36^ | −33542 | 0.955 | rs2188962 | 1.0×10^−52^ | 23128233 | Inflammatory bowel disease | *IRF1,IL13,CSF2,SLC22A4,IL4,IL3,IL5,PDLIM4,SLC22A5,ACSL6* |
| 5q31.1 | 131804347 | rs2248116 | 4.8×10^−36^ | −33542 | 0.955 | rs2188962 | 1.0×10^−7^ | 20570966 | Crohn’s disease | *IBD5* |
| 5q31.1 | 131804347 | rs2248116 | 4.8×10^−36^ | −33542 | 0.955 | rs2188962 | 2.0×10^−18^ | 18587394 | Crohn’s disease | Intergenic |
| 5q31.1 | 131804347 | rs2248116 | 4.8×10^−36^ | −19954 | 0.961 | rs12521868 | 1.0×10^−20^ | 21102463 | Crohn’s disease | *SLC22A4,SLC22A5,IRF1,IL3* |
| 6p21.32 | 32427179 | rs1548306 | 2.6×10^−27^ | −15533 | 0.535 | rs7192 | 3.0×10^−6^ | 22541561 | Non-obstructive azoospermia | *HLA-DRA* |
| 6p21.32 | 32427179 | rs1548306 | 2.6×10^−27^ | −13720 | 0.535 | rs2227139 | 1.0×10^−7^ | 19820697 | Haematological parameters | Intergenic |
| 6p21.32 | 32427179 | rs1548306 | 2.6×10^−27^ | 237279 | 0.516 | rs2647012 | 2.0×10^−21^ | 21533074 | Follicular lymphoma | *HLA-DQB1* |
| 6p21.32 | 32427179 | rs1548306 | 2.6×10^−27^ | 237279 | 0.516 | rs2647012 | 8.0×10^−6^ | 21408207 | Systemic lupus erythematosus | *HLA-DQA1, HLA-DQA2* |
| 6p21.32 | 32427179 | rs1548306 | 2.6×10^−27^ | 241157 | 0.517 | rs2647046 | 2.0×10^−6^ | 23349640 | Lymphoma | *HLA-DQB1, HLA-DQA2* |
| 6p21.32 | 32427179 | rs1548306 | 2.6×10^−27^ | 254452 | 0.608 | rs9275596 | 2.0×10^−26^ | 21399633 | Nephropathy | *HLA-DRB1, HLA-DQA1, HLA-DQB1* |
| 22q12.3 | 37319589 | rs5750339 | 4.0×10^−21^ | −9543 | 0.613 | rs2075726 | 9.0×10^−6^ | 22138694 | Ankylosing spondylitis |  |
| 19p13.2 | 8790198 | rs2967678 | 2.6×10^−14^ | −817 | 1 | rs2164983 | 7.0×10^−9^ | 22197932 | Atopic dermatitis | *ACTL9* |
| 12q13.3 | 57510309 | rs324014 | 1.4×10^−12^ | −6534 | 0.682 | rs167769 | 2.0×10^−6^ | 20208534 | Eosinophilic esophagitis (paediatric) | *STAT6* |
| 12q13.3 | 57510309 | rs324014 | 1.4×10^−12^ | 16974 | 0.599 | rs11172113 | 1.0×10^−10^ | 23793025 | Migraine without aura | *LRP1* |
| 12q13.3 | 57510309 | rs324014 | 1.4×10^−12^ | 16974 | 0.599 | rs11172113 | 1.0×10^−6^ | 23793025 | Migraine - clinic-based | *LRP1* |
| 12q13.3 | 57510309 | rs324014 | 1.4×10^−12^ | 16974 | 0.599 | rs11172113 | 4.0×10^−19^ | 23793025 | Migraine | *LRP1* |
| 12q13.3 | 57510309 | rs324014 | 1.4×10^−12^ | 16974 | 0.599 | rs11172113 | 8.0×10^−6^ | 23284291 | Pulmonary function (interaction) | *LRP1* |
| 12q13.3 | 57510309 | rs324014 | 1.4×10^−12^ | 16974 | 0.599 | rs11172113 | 3.0×10^−8^ | 22683712 | Migraine | *LRP1* |
| 12q13.3 | 57510309 | rs324014 | 1.4×10^−12^ | 16974 | 0.599 | rs11172113 | 1.0×10^−8^ | 21946350 | Pulmonary function | *LRP1* |
| 12q13.3 | 57510309 | rs324014 | 1.4×10^−12^ | 16974 | 0.599 | rs11172113 | 4.0×10^−9^ | 21666692 | Migraine | *LRP1* |
| 17q21.2 | 40526273 | rs12949918 | 2.8×10^−9^ | −18293 | 0.774 | rs9891119 | 2.0×10^−15^ | 23266558 | Crohn’s disease | *STAT3* |
| 17q21.2 | 40526273 | rs12949918 | 2.8×10^−9^ | −18293 | 0.774 | rs9891119 | 2.0×10^−10^ | 21833088 | Multiple sclerosis | *STAT3* |
| 17q21.2 | 40526273 | rs12949918 | 2.8×10^−9^ | −12072 | 0.979 | rs744166 | 3.0×10^−10^ | 20159113 | Multiple sclerosis | *STAT3* |
| 17q21.2 | 40526273 | rs12949918 | 2.8×10^−9^ | −12072 | 0.979 | rs744166 | 7.0×10^−12^ | 18587394 | Crohn’s disease | *STAT3* |
| 17q21.2 | 40526273 | rs12949918 | 2.8×10^−9^ | 1271 | 0.962 | rs12942547 | 6.0×10^−22^ | 23128233 | Inflammatory bowel disease | *STAT3,STAT5B,STAT5A* |
| 3p24.3 | 18784423 | rs6809854 | 4.2×10^−9^ | 0 | 1 | rs6809854 | 1.0×10^−7^ | 20953190 | Psoriasis | Intergenic |
| 1q21.3 | 154428283 | rs12133641 | 2.5×10^−8^ | −9404 | 0.927 | rs4537545 | 2.0×10^−14^ | 19567438 | C-reactive protein | *IL6R* |
| 1q21.3 | 154428283 | rs12133641 | 2.5×10^−8^ | −2019 | 0.977 | rs4129267 | 6.0×10^−27^ | 23969696 | Fibrinogen | *IL6R* |
| 1q21.3 | 154428283 | rs12133641 | 2.5×10^−8^ | −2019 | 0.977 | rs4129267 | 2.0×10^−8^ | 21907864 | Asthma | *IL6R* |
| 1q21.3 | 154428283 | rs12133641 | 2.5×10^−8^ | −2019 | 0.977 | rs4129267 | 2.0×10^−48^ | 21300955 | C-reactive protein | *IL6R* |
| 1q21.3 | 154428283 | rs12133641 | 2.5×10^−8^ | −2019 | 0.977 | rs4129267 | 2.0×10^−57^ | 18464913 | Protein quantitative trait loci | *IL6R* |
| 1q21.3 | 154428283 | rs12133641 | 2.5×10^−8^ | −2019 | 0.977 | rs4129267 | 7.0×10^−6^ | 17903307 | Pulmonary function | *IL6R* |
| 3q28 | 188101861 | rs13079741 | 3.2×10^−8^ | −29348 | 0.62 | rs9865818 | 3.0×10^−10^ | 23817571 | Allergic sensitization | *LPP, BCL6* |
| 3q28 | 188101861 | rs13079741 | 3.2×10^−8^ | −14233 | 0.773 | rs9851967 | 9.0×10^−8^ | 22951725 | Vitiligo | *LPP* |
| 3q28 | 188101861 | rs13079741 | 3.2×10^−8^ | 10693 | 0.949 | rs1464510 | 1.0×10^−11^ | 20410501 | Vitiligo | *LPP* |
| 3q28 | 188101861 | rs13079741 | 3.2×10^−8^ | 10693 | 0.949 | rs1464510 | 3.0×10^−40^ | 20190752 | Celiac disease | *LPP* |
| 3q28 | 188101861 | rs13079741 | 3.2×10^−8^ | 10693 | 0.949 | rs1464510 | 5.0×10^−9^ | 18311140 | Celiac disease | *LPP* |
| 3q28 | 188101861 | rs13079741 | 3.2×10^−8^ | 27118 | 0.786 | rs9860547 | 1.0×10^−9^ | 23817569 | Self-reported allergy | *LPP, BCL6* |
| 1q23.3 | 160630143 | rs11265461 | 6.8×10^−8^ | 0 | 1 | rs11265461 | 2.0×10^−7^ | 22479419 | Schizophrenia (treatment refractory) | *SLAMF1* |

**Table S19. NHGRI GWAS Catalogue annotations near itch intensity from mosquito bite index SNPs.** Region, cytogenetic band; position, build 37 map position of the index SNP from Supplementary Table 9; relationship, the distance and r^2^ between the index SNP and the annotation from NHGRI GWAS Catalogue; SNP, reported best proxy, defined as the SNP with the highest r^2^, within 500kb and with r^2^>0.5; *P,* corresponding significance level; study ID, PubMed identifier; trait, corresponding phenotype; gene, nearby genes to the significant SNPs.

| **Itch intensity adjusted by bite size** | | | | **Relationship** | | **Annotation** | | | | |
| --- | --- | --- | --- | --- | --- | --- | --- | --- | --- | --- |
| region | position | **SNP** | ***P*** | **Distance** | **r^2^** | **SNP** | ***P*** | **Study ID** | **Trait** | **Gene** |
| 5q31.1 | 131811182 | rs11741255 | 3.0×10^−22^ | −125036 | 0.629 | rs10058074 | 4.0×10^−12^ | 23563607 | Height | *FLJ44796* |
| 5q31.1 | 131811182 | rs11741255 | 3.0×10^−22^ | −40377 | 0.928 | rs2188962 | 1.0×10^−52^ | 23128233 | Inflammatory bowel disease | *IRF1,IL13,CSF2,SLC22A4,IL4,IL3,IL5,PDLIM4,SLC22A5,ACSL6* |
| 5q31.1 | 131811182 | rs11741255 | 3.0×10^−22^ | −40377 | 0.928 | rs2188962 | 1.0×10^−7^ | 20570966 | Crohn’s disease | *IBD5* |
| 5q31.1 | 131811182 | rs11741255 | 3.0×10^−22^ | −40377 | 0.928 | rs2188962 | 2.0×10^−18^ | 18587394 | Crohn’s disease | Intergenic |
| 5q31.1 | 131811182 | rs11741255 | 3.0×10^−22^ | −26789 | 0.933 | rs12521868 | 1.0×10^−20^ | 21102463 | Crohn’s disease | *SLC22A4,SLC22A5,IRF1,IL3* |
| 6p21.32 | 32427179 | rs1548306 | 3.5×10^−13^ | −15533 | 0.535 | rs7192 | 3.0×10^−6^ | 22541561 | Non-obstructive azoospermia | *HLA-DRA* |
| 6p21.32 | 32427179 | rs1548306 | 3.5×10^−13^ | −13720 | 0.535 | rs2227139 | 1.0×10^−7^ | 19820697 | Haematological parameters | Intergenic |
| 6p21.32 | 32427179 | rs1548306 | 3.5×10^−13^ | 237279 | 0.516 | rs2647012 | 2.0×10^−21^ | 21533074 | Follicular lymphoma | *HLA-DQB1* |
| 6p21.32 | 32427179 | rs1548306 | 3.5×10^−13^ | 237279 | 0.516 | rs2647012 | 8.0×10^−6^ | 21408207 | Systemic lupus erythematosus | *HLA-DQA1, HLA-DQA2* |
| 6p21.32 | 32427179 | rs1548306 | 3.5×10^−13^ | 241157 | 0.517 | rs2647046 | 2.0×10^−6^ | 23349640 | Lymphoma | *HLA-DQB1, HLA-DQA2* |
| 6p21.32 | 32427179 | rs1548306 | 3.5×10^−13^ | 254452 | 0.608 | rs9275596 | 2.0×10^−26^ | 21399633 | Nephropathy | *HLA-DRB1, HLA-DQA1, HLA-DQB1* |
| 19p13.2 | 8786913 | rs4499342 | 2.4×10^−11^ | 2468 | 0.991 | rs2164983 | 7.0×10^−9^ | 22197932 | Atopic dermatitis | *ACTL9* |
| 17q21.2 | 40523725 | rs9897389 | 2.0×10^−7^ | −15745 | 0.791 | rs9891119 | 2.0×10^−15^ | 23266558 | Crohn’s disease | *STAT3* |
| 17q21.2 | 40523725 | rs9897389 | 2.0×10^−7^ | −15745 | 0.791 | rs9891119 | 2.0×10^−10^ | 21833088 | Multiple sclerosis | *STAT3* |
| 17q21.2 | 40523725 | rs9897389 | 2.0×10^−7^ | −9524 | 0.995 | rs744166 | 3.0×10^−10^ | 20159113 | Multiple sclerosis | *STAT3* |
| 17q21.2 | 40523725 | rs9897389 | 2.0×10^−7^ | −9524 | 0.995 | rs744166 | 7.0×10^−12^ | 18587394 | Crohn’s disease | *STAT3* |
| 17q21.2 | 40523725 | rs9897389 | 2.0×10^−7^ | 3819 | 0.979 | rs12942547 | 6.0×10^−22^ | 23128233 | Inflammatory bowel disease | *STAT3,STAT5B,STAT5A* |
| 12q13.3 | 57510309 | rs324014 | 3.9×10^−7^ | −6534 | 0.682 | rs167769 | 2.0×10^−6^ | 20208534 | Eosinophilic esophagitis (paediatric) | *STAT6* |
| 12q13.3 | 57510309 | rs324014 | 3.9×10^−7^ | 16974 | 0.599 | rs11172113 | 1.0×10^−10^ | 23793025 | Migraine without aura | *LRP1* |
| 12q13.3 | 57510309 | rs324014 | 3.9×10^−7^ | 16974 | 0.599 | rs11172113 | 1.0×10^−6^ | 23793025 | Migraine - clinic-based | *LRP1* |
| 12q13.3 | 57510309 | rs324014 | 3.9×10^−7^ | 16974 | 0.599 | rs11172113 | 4.0×10^−19^ | 23793025 | Migraine | *LRP1* |
| 12q13.3 | 57510309 | rs324014 | 3.9×10^−7^ | 16974 | 0.599 | rs11172113 | 8.0×10^−6^ | 23284291 | Pulmonary function (interaction) | *LRP1* |
| 12q13.3 | 57510309 | rs324014 | 3.9×10^−7^ | 16974 | 0.599 | rs11172113 | 3.0×10^−8^ | 22683712 | Migraine | *LRP1* |
| 12q13.3 | 57510309 | rs324014 | 3.9×10^−7^ | 16974 | 0.599 | rs11172113 | 1.0×10^−8^ | 21946350 | Pulmonary function | *LRP1* |
| 12q13.3 | 57510309 | rs324014 | 3.9×10^−7^ | 16974 | 0.599 | rs11172113 | 4.0×10^−9^ | 21666692 | Migraine | *LRP1* |
| 2p25.1 | 8458080 | rs13412757 | 4.4×10^−7^ | −15832 | 0.761 | rs10174949 | 1.0×10^−7^ | 23817569 | Self-reported allergy | *ID2* |

**Table S20. NHGRI GWAS Catalogue annotations for index SNPs near itch intensity from mosquito bite (adjusted by bite size).** Region, cytogenetic band; position, build 37 map position of the index SNP from Supplementary Table 10; relationship, the distance and r^2^ between the index SNP and the annotation from NHGRI GWAS Catalogue; SNP, reported best proxy, defined as the SNP with the highest r^2^, within 500kb and with r^2^>0.5; *P,* corresponding significance level; study ID, PubMed identifier; trait, corresponding phenotype; gene, nearby genes to the significant SNPs.

| **Itch intensity adjusted by bite size and excluding responders positive for common immune-related conditions** | | | | **Relationship** | | **Annotation** | | | | |
| --- | --- | --- | --- | --- | --- | --- | --- | --- | --- | --- |
| **region** | **position** | **SNP** | ***P*** | **Distance** | **r^2^** | **SNP** | ***P*** | **Study ID** | **trait** | **Gene** |
| 5q31.1 | 131804347 | rs2248116 | 3.5×10^−21^ | −151818 | 0.667 | rs11950562 | 7.0×10^−11^ | 24816252 | Blood metabolite levels | *SLC22A4* |
| 5q31.1 | 131804347 | rs2248116 | 3.5×10^−21^ | −118201 | 0.653 | rs10058074 | 4.0×10^−12^ | 23563607 | Height | *FLJ44796* |
| 5q31.1 | 131804347 | rs2248116 | 3.5×10^−21^ | −33542 | 0.955 | rs2188962 | 1.0×10^−52^ | 23128233 | Inflammatory bowel disease | *IRF1,IL13,CSF2,SLC22A4,IL4,IL3,IL5,PDLIM4,SLC22A5,ACSL6* |
| 5q31.1 | 131804347 | rs2248116 | 3.5×10^−21^ | −33542 | 0.955 | rs2188962 | 1.0×10^−7^ | 20570966 | Crohn’s disease | *IBD5* |
| 5q31.1 | 131804347 | rs2248116 | 3.5×10^−21^ | −33542 | 0.955 | rs2188962 | 2.0×10^−18^ | 18587394 | Crohn’s disease | Intergenic |
| 5q31.1 | 131804347 | rs2248116 | 3.5×10^−21^ | −19954 | 0.961 | rs12521868 | 1.0×10^−20^ | 21102463 | Crohn’s disease | *SLC22A4,SLC22A5,IRF1,IL3* |
| 6p21.32 | 32626739 | rs12055445 | 1.5×10^−10^ | 31571 | 0.737 | rs9469220 | 2.0×10^−7^ | 23146381 | IgE levels | *HLA-DQB1* |
| 6p21.32 | 32626739 | rs12055445 | 1.5×10^−10^ | 31571 | 0.737 | rs9469220 | 2.0×10^−6^ | 17554300 | Crohn’s disease |  |
| 6p21.32 | 32626739 | rs12055445 | 1.5×10^−10^ | 54892 | 0.531 | rs9275596 | 2.0×10^−26^ | 21399633 | Nephropathy | *HLA-DRB1, HLA-DQA1, HLA-DQB1* |
| 19p13.2 | 8786913 | rs4499342 | 2.7×10^−9^ | 2468 | 0.991 | rs2164983 | 7.0×10^−9^ | 22197932 | Atopic dermatitis | *ACTL9* |

**Table S21. NHGRI GWAS Catalogue annotations for index SNPs near itch intensity from mosquito bite (adjusted for bite size and excluding responders positive for common immune-related conditions).** Region, cytogenetic band; position, build 37 map position of the index SNP from Table 3; relationship, the distance and r^2^ between the index SNP and the annotation from NHGRI GWAS Catalogue; SNP, reported best proxy, defined as the SNP with the highest r^2^, within 500kb and with r^2^>0.5; *P,* corresponding significance level; study ID, PubMed identifier; trait, corresponding phenotype; gene, nearby genes to the significant SNPs.

| **Attractiveness** | | | | **Relationship** | | **Annotation** | | | | |
| --- | --- | --- | --- | --- | --- | --- | --- | --- | --- | --- |
| **region** | **position** | **SNP** | ***P*** | **Distance** | **r^2^** | **SNP** | ***P*** | **Study ID** | **Trait** | **Genes** |
| 5q31.1 | 131371999 | rs1858074 | 2.40×10^−9^ | 30739 | 0.603 | rs3091338 | 4.00×10^−8^ | 22412388 | Crohn’s disease | *IL3, ACSL6, P4HA2, PDLIM4, SLC22A4* |
| 6p21.32 | 32410941 | rs9268659 | 3.50×10^−9^ | -187683 | 0.546 | rs3130320 | 3.00E×10^−6^ | 21408207 | Systemic lupus erythematosus | *NOTCH4, C6orf10* |
| 6p21.32 | 32410941 | rs9268659 | 3.50×10^−9^ | -2414 | 0.504 | rs9268645 | 1.00×10^−100^ | 19430480 | Type 1 diabetes | *MHC* |
| 6p21.32 | 32410941 | rs9268659 | 3.50×10^−9^ | 705 | 0.802 | rs7192 | 3.00×10^−6^ | 22541561 | Non-obstructive azoospermia | *HLA-DRA* |
| 6p21.32 | 32410941 | rs9268659 | 3.50×10^−9^ | 2518 | 0.789 | rs2227139 | 1.00×10^−7^ | 19820697 | Haematological parameters | Intergenic |

**Supplementary Table S22. NHGRI GWAS Catalogue annotations for near attractiveness to mosquito index SNPs.** Region, cytogenetic band; position, build 37 map position of the index SNP from Table 4; relationship, the distance and r^2^ between the index SNP and the annotation from NHGRI GWAS Catalogue; SNP, reported best proxy, defined as the SNP with the highest r^2^, within 500kb and with r^2^>0.5; *P,* corresponding significance level; study ID, PubMed identifier; trait, corresponding phenotype; gene, nearby genes to the significant SNPs.

|  | Mosquito-phenotype association | | | | Immunochip association | | | |
| --- | --- | --- | --- | --- | --- | --- | --- | --- |
| Mosquito trait dataset | Region | Position | SNP | *P* | Trait | *P* | Gene | Study ID |
| Itch intensity (unadjusted by bite size) | 17q21.2 | 40526273 | rs12949918 | 2.8×10^−9^ | Crohn's disease | 1.4x10^-7^ | *STAT3* | 21102463 |
| Itch intensity (unadjusted by bite size) | 17q21.2 | 40526273 | rs12949918 | 2.8×10^−9^ | Ulcerative colitis | 1.0x10^-6^ | *STAT3* | 21297633 |
| Itch intensity (unadjusted by bite size) | 17q21.2 | 40526273 | rs12949918 | 2.8×10^−9^ | Multiple sclerosis | 2.0x10^-5^ | *STAT3* | 18971939 |
| Itch intensity (unadjusted by bite size) | 3q28 | 188101861 | rs13079741 | 3.2×10^−8^ | Celiac disease | 8.7x10^-42^ | *LPP* | 22057235 |
| Itch intensity (unadjusted by bite size) | 3q28 | 188101861 | rs13079741 | 3.2×10^−8^ | Autoimmune thyroid disease | 1.0x10^-6^ | *LPP* | 22922229 |
| Itch intensity (adjusted by bite size, excluding those positive for an immune condition) | 22q12.3 | 37298344 | rs5756391 | 5.5×10^−7^ | Multiple sclerosis | 3.5x10^-5^ |  | 18971939 |
| Bite size | 9q32 | 117693631 | rs2075533 | 4.7×10^−7^ | Multiple sclerosis | 2.6x10^-4^ | *TNFSF8* | 18971939 |
| Bite size | 3q28 | 188115682 | rs9815073 | 1.1×10^−7^ | Autoimmune thyroid disease | 1.4x10^-4^ | *LPP* | 22922229 |
| Itch intensity (unadjusted by bite size) | 11q24.3 | 128418353 | rs35488337 | 1.0×10^−7^ | Celiac disease | 8.2x10^-5^ | *ETS1* | 22057235 |

**Supplementary Table S23. Immunobase annotations for mosquito-related trait index SNPs.** Region, cytogenetic band; position, build 37 map position of the corresponding index SNP from the mosquito trait dataset indicated; SNP, index SNP; *P,* significance level; annotation from Immunobase; trait, immune disease associated with the mosquito trait SNP; *P,* corresponding significance level; gene; nearby genes to the significant SNP, Study ID, PubMed identifier.

|  | **Mosquito-trait association** | | | | **Relationship** | | **Nonsynonymous SNP annotation** | | |
| --- | --- | --- | --- | --- | --- | --- | --- | --- | --- |
| **Dataset** | **region** | **position** | **SNP** | ***P*** | **Distance** | **r^2^** | **SNP** | **Gene** | **Amino acid change** |
| Bite size | 6p21.32 | 32672089 | rs3134995 | 1.9×10^−24^ | −61628 | 0.505 | rs9260 | *HLA-DQA1* | M230V |
| Bite size | 5q31.1 | 131607300 | rs55722650 | 4.8×10^−19^ | 69020 | 0.851 | rs1050152 | *SLC22A4* | L503F |
| Bite size | 2q21.3 | 136707982 | rs6754311 | 1.6×10^−10^ | −152323 | 0.601 | rs2322659 | *LCT* | N1639S |
| Bite size | 1p36.22 | 12175658 | rs2230624 | 1.1×10^−7^ | 0 | 1 | rs2230624 | *TNFRSF8* | C273? |
| Bite size | 1q21.3 | 154428283 | rs12133641 | 4.3×10^−7^ | −1313 | 0.972 | rs2228145 | *IL6R* | D358A |
| Bite size | 5q31.1 | 131804347 | rs2248116 | 4.8×10^−36^ | −128027 | 0.835 | rs1050152 | *SLC22A4* | L503F |
| Bite size | 6p21.32 | 32427179 | rs1548306 | 2.6×10^−27^ | −15533 | 0.535 | rs7192 | *HLA-DRA* | L242V |
| Bite size | 1q21.3 | 154428283 | rs12133641 | 2.5×10^−8^ | −1313 | 0.972 | rs2228145 | *IL6R* | D358A |
| Itch intensity adjusted by bite size | 5q31.1 | 131811182 | rs11741255 | 3.0×10^−22^ | −134862 | 0.808 | rs1050152 | *SLC22A4* | L503F |
| Itch intensity adjusted by bite size | 6p21.32 | 32427179 | rs1548306 | 3.5×10^−13^ | −15533 | 0.535 | rs7192 | *HLA-DRA* | L242V |
| Itch intensity (adjusted by bite size, excluding those positive for an immune condition) | 5q31.1 | 131804347 | rs2248116 | 3.5×10^−21^ | −128027 | 0.835 | rs1050152 | *SLC22A4* | L503F |
| Attractiveness | 6p21.32 | 32410941 | rs9268659 | 3.50E-09 | 705 | 0.802 | rs7192 | *HLA-DRA* | L242V |
| Attractiveness | 6p21.33 | 31836827 | rs521977 | 5.90E-07 | 92187 | 0.544 | rs437179 | *SKIV2L* | M214L |

**Table S24.Nonsynonymous SNPs near mosquito-trait index SNPs.** Dataset, mosquito-associated trait; accompanying region, position, index SNP, and significance level; relationship, distance and r^2^ between the mosquito-trait index SNP and the nonsynonymous SNP annotation; includes the identifier for SNPs within 500kb and r^2^>0.5, affected gene and amino acid change associated with this SNP. Coding SNP annotations were taken from the UCSC Genome Browser’s snp138CodingDbSnp table.

|  | Mosquito-phenotype association | | | | Relationship | | eQTL annotation | | | | | | |
| --- | --- | --- | --- | --- | --- | --- | --- | --- | --- | --- | --- | --- | --- |
| Dataset | region | position | SNP | *P* | Distance | r2 | SNP | Distance | Gene | *P* | r2 | Tissue | Study ID |
| Bite size | 12q15 | 68390201 | rs2906856 | 7.9x10^-12^ | 181 | 0.856 | rs1468487 | 255967 | *IL22* | 3.1x10^-5^ | 0.06 | B-Cell | 22446964 |
| Bite size | 1q21.3 | 154428283 | rs12133641 | 4.3x10^-7^ | -32444 | 0.892 | rs6684439 | 538776 | *PYGO2* | 3.0x10^-6^ | 0.179 | Brain | 25174004 |
| Bite size | 1q21.3 | 154428283 | rs12133641 | 4.3x10^-7^ | -12887 | 0.884 | rs4845372 | 475208 | *SLC39A1* | 4.8x10^-6^ | 0.173 | Brain | 25174004 |
| Bite size | 1q21.3 | 154428283 | rs12133641 | 4.3x10^-7^ | -9534 | 0.933 | rs4576655 | 718366 | *INTS3* | 5.6x10^-6^ | 0.171 | Brain | 25174004 |
| Bite size | 2q21.3 | 136707982 | rs6754311 | 1.6x10^-10^ | -380914 | 0.77 | rs4988226 | 12406 | *MCM6* | 6.2x10^-184^ |  | Whole_Blood | 24013639 |
| Bite size | 2q21.3 | 136707982 | rs6754311 | 1.6x10^-10^ | -93727 | 0.77 | rs309180 | 0 | *MCM6* | 3.5x10^-43^ | 0.123 | Monocyte | 20502693 |
| Bite size | 2q21.3 | 136707982 | rs6754311 | 1.6x10^-10^ | -22754 | 0.781 | rs309160 | 51232 | *MCM6* | 2.4x10^-26^ |  | Whole_Blood | 24092820 |
| Bite size | 2q21.3 | 136707982 | rs6754311 | 1.6x10^-10^ | -22754 | 0.781 | rs309160 | -87036 | *MCM6* | 3.2x10^-9^ | 0.118 | Monocyte | 22446964 |
| Bite size | 2q21.3 | 136707982 | rs6754311 | 1.6x10^-10^ | -166240 | 0.516 | rs932206 | 47244 | *CXCR4* | 3.7x10^-7^ |  | Whole_Blood | 24013639 |
| Bite size | 2q21.3 | 136707982 | rs6754311 | 1.6x10^-10^ | 109634 | 0.641 | rs2011946 | 183620 | *MCM6* | 5.4x10^-7^ | 0.271 | Nerve_Tibial | 23715323 |
| Bite size | 2q21.3 | 136707982 | rs6754311 | 1.6x10^-10^ | 78669 | 0.698 | rs12475139 | 152655 | *MCM6* | 6.6x10^-7^ | 0.22 | Artery_Tibial | 23715323 |
| Bite size | 2q21.3 | 136707982 | rs6754311 | 1.6x10^-10^ | -71658 | 0.633 | rs55830620 | 2328 | *MCM6* | 2.5x10^-6^ | 0.349 | Artery_Aorta | 23715323 |
| Bite size | 2q21.3 | 136707982 | rs6754311 | 1.6x10^-10^ | -228680 | 0.59 | rs3754692 | 184945 | *DARS* | 4.3x10^-6^ | 0.212 | Adipose_Subcutaneous | 23715323 |
| Bite size | 2q21.3 | 136707982 | rs6754311 | 1.6x10^-10^ | -152323 | 0.601 | rs2322659 | 42533 | *MCM6* | 0.00021 | 0.048 | B-Cell | 22446964 |
| Bite size | 3q28 | 188115682 | rs9815073 | 1.1x10^-7^ | -10626 | 0.646 | rs9864529 | 641581 | *BCL6* | 5.2x10^-10^ | 0.028 | Monocyte | 20502693 |
| Bite size | 5q31.1 | 131607300 | rs55722650 | 4.8x10^-19^ | 106745 | 0.695 | rs10058074 | 44836 | *SLC22A5* | 9.8x10^-198^ |  | Whole_Blood | 24013639 |
| Bite size | 5q31.1 | 131607300 | rs55722650 | 4.8x10^-19^ | 96919 | 0.851 | rs1050152 | 3277 | *SLC22A4* | 4.5x10^-92^ |  | Whole_Blood | 24013639 |
| Bite size | 5q31.1 | 131607300 | rs55722650 | 4.8x10^-19^ | 98158 | 0.687 | rs2631367 | 0 | *SLC22A5* | 8x10^-90^ |  | Whole_Blood | 24092820 |
| Bite size | 5q31.1 | 131607300 | rs55722650 | 4.8x10^-19^ | 100129 | 0.687 | rs2631360 | 0 | *SLC22A5* | 6.7x10^-85^ | 0.23 | Monocyte | 20502693 |
| Bite size | 5q31.1 | 131607300 | rs55722650 | 4.8x10^-19^ | 109750 | 0.699 | rs17622208 | 13932 | *SLC22A5* | 4.3x10^-37^ | 0.44 | Monocyte | 22446964 |
| Bite size | 5q31.1 | 131607300 | rs55722650 | 4.8x10^-19^ | -43799 | 0.713 | rs113823725 | 0 | *P4HA2* | 2.1x10^-16^ | 0.529 | Thyroid | 23715323 |
| Bite size | 5q31.1 | 131607300 | rs55722650 | 4.8x10^-19^ | -44400 | 0.756 | rs72793280 | 34399 | *P4HA2-AS1* | 2.8x10^-12^ | 0.42 | Thyroid | 23715323 |
| Bite size | 5q31.1 | 131607300 | rs55722650 | 4.8x10^-19^ | 109750 | 0.699 | rs17622208 | 13932 | *SLC22A5* | 1.4x10^-11^ | 0.151 | B-Cell | 22446964 |
| Bite size | 5q31.1 | 131607300 | rs55722650 | 4.8x10^-19^ | -31962 | 0.717 | rs12521097 | 11642 | *P4HA2* | 4.4x10^-9^ | 0.256 | Brain | 25174004 |
| Bite size | 5q31.1 | 131607300 | rs55722650 | 4.8x10^-19^ | -51126 | 0.503 | rs6596070 | 149270 | *SLC22A5* | 5.2x10^-9^ | 0.311 | Skin_Sun_Exposed_Lower_leg | 23715323 |
| Bite size | 5q31.1 | 131607300 | rs55722650 | 4.8x10^-19^ | 100129 | 0.687 | rs2631360 | 0 | *SLC22A5* | 6.5x10^-9^ | 0.276 | Whole_Blood | 23715323 |
| Bite size | 5q31.1 | 131607300 | rs55722650 | 4.8x10^-19^ | -15575 | 0.773 | rs112537099 | 39283 | *P4HA2* | 4.4x10^-8^ | 0.078 | Lymphoblastoid | 24037378 |
| Bite size | 5q31.1 | 131607300 | rs55722650 | 4.8x10^-19^ | -198458 | 0.719 | rs1469149 | 267437 | *SLC22A4* | 1.7x10^-7^ | 0.093 | Monocyte | 22446964 |
| Bite size | 5q31.1 | 131607300 | rs55722650 | 4.8x10^-19^ | -45928 | 0.717 | rs72793278 | 31992 | *PDLIM4* | 2.2x10^-7^ | 0.254 | Skin_Sun_Exposed_Lower_leg | 23715323 |
| Bite size | 5q31.1 | 131607300 | rs55722650 | 4.8x10^-19^ | 100129 | 0.687 | rs2631360 | 0 | *SLC22A5* | 4.6x10^-7^ | 0.225 | Lung | 23715323 |
| Bite size | 5q31.1 | 131607300 | rs55722650 | 4.8x10^-19^ | 86777 | 0.707 | rs4705938 | 20331 | *SLC22A5* | 1.3x10^-6^ | 0.19 | Brain | 25174004 |
| Bite size | 5q31.1 | 131607300 | rs55722650 | 4.8x10^-19^ | -170814 | 0.513 | rs10065787 | 268958 | *SLC22A5* | 4.0x10^-6^ | 0.254 | Esophagus_Mucosa | 23715323 |
| Bite size | 5q31.1 | 131607300 | rs55722650 | 4.8x10^-19^ | 45229 | 0.699 | rs11950562 | 0 | *AC034220.3* | 4.9x10^-6^ | 0.184 | Whole_Blood | 23715323 |
| Bite size | 5q31.1 | 131607300 | rs55722650 | 4.8x10^-19^ | -16766 | 0.548 | rs7727544 | 419037 | *IL4* | 1.4x10^-5^ | 0.065 | B-Cell | 22446964 |
| Bite size | 6p21.32 | 32672089 | rs3134995 | 1.9x10^-24^ | -41581 | 0.585 | rs9274184 | 0 | *HLA-DQB1* | 2.5x10^-40^ | 0.776 | Whole_Blood | 23715323 |
| Bite size | 6p21.32 | 32672089 | rs3134995 | 1.9x10^-24^ | -38494 | 0.789 | rs9274469 | 0 | *HLA-DQB1* | 9x10^-35^ | 0.778 | Lung | 23715323 |
| Bite size | 6p21.32 | 32672089 | rs3134995 | 1.9x10^-24^ | -40354 | 0.597 | chr6:32631735:I | 0 | *HLA-DQB1* | 1.5x10^-33^ | 0.765 | Artery_Tibial | 23715323 |
| Bite size | 6p21.32 | 32672089 | rs3134995 | 1.9x10^-24^ | -38211 | 0.74 | rs9274489 | 0 | *HLA-DQB1* | 1.1x10^-31^ | 0.788 | Adipose_Subcutaneous | 23715323 |
| Bite size | 6p21.32 | 32672089 | rs3134995 | 1.9x10^-24^ | -38958 | 0.732 | rs9274436 | 0 | *HLA-DQB1* | 3.7x10^-30^ | 0.766 | Thyroid | 23715323 |
| Bite size | 6p21.32 | 32672089 | rs3134995 | 1.9x10^-24^ | -38958 | 0.732 | rs9274436 | 2904 | *XXbac-BPG254F23.6* | 1.1x10^-28^ | 0.708 | Lung | 23715323 |
| Bite size | 6p21.32 | 32672089 | rs3134995 | 1.9x10^-24^ | -41884 | 0.698 | rs9274115 | 0 | *HLA-DQB1* | 1.9x10^-27^ | 0.773 | Nerve_Tibial | 23715323 |
| Bite size | 6p21.32 | 32672089 | rs3134995 | 1.9x10^-24^ | -38259 | 0.663 | rs9274485 | 0 | *HLA-DQB1* | 3.3x10^-27^ | 0.72 | Skin_Sun_Exposed_Lower_leg | 23715323 |
| Bite size | 6p21.32 | 32672089 | rs3134995 | 1.9x10^-24^ | -45007 | 0.615 | rs9273394 | 1050 | *HLA-DQB1-AS1* | 2.6x10^-24^ | 0.689 | Adipose_Subcutaneous | 23715323 |
| Bite size | 6p21.32 | 32672089 | rs3134995 | 1.9x10^-24^ | -38345 | 0.861 | rs9274478 | 3517 | *XXbac-BPG254F23.6* | 3x10^-24^ | 0.727 | Nerve_Tibial | 23715323 |
| Bite size | 6p21.32 | 32672089 | rs3134995 | 1.9x10^-24^ | -40353 | 0.612 | rs9274281 | 1509 | *XXbac-BPG254F23.6* | 4x10^-23^ | 0.657 | Skin_Sun_Exposed_Lower_leg | 23715323 |
| Bite size | 6p21.32 | 32672089 | rs3134995 | 1.9x10^-24^ | -41581 | 0.585 | rs9274184 | 0 | *HLA-DQB1* | 6.2x10^-23^ | 0.738 | Esophagus_Mucosa | 23715323 |
| Bite size | 6p21.32 | 32672089 | rs3134995 | 1.9x10^-24^ | -45007 | 0.615 | rs9273394 | 1050 | *HLA-DQB1-AS1* | 2.6x10^-20^ | 0.691 | Esophagus_Mucosa | 23715323 |
| Bite size | 6p21.32 | 32672089 | rs3134995 | 1.9x10^-24^ | -41381 | 0.653 | rs9274196 | 481 | *XXbac-BPG254F23.6* | 3.2x10^-20^ | 0.617 | Adipose_Subcutaneous | 23715323 |
| Bite size | 6p21.32 | 32672089 | rs3134995 | 1.9x10^-24^ | -40306 | 0.624 | rs9274284 | 0 | *HLA-DQB1* | 1.5x10^-18^ | 0.676 | Esophagus_Muscularis | 23715323 |
| Bite size | 6p21.32 | 32672089 | rs3134995 | 1.9x10^-24^ | -12774 | 0.547 | rs141126313 | 29088 | *XXbac-BPG254F23.6* | 2.2x10^-18^ | 0.503 | Whole_Blood | 23715323 |
| Bite size | 6p21.32 | 32672089 | rs3134995 | 1.9x10^-24^ | -36243 | 0.947 | rs9274614 | 0 | *HLA-DQB1* | 1.6x10^-17^ | 0.675 | Heart_Left_Ventricle | 23715323 |
| Bite size | 6p21.32 | 32672089 | rs3134995 | 1.9x10^-24^ | -36998 | 0.724 | rs4311549 | 0 | *HLA-DQB1* | 1.2x10^-16^ | 0.736 | Artery_Aorta | 23715323 |
| Bite size | 6p21.32 | 32672089 | rs3134995 | 1.9x10^-24^ | -36998 | 0.724 | rs4311549 | 4864 | *XXbac-BPG254F23.6* | 2.5x10^-15^ | 0.503 | Thyroid | 23715323 |
| Bite size | 6p21.32 | 32672089 | rs3134995 | 1.9x10^-24^ | -25189 | 0.612 | rs114856834 | 10740 | *HLA-DQB1* | 3.1x10^-14^ | 0.659 | Stomach | 23715323 |
| Bite size | 6p21.32 | 32672089 | rs3134995 | 1.9x10^-24^ | -45007 | 0.615 | rs9273394 | 1050 | *HLA-DQB1-AS1* | 2.3x10^-13^ | 0.634 | Stomach | 23715323 |
| Bite size | 6p21.32 | 32672089 | rs3134995 | 1.9x10^-24^ | -25189 | 0.612 | rs114856834 | 16673 | *XXbac-BPG254F23.6* | 1.2x10^-12^ | 0.396 | Artery_Tibial | 23715323 |
| Bite size | 6p21.32 | 32672089 | rs3134995 | 1.9x10^-24^ | -36243 | 0.947 | rs9274614 | 5619 | *XXbac-BPG254F23.6* | 2.6x10^-10^ | 0.54 | Artery_Aorta | 23715323 |
| Bite size | 6p21.32 | 32672089 | rs3134995 | 1.9x10^-24^ | 69899 | 0.876 | rs3830058 | 7423 | *AL662789.11* | 1.1x10^-9^ |  | Whole_Blood | 24013639 |
| Bite size | 6p21.32 | 32672089 | rs3134995 | 1.9x10^-24^ | -38047 | 0.6 | chr6:32634042:I | 3815 | *XXbac-BPG254F23.6* | 6.3x10^-9^ | 0.407 | Heart_Left_Ventricle | 23715323 |
| Bite size | 6p21.32 | 32672089 | rs3134995 | 1.9x10^-24^ | -393454 | 0.511 | rs9268199 | -447287 | *SLC44A4* | 9.7x10^-5^ | 0.053 | B-Cell | 22446964 |
| Bite size | 7p22.1 | 4769030 | rs7793919 | 2.8x10^-8^ | -17852 | 0.861 | rs7784748 | 26020 | *FOXK1* | 5.6x10^-44^ |  | Whole_Blood | 24013639 |
| Bite size | 7p22.1 | 4769030 | rs7793919 | 2.8x10^-8^ | 15622 | 0.861 | rs7784748 | 0 | *FOXK1* | 3.1x10^-11^ |  | Whole_Blood | 24092820 |
| Bite size | 7p22.1 | 4769030 | rs7793919 | 2.8x10^-8^ | 14176 | 0.627 | rs10232008 | 0 | *FOXK1* | 1.3x10^-7^ | 0.235 | Whole_Blood | 23715323 |
| Bite size | 7p22.1 | 4769030 | rs7793919 | 2.8x10^-8^ | -2647 | 0.691 | rs6944865 | 0 | *FOXK1* | 1.7x10^-6^ | 0.018 | Monocyte | 20502693 |
| Bite size | 9q32 | 117693631 | rs2075533 | 4.7x10^-7^ | -29420 | 0.895 | rs2295800 | 1088 | *TNFSF8* | 2.6x10^-8^ | 0.105 | Monocyte | 22446964 |
| Bite size | 12q13.3 | 57493727 | rs3024971 | 5.9x10^-8^ | -4018 | 1 | rs1059513 | 642107 | *TSPAN31* | 2.2x10^-7^ | 0.212 | Brain White Matter | 25174004 |
| Bite size | 12q13.3 | 57493727 | rs3024971 | 5.9x10^-8^ | -4018 | 1 | rs1059513 | 660089 | *CDK4,TSPAN31* | 5.8x10^-7^ | 0.2 | Brain White Matter | 25174004 |
| Bite size | 12q13.3 | 57493727 | rs3024971 | 5.9x10^-8^ | -4018 | 1 | rs1059513 | 598197 | *OS9* | 8.0x10^-7^ | 0.196 | Brain White Matter | 25174004 |
| Bite size | 12q15 | 68390201 | rs2906856 | 7.9x10^-12^ | 36427 | 0.792 | rs2870960 | 43403 | *GS1-410F4.2* | 5.8x10^-46^ | 0.422 | Lymphoblastoid | 24037378 |
| Bite size | 12q15 | 68390201 | rs2906856 | 7.9x10^-12^ | 23477 | 0.805 | rs2051993 | 0 | *IFNG-AS1* | 3.8x10^-13^ | 0.41 | Artery_Tibial | 23715323 |
| Bite size | 12q15 | 68390201 | rs2906856 | 7.9x10^-12^ | 31421 | 0.792 | rs12228471 | 173509 | *IL26* | 9.7x10^-13^ |  | Whole_Blood | 24092820 |
| Bite size | 12q15 | 68390201 | rs2906856 | 7.9x10^-12^ | 36835 | 0.809 | rs17104682 | 0 | *IFNG-AS1* | 1.0x10^-11^ | 0.353 | Whole_Blood | 23715323 |
| Bite size | 12q15 | 68390201 | rs2906856 | 7.9x10^-12^ | 23477 | 0.805 | rs2051993 | 0 | *IFNG-AS1* | 1.2x10^-8^ | 0.273 | Muscle_Skeletal | 23715323 |
| Bite size | 12q15 | 68390201 | rs2906856 | 7.9x10^-12^ | 9877 | 0.851 | rs12582272 | 801831 | *MDM2* | 9.9x10^-7^ | 0.193 | Brain White Matter | 25174004 |
| Bite size | 22q12.3 | 37319589 | rs5750339 | 9.2x10^-21^ | -21245 | 0.641 | rs5756391 | -26299 | *NCF4* | 7.6x10-5 | 0.055 | B-Cell | 22446964 |
| Itch intensity (unadjusted by bite size) | 1q32.1 | 206937245 | rs6673928 | 6.4×10^−8^ | 4284 | 0.993 | rs3024498 | 30686 | *IL19* | 1.5×10^−16^ | 0.168 | Lymphoblastoid | 24037378 |
| Itch intensity adjusted by bite size | 5q31.1 | 131811182 | rs11741255 | 3.0×10^−22^ | −94132 | 0.653 | rs17622208 | 13932 | *SLC22A5* | 1.4×10^−11^ | 0.151 | B-Cell | 22446964 |
| Itch intensity adjusted by bite size | 5q31.1 | 131811182 | rs11741255 | 3.0×10^−22^ | −247681 | 0.541 | rs113823725 | 0 | *P4HA2* | 1.6×10^−12^ | 0.45 | Thyroid | 23715323 |
| Itch intensity adjusted by bite size | 5q31.1 | 131811182 | rs11741255 | 3.0×10^−22^ | −402340 | 0.56 | rs1469149 | 267437 | *SLC22A4* | 1.7×10^−7^ | 0.093 | Monocyte | 22446964 |
| Itch intensity adjusted by bite size | 5q31.1 | 131811182 | rs11741255 | 3.0×10^−22^ | −103753 | 0.641 | rs2631360 | 0 | *SLC22A5* | 3.6×10^−8^ | 0.255 | Whole Blood | 23715323 |
| Itch intensity adjusted by bite size | 5q31.1 | 131811182 | rs11741255 | 3.0×10^−22^ | −94132 | 0.653 | rs17622208 | 13932 | *SLC22A5* | 4.3×10^−37^ | 0.44 | Monocyte | 22446964 |
| Itch intensity adjusted by bite size | 5q31.1 | 131811182 | rs11741255 | 3.0×10^−22^ | −219457 | 0.521 | rs112537099 | 39283 | *P4HA2* | 4.4×10^−8^ | 0.078 | Lymphoblastoid | 24037378 |
| Itch intensity adjusted by bite size | 5q31.1 | 131811182 | rs11741255 | 3.0×10^−22^ | −219457 | 0.521 | rs112537099 | 63224 | *P4HA2-AS1* | 4.6×10^−11^ | 0.405 | Thyroid | 23715323 |
| Itch intensity adjusted by bite size | 5q31.1 | 131811182 | rs11741255 | 3.0×10^−22^ | −246681 | 0.515 | rs111547866 | 35240 | *AC063976.6* | 5.0×10^−7^ | 0.261 | Thyroid | 23715323 |
| Itch intensity adjusted by bite size | 5q31.1 | 131811182 | rs11741255 | 3.0×10^−22^ | −103753 | 0.641 | rs2631360 | 0 | *SLC22A5* | 6.7×10^−85^ | 0.23 | Monocyte | 20502693 |
| Itch intensity adjusted by bite size | 22q12.3 | 37298344 | rs5756391 | 1.4×10^−8^ | 0 | 1 | rs5756391 | −26299 | *NCF4* | 7.6×10^−5^ | 0.055 | B-Cell | 22446964 |
| Itch intensity adjusted by bite size | 22q12.3 | 37298344 | rs5756391 | 5.5×10^−7^ | 0 | 1 | rs5756391 | −26299 | *NCF4* | 7.6×10^−5^ | 0.055 | B-Cell | 22446964 |
| Attractiveness | 5q31.1 | 131371999 | rs1858074 | 2.4x10^-9^ | 36078 | 0.578 | rs2069616 | 322905 | *SLC22A5* | 2.4x10^-20^ | 0.263 | Monocyte | 22446964 |
| Attractiveness | 5q31.1 | 131371999 | rs1858074 | 2.4x10^-9^ | 36843 | 0.663 | rs1469149 | 267437 | *SLC22A4* | 1.7x10^-7^ | 0.093 | Monocyte | 22446964 |
| Attractiveness | 5q31.1 | 131371999 | rs1858074 | 2.4x10^-9^ | 36078 | 0.578 | rs2069616 | 322905 | *SLC22A5* | 5.8x10^-7^ | 0.086 | B-cell | 22446964 |
| Attractiveness | 6p21.32 | 32410941 | rs9268659 | 3.5x10^-9^ | 1598 | 0.802 | rs7195 | 0 | *HLA-DRA* | 1.4x10^-26^ | 0.077 | Monocyte | 20502693 |
| Attractiveness | 6p21.32 | 32410941 | rs9268659 | 3.5x10^-9^ | 109517 | 0.802 | rs7194 | -2616961 | *HLA-G* | 1.0x10^-10^ | 0.81 | Liver | 18462017 |
| Attractiveness | 6p21.32 | 32410941 | rs9268659 | 3.5x10^-9^ | 109517 | 0.802 | rs7194 | -4833 | *HLA-DRA* | 8.3x10^-8^ | 0.81 | Liver | 18462017 |
| Attractiveness | 6p21.32 | 32410941 | rs9268659 | 3.5x10^-9^ | -804 | 0.518 | rs3129883 | -1238600 | *HCG27* | 0.00011 | 0.052 | Monocyte | 22446964 |
| Attractiveness | 6p21.32 | 32410941 | rs9268659 | 3.5x10^-9^ | -190457 | 0.525 | rs3115572 | -525148 | *DDAH2* | 0.00042 | 0.044 | Monocyte | 22446964 |
| Attractiveness | 6p21.32 | 32410941 | rs9268659 | 3.5x10^-9^ | -225 | 0.662 | rs9268658 | -613007 | *HSPA1B* | 0.0005 | 0.042 | Monocyte | 22446964 |
| Attractiveness | 6p21.33 | 31836827 | rs521977 | 5.9x10^-7^ | 90515 | 0.526 | rs440454 | 557857 | *HLA-DRB5* | 1.5x10^-43^ | 0.124 | Monocyte | 20502693 |
| Attractiveness | 6p21.33 | 31836827 | rs521977 | 5.9x10^-7^ | 90515 | 0.526 | rs440454 | 619192 | *HLA-DRB1* | 8.4x10^-14^ | 0.04 | Monocyte | 20502693 |
| Attractiveness | 6p21.33 | 31836827 | rs521977 | 5.9x10^-7^ | 85427 | 0.566 | rs630379 | 626361 | *LOC642073* | 2.0x10^-13^ | 0.176 | B-cell | 22446964 |
| Attractiveness | 6p21.33 | 31836827 | rs521977 | 5.9x10^-7^ | 90515 | 0.526 | rs440454 | 677791 | *HLA-DQA1* | 3.8x10^-11^ | 0.032 | Monocyte | 20502693 |
| Attractiveness | 6p21.33 | 31836827 | rs521977 | 5.9x10^-7^ | 90515 | 0.526 | rs440454 | 235278 | *NOTCH4* | 1.5x10^-8^ | 0.024 | Monocyte | 20502693 |
| Attractiveness | 6p21.33 | 31836827 | rs521977 | 5.9x10^-7^ | 110633 | 0.574 | rs389883 | 710934 | *HLA-C* | 5.1x10^-7^ | 0.294 | Lymphoblastoid | 19644074 |
| Attractiveness | 6p21.33 | 31836827 | rs521977 | 5.9x10^-7^ | 90515 | 0.526 | rs440454 | -1033183 | *VARS2* | 3.2x10^-6^ | 0.075 | B-cell | 22446964 |
| Attractiveness | 6p21.33 | 31836827 | rs521977 | 5.9x10^-7^ | 90515 | 0.526 | rs440454 | 563158 | *HLA-DRB4* | 7.2x10^-6^ | 0.016 | Monocyte | 20502693 |
| Attractiveness | 6p21.33 | 31836827 | rs521977 | 5.9x10^-7^ | 110633 | 0.574 | rs389883 | 710934 | *HLA-C* | 0.00017 | 0.177 | Fibroblast | 19644074 |
| Attractiveness | 6p21.33 | 31836827 | rs521977 | 5.9x10^-7^ | 99841 | 0.566 | rs410851 | -1225449 | *IER3* | 0.00038 | 0.044 | Monocyte | 22446964 |

**Table S25. Expression QTLs near mosquito-trait index SNPs.** Cases are highlighted, by endpoint, where an index SNP is within 500 kb and r2>0.5 with a SNP that has been reported to be an eQTL index SNP (i.e. the strongest SNP associated with gene expression for a particular study, tissue and gene) from the literature. Datasets used for the eQTL lookup are contained in Supplementary Table 34. Mosquito-phenotype association; Dataset, mosquito-associated trait; region, accompanying region, position, index SNP, and significance level; relationship, distance and r^2^ between the mosquito-trait index SNP and the annotation; eQTL annotation, SNP identifier for lead variant that drives eQTL; distance, the absolute distance in base pairs between that SNP and the associated gene; the associated gene; the reported *p* value; r^2^, the proportion of variance in expression explained by the association; tissue, the tissue context that the eQTL was identified in; study ID, PubMed identifier.

A.

**
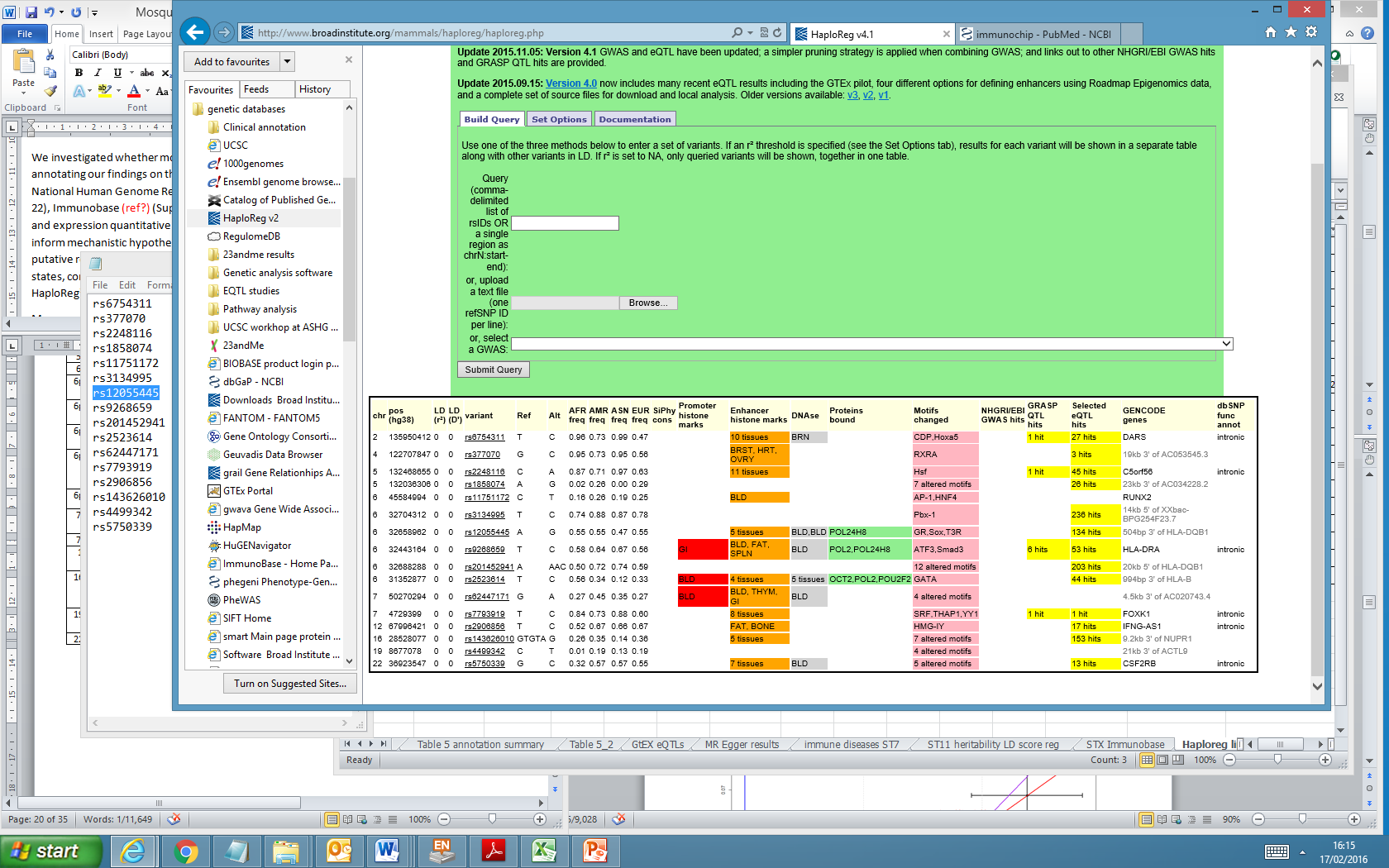
**

B.

| SNP name | Link |
| --- | --- |
| rs6754311 | <http://www.broadinstitute.org/mammals/haploreg/detail_v4.1.php?query=&id=rs6754311> |
| rs377070 | <http://www.broadinstitute.org/mammals/haploreg/detail_v4.1.php?query=&id=rs377070> |
| rs2248116 | <http://www.broadinstitute.org/mammals/haploreg/detail_v4.1.php?query=&id=rs2248116> |
| rs1858074 | <http://www.broadinstitute.org/mammals/haploreg/detail_v4.1.php?query=&id=rs1858074> |
| rs11751172 | <http://www.broadinstitute.org/mammals/haploreg/detail_v4.1.php?query=&id=rs11751172> |
| rs3134995 | <http://www.broadinstitute.org/mammals/haploreg/detail_v4.1.php?query=&id=rs3134995> |
| rs12055445 | <http://www.broadinstitute.org/mammals/haploreg/detail_v4.1.php?query=&id=rs12055445> |
| rs9268659 | <http://www.broadinstitute.org/mammals/haploreg/detail_v4.1.php?query=&id=rs9268659> |
| rs201452941 | <http://www.broadinstitute.org/mammals/haploreg/detail_v4.1.php?query=&id=rs201452941> |
| rs2523614 | <http://www.broadinstitute.org/mammals/haploreg/detail_v4.1.php?query=&id=rs2523614> |
| rs62447171 | <http://www.broadinstitute.org/mammals/haploreg/detail_v4.1.php?query=&id=rs62447171> |
| rs7793919 | <http://www.broadinstitute.org/mammals/haploreg/detail_v4.1.php?query=&id=rs7793919> |
| rs2906856 | <http://www.broadinstitute.org/mammals/haploreg/detail_v4.1.php?query=&id=rs2906856> |
| rs143626010 | <http://www.broadinstitute.org/mammals/haploreg/detail_v4.1.php?query=&id=rs143626010> |
| rs4499342 | <http://www.broadinstitute.org/mammals/haploreg/detail_v4.1.php?query=&id=rs4499342> |
| rs5750339 | <http://www.broadinstitute.org/mammals/haploreg/detail_v4.1.php?query=&id=rs5750339> |

**Table S26. HaploReg functional annotations for mosquito-trait index SNPs.** (A) Screenshot of the summarised HaploReg results for 15 independent GWS mosquito-trait index SNPs (removing tagging SNPs across traits, r^2^>0.5), plus GWS index SNP rs201452941 from female-specific itch intensity GWAS, and (B) links for detailed HaploReg functional annotation.

| Phenotype | Immune Cell Trait | Parent | Grandparent | Wilcoxon 2-sided P-value | Direction of Effect of Mosquito Loci |
| --- | --- | --- | --- | --- | --- |
| Itch Intensity | CM (CD4+ CD8+) AC | CD4+ CD8+ | T lymphocytes | 0.001603886 | Greater Enrichment |
| Itch Intensity | CM CD8+ AC | CD8+ | T lymphocytes | 0.004069204 | Greater Enrichment |
| Itch Intensity | CM CD8br AC | CD8br | T lymphocytes | 0.005088092 | Greater Enrichment |
| Itch Intensity | CM (CD4+ CD8+) %GP | CD4+ CD8+ | T lymphocytes | 0.008555284 | Greater Enrichment |
| Itch Intensity | CD28- CD8dim AC | CD8dim | T lymphocytes | 0.018312556 | Less Enrichment |
| Itch Intensity | CD45RA+ CD28- CD8dim AC | CD8dim | T lymphocytes | 0.036299029 | Less Enrichment |
| Itch Intensity | CM CD8dim %P | CD8dim | T lymphocytes | 0.043584485 | Greater Enrichment |
| Itch Intensity | CD28- CD8dim %GP | CD8dim | T lymphocytes | 0.049868293 | Less Enrichment |
| Bite Size | naive (CD4+ CD8+) %GP | CD4+ CD8+ | T lymphocytes | 0.004965346 | Greater Enrichment |
| Bite Size | CM CD4+ %P | CD4+ | T lymphocytes | 0.00719732 | Greater Enrichment |
| Bite Size | CM CD8+ %GP | CD8+ | T lymphocytes | 0.009345234 | Greater Enrichment |
| Bite Size | TD (CD4- CD8-) %P | CD4- CD8- | T lymphocytes | 0.011447194 | Greater Enrichment |
| Bite Size | CD4+ not Treg AC | CD4+ | T lymphocytes | 0.013022973 | Greater Enrichment |
| Bite Size | CD28- CD8dim %P | CD8dim | T lymphocytes | 0.015342181 | Less Enrichment |
| Bite Size | CD28+ CD8dim %P | CD8dim | T lymphocytes | 0.018438265 | Less Enrichment |
| Bite Size | CD28- CD8dim %GP | CD8dim | T lymphocytes | 0.019128357 | Less Enrichment |
| Bite Size | CM CD8br %GP | CD8br | T lymphocytes | 0.025055759 | Greater Enrichment |
| Bite Size | naive (CD4+ CD8+) AC | CD4+ CD8+ | T lymphocytes | 0.028181134 | Greater Enrichment |
| Bite Size | CD28- CD8dim AC | CD8dim | T lymphocytes | 0.037151081 | Less Enrichment |
| Bite Size | TD CD4+ %P | CD4+ | T lymphocytes | 0.040043712 | Less Enrichment |
| Bite Size | CM CD8+ AC | CD8+ | T lymphocytes | 0.040608876 | Greater Enrichment |
| Bite Size | CM CD4+ %GP | CD4+ | T lymphocytes | 0.042053317 | Greater Enrichment |
| Bite Size | naive CD8+ AC | CD8+ | T lymphocytes | 0.04285704 | Greater Enrichment |
| Bite Size | TD CD4+ AC | CD4+ | T lymphocytes | 0.042953613 | Less Enrichment |
| Bite Size | EM CD8dim %GP | CD8dim | T lymphocytes | 0.049679501 | Greater Enrichment |
| Attractiveness | CM (CD4- CD8-) %P | CD4- CD8- | T lymphocytes | 0.000652671 | Greater Enrichment |
| Attractiveness | CD28+ CD8br AC | CD8br | T lymphocytes | 0.003332127 | Greater Enrichment |
| Attractiveness | CD4+ Treg %P | CD4+ | T lymphocytes | 0.011475136 | Greater Enrichment |
| Attractiveness | CD4+ not Treg %P | CD4+ | T lymphocytes | 0.011531931 | Greater Enrichment |
| Attractiveness | naive CD8br %P | CD8br | T lymphocytes | 0.027429504 | Greater Enrichment |
| Attractiveness | naive CD8+ %P | CD8+ | T lymphocytes | 0.028533381 | Greater Enrichment |
| Attractiveness | CM (CD4- CD8-) %GP | CD4- CD8- | T lymphocytes | 0.031245949 | Greater Enrichment |
| Attractiveness | EM (CD4+ CD8+) AC | CD4+ CD8+ | T lymphocytes | 0.033010318 | Greater Enrichment |
| Attractiveness | CD28+ CD8br %GP | CD8br | T lymphocytes | 0.033157209 | Greater Enrichment |
| Attractiveness | CD45RA- (CD4- CD8-) AC | CD4- CD8- | T lymphocytes | 0.035570329 | Greater Enrichment |
| Attractiveness | EM (CD4- CD8-) AC | CD4- CD8- | T lymphocytes | 0.038561377 | Greater Enrichment |
| Attractiveness | CD45RA- (CD4+ CD8+) AC | CD4+ CD8+ | T lymphocytes | 0.040237524 | Greater Enrichment |

**Table S28. Enrichment of GWAS loci explaining variation in T lymphocyte cell subtypes.** Wilcoxon *P*-values and direction of effect testing for enrichment of mosquito trait SNPs in immune cell subset. EM = Effector Memory, CM=Central Memory, TD= Terminally Differentiated , %P = Percentage of parent cell lineage, %GP = Percentage of grandparent cell lineage, AC=Absolute Count, br=Bright.

**Table S29. Comparison of effect size and P values between traits for index significant SNPs of each trait (mosquito bite size variation, itch intensity from mosquito bites and perceived attractiveness to mosquitos).** The summary statistics for the top hits of each trait are highlighted in gold, and the corresponding summary statistics for the other two traits are shown in adjacent columns as labelled. Region, cytogenetic band; chr, chromosome; position, build 37 map position of the SNP; alleles A and B are assigned based on their alphabetical order; BAF, B allele frequency across all study participants; gene context, gene(s) spanning or flanking (<1Mb away from) the index SNP: brackets indicate the position of the SNP, and dashes indicate distance to a flanking gene (-, >1 kb;  ̶ , >10kb;  ̶ ̶ , >100kb); effect size, magnitude of effect for the B allele; CI, confidence interval; ; *P,* λ adjusted significance level; N/A, SNP data not available

**Supplementary Note**

**23andMe Surveys and Scoring Logic**

The following section shows excerpts from the surveys used to define quantitative analysis or cases and controls for each mosquito-related trait in the 23andMe cohort.

***Survey: Mosquito bite size***

[Q1] When you get bitten by a mosquito, how big do the bites usually get? (research participants answered one of the following):

- Much less than 1/4 inch (much less than the width of a pencil)
- Around 1/4 inch (about the width of a pencil)
- Around 1/2 inch (about the width of a plain M&M)
- Around 3/4 inch (about the width of an American dime)
- Around 1 inch (about the width of an American quarter)
- Much more than an inch (much more than the width of an American quarter)
- I’m not sure

***Survey: Mosquito itch intensity***

[Q2] When you are bitten by mosquitos, how much do the bites typically itch? (research participants answered one of the following):

- Very badly (impossible to ignore)
- Somewhat badly (definitely noticeable, at times hard to ignore)
- Only mildly (noticeably itching, but easy to ignore)
- Not at all (no noticeable itching)
- I’m not sure

***Survey: Mosquito attractiveness***

**[Q3]** When you are exposed to mosquitoes, how much do you get bitten? (research participants answered one of the following):

- Less than the people around me
- More than the people around me
- I’m not sure

**Logic for genetic analysis on 23andMe self-reported questions, quantitative analysis and selecting cases and controls**

***Mosquito bite size***

- Research participant responses to question [Q1] were analysed quantitatively where bite size was scored from small in size to bigger in size (positive direction), excluding responders selecting ‘I’m not sure.’

***Mosquito itch intensity***

- Research participant responses to question [Q2] were analysed quantitatively, where itch intensity was scored from very intense itch to no itch (negative direction), excluding responders selecting ‘I’m not sure.’

***Mosquito attractiveness***

- Cases were defined as those answering positive to ‘Less than the people around me’ to question [Q3], and controls were defined as those answering positive to ‘More than the people around me.’

**Regional plots**

**Regional plots of mosquito bite size associations.** Symbol colours indicate LD with the index SNP, which is labeled and coloured purple. Open circles indicate imputed variants, filled circles indicate partially genotyped variants, and filled squares indicate fully genotyped variants. Plots were generated using LocusZoom (1). Results are in NCBI Build 37 coordinates.

1. Regional plot for SNP rs2248116, close to *IRF1*


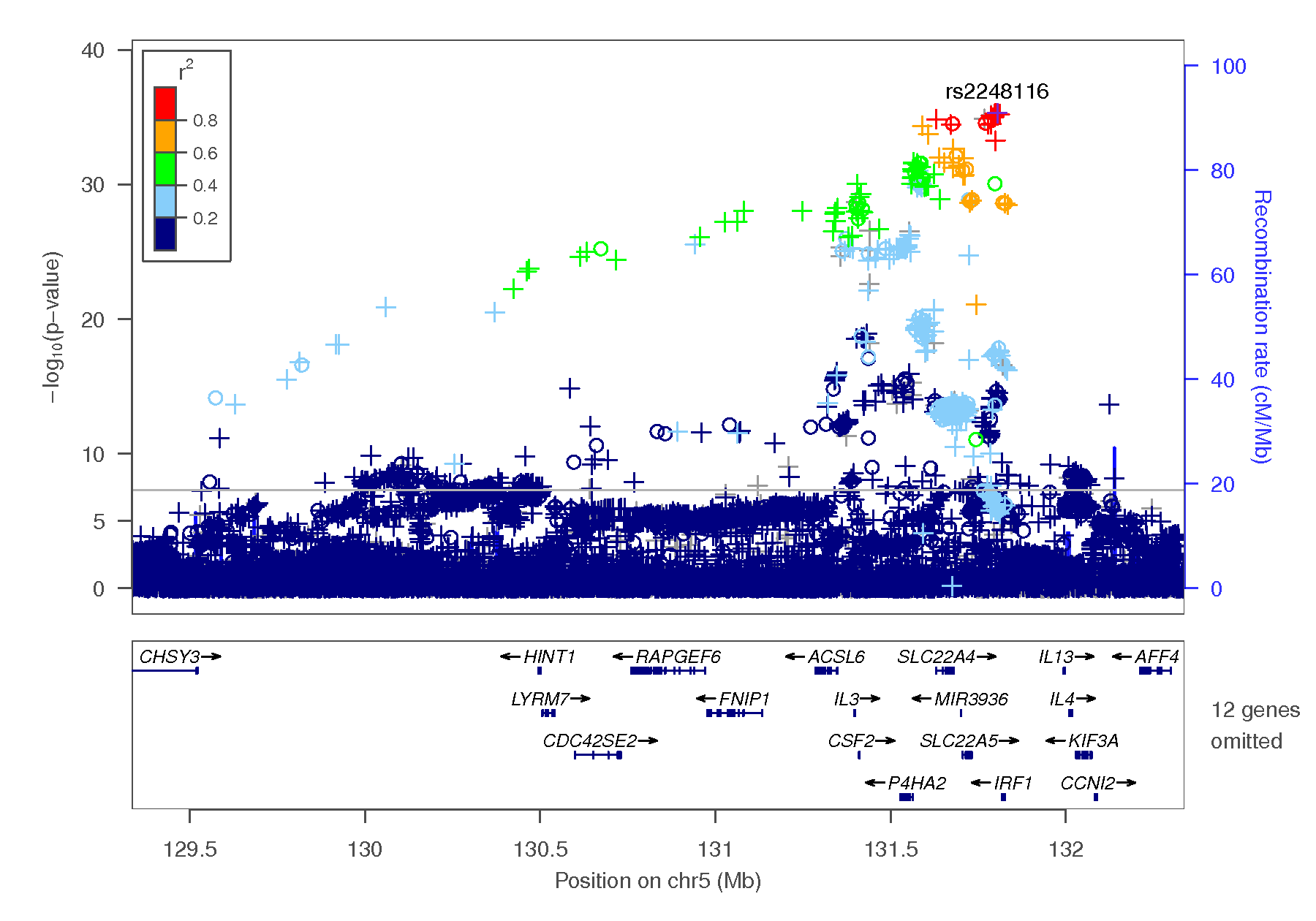


1. Regional plot for SNP rs309392, close to *IL21*


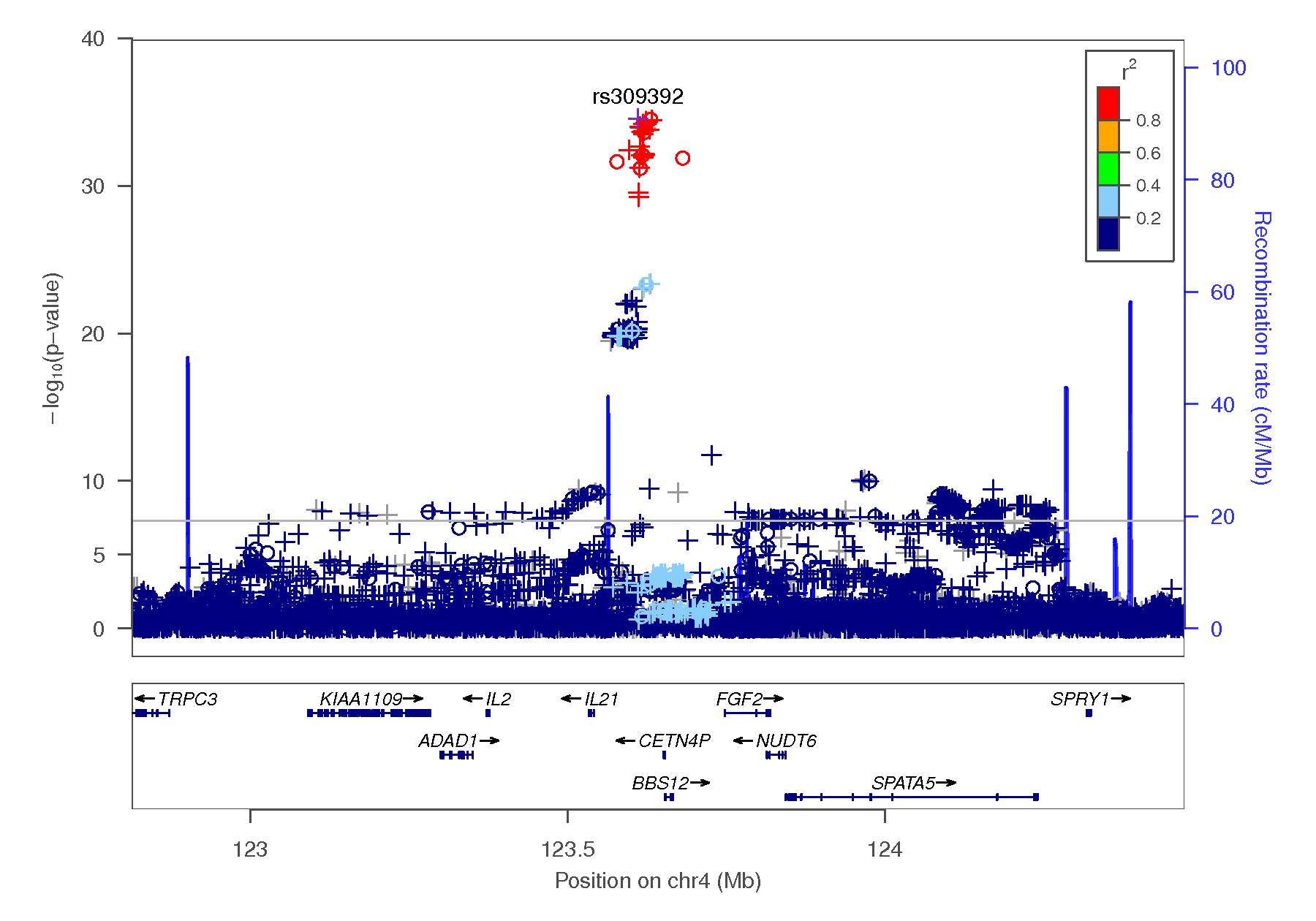


1. Regional plot for SNP rs1548306, close to *HLA-DRA*


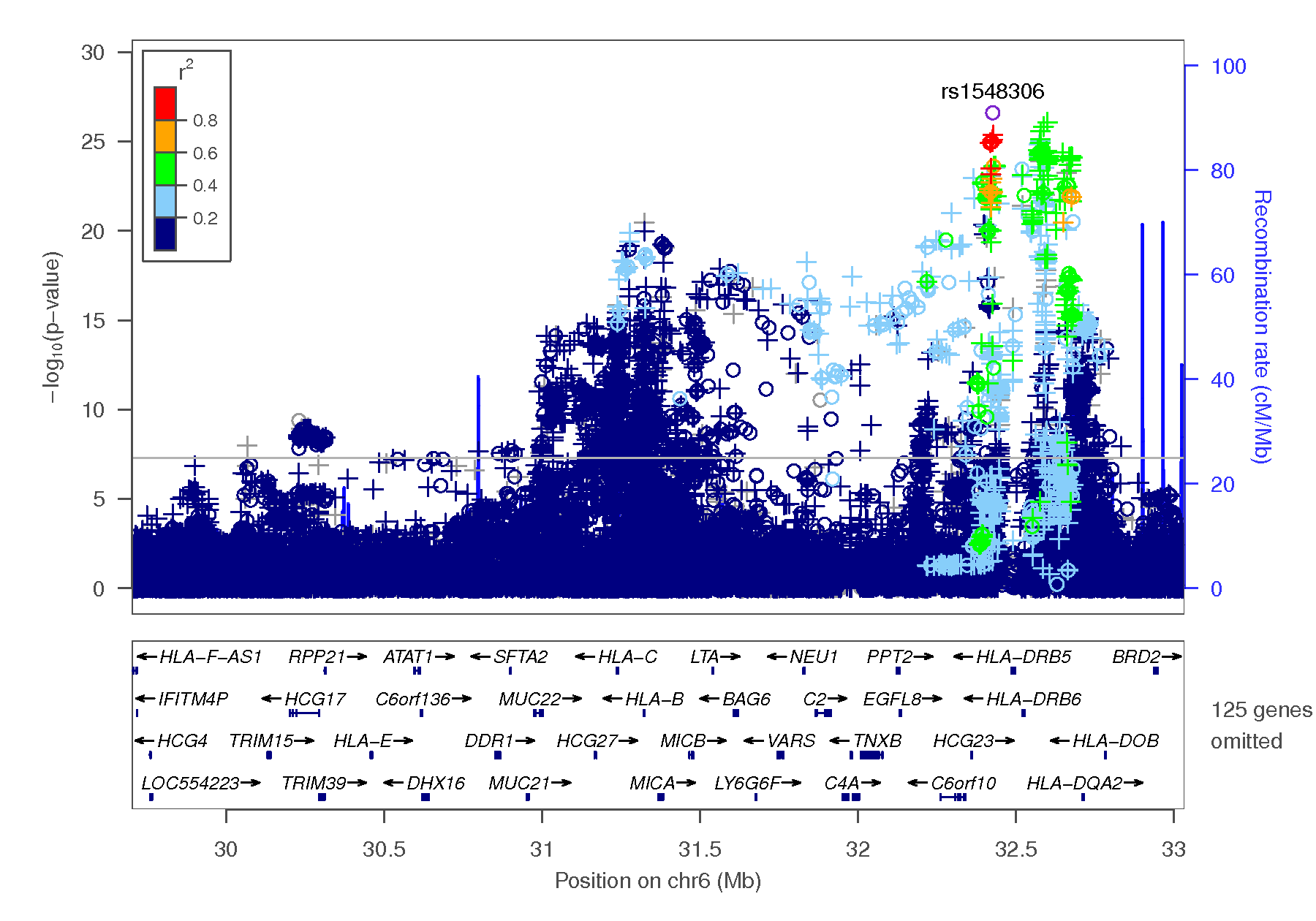


1. Regional plot for rs5750339, co-localising with *CSF2RB*


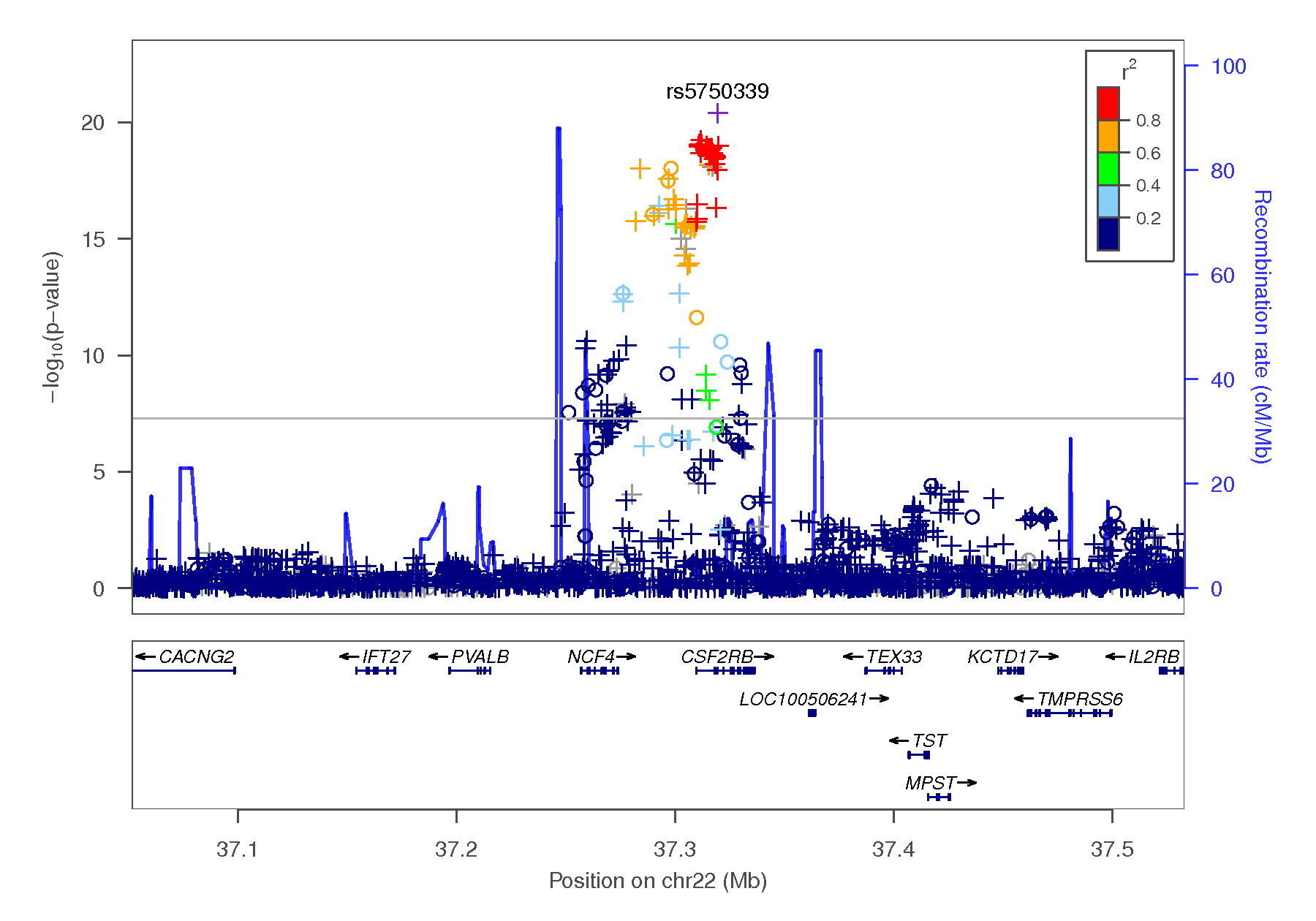


1. Regional plot of rs12832728, close to *IFNG*


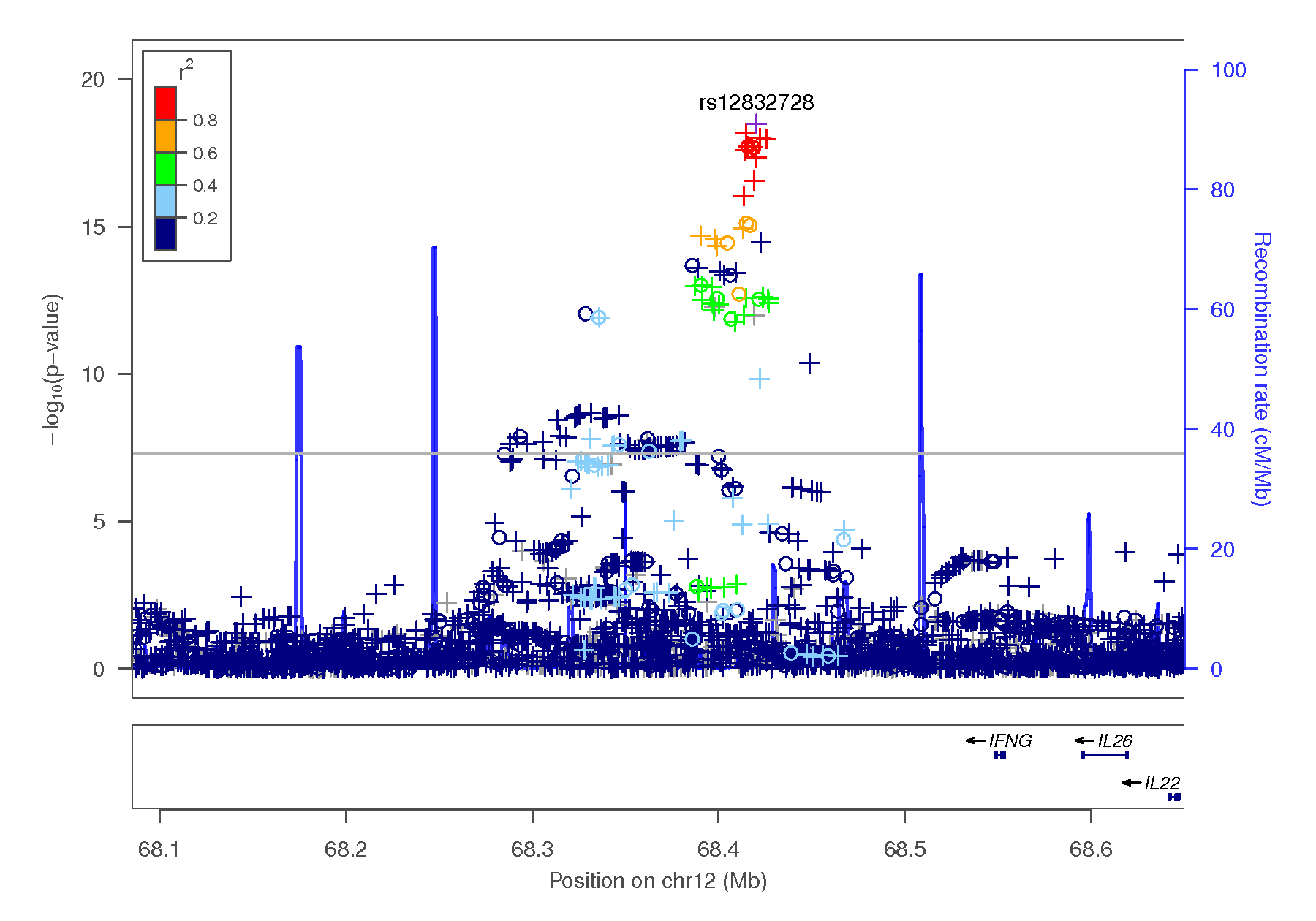


1. Regional plot of rs778798, close to *NRTN*


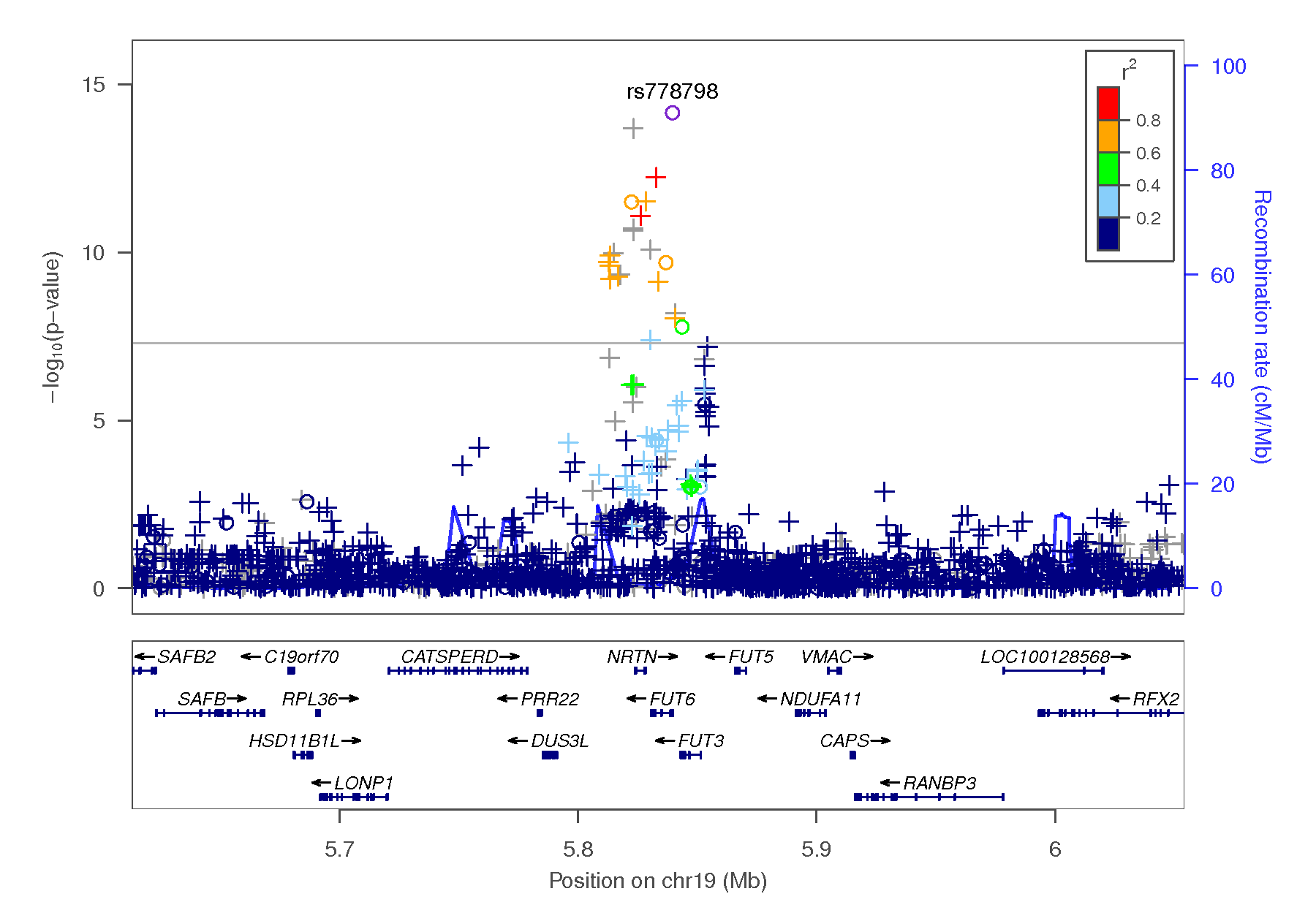


1. Regional plot of rs2967678, close to *ACTL9*


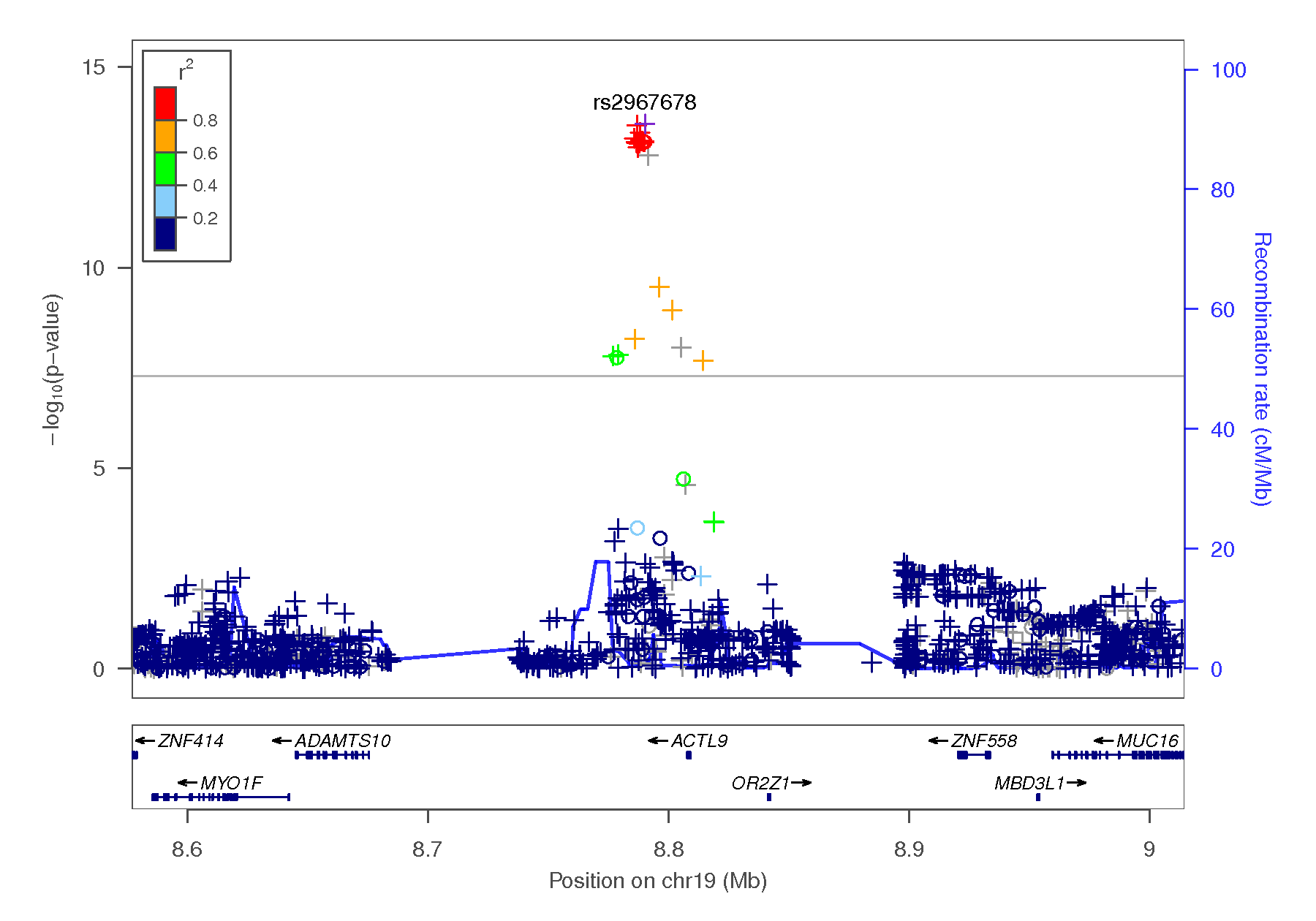


1. Regional plot of rs324014, close to *STAT6*


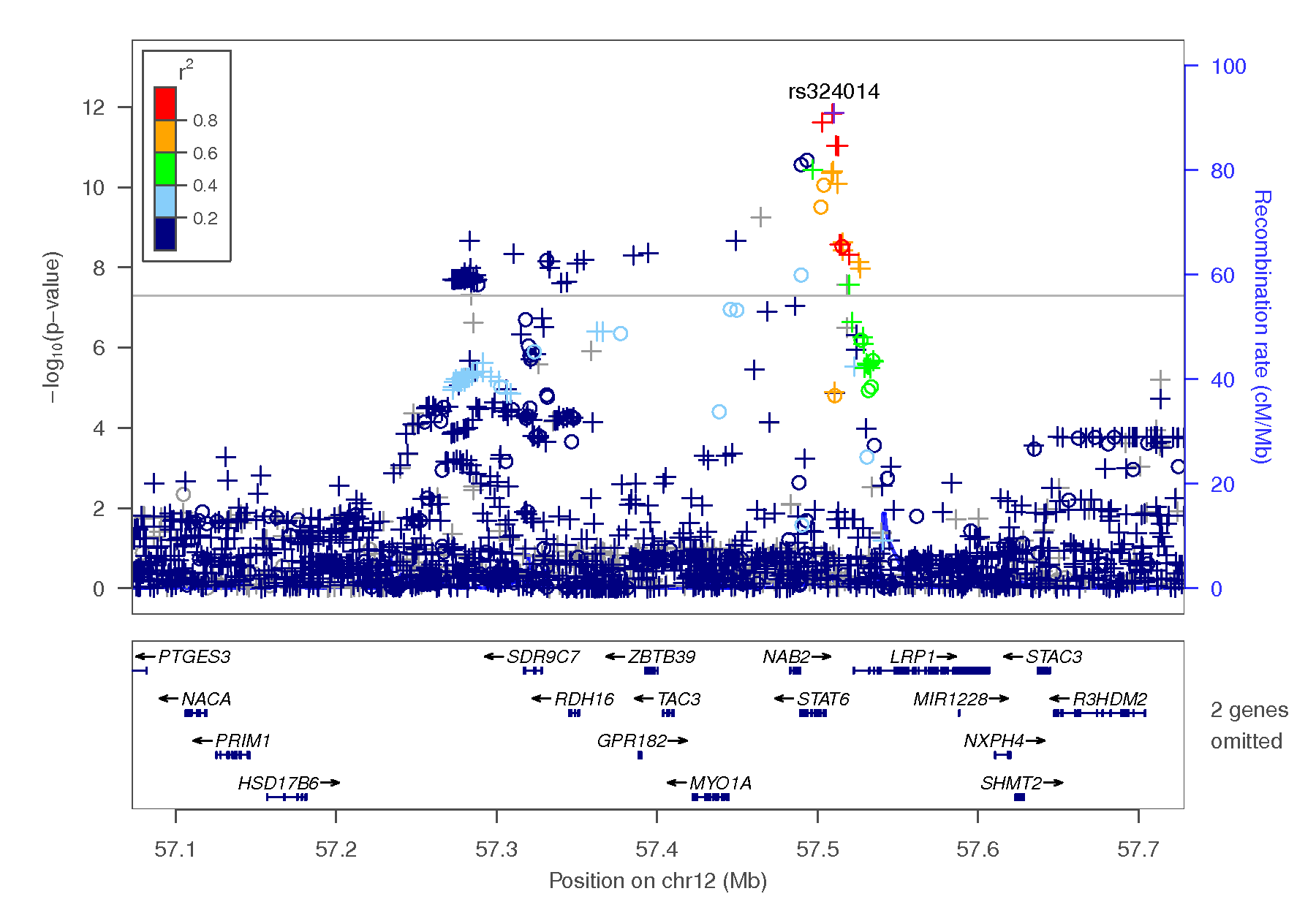


1. Regional plot of rs2085423, close to *IKZF1*


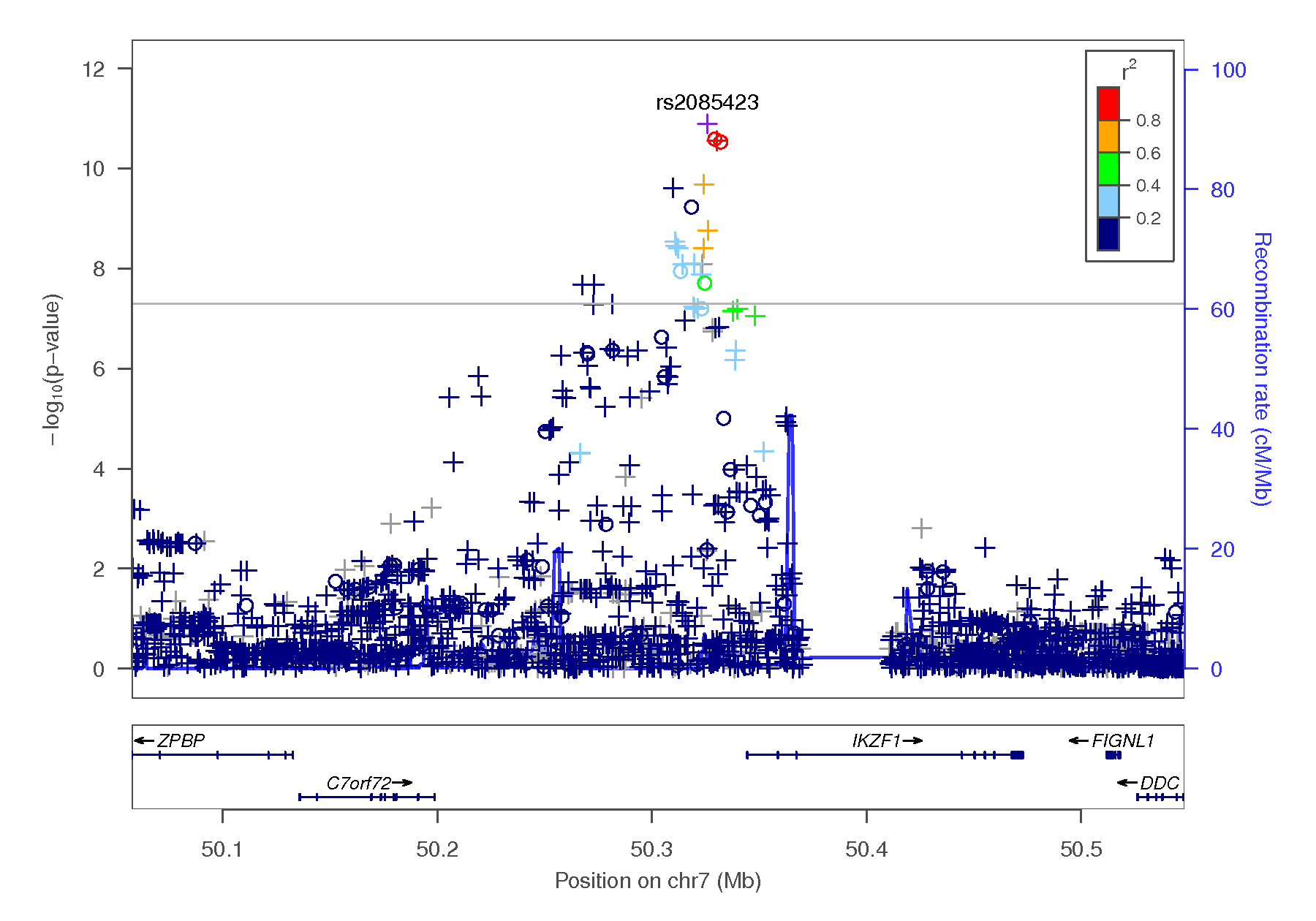


1. Regional plot of rs3111414, co-localising with *LINC00299*


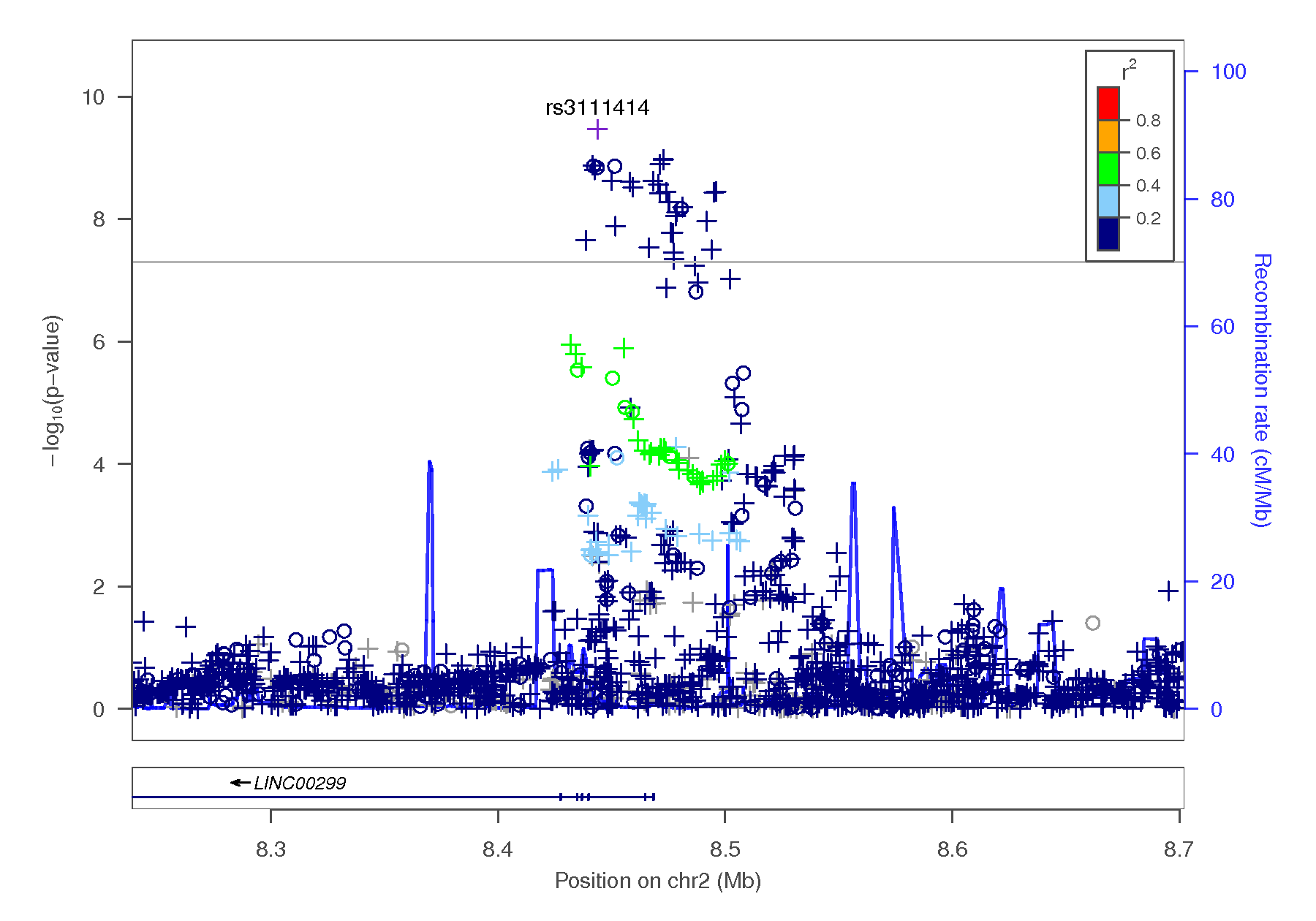


1. Regional plot of rs11751172, close to *RUNX2*


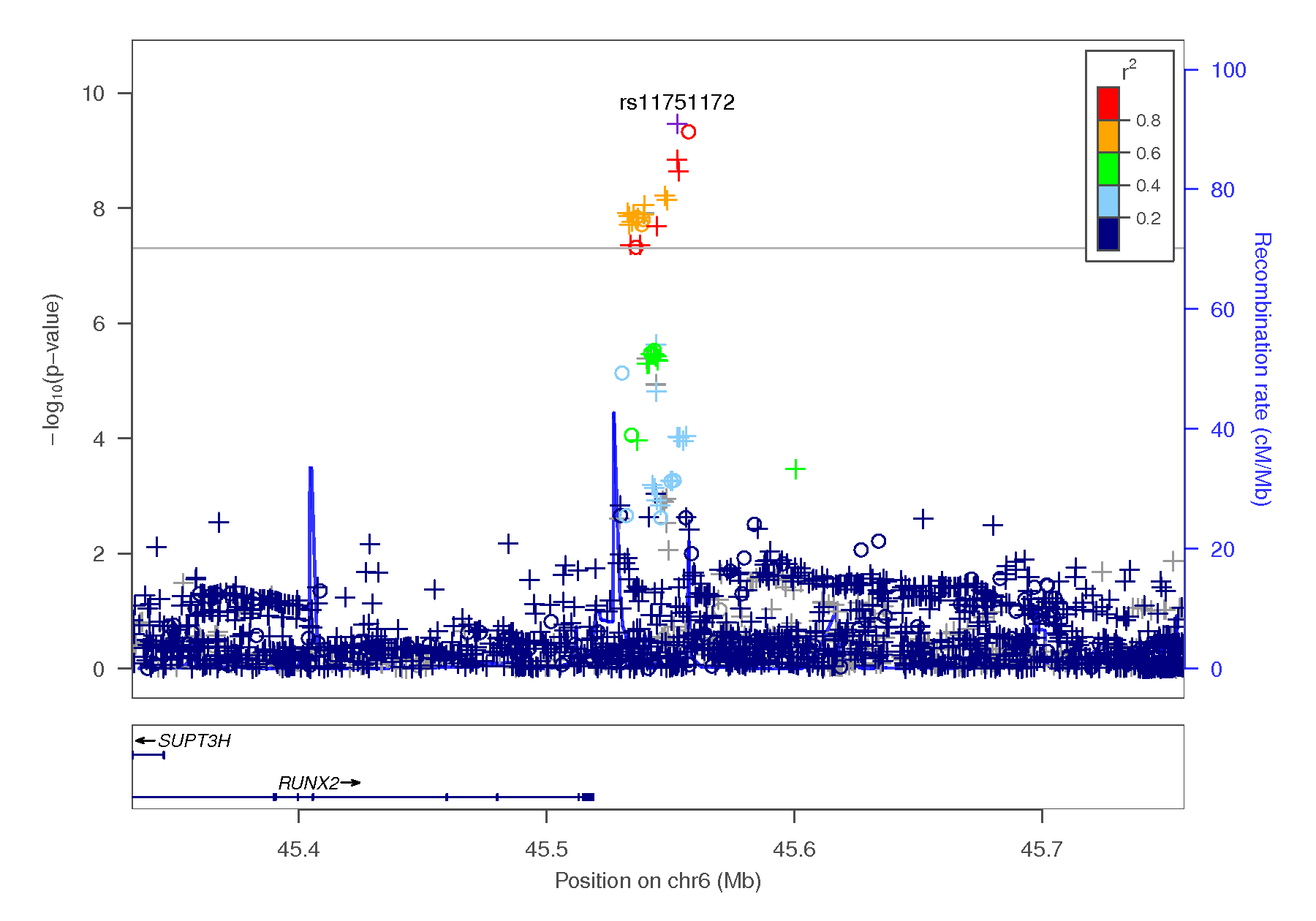


1. Regional plot of rs12949918, co-localising with *STAT3*


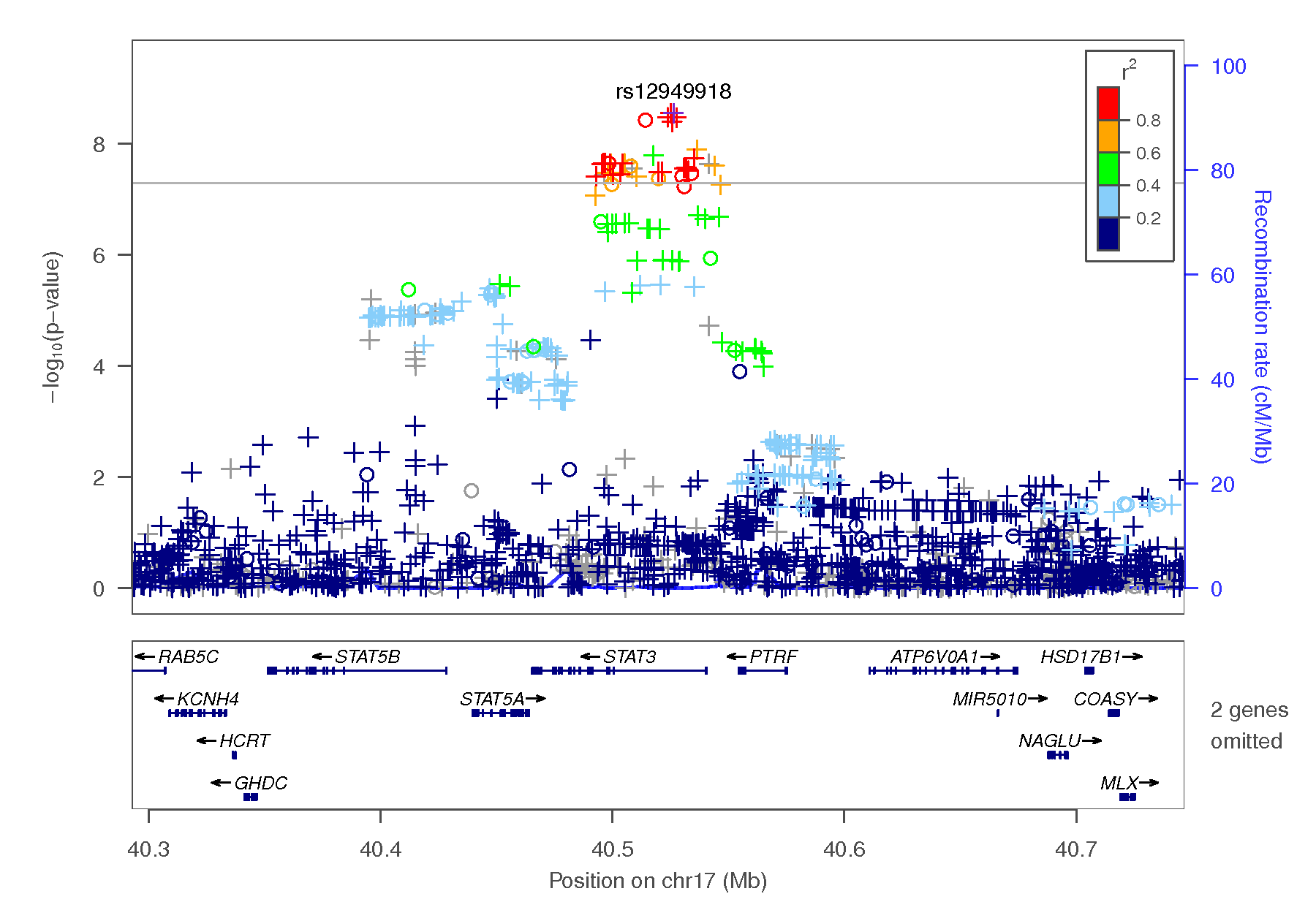


1. Regional plot of rs6809854, close to *SATB1*


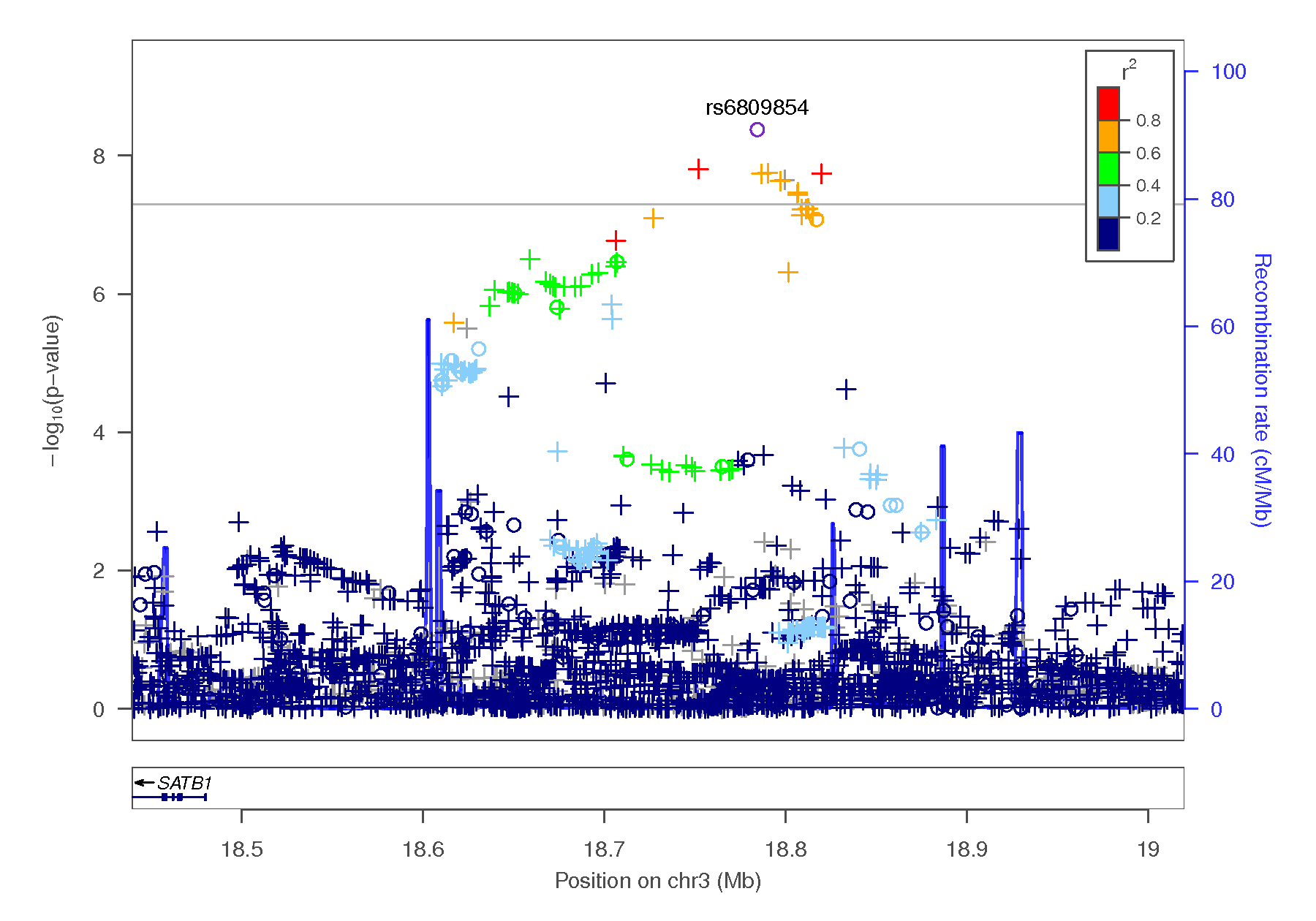


1. Regional plot of rs149082847, close to *LRRC32*


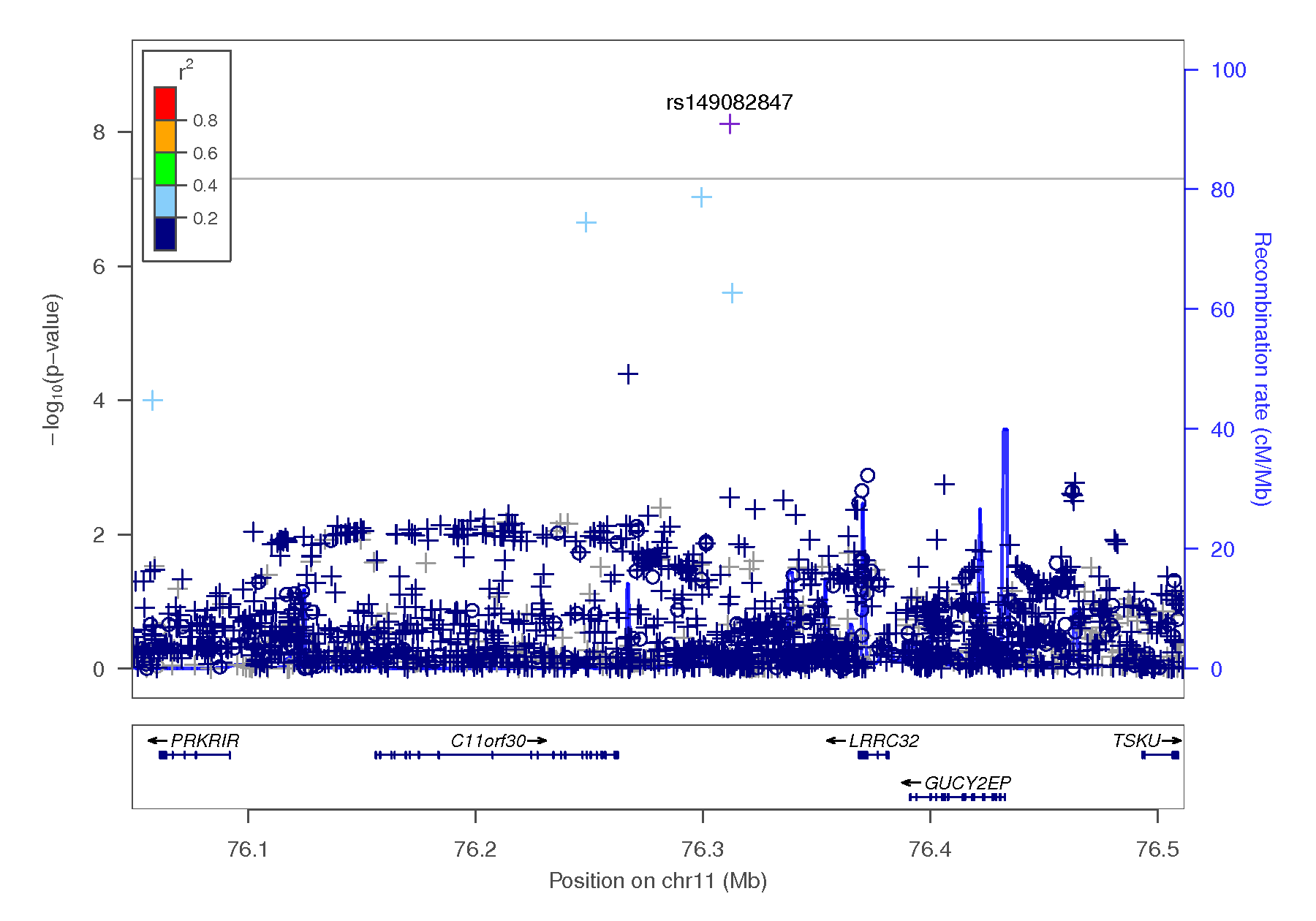


1. Regional plot of rs12133641, co-localising with *IL6R*


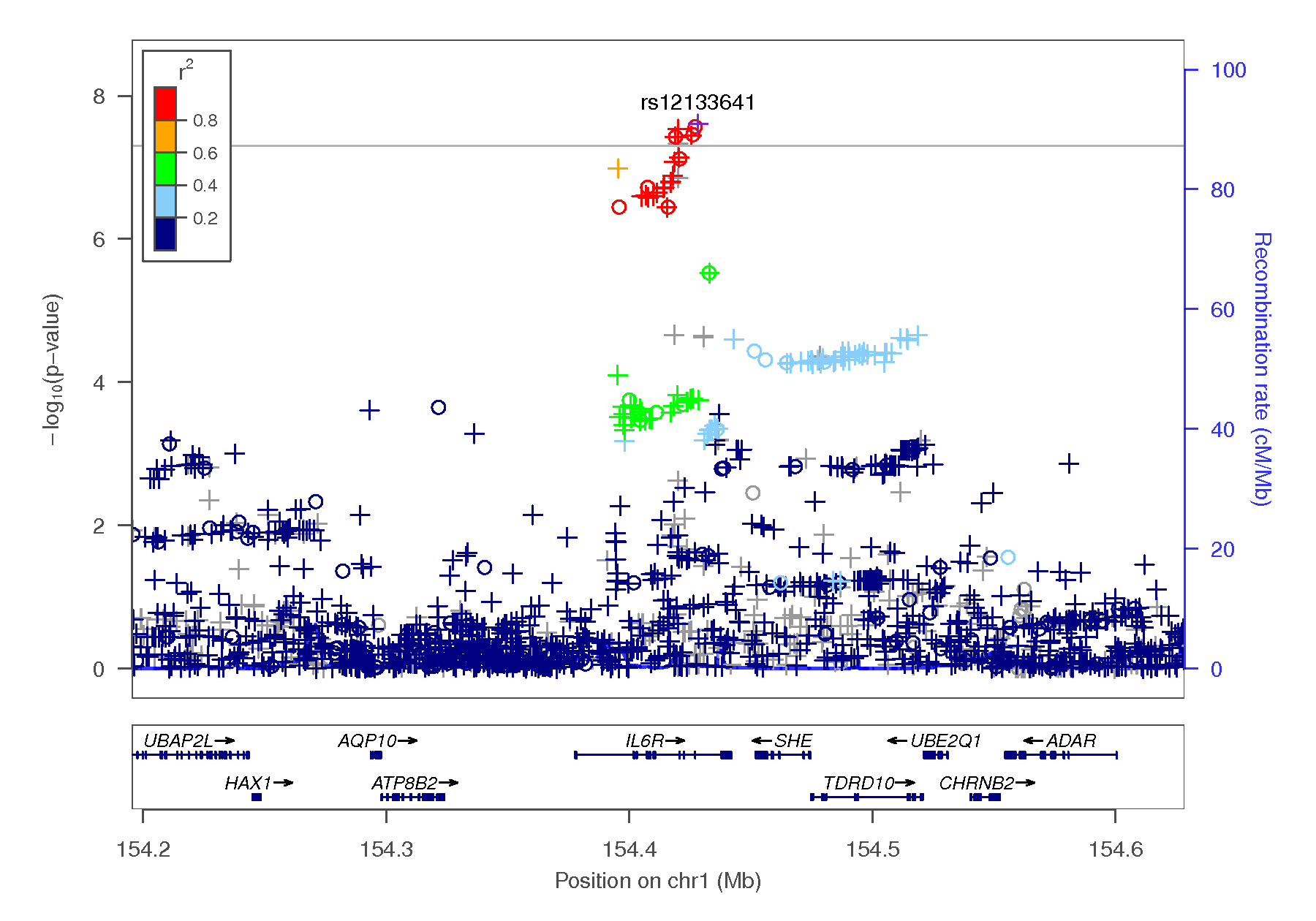


1. Regional plot of rs2282718, co-localising with *RUNX3*


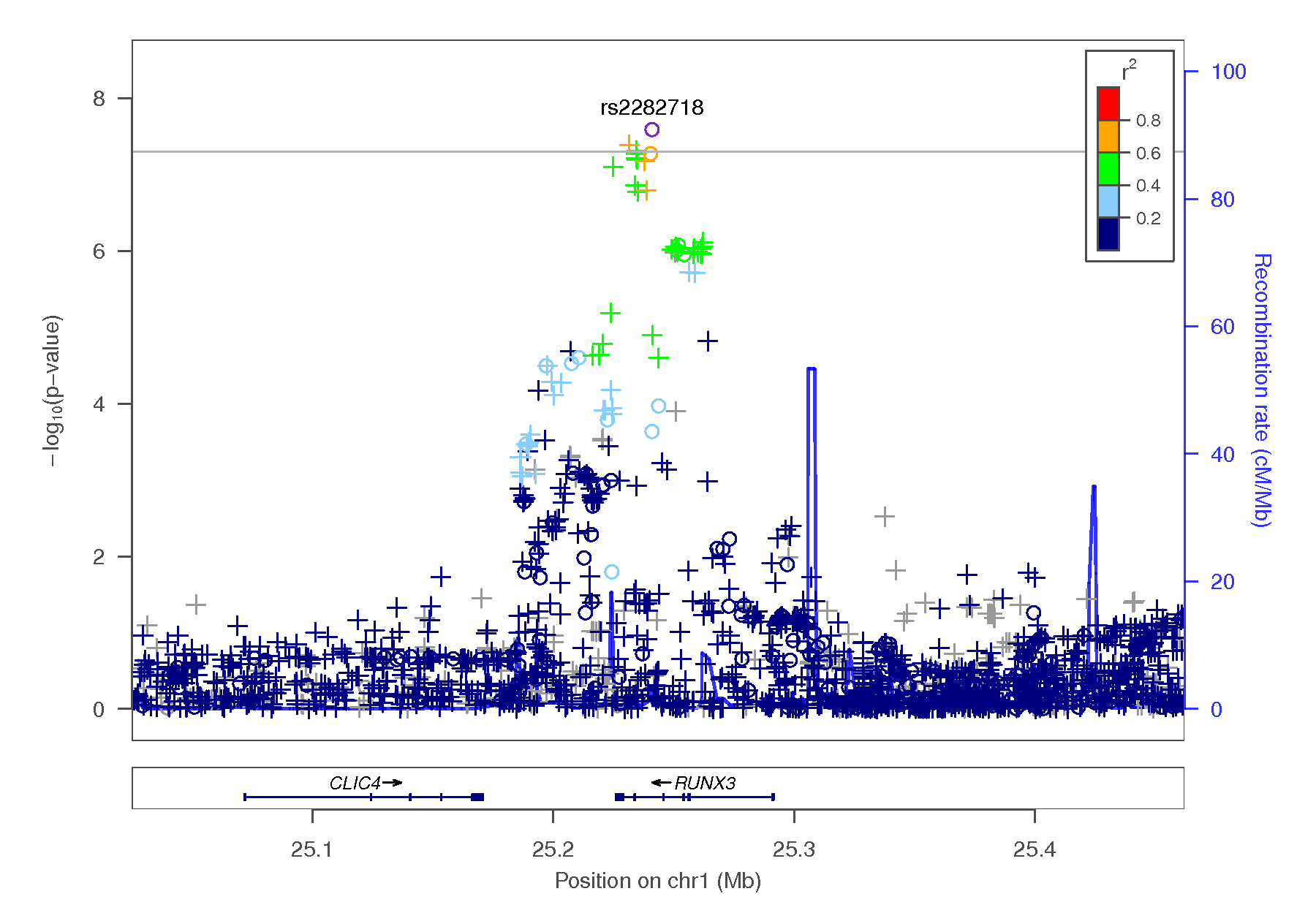


1. Regional plot of rs10261971, co-localising with *FOXK1*


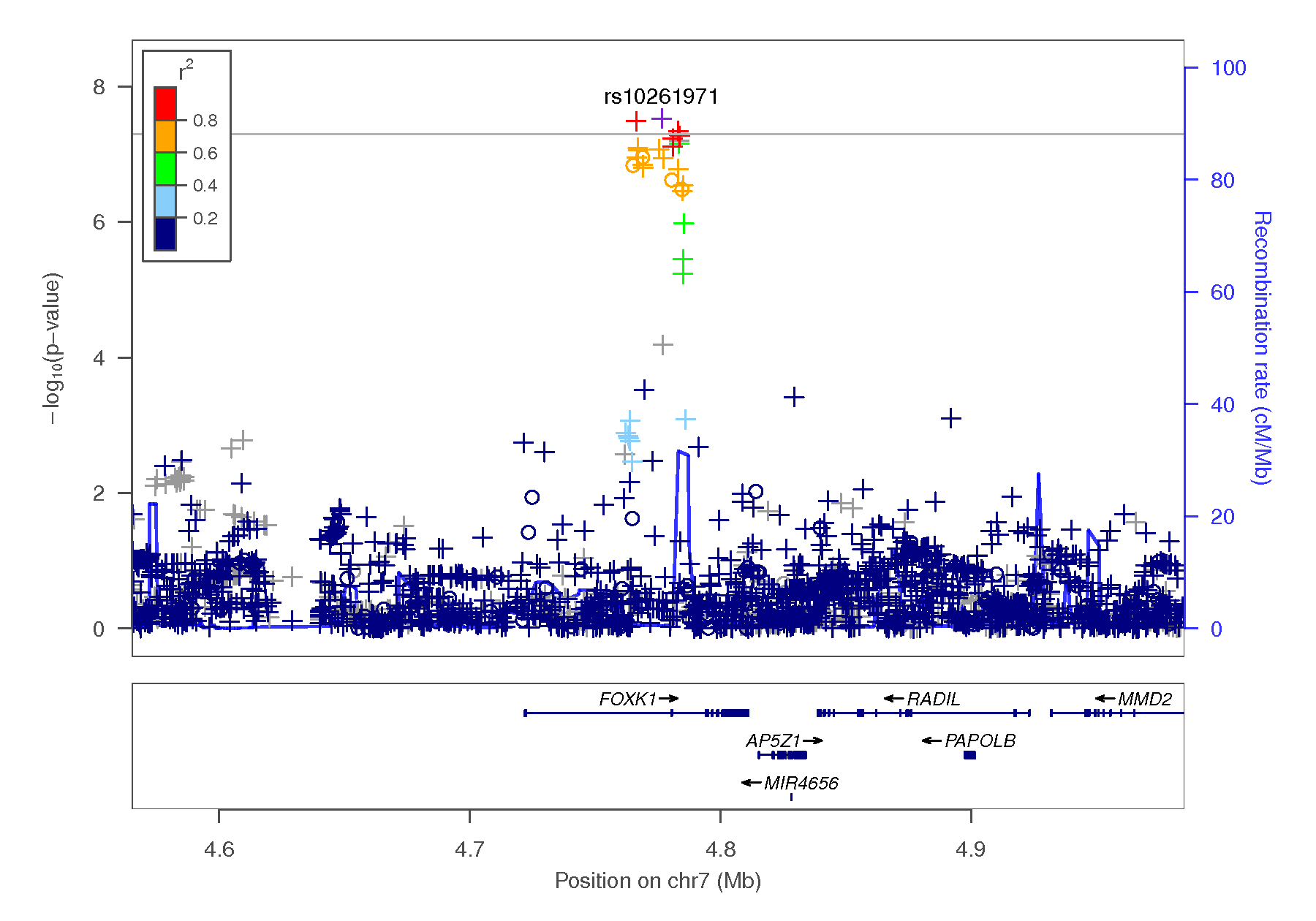


1. Regional plot of rs13079741, co-localising with *LPP*


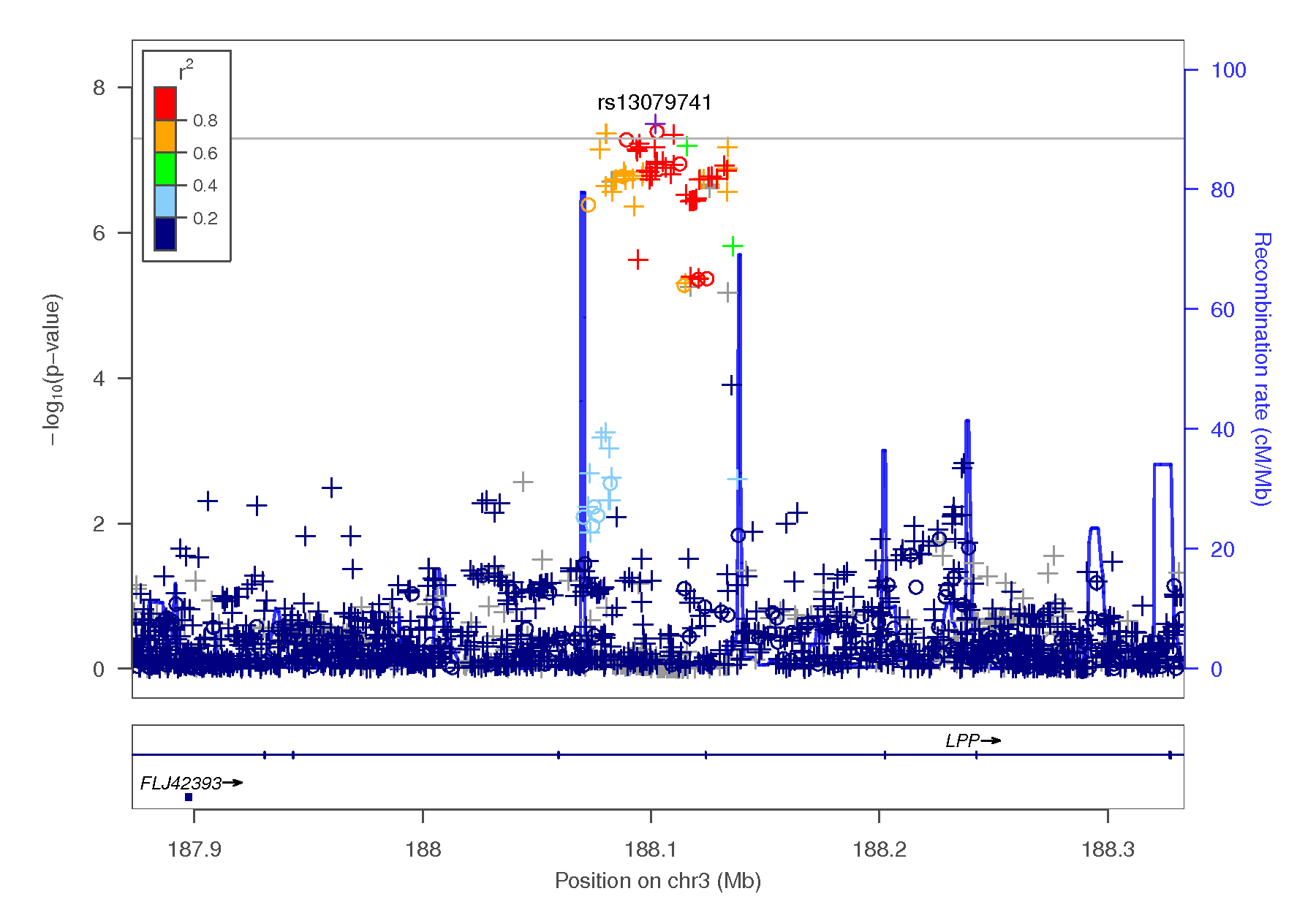


1. Regional plot of rs12125543, co-localising with *SOX13*


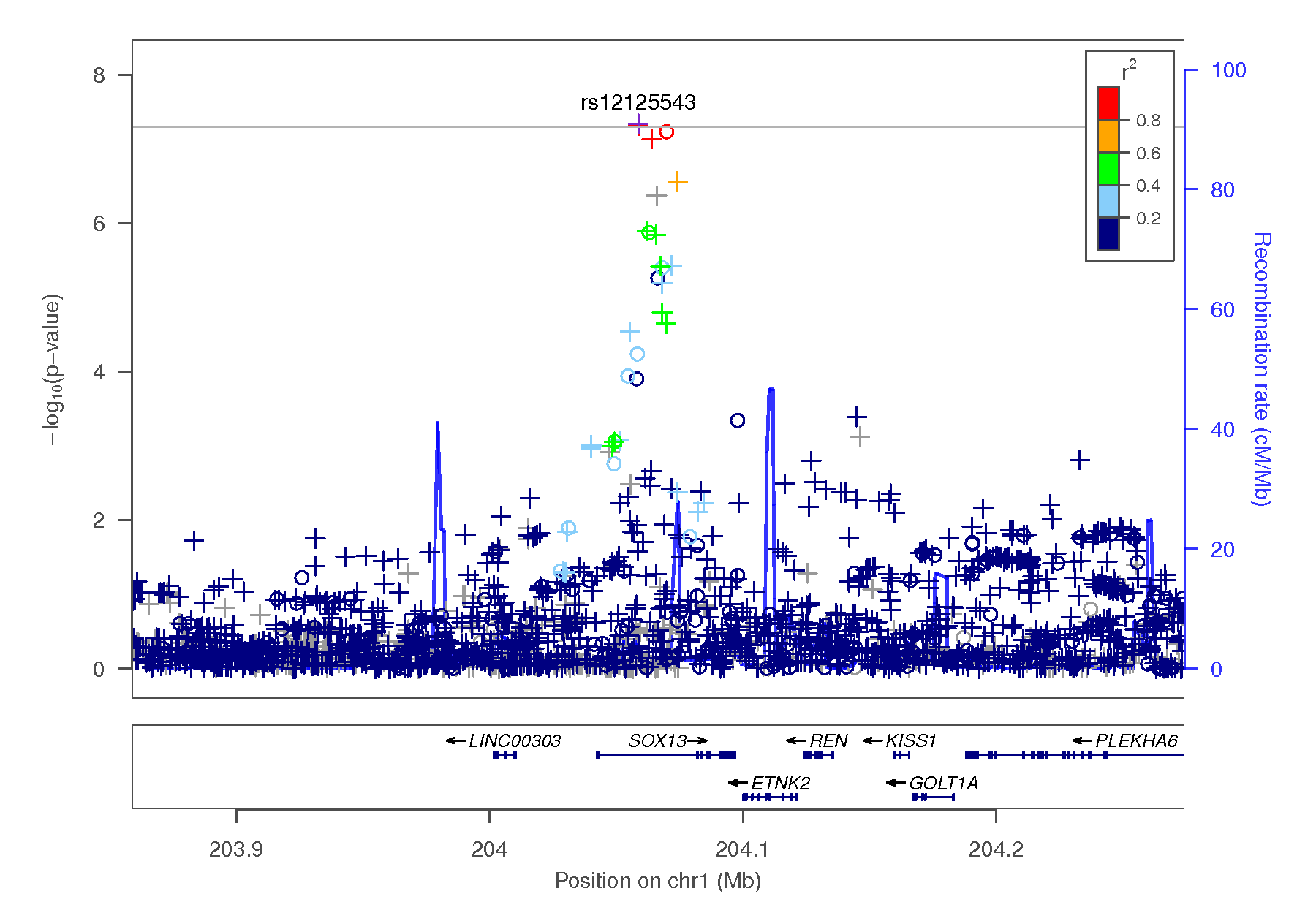


1. Regional plot of rs6673928, close to *IL10*


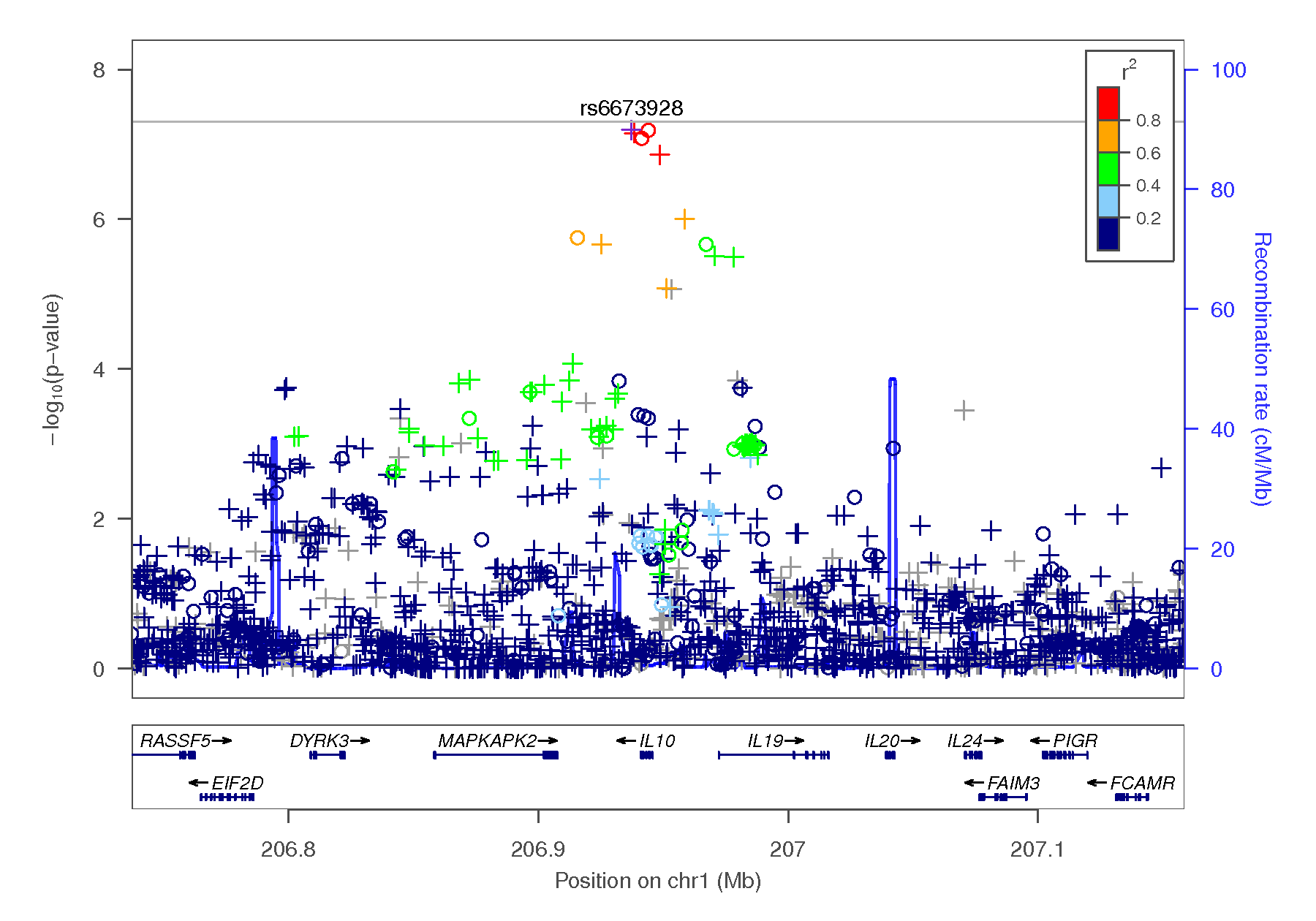


1. Regional plot of rs11265461, close to *CD48*


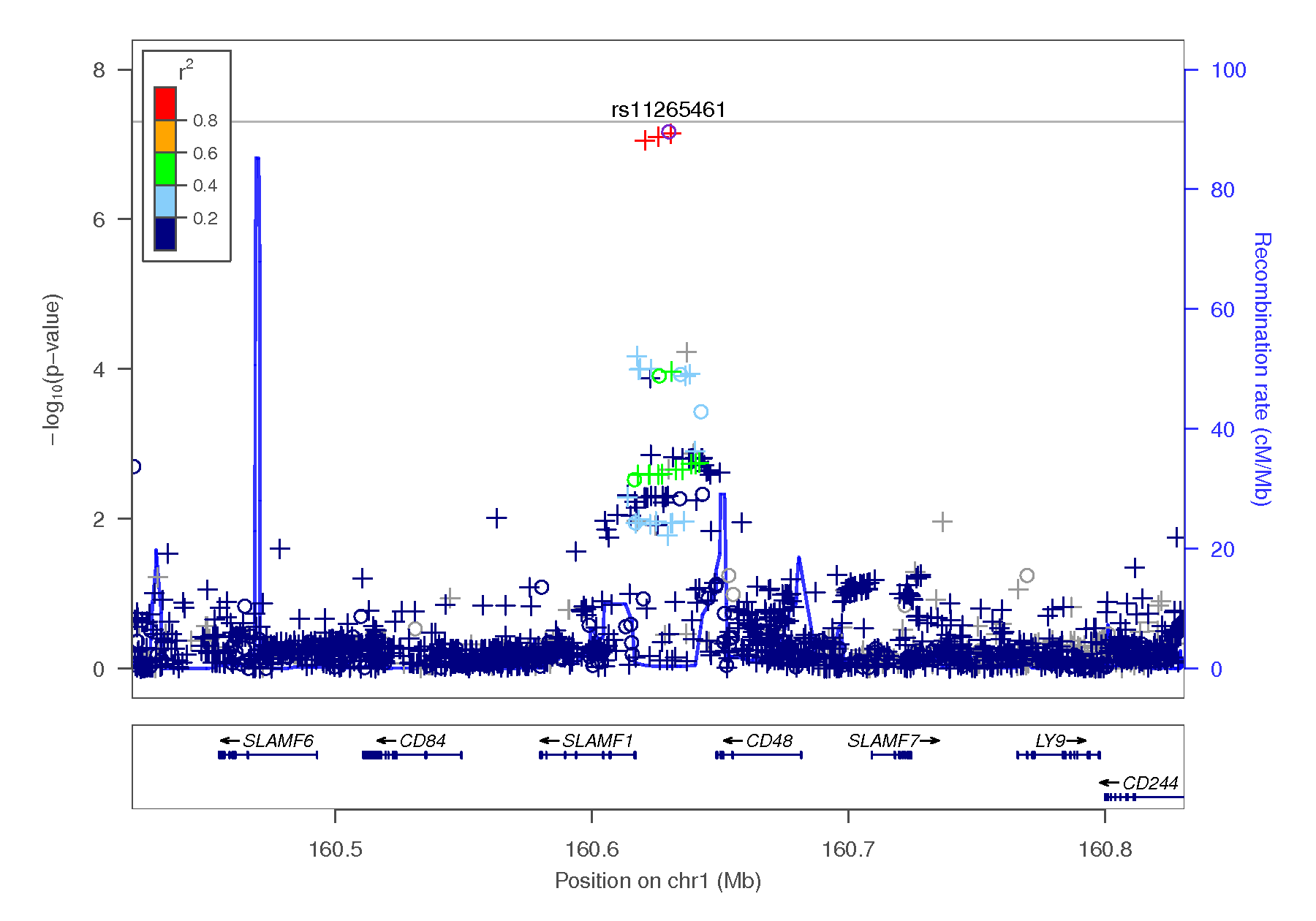


1. Regional plot of rs12123821, close to *HRNR*


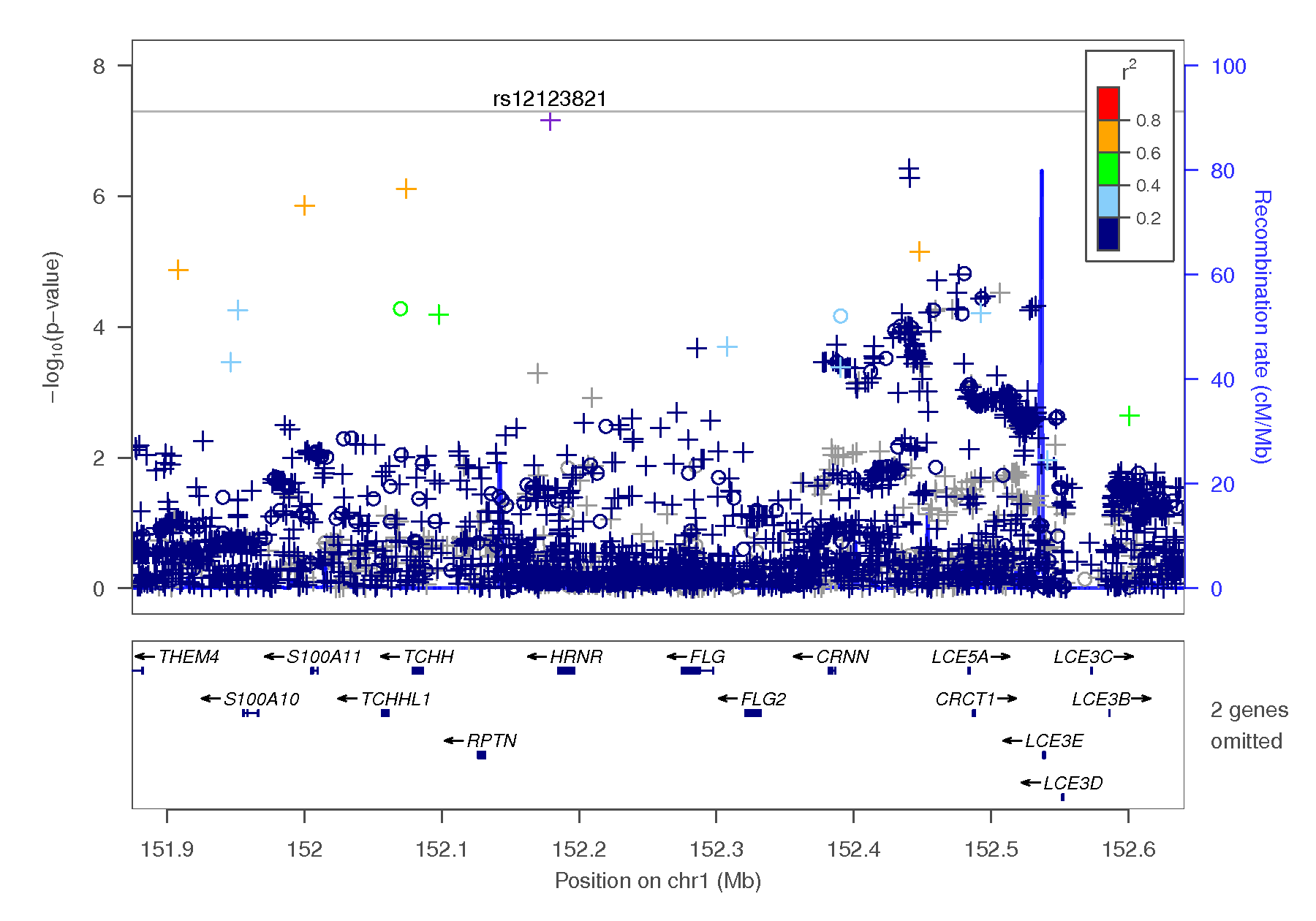


1. Regional plot of rs72816448, close to *FOXN2*


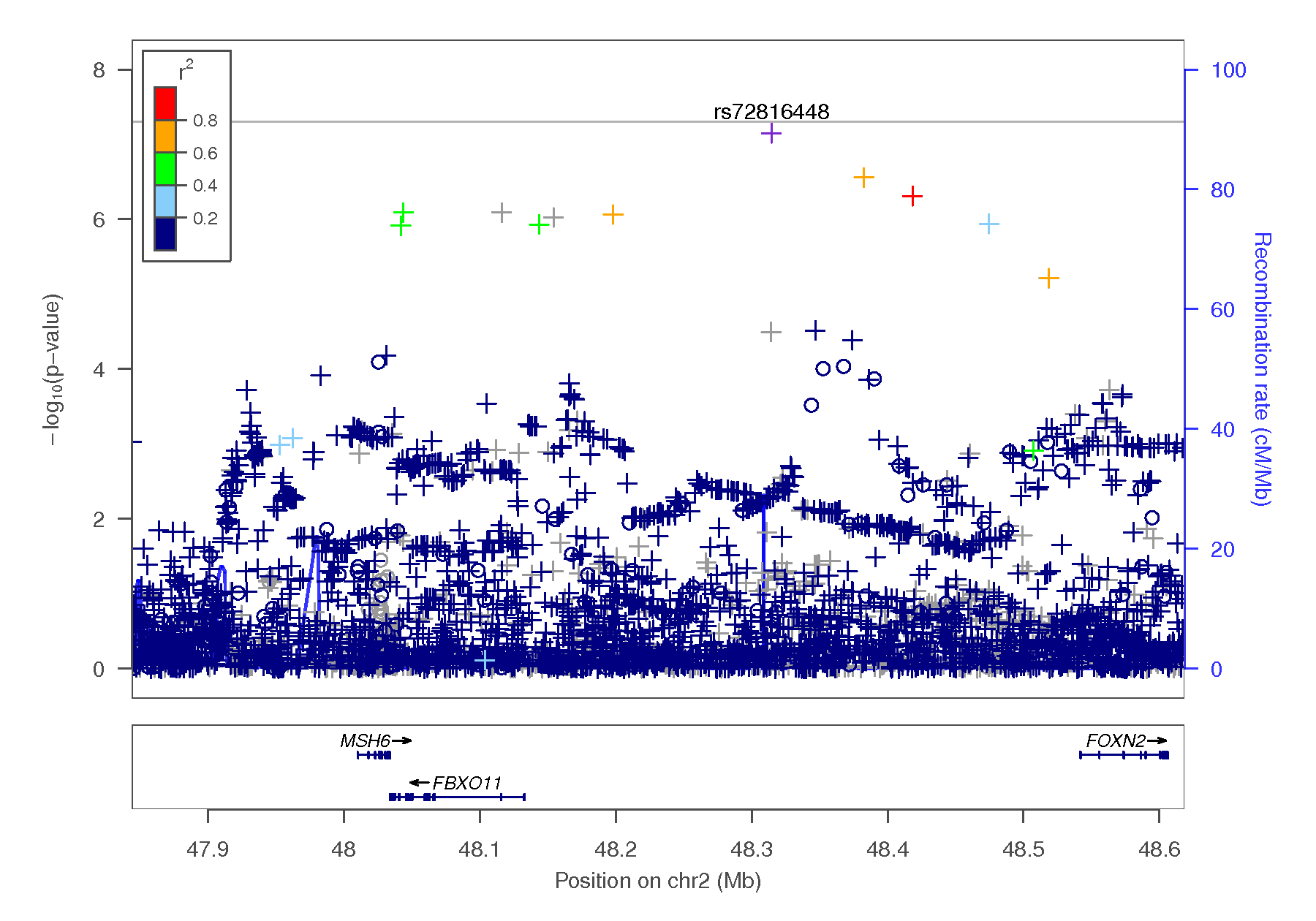


1. Regional plot of rs78037977, close to *FASLG*


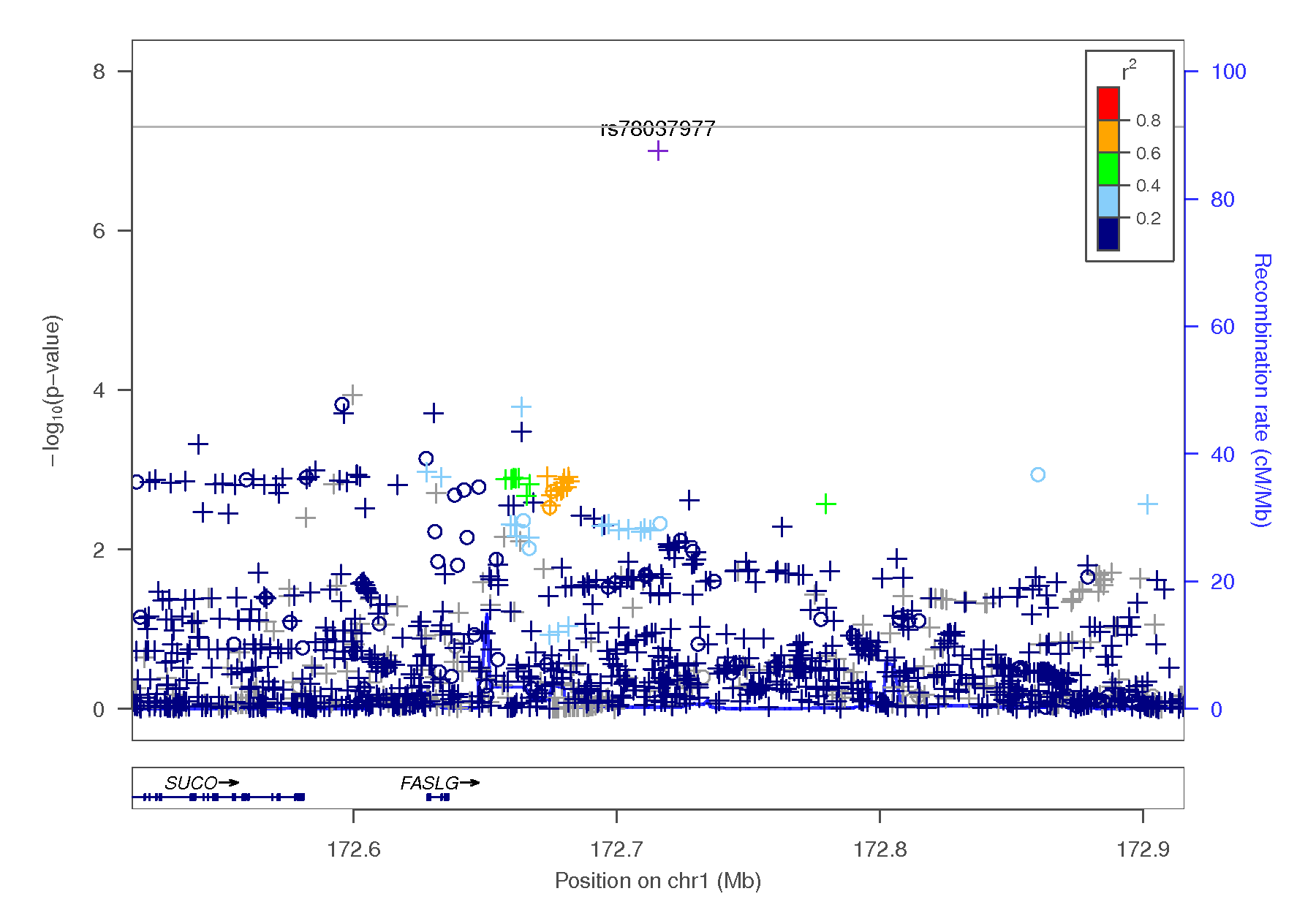


1. Regional plot of rs35488337, co-localising with *ETS1*


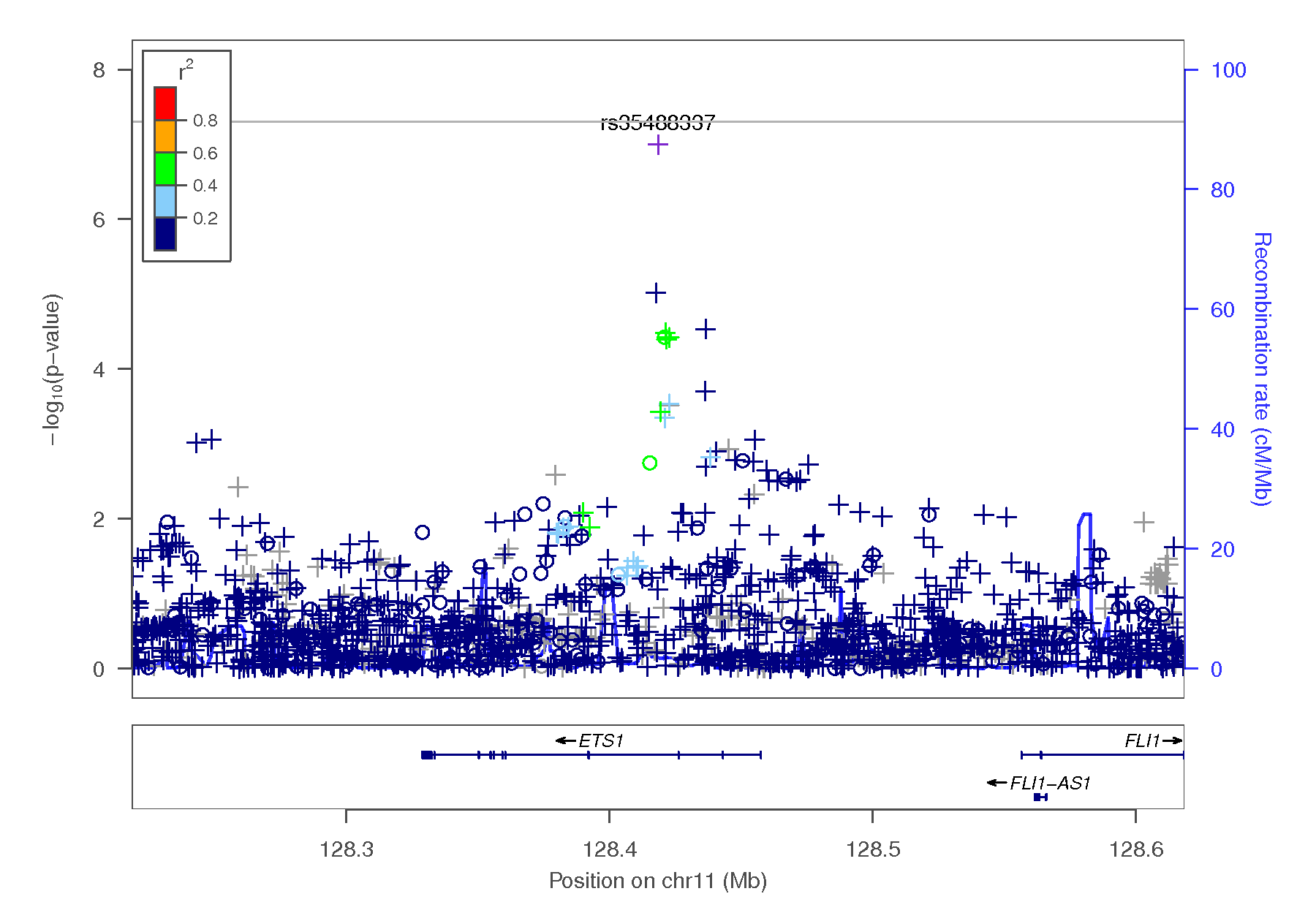


**Regional plots of itch intensity from mosquito bites**

1. Regional plot of rs2248116, close to *IRF1*


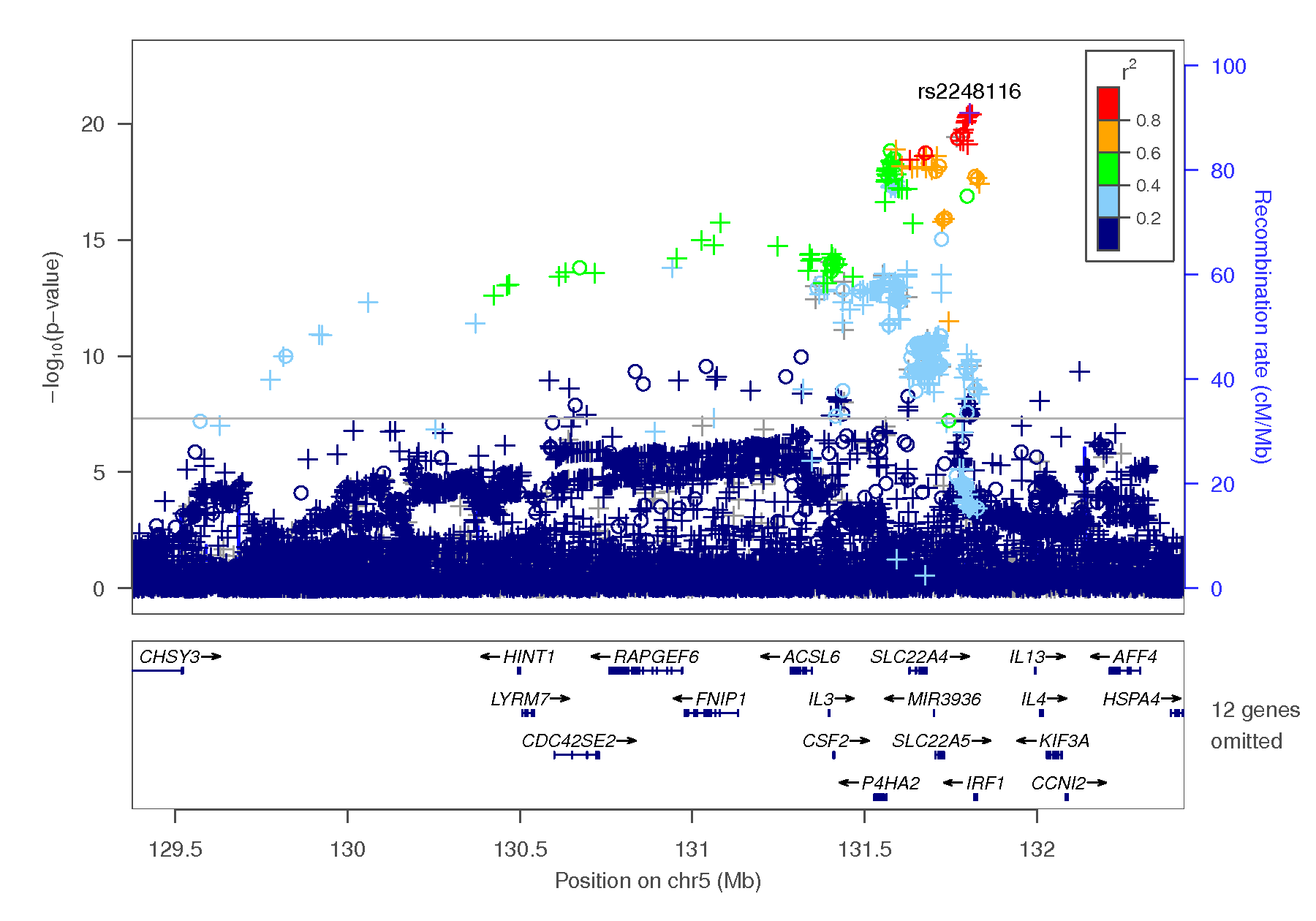


1. Regional plot of rs309394, close to *IL21*


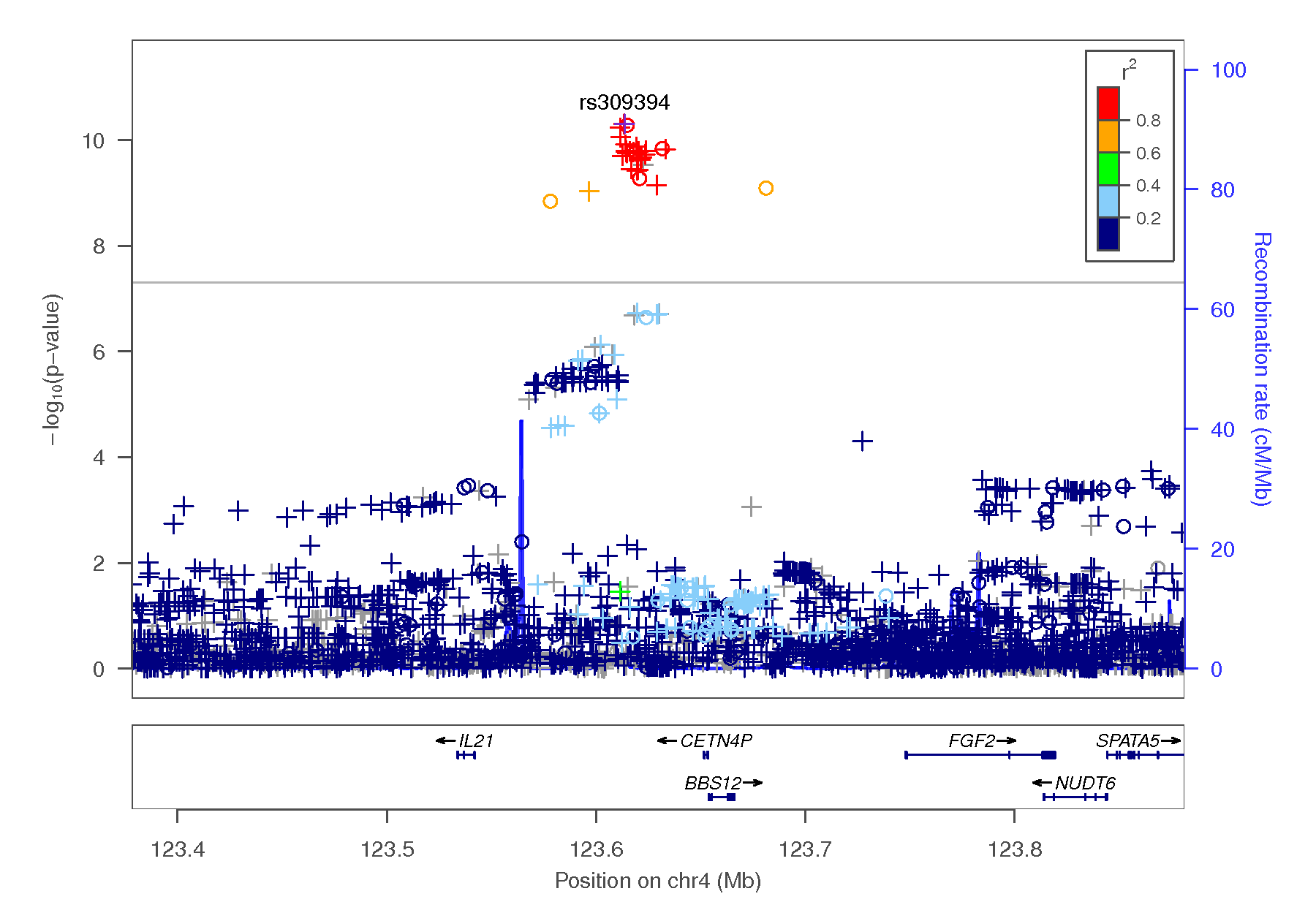


1. Regional plot of rs12055445, close to *HLA-DQB1*


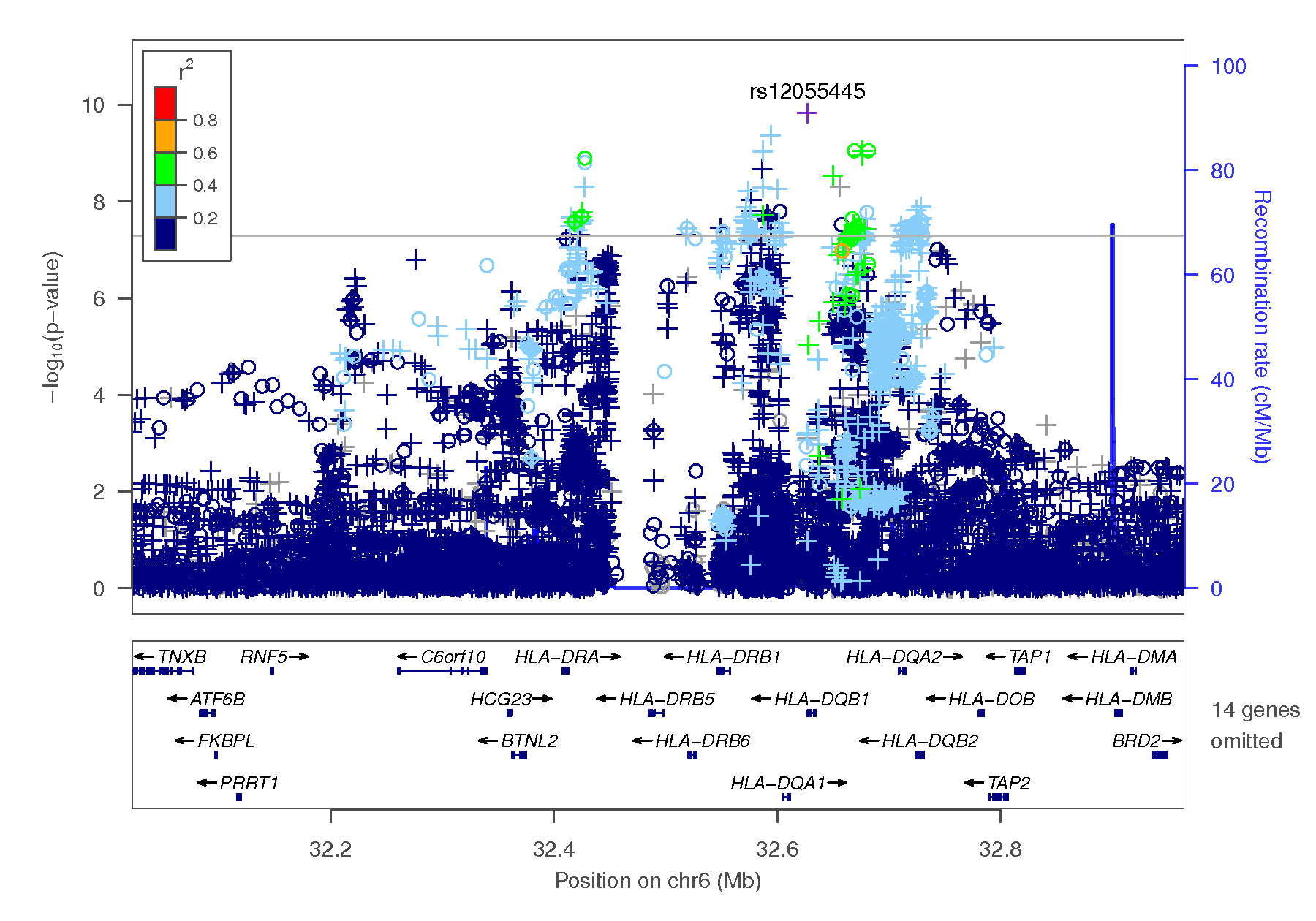


1. Regional plot of rs2523614, close to *HLA-B*


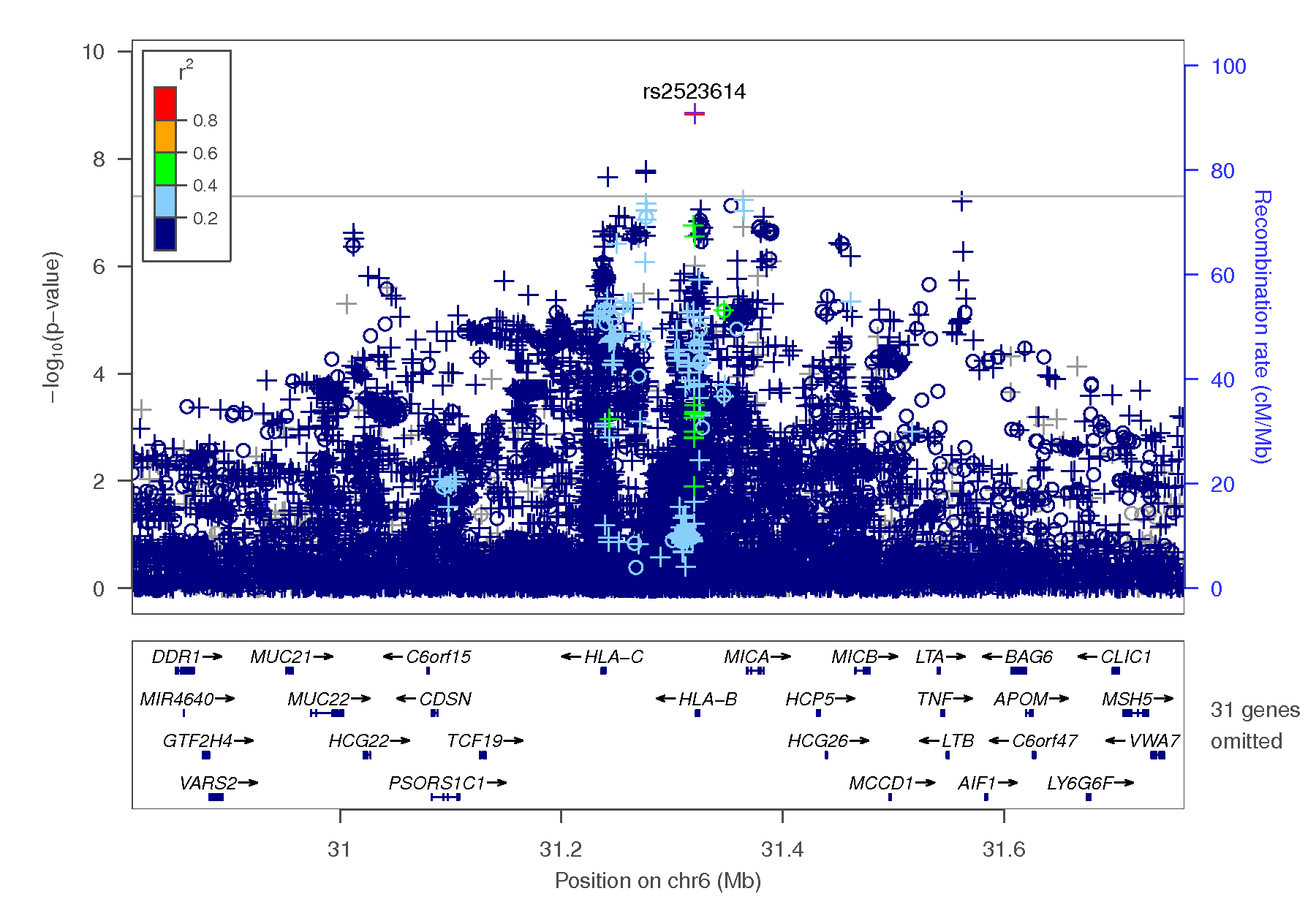


1. Regional plot of rs2814244, close to *IFNG*


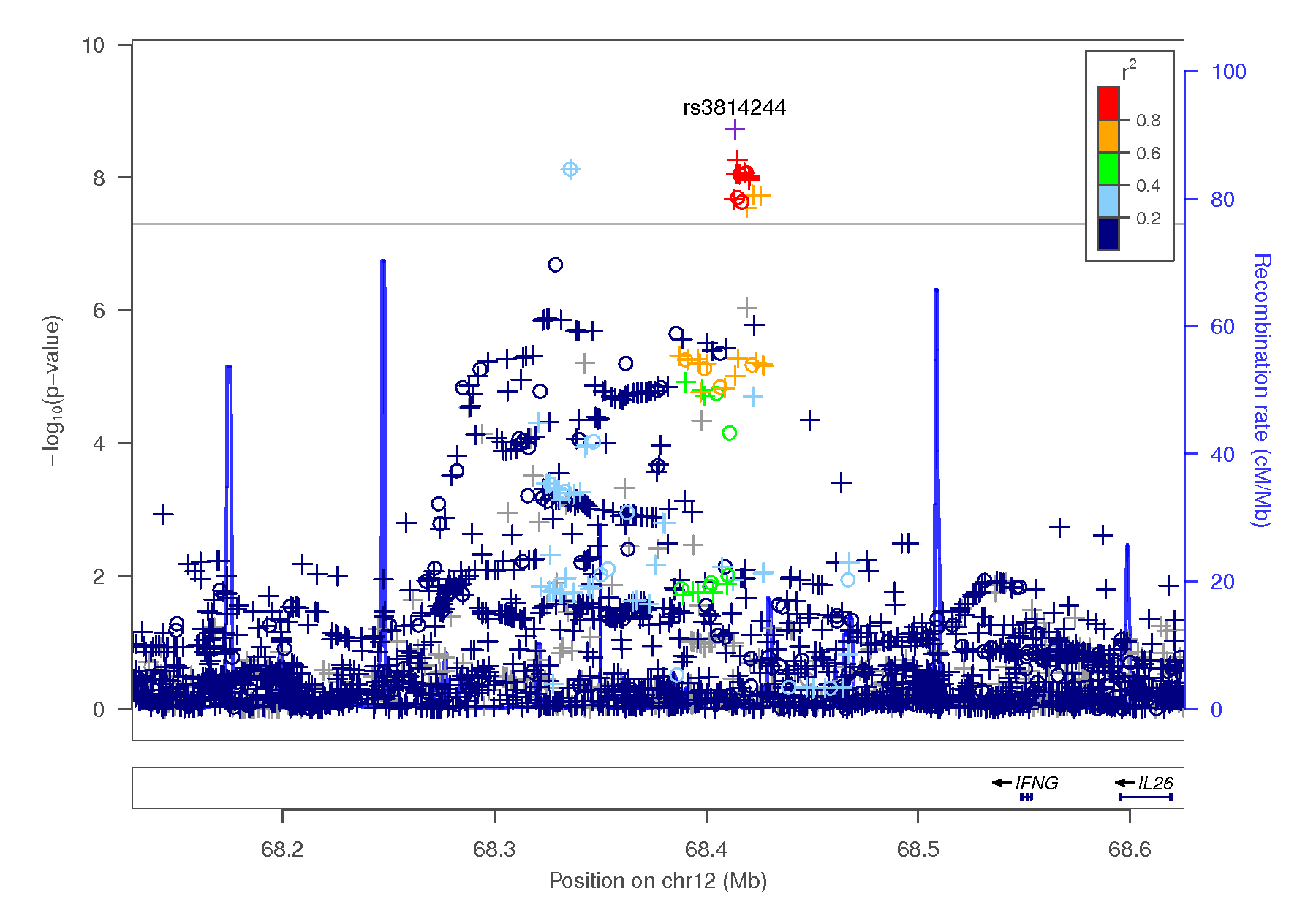


1. Regional plot of rs4499342, close to *ACTL9*


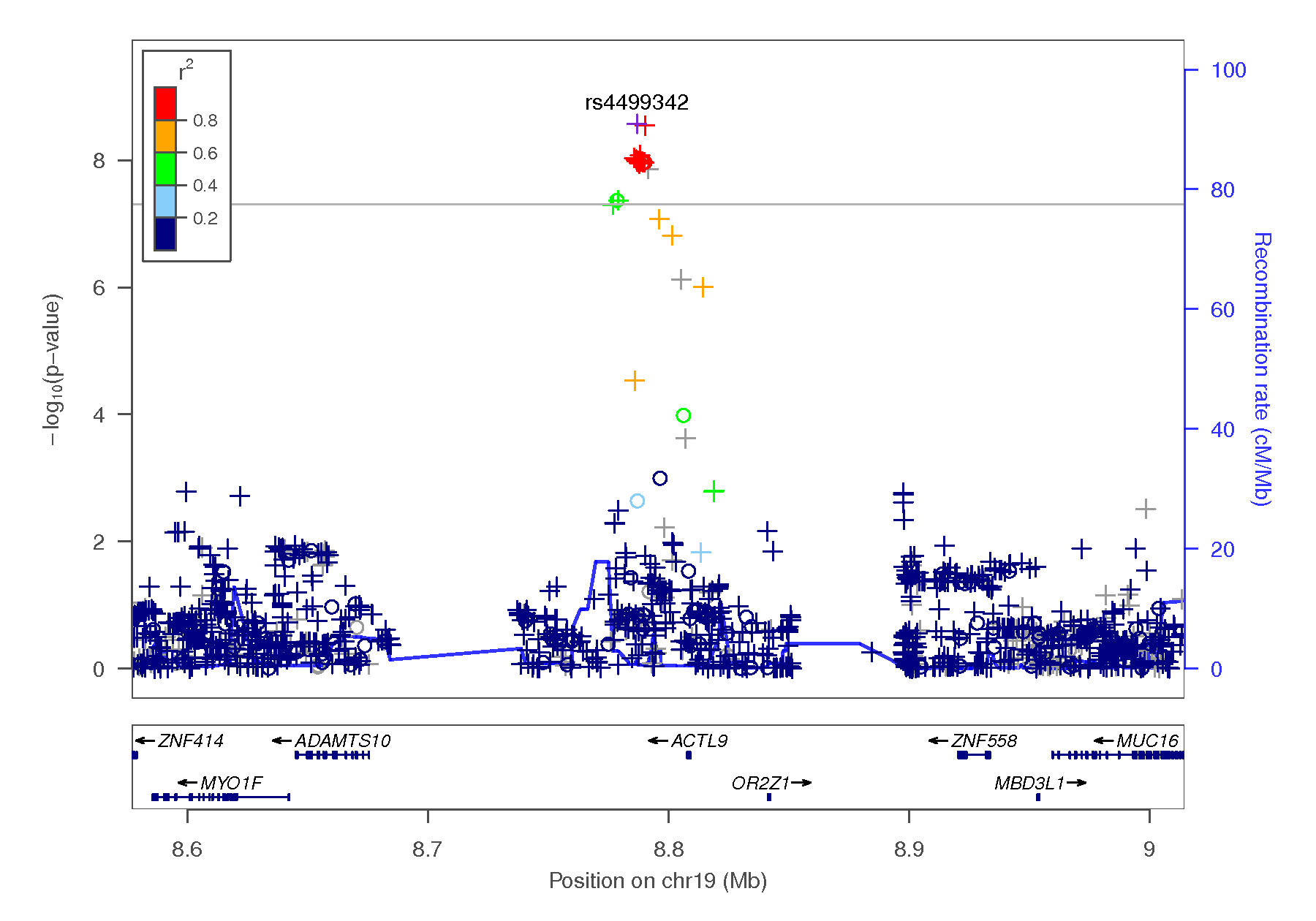


1. Regional plot of rs778798, co-localising with *FUT6*


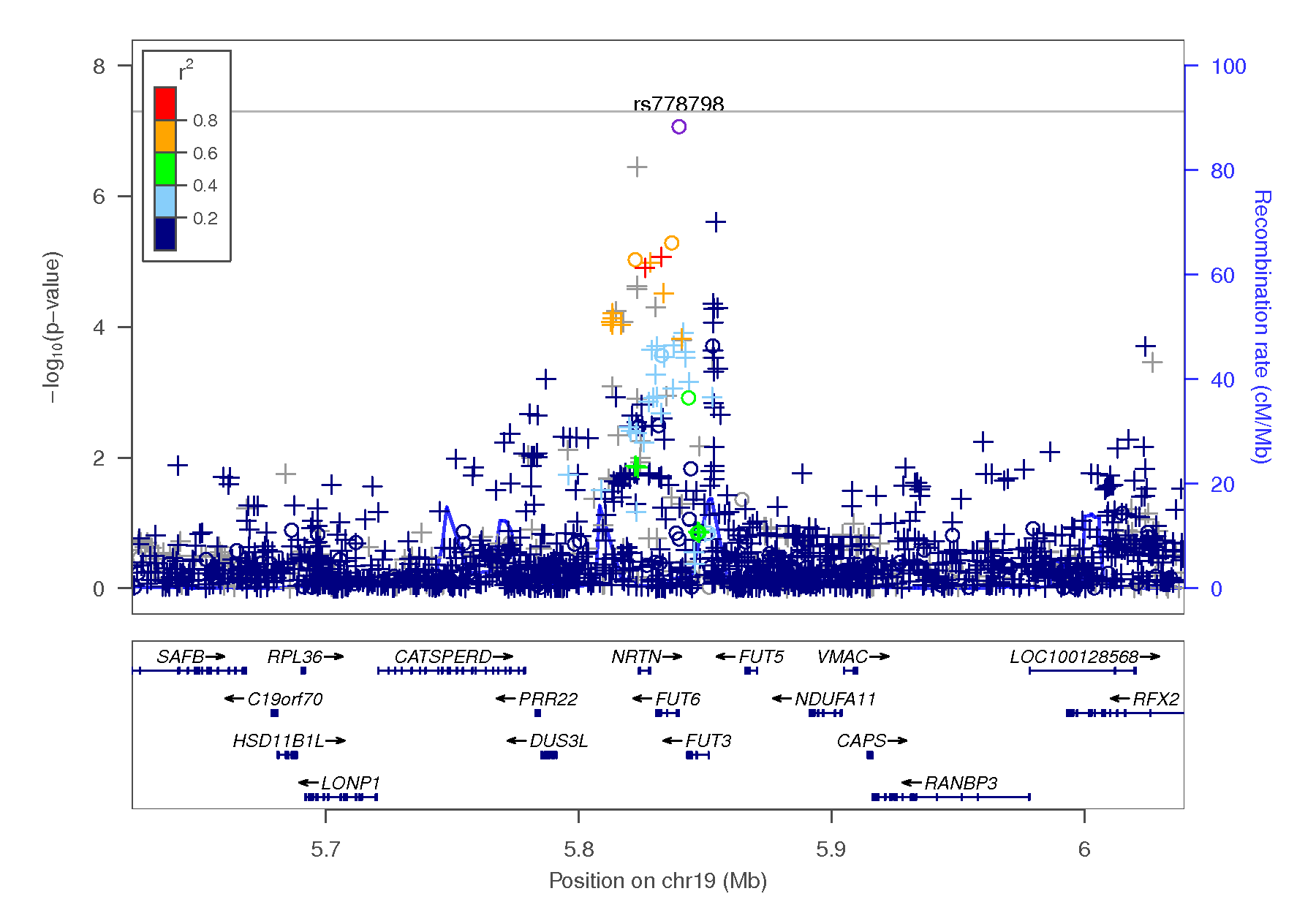


1. Regional plot of rs5796229, co-localising with *LTBR*


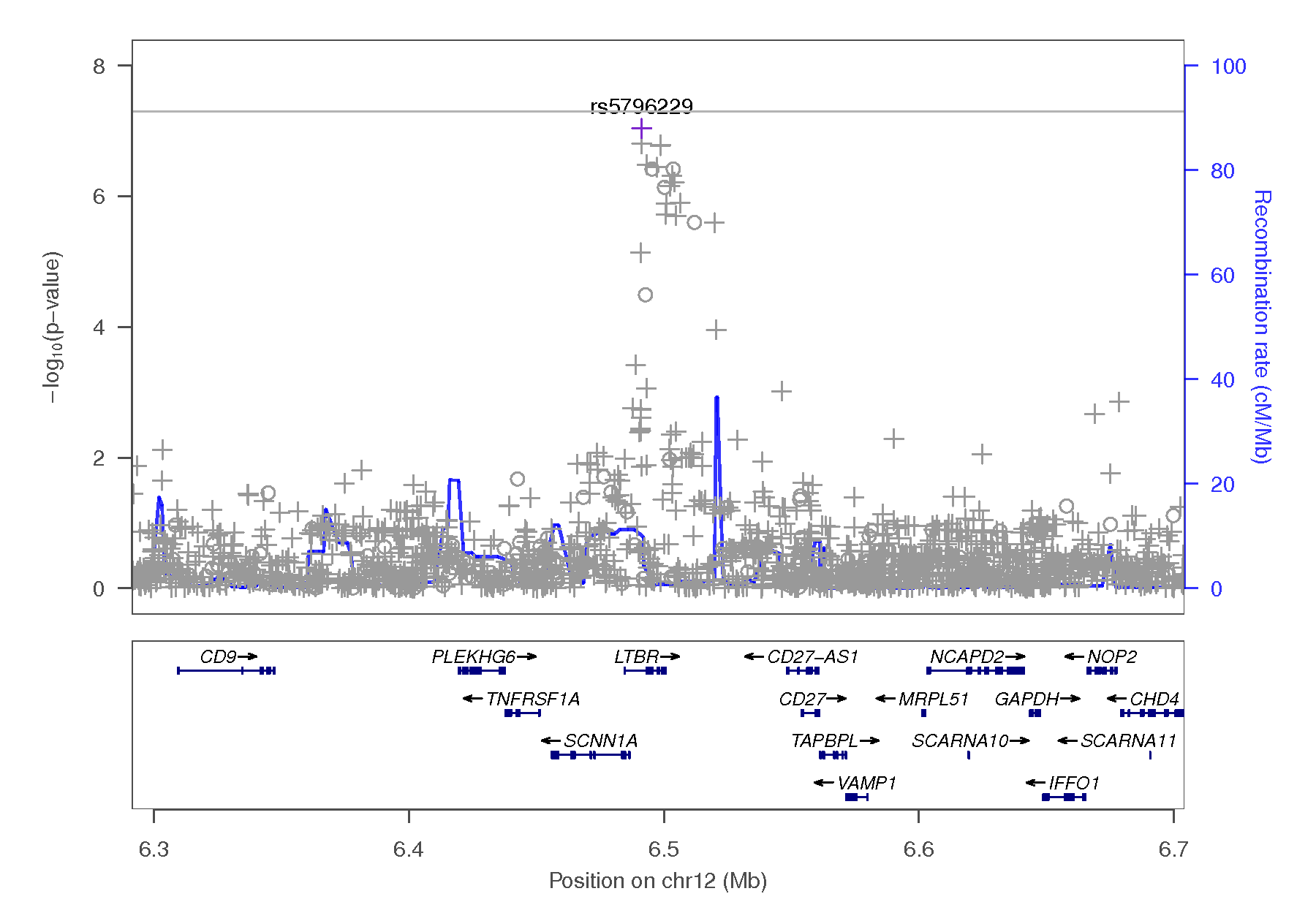


1. Regional plot of rs183794680, close to *ARID3B*


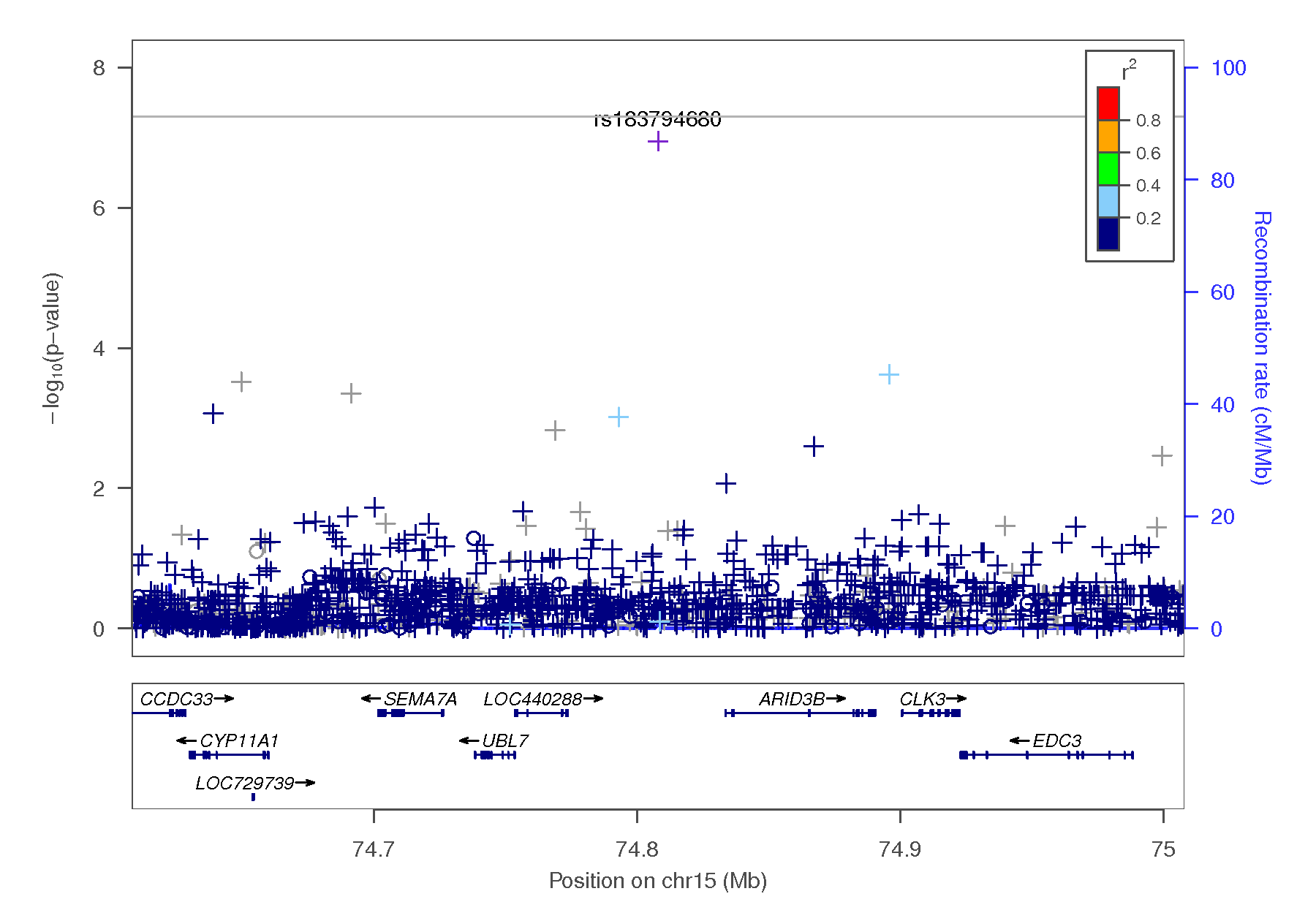


1. Regional plot of rs72816448, close to *FBXO11*


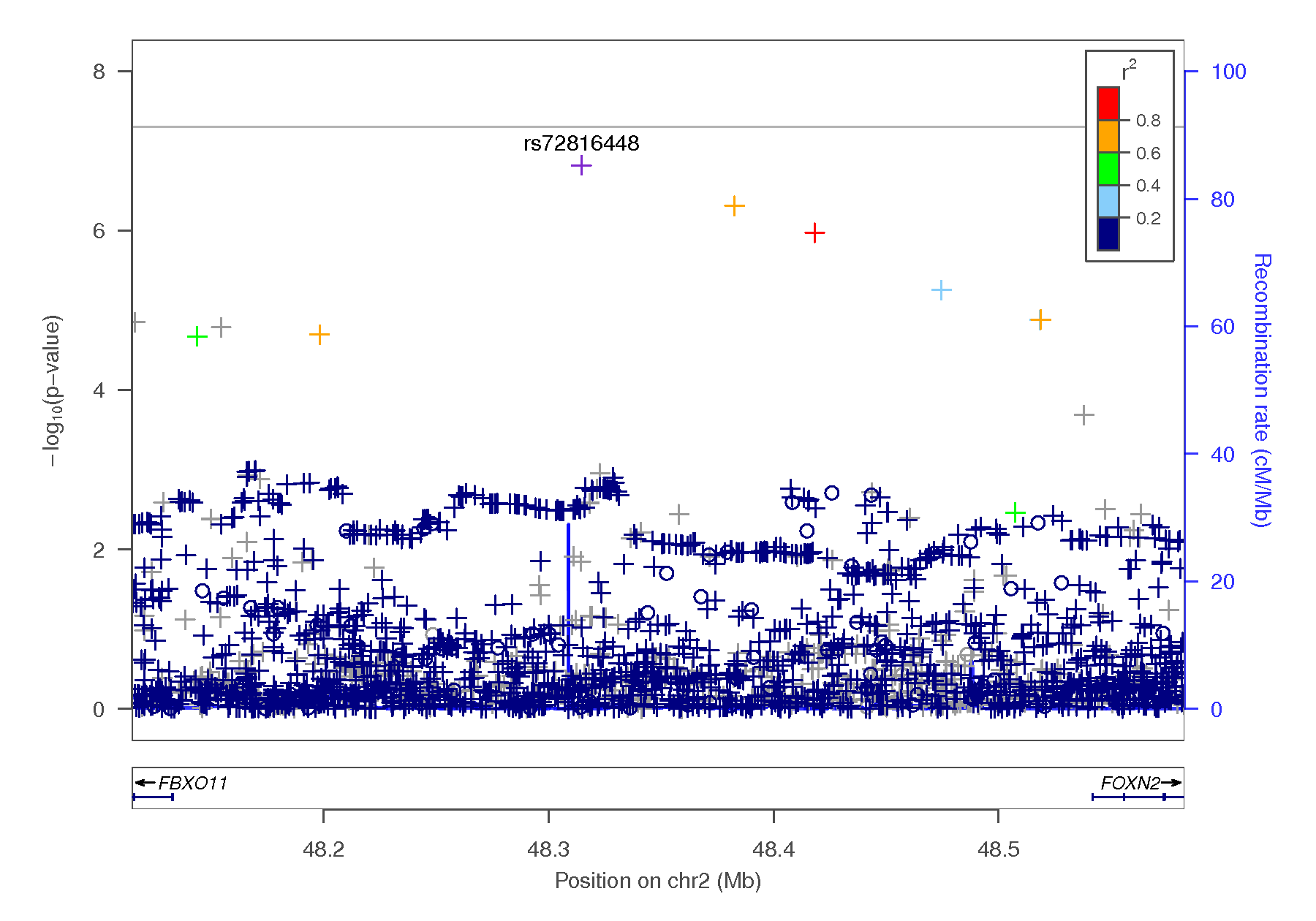


1. Regional plot of rs10117812, co-localising with *MAPKAP1*


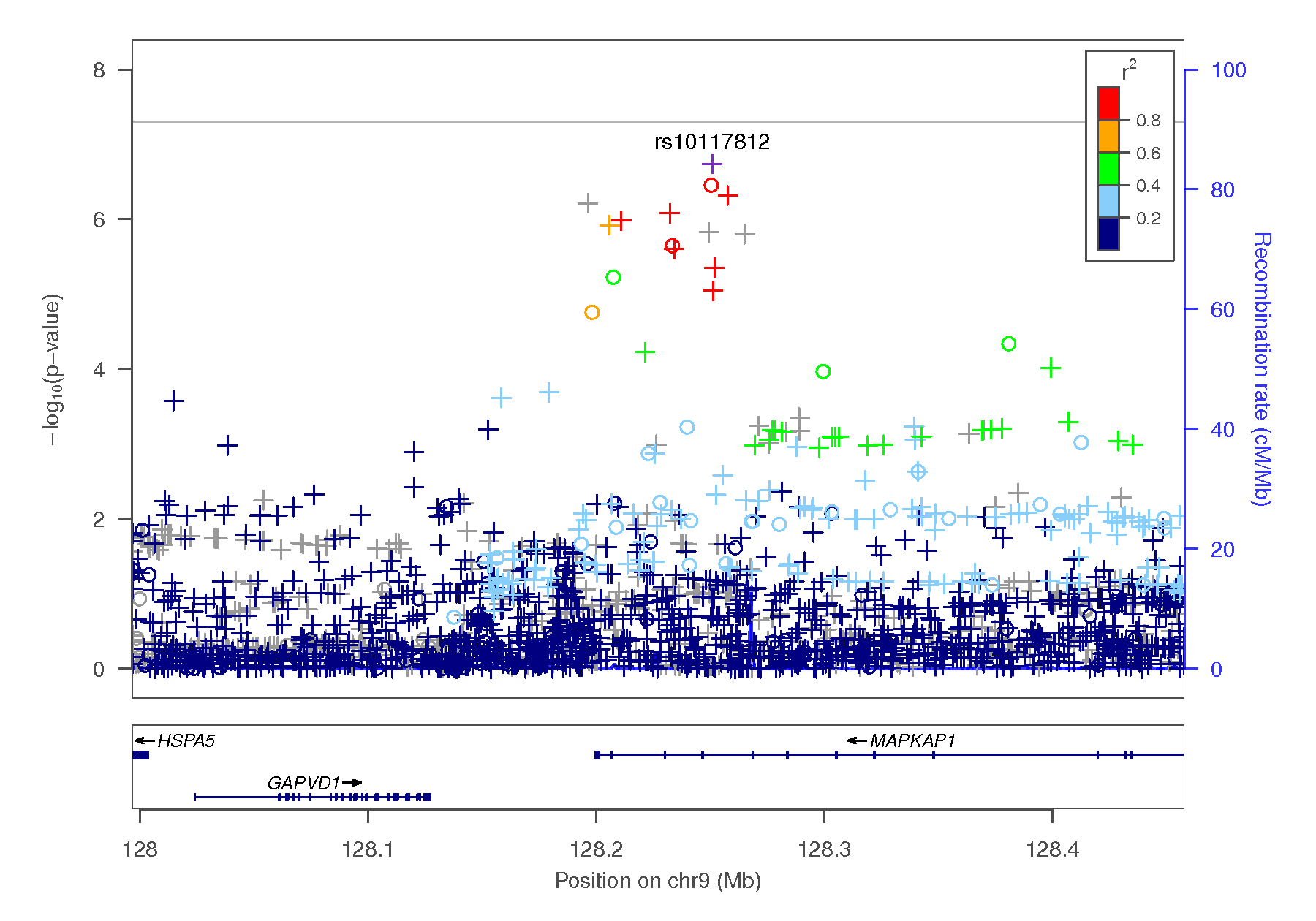


1. Regional plot of rs4978899, close to *TXN*


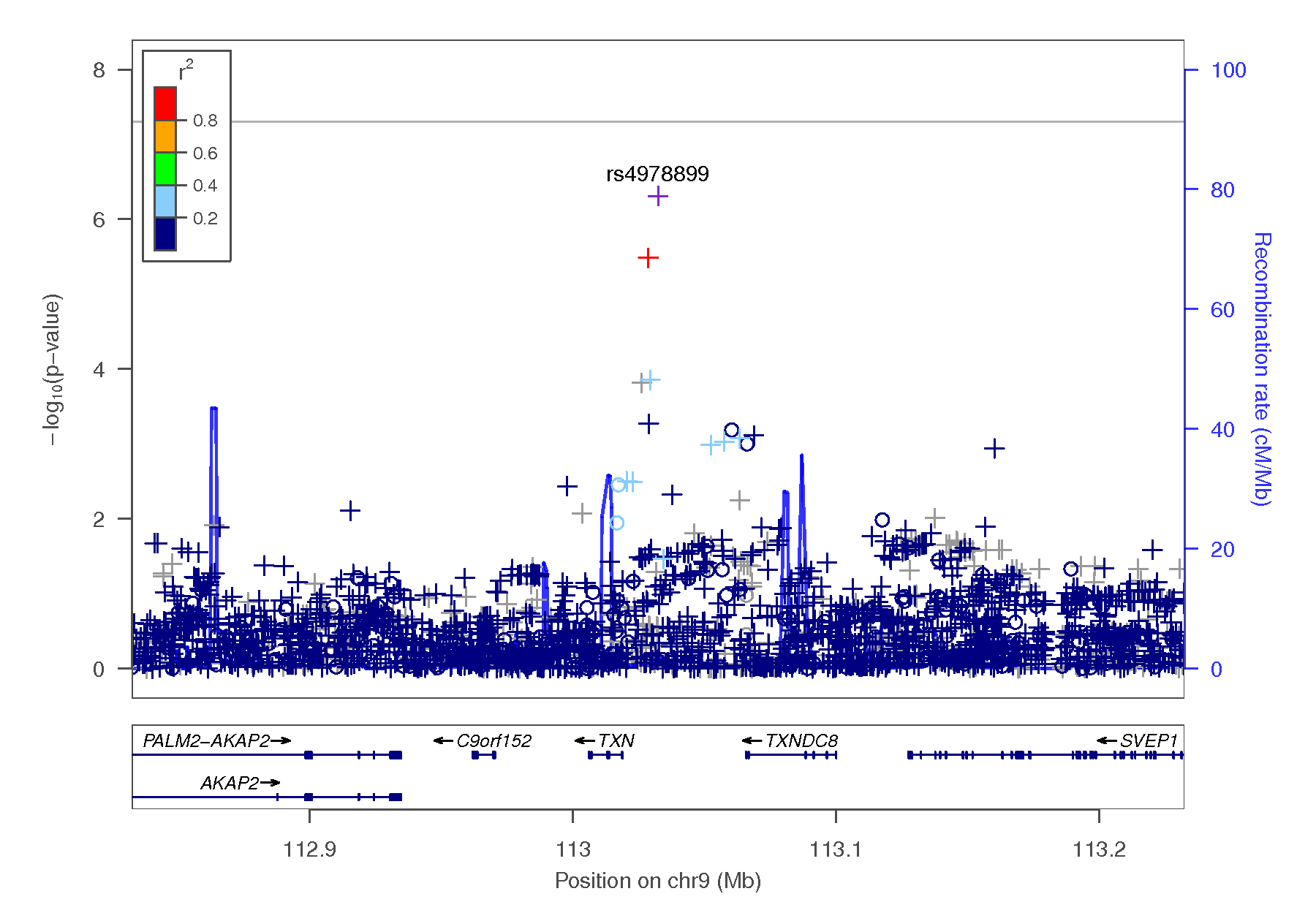


1. Regional plot of rs5756391, close to *CSF2RB*


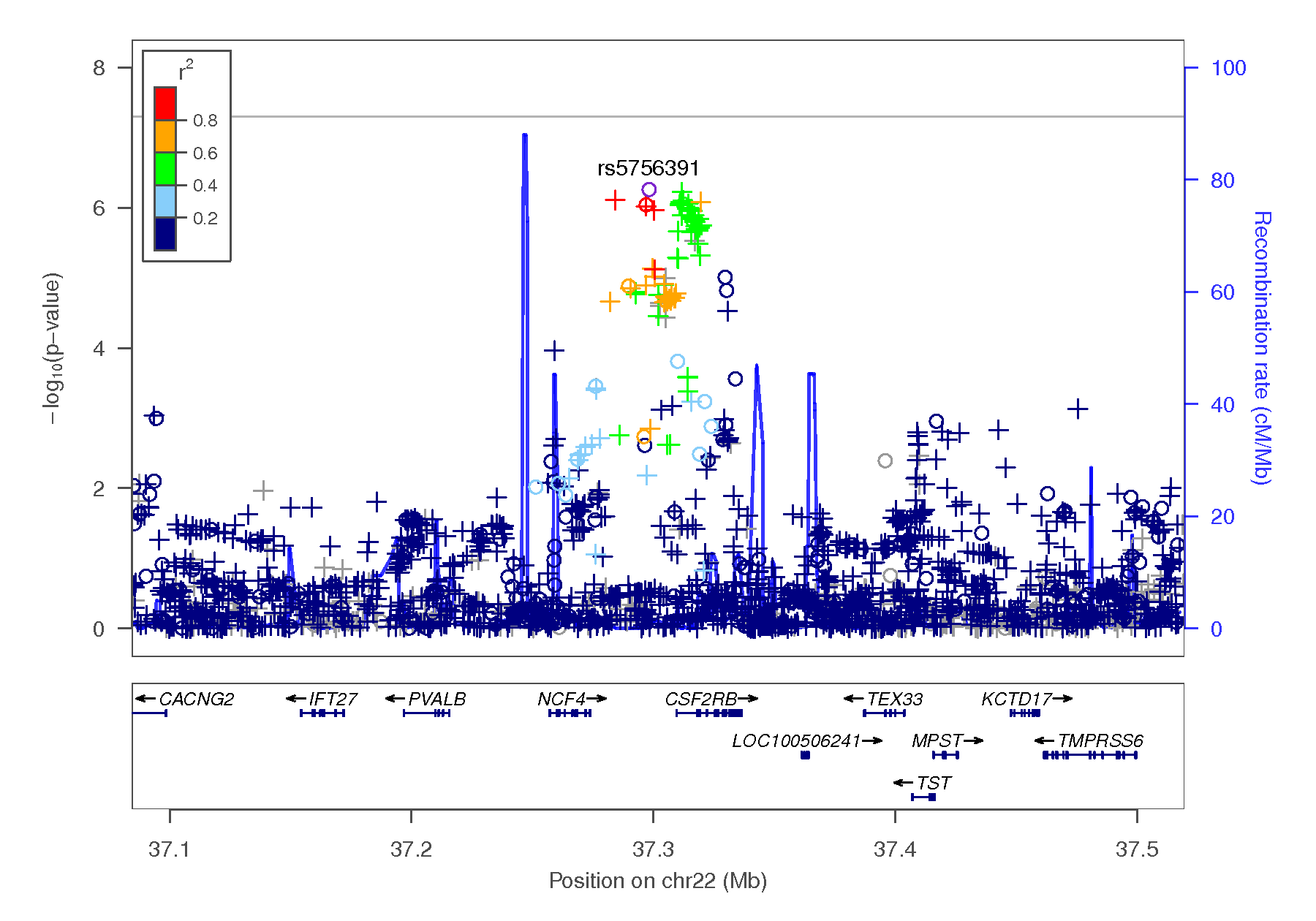


1. Regional plot of rs140260076, close to *LOC728342*


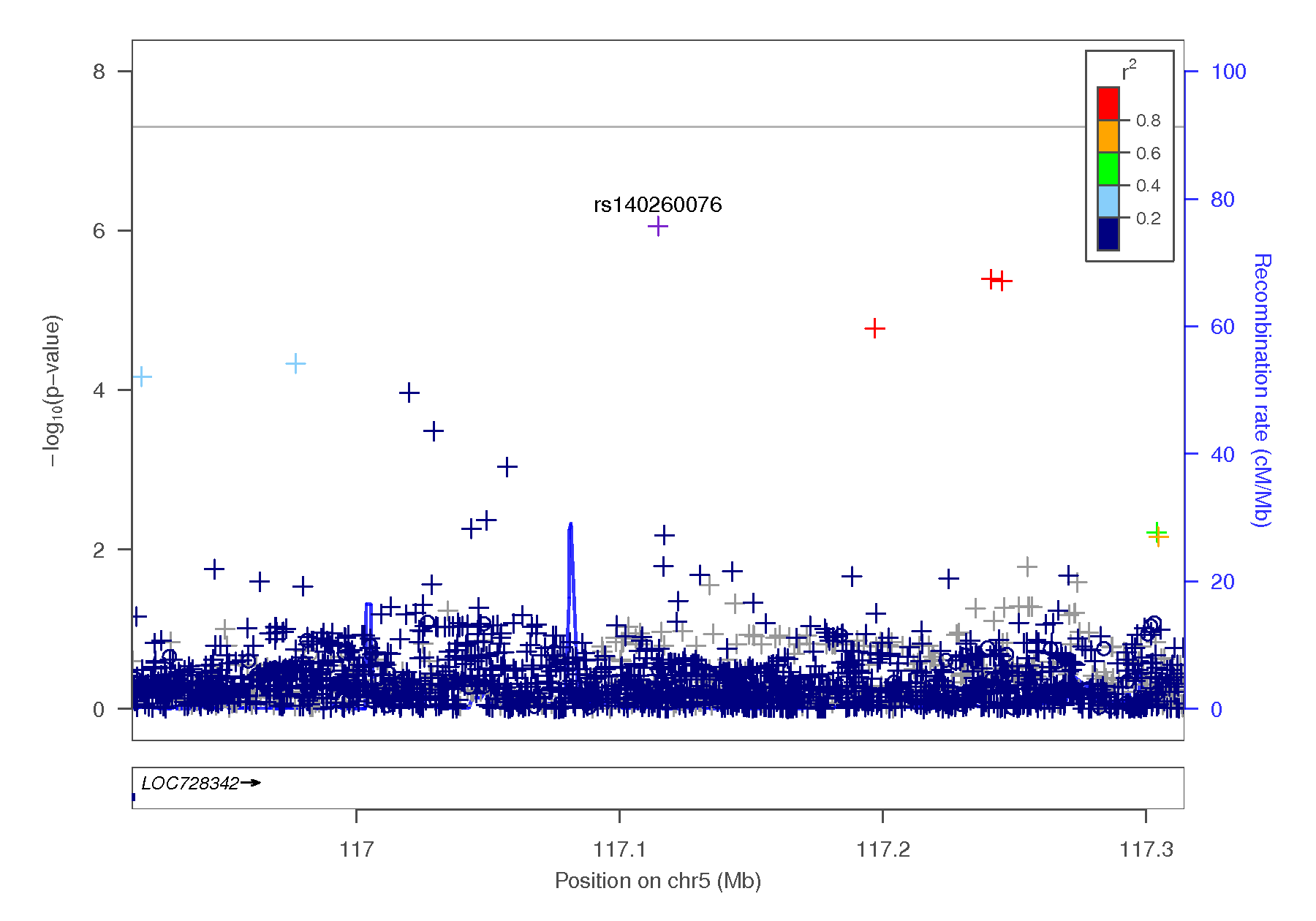


1. Regional plot of rs55665660, co-localising with *LOC285419*


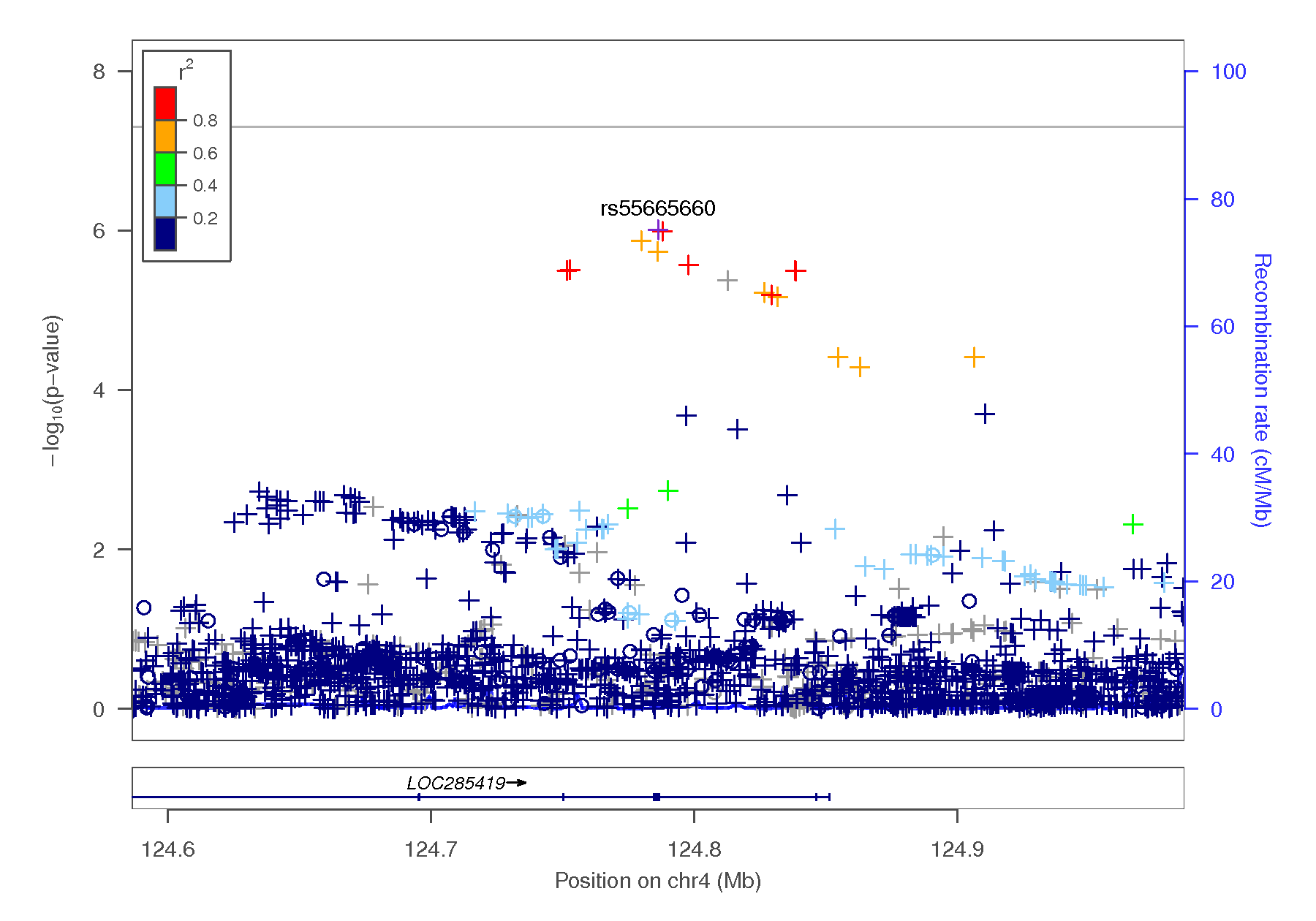


**Regional plots for perceived attractiveness to mosquitoes.**

1. Regional plots of rs309403, close to *IL21*


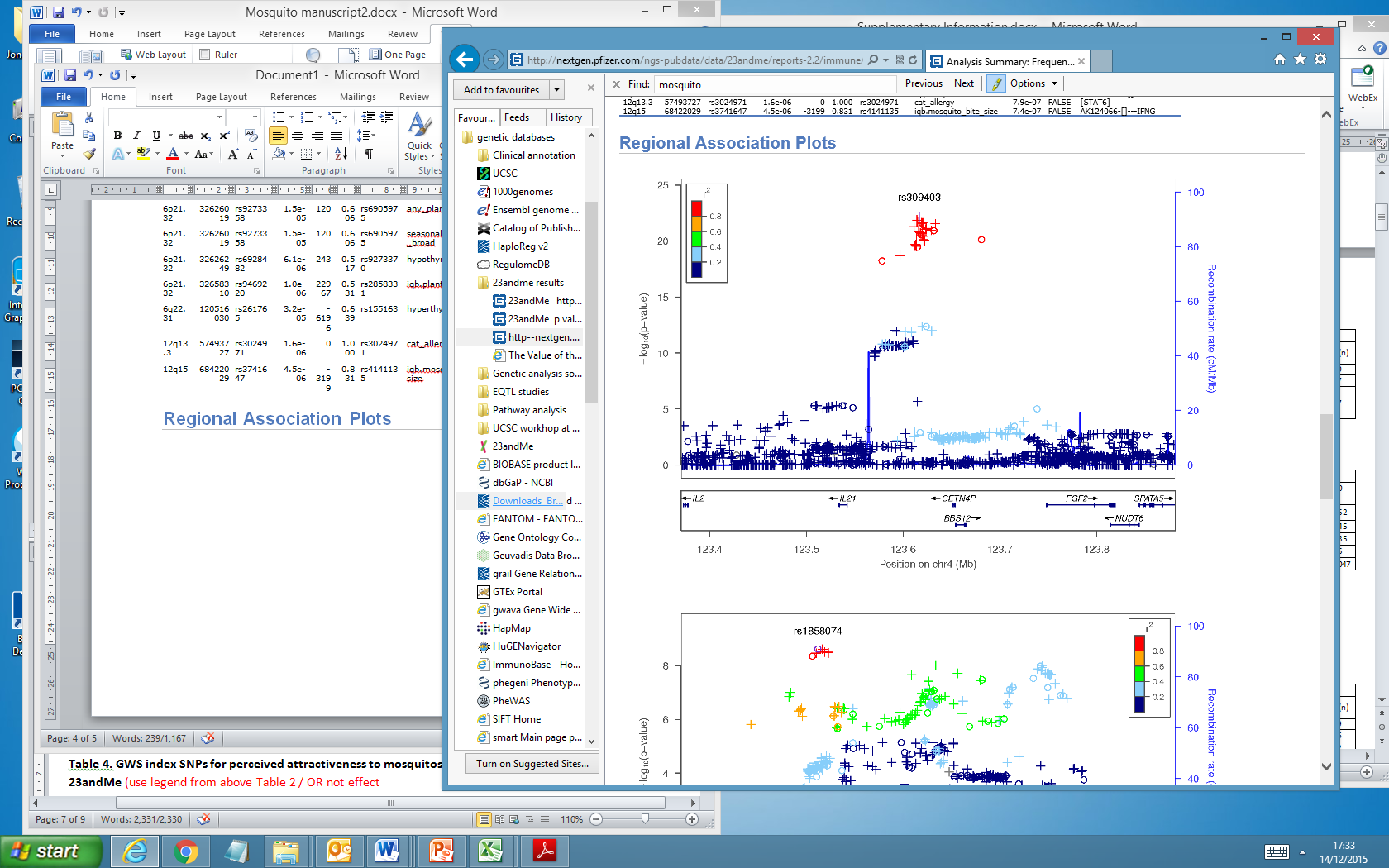


1. Regional plot of rs1858074, close to *IL3*


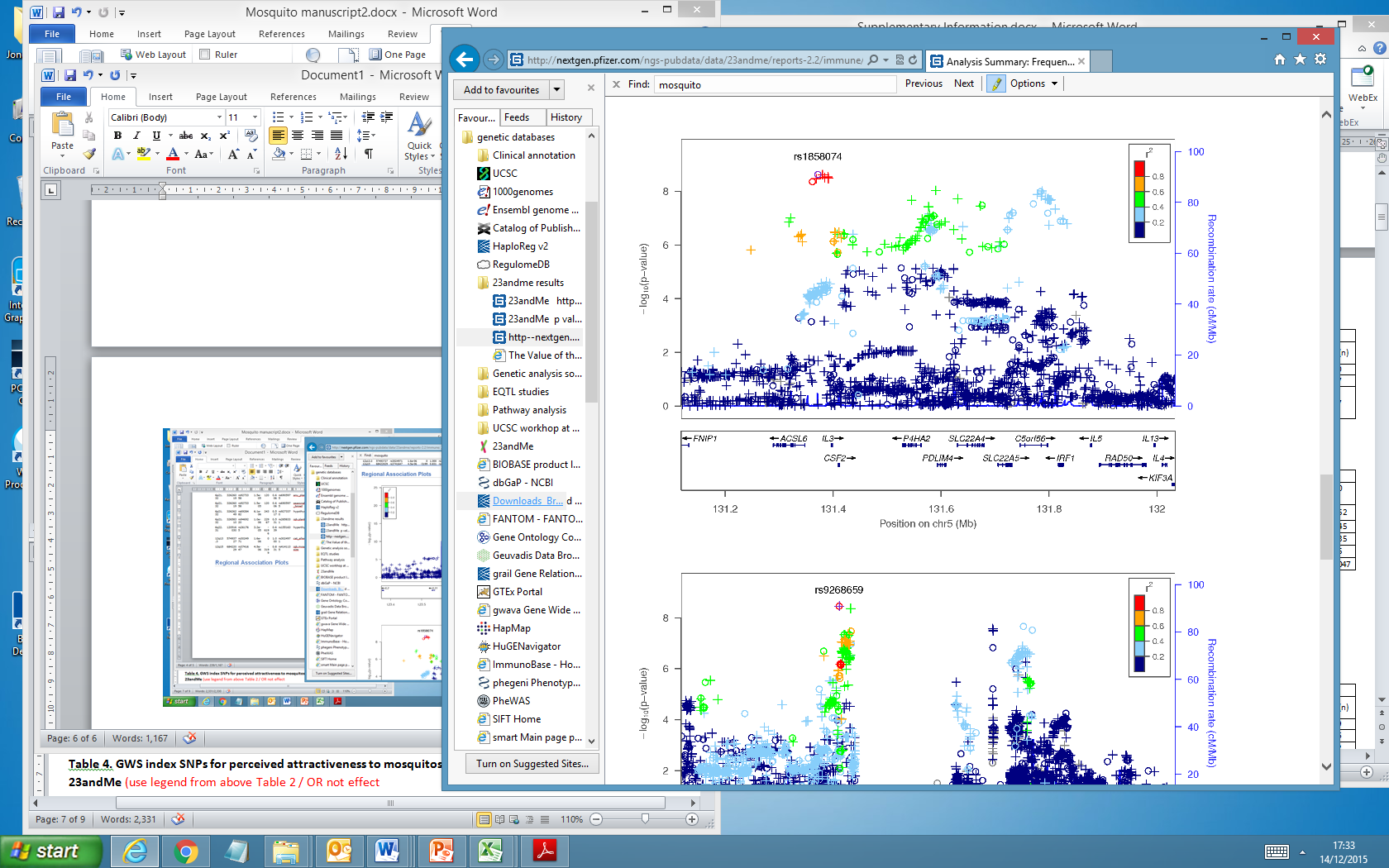


1. Regional plot of rs9268659, co-localising with *HLA-DRA*


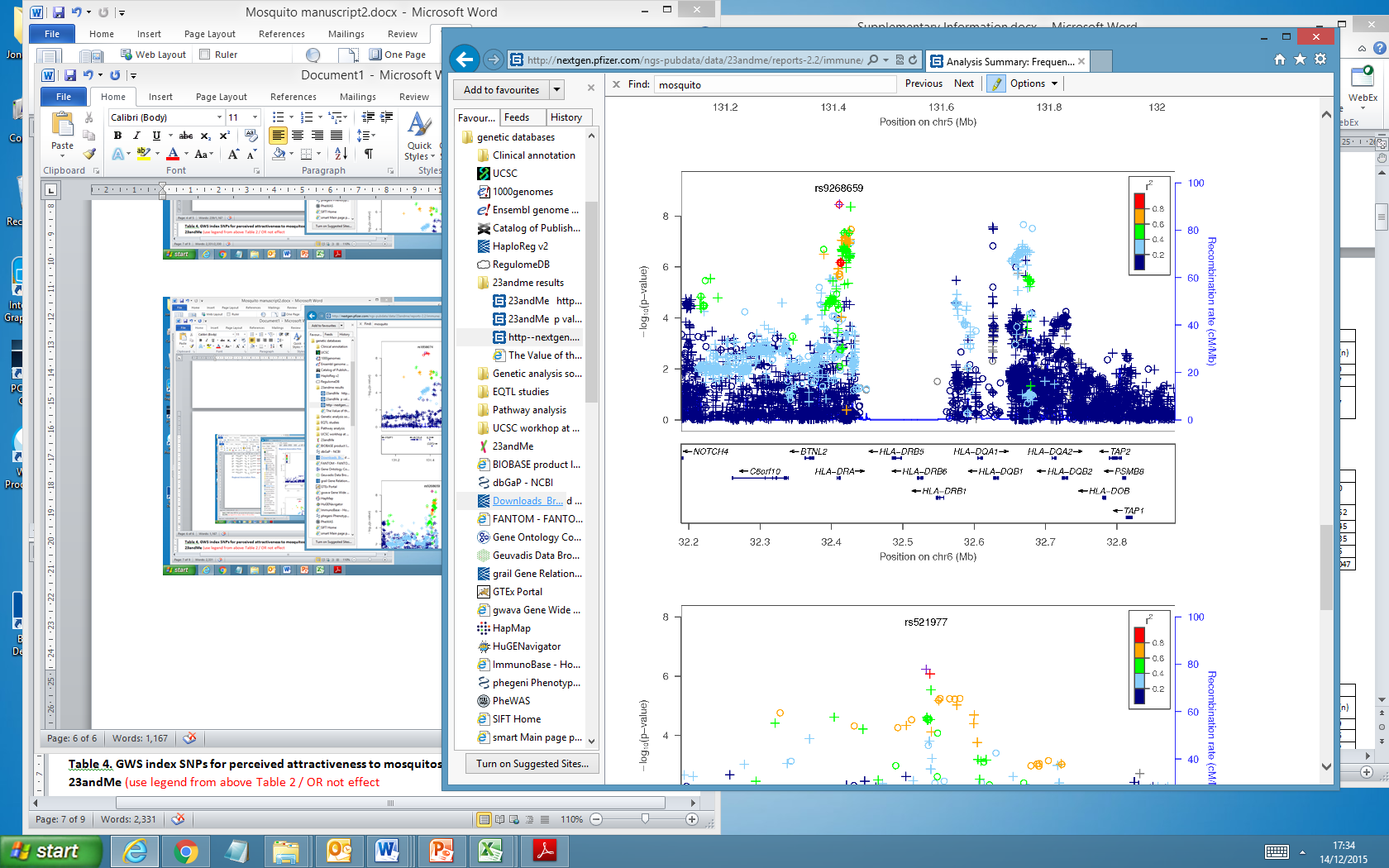


1. Regional plot of rs521977, co-localising with *SLC44A4*


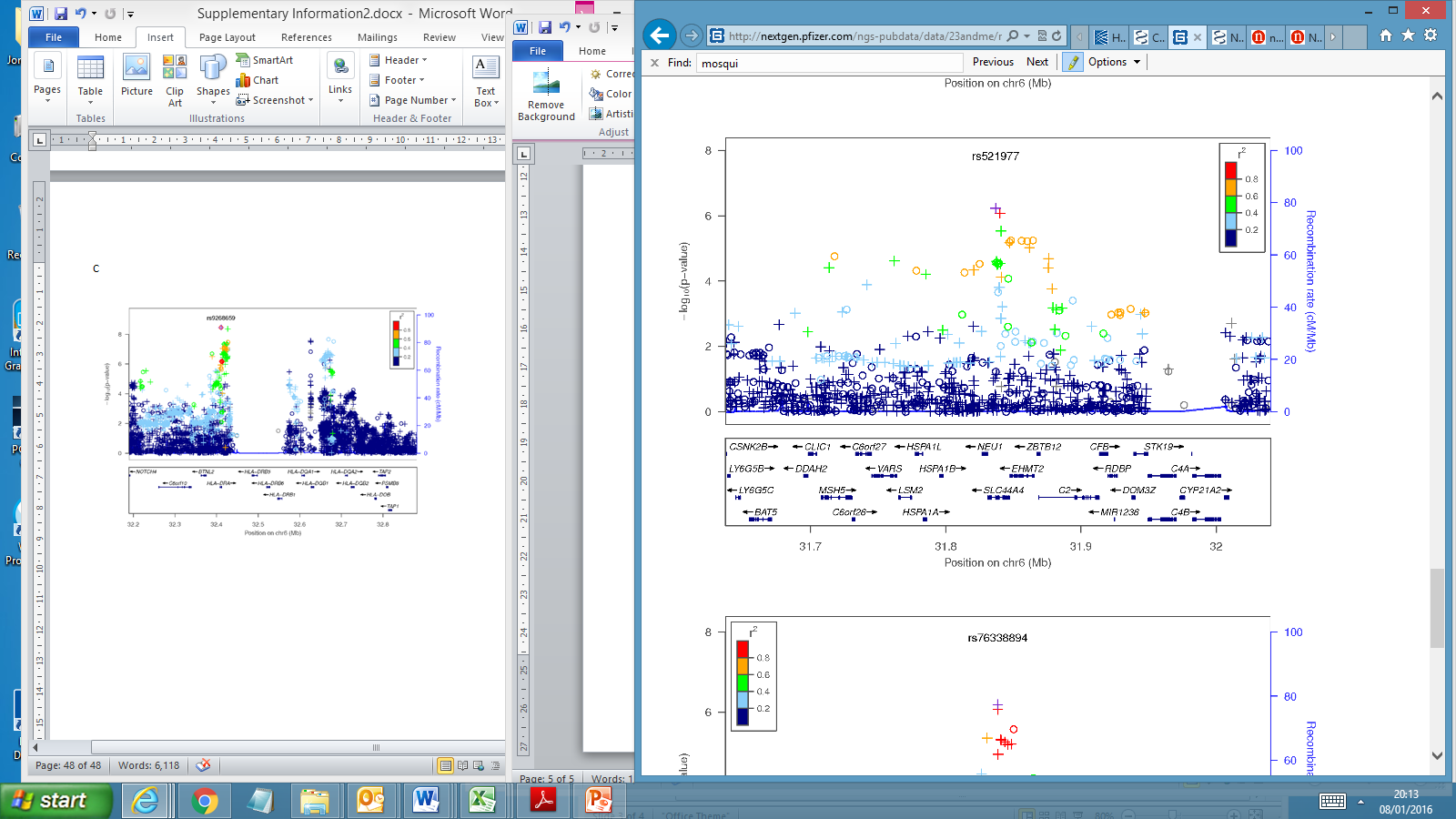


1. Regional plot of rs76338894, close to *NPHP4*


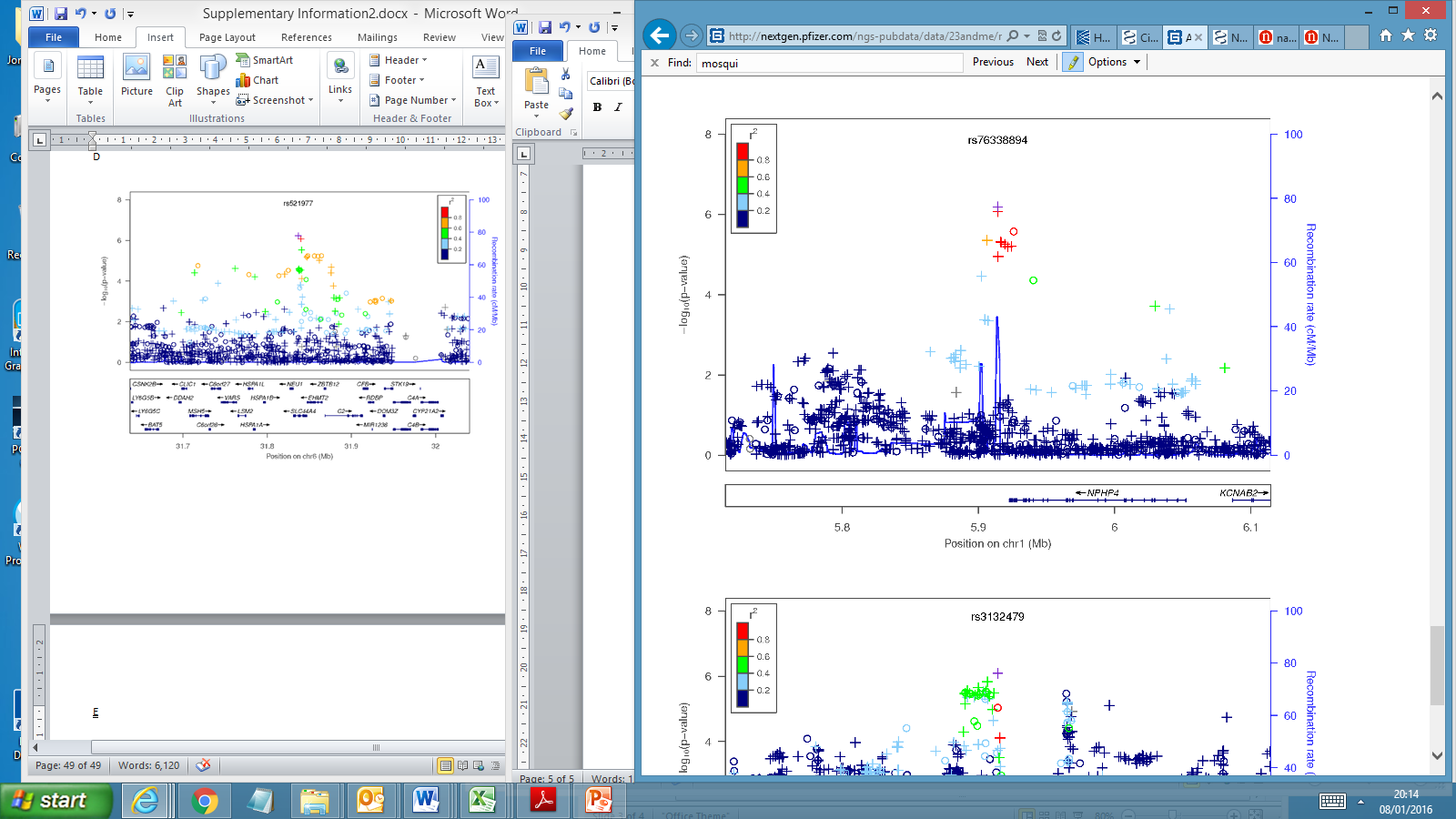


1. Regional plot of rs3132479, close to *HLA-C*


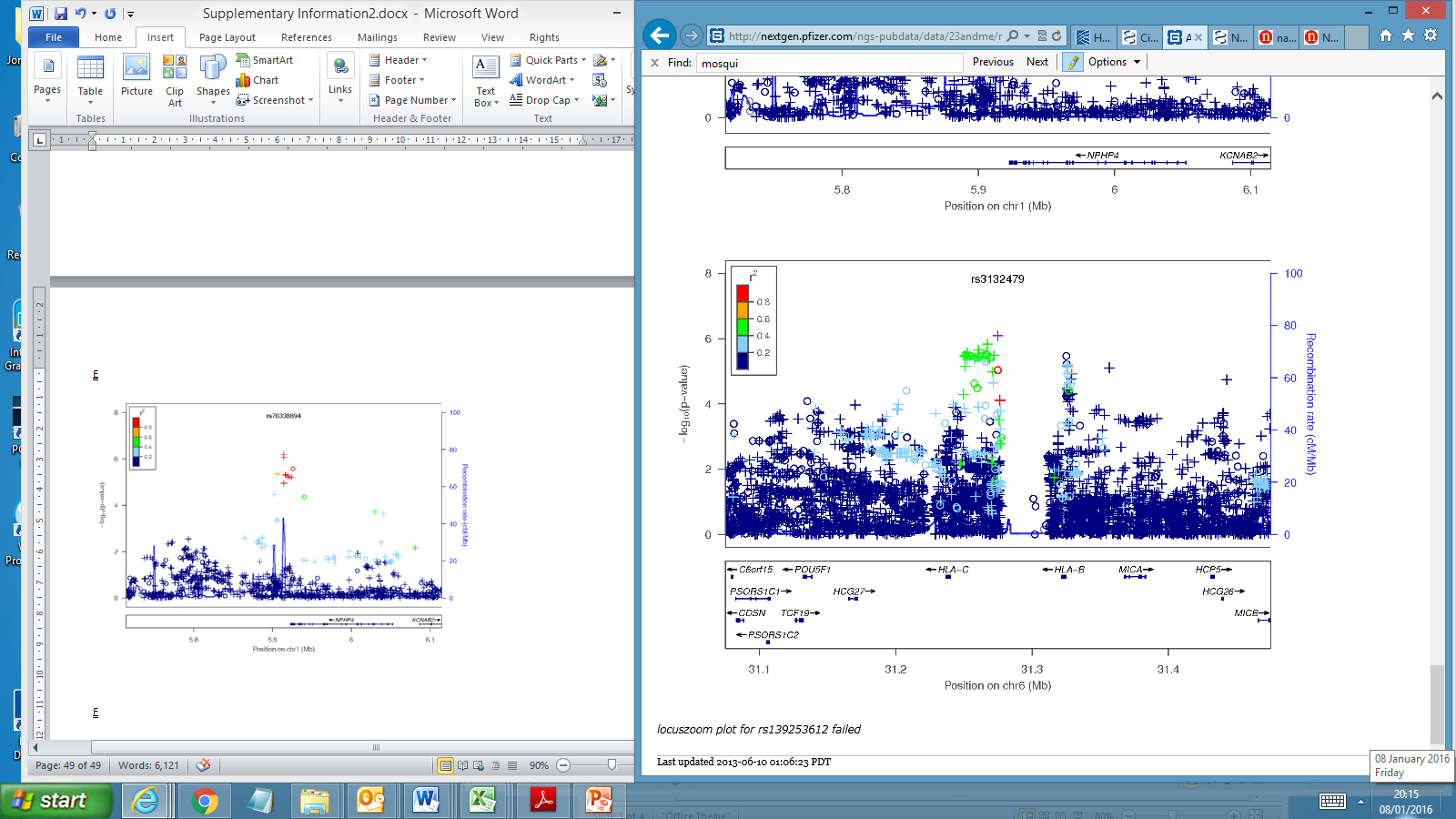


**Suggestive Associations with mosquito bite size**

A sub-GWS association identified for mosquito bite size (rs3024971, *P=*5.9×10^−8^, flanking *STAT6*), has been previously linked to concentration of plasma IgE (2), and also identified as risk factor for allergic sensitisation (3).

Genetic associations identified at *IL6R* on 1q21.1 with bigger mosquito bite sizes and greater mosquito itch intensity (rs12133641; *P* =4.3x10^‑7^ and *P* =2.5x10^-5^, and respectively). This index variant has previously been identified as risk factors for rheumatoid arthritis (4), asthma (5), and ankylosing spondylitis (6), and correlates with increased levels of IL-6 soluble receptor levels present in serum plasma from healthy individuals (7). Further investigation shows this SNP is in high LD (r^2^ =0.97) with rs2228145, a missense polymorphism which encodes D358A, located at cleavage site in the protein shown to be critical for release of the soluble form of the receptor. This rs2228145 variant was recently identified as a risk factor for development of AD (8, 9).

We observed two sub-GWS associations with bigger mosquito bite size, at chr1p36.22 co-localising with *TNFRSF8* (rs2230624, *P*=1.1×10^−7^), and at 9q32 near *TNFSF8* (rs2075533, *P*=4.7×10^−7^). These genes encode the CD30 receptor and the CD30 ligand (CD135), respectively. The CD30 receptor and its ligand are members of the TNF superfamily of cytokines. Both are expressed at low levels in resting lymphocytes, monocytes and NK cells but are upregulated in chronic inflammatory conditions, including lupus, asthma, and AD (10). On further inspection, the *TNFRSF8* index variant encodes a rare (MAF 0.01) nonsynonymous polymorphism, C273F and is an eQTL for *TNFSF8* in peripheral blood leukocytes.

We identified a sub-significant association at 3p28 with bite size, at *LPP* the gene for the lipoma-preferred partner protein (LPP, rs9815073, *P*= 1.1×10^−7^). LPP is an actin cytoskeletal protein, and has been previously linked by GWAS to risk of allergic sensitisation (11), vitiligo (12) and celiac disease (13).

In the mosquito bite size analysis, there was sub-significant variation upstream of *HRH2* which encodes the histamine receptor H_2_ (rs112724722; *P*=5.3x10^-7^). The index variant is in high LD (r^2^>0.8) with SNPs located intronic to *HRH2* and which also co-localise with promoter and enhancer histone marks in leukocytes, suggesting a possible functional role in transcriptional regulation. Histamine is a well-characterised, important inflammatory mediator that controls many physiological functions. Histamine has four receptors whose expression varies by cell type, with the H_2_ receptor being linked to gastric acid secretion (14). The H_1_ receptor is a key therapeutic target for allergic conditions, however it is the H_4_ receptor gene that has been suggested to harbour genetic risk factors for asthma (15), and AD (16).

The variant rs200872239 was correlated with bite size (*P*=6.8x10^-7^), and is situated intronic to *RUNX3.* RUNX is an important transcription factor, critical for development of myeloid immune cells (17).

Bite size GWAS also identified a sub-GWS association in close proximity to, and an eQTL for *IL-10* (rs6673928, *P*=2.1x10^-5^). IL-10 is a key cytokine in the mast cell response to mosquito saliva antigens (18).

**Suggestive Associations with mosquito itch intensity**

A sub-significant association at chr19p13.3 for mosquito itch intensity co-localised with the *FUT6* locus (rs778798, *P*= 8.6×10^−8^). Variants in high LD (r^2^ >0.8) flank the *NTRN* gene, which encodes neuturin, a prominent neurotropic growth factor. Whilst *FUT6* is not expressed in either immune cells, neuronal or epidermal tissue, *NRTN* is expressed by various leukocytes, and is upregulated following stimulation by IFN-γ and lipopolysaccharide (19). Intradermal injection of NGF in mice sensitises pruriceptors and enhances itch response (20). The canonical neuturin receptor GRFA2 is highly expressed by sensory neurons, and single mouse dorsal root ganglia RNA-sequencing transcriptome analysis identified GFRA2 as an important marker for a specific itch responsive neuronal subtype (21).

Another sub-significant loci for mosquito itch intensity (rs5796229, *P*=9.1×10^−8^) at 12p13.3 located intergenic between *SCNN1A* and *LTBR*, genes that could both be plausible functional candidates. *SCNN1A* encodes a subunit in the non-voltage gated epithelial sodium channel (ENaC) complex, and is expressed in neuronal tissue. In invertebrates, ENaC signalling is involved in the function of mechanically activated sensory neurons, in the context of mechanical and thermal nociceptive stimuli (22). *LTBR* encodes the lymphotoxin β receptor, a member of the TNF superfamily, an important player contributing to the complexity of immune cell communication, especially in the context of antigen recognition and education of lymphocytes in lymph nodes. LTβR is expressed by stromal and mesenchymal cells, follicular dendritic cells, and monocytes, where its counterpart ligands LIGHT and LTαβ are expressed by activated T, B and NK cells (23).

A sub-GWS association was identified at chr1q21.3 between rs12123821 and increased itch intensity (*P*= 1.14x10^-5^). The index SNP is located intronic to *HRNR*, a gene that encodes the hornerin protein. Hornerin is thought to play a role in skin barrier function, as inferred from expression data from skin epidermis from normal individuals and also AD cases (24). A variant in the vicinity of *HRNR* has been previously identified as a risk factor for AD (25), however our mosquito itch locus is not in LD with this AD-risk SNP. The AD risk SNP is in high LD with a small number of nonsynonymous SNPs in both *HRNR* and also in the *FLG* gene, which encodes the protein filaggrin. Linkage studies in familial AD have previously found nonsense mutations in *FLG* at chr1q21.3 to be causal for disease (26). Both *HRNR* and *FLG* are within a gene region on chr1q21 referred to as the ‘epidermal differentiation complex’ named accordingly due to an enrichment for genes required for epidermal structure and function (27).

**Supplementary References**

1. Pruim RJ*, et al.* LocusZoom: regional visualization of genome-wide association scan results. *Bioinformatics (Oxford, England)* **26**, 2336-2337 (2010).

2. Granada M*, et al.* A genome-wide association study of plasma total IgE concentrations in the Framingham Heart Study. *The Journal of allergy and clinical immunology* **129**, 840-845.e821 (2012).

3. Bonnelykke K*, et al.* Meta-analysis of genome-wide association studies identifies ten loci influencing allergic sensitization. *Nature genetics* **45**, 902-906 (2013).

4. Okada Y*, et al.* Genetics of rheumatoid arthritis contributes to biology and drug discovery. *Nature* **506**, 376-381 (2014).

5. Ferreira MA*, et al.* Identification of IL6R and chromosome 11q13.5 as risk loci for asthma. *Lancet (London, England)* **378**, 1006-1014 (2011).

6. Cortes A*, et al.* Identification of multiple risk variants for ankylosing spondylitis through high-density genotyping of immune-related loci. *Nature genetics* **45**, 730-738 (2013).

7. Ferreira RC*, et al.* Functional IL6R 358Ala allele impairs classical IL-6 receptor signaling and influences risk of diverse inflammatory diseases. *PLoS Genet* **9**, e1003444 (2013).

8. Esparza-Gordillo J*, et al.* A functional IL-6 receptor (IL6R) variant is a risk factor for persistent atopic dermatitis. *The Journal of allergy and clinical immunology* **132**, 371-377 (2013).

9. Paternoster L*, et al.* Meta-analysis of genome-wide association studies identifies three new risk loci for atopic dermatitis. *Nature genetics* **44**, 187-192 (2012).

10. Oflazoglu E, Simpson EL, Takiguchi R, Grewal IS, Hanifin JM, Gerber HP. CD30 expression on CD1a+ and CD8+ cells in atopic dermatitis and correlation with disease severity. *European journal of dermatology : EJD* **18**, 41-49 (2008).

11. Hinds DA*, et al.* A genome-wide association meta-analysis of self-reported allergy identifies shared and allergy-specific susceptibility loci. *Nature genetics* **45**, 907-911 (2013).

12. Jin Y*, et al.* Variant of TYR and autoimmunity susceptibility loci in generalized vitiligo. *The New England journal of medicine* **362**, 1686-1697 (2010).

13. Dubois PC*, et al.* Multiple common variants for celiac disease influencing immune gene expression. *Nature genetics* **42**, 295-302 (2010).

14. MacGlashan D, Jr. Histamine: A mediator of inflammation. *The Journal of allergy and clinical immunology* **112**, S53-59 (2003).

15. Simon T*, et al.* Asthma endophenotypes and polymorphisms in the histamine receptor HRH4 gene. *International archives of allergy and immunology* **159**, 109-120 (2012).

16. Yu B*, et al.* Polymorphisms in human histamine receptor H4 gene are associated with atopic dermatitis. *The British journal of dermatology* **162**, 1038-1043 (2010).

17. Puig-Kroger A, Corbi A. RUNX3: a new player in myeloid gene expression and immune response. *Journal of cellular biochemistry* **98**, 744-756 (2006).

18. Depinay N, Hacini F, Beghdadi W, Peronet R, Mecheri S. Mast cell-dependent down-regulation of antigen-specific immune responses by mosquito bites. *Journal of immunology (Baltimore, Md : 1950)* **176**, 4141-4146 (2006).

19. Vargas-Leal V, Bruno R, Derfuss T, Krumbholz M, Hohlfeld R, Meinl E. Expression and function of glial cell line-derived neurotrophic factor family ligands and their receptors on human immune cells. *Journal of immunology (Baltimore, Md : 1950)* **175**, 2301-2308 (2005).

20. Rukwied RR, Main M, Weinkauf B, Schmelz M. NGF sensitizes nociceptors for cowhage- but not histamine-induced itch in human skin. *The Journal of investigative dermatology* **133**, 268-270 (2013).

21. Usoskin D*, et al.* Unbiased classification of sensory neuron types by large-scale single-cell RNA sequencing. *Nature neuroscience* **18**, 145-153 (2015).

22. Ben-Shahar Y. Sensory functions for degenerin/epithelial sodium channels (DEG/ENaC). *Advances in genetics* **76**, 1-26 (2011).

23. McCarthy DD, Summers-Deluca L, Vu F, Chiu S, Gao Y, Gommerman JL. The lymphotoxin pathway: beyond lymph node development. *Immunologic research* **35**, 41-54 (2006).

24. Wu Z*, et al.* Highly complex peptide aggregates of the S100 fused-type protein hornerin are present in human skin. *The Journal of investigative dermatology* **129**, 1446-1458 (2009).

25. Esparza-Gordillo J*, et al.* A common variant on chromosome 11q13 is associated with atopic dermatitis. *Nature genetics* **41**, 596-601 (2009).

26. Palmer CN*, et al.* Common loss-of-function variants of the epidermal barrier protein filaggrin are a major predisposing factor for atopic dermatitis. *Nature genetics* **38**, 441-446 (2006).

27. Mischke D, Korge BP, Marenholz I, Volz A, Ziegler A. Genes encoding structural proteins of epidermal cornification and S100 calcium-binding proteins form a gene complex ("epidermal differentiation complex") on human chromosome 1q21. *The Journal of investigative dermatology* **106**, 989-992 (1996).
